# Understanding Pain: From Cells to Brain to Individual Perceptions

**DOI:** 10.1080/24740527.2019.1599266

**Published:** 2019-04-02

## Clinicians’ Perception of a Tailored Web-Based Intervention for the Self-Management of Pain After Cardiac Surgery

Géraldine Martorella^a^, Lucinda Graven^a^, Glenna Schluck^a^, Mélanie Bérubé^b^, and Céline Gélinas^b^

^a^College of Nursing, Florida State University, Tallahassee, Florida, USA; ^b^Ingram School of Nursing, McGill University, Montreal, Quebec, Canada

**CONTACT** Geraldine Martorella gmartorella@fsu.edu

© 2019 The Author(s). Published with license by Taylor & Francis Group, LLC.

This is an Open Access article distributed under the terms of the Creative Commons Attribution License (http://creativecommons.org/licenses/by/4.0/), which permits unrestricted use, distribution, and reproduction in any medium, provided the original work is properly cited.

**Introduction/Aim**: Cardiac surgeries rank among the most frequent surgical procedures and present a risk of chronic post-surgical pain (CPSP). As a first step in preventing CPSP, a tailored Web-based intervention was developed and successfully tested to tackle pain management during hospitalization. Before proceeding to further development, preliminary acceptability of the intervention must be evaluated. The purpose of this study was to examine clinicians’ perception of a Web-based tailored intervention for pain management in the early recovery phase.

**Methods**: A parallel mixed methods approach was used to assess clinicians’ acceptability of the intervention in the early recovery phase (first month after surgery).

**Results**: 249 participants completed an online survey and 10 participants were individually interviewed. Overall, the intervention was rated as acceptable for the early recovery phase. No difference was found in acceptability ratings by demographics. The intervention was rated as appropriate by 79% of participants. Although clinicians seemingly would recommend it to their patients, they did not perceive that their patients would be as highly willing to use it. Interviews highlighted several strengths of the intervention, such as postoperative pain awareness, customization of content, and flexible dosage and schedule. However, the main weakness was related to patient adherence.

**Discussion/Conclusions**: Opting for a hybrid format and integrating individual treatment preferences could enhance the coaching experience. Considering that the intervention has demonstrated positive effects on the pain experience in the first week after cardiac surgery, it seems logical to explore its potential impact after discharge on the transition to CPSP.

## Tailored Web-Based Interventions for Pain: Systematic Review and Meta-Analysis

Geraldine Martorella^a^, Madalina Boitor^b^, Mélanie Bérubé^b^, Suzanne Fredericks^c^, Sylvie Le May^d^, and Céline Gélinas^b^

^a^College of Nursing, Florida State University, Tallahassee, Florida, USA; ^b^Ingram School of Nursing, McGill University, Montreal, Quebec, Canada; ^c^Daphne Cockwell School of Nursing, Ryerson University, Toronto, Ontario, Canada; ^d^Faculty of Nursing, University of Montreal, Montreal, Quebec, Canada

**CONTACT** Geraldine Martorella gmartorella@fsu.edu

© 2019 The Author(s). Published with license by Taylor & Francis Group, LLC.

This is an Open Access article distributed under the terms of the Creative Commons Attribution License (http://creativecommons.org/licenses/by/4.0/), which permits unrestricted use, distribution, and reproduction in any medium, provided the original work is properly cited.

**Introduction/Aim**: Alternative intervention formats have been implemented for people with pain. Several systematic reviews on Web-based interventions for pain have been conducted but the contribution of tailored Web-based interventions has not been evaluated.

The aim was to examine the effects of tailored Web-based interventions for adults compared to usual care, face to face and standardized Web-based interventions on pain intensity? The effects on physical and psychological functions were also assessed.

**Methods**: A systematic review was conducted (January 2000-December 2015). The DerSimonian-Laird random effects models were used to calculate effect estimates. Five outcomes were evaluated: pain intensity, pain-related disability, anxiety, depression and pain catastrophizing. Three time intervals were selected: short [<1 month], medium [1–6 months], and long-term [6–12 months] effects.

**Results**: After full-text review, 17 studies were eligible. Only one study concerning acute pain was removed from the meta-analysis. When compared to usual care, benefits were observed immediately after with small effect sizes (<0.40) for pain intensity (N = 1310, P = 0.003) and pain-related disability (N = 953, P < 0.001). No improvements were observed at follow-up. When compared to the active control group, no improvements were found, except for a small effect size on pain catastrophizing (N = 333, P < 0.001) immediately after the intervention.

**Discussion/Conclusions**: Tailored Web-based interventions did not prove to be more efficient than standardized Web-based interventions. Some efficacy was shown on pain catastrophizing when compared to active control interventions. Considering the diversity of tailored Web-based interventions for chronic pain, their efficacy is yet to be explored. Moreover, their contribution to acute pain management is embryonic.

## Validation of the Critical-Care Pain Observational Tool (CPOT) to Detect Oropharyngeal Pain in Mechanically Ventilated Adults

Craig M. Dale^a^, Virginia Prendergast^b^, Céline Gélinas^c^, and Louise Rose 0000-0003-1700-3972^d^

^a^Lawrence S. Bloomberg Faculty of Nursing, University of Toronto, Toronto, Ontario, Canada; ^b^Barrow Neurological Institute, Phoenix, Arizona, USA; ^c^Ingram School of Nursing, McGill University, Montreal, Quebec, Canada; ^d^Department of Critical Care Medicine, Sunnybrook Health Sciences Centre, Toronto, Ontario, Canada

**CONTACT** Craig M. Dale craig.dale@utoronto.ca;@craig_dale1

© 2019 The Author(s). Published with license by Taylor & Francis Group, LLC.

This is an Open Access article distributed under the terms of the Creative Commons Attribution License (http://creativecommons.org/licenses/by/4.0/), which permits unrestricted use, distribution, and reproduction in any medium, provided the original work is properly cited.

**Introduction/Aim**: To test the reliability and validity of the Critical-Care Pain Observation Tool (CPOT) to detect oropharyngeal pain in critically ill mechanical ventilated adults during routine oral care procedures.

**Methods**: We conducted a prospective observational study in two intensive care units (ICU) in a university-affiliated hospital in Toronto, Canada. Two trained research coordinators independently observed patient behaviors during 2 non-painful (rest and gentle touch) and 3 potentially painful (oral suctioning, tooth brushing, and swabbing with a sponge toothette) oral procedures using the CPOT. Patients were stratified by level of consciousness. We used standard procedures to evaluate criterion validation, discriminative validation, and inter-rater reliability.

**Results**: We recruited 98 patients, primarily intubated (92. 9%), and male (63. 3%). The proportion of patients with CPOT scores >2 indicating pain presence during oral procedures were: oral suction (42.9%); oral swabbing (38.7%); and tooth brushing (29.7%). Level of consciousness did not influence CPOT scores (F = 2.75; P = .07). Criterion validity of the CPOT to detect pain presence was supported during tooth brushing (AUC 0. 80, p = 0. 05) and oral suction (AUC 0. 72, p = 0. 03) but not oral swabbing (AUC 0. 68, p = 0. 16). Mean CPOT scores were significantly higher in the 3 painful exposures compared to the rest conditions demonstrating discriminative validation. Inter-rater reliability was excellent for total CPOT scores (ICC = .78-.91).

**Discussion/Conclusions**: The CPOT is valid and reliable for the detection of oropharyngeal pain during tooth brushing and oral suctioning procedures in adults experiencing invasive mechanical ventilation.

## Effects of Vitamin B_12_ and Ketorolac on Pain in Long Evans Rats

Mizanur Rahman^a^, Noorzahan Begum^b^, Taskina Ali^b^, Mahadi Abdur Rouf^c^, and Shahriar Masood^d^

^a^Department of Physiology, Enam Medical College, Savar, Bangladesh; ^b^Department of Physiology, Bangabandhu Sheikh Mujib Medical University (BSMMU), Dhaka, Bangladesh; ^c^Department of Physiology, Northern International Medical College, Dhaka, Bangladesh; ^d^Department of Physiology, Jahurul Islam Medical College, Bajitpur, Kishoregonj, Bangladesh

**CONTACT** Mizanur Rahman mizandr001@gmail.com Department of Physiology, Enam Medical College, Bangladesh

© 2019 The Author(s). Published with license by Taylor & Francis Group, LLC.

This is an Open Access article distributed under the terms of the Creative Commons Attribution License (http://creativecommons.org/licenses/by/4.0/), which permits unrestricted use, distribution, and reproduction in any medium, provided the original work is properly cited.

**Introduction**: Effects of vitamin B_12_ on pain have been demonstrated in different animal and human studies. But comparison of these effects with similar effects of ketorolac tromethamine (KT) and their combination have not been established. To assess the effects of vitamin B_12_ on pain and also to compare them with those of the combinations of vitamin B_12_ with KT in rat models.

**Methods**: This experimental study was conducted in the Department of Physiology, Bangabandhu Sheikh Mujib Medical University, Dhaka, from March 2015 to February 2016. For this, 20 (twenty) Long Evans rats (215 ± 35 gm) of both sexes (Ref: Banode SV, Borkar AS, Badwaik RT. Effect of ketorolac on opioid induced antinociception in rats. IJMPS. 2012;3:7–13) were divided into control (A, with 5 ml/kg normal saline Ref: Moallem SA, Hosseinzadeh H, Farahi S. A study of acute and chronic anti-nociceptive and antiinflammatory effects of thiamine in mice. Iran Biomed J. 2008; 12 (3):173-178.) and experimental (B1, with 15 mg/kg B_12_; B2, with 10 mg/kg KT; B3, with B_12_+ KT) (Ref: Banode SV, Borkar AS, Badwaik RT. Effect of ketorolac on opioid induced antinociception in rats.IJMPS. 2012; 3(3):7-13; Imtiaz M. Effect of vitamin B12 and folic acid and their combination on pain and inflammation in rats.[Thesis] [Dhaka (Bangladesh)]: BSMMU. 2011.) groups with 5 rats in each group. All the drugs and vitamin were administered intraperitoneally (Ref: Kim MJ, Hong BH, Zhang EJ, Ko YK, Lee WH. Antinociceptive effects of intraperitoneal and intrathecal vitamin E in rat formalin test. Korean Journal of Pain. 2012; 25(4):238-244.) in a single dose just one hour before formalin test. Statistical analysis was done by ANOVA, followed by Bonferroni post hoc test. In the interpretation of results, p ≤ 0.05 was considered as significant.

**Results**: B_12_ lowered only the jerking frequency and KT lowered both jerking frequency and flexing-licking duration significantly (p ≤ 0.001) in the late phase of formalin test. On the other hand, combination of B_12_ and KT significantly (p ≤ 0.001) lowered both the study variables in all 3 phases of formalin test.

**Conclusion**: From this study it may be concluded that, vitamin B_12_ possess analgesic effects and combination of B_12_ with KT is more effective than those of their individual administration.

**Disclosure statement**

No potential conflict of interest was reported by the authors.

**Figure 1.**
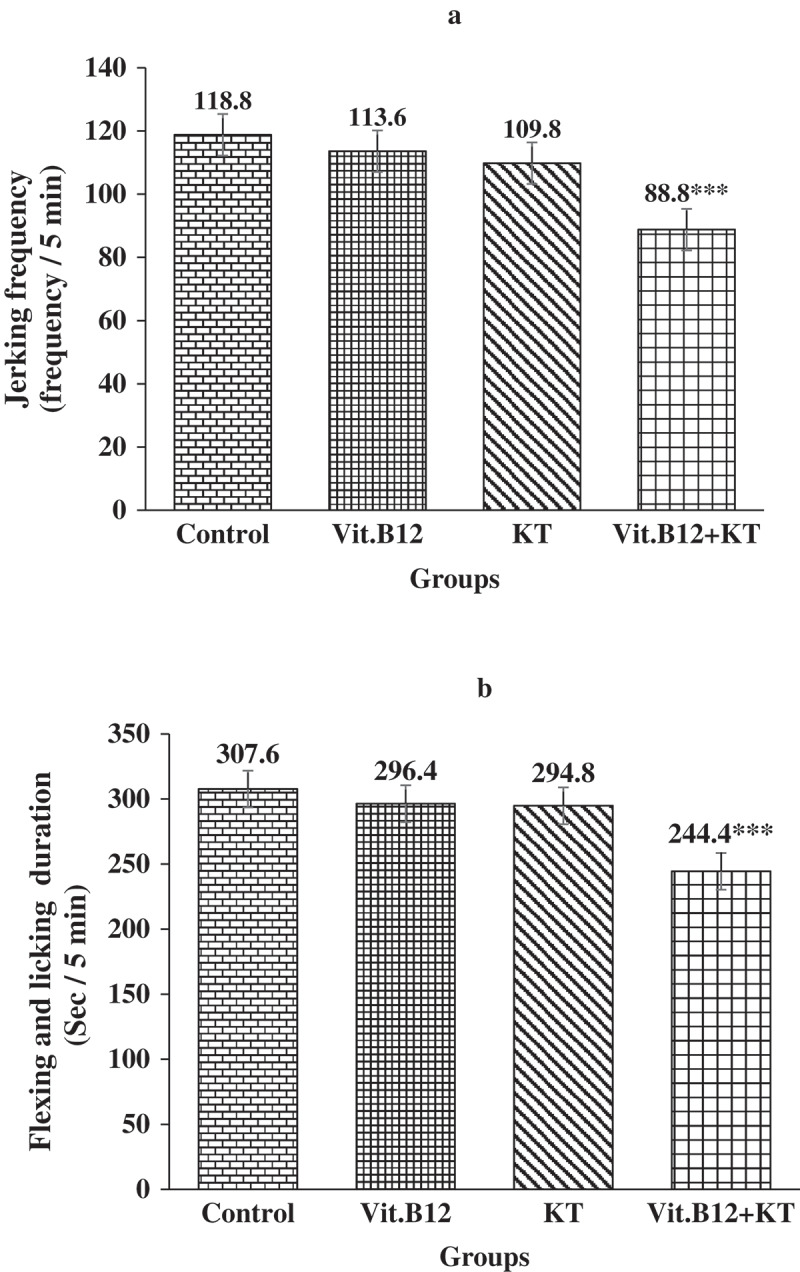
Frequency of jerking (A) and duration of flexing and licking (B) in early phase of formalin test in different groups of rats. Each bar symbolizes for mean ± SE for 5 rats. *** = p ≤ 0.001, compared to those of control. KT = Ketorolac tromethamine.

**Figure 2.**
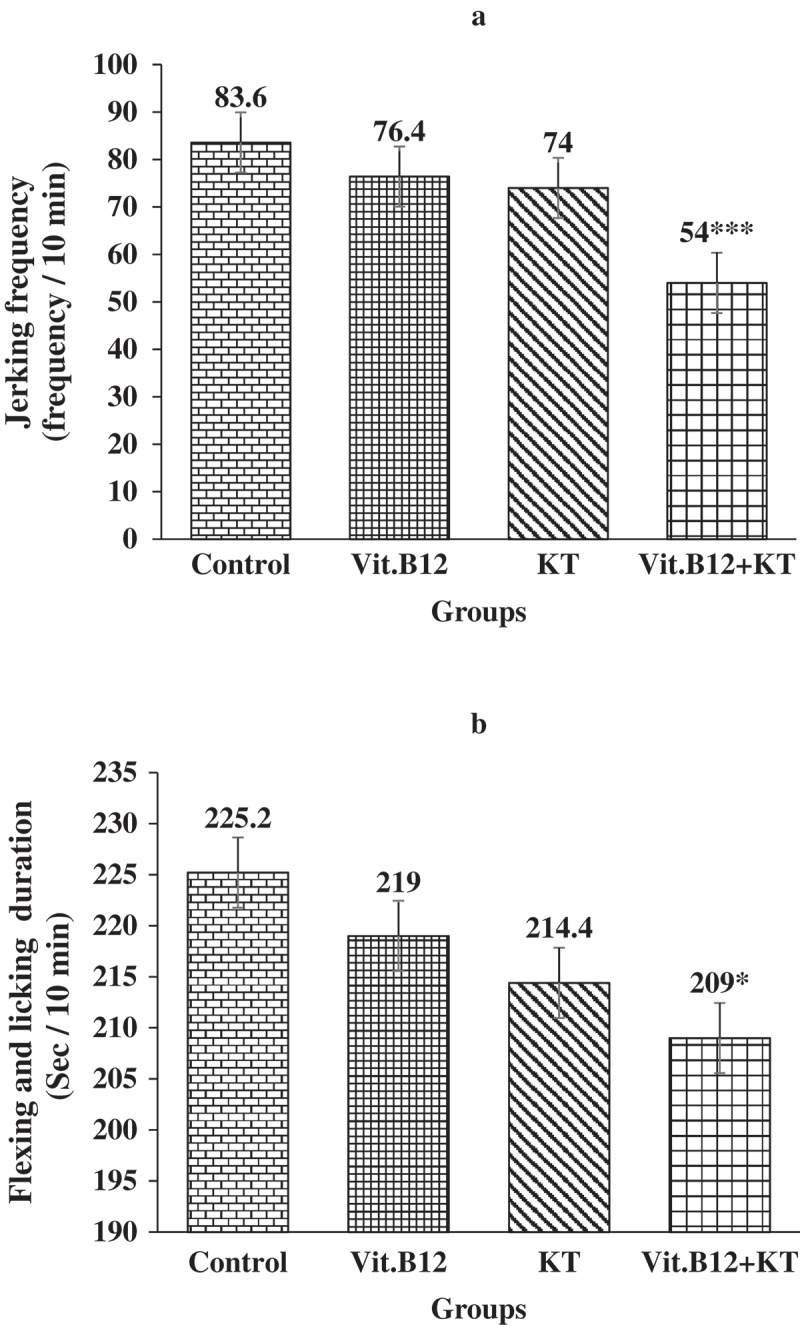
Frequency of jerking (A) and Duration of flexing and licking (B) in the interphase of formalin test in different groups of rats. Each bar symbolizes for mean ± SE for 5 rats. *** = p ≤ 0.001 and * = p ≤ 0.05 compared to those of control. KT = Ketorolac tromethamine.

**Figure 3.**
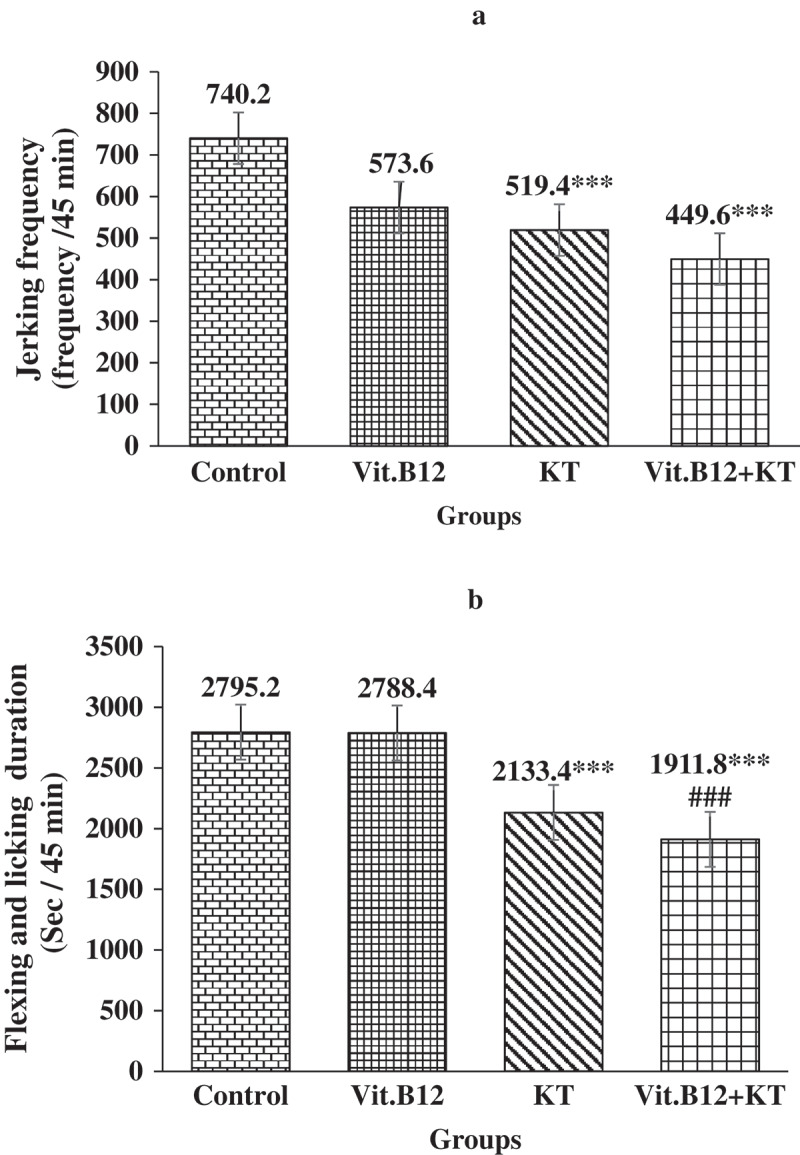
Frequency of jerking (A) and duration of flexing and licking (B) in late phase of formalin test in different groups of rats. Each bar symbolizes for mean ± SE for 5 rats. *** = p ≤ 0.001, compared to control and ### = p ≤ 0.001, comparison between only KT vs KT + Vit.B_12._ KT = Ketorolac tromethamin.

References1.
Imtiaz
M
Effect of vitamin B_12_ and folic acid and their combination on pain and inflammation in rats.[Thesis] [
Dhaka (Bangladesh)]:
BSMMU. 2011.2.
Banode
SV, Borkar
AS, Badwaik
RT.
Effect of ketorolac on opioid induced antinociception in rats. IJMPS. 2012;3:7–13.3.
Moallem
SA, Hosseinzadeh
H, Farahi
S. A study of acute and chronic anti-nociceptive and anti-inflammatory effects of thiamine in mice. Iran Biomed J. 2008;12:173–78.187628214.
Kim
MJ, Hong
BH, Zhang
EJ, Ko
YK, Lee
WH. Antinociceptive effects of intraperitoneal and intrathecal vitamin E in rat formalin test. Korean J Pain. 2012;25(4):238–44. doi:
10.3344/kjp.2012.25.4.238.23091684PMC3468800

## DELTA-9-Tetrahydrocannabinol in Neuropathic Pain and Comorbid Insomnia

Martha López-Canul^a^, Alexandra Teggin^a^, Maria-Luisa Vigano^a^, Luca Posa^a^, Danilo De Gregorio^a^, Shelly Yin^a^, and Gabriella Gobbi^a^

Department of Psychiatry Montreal, McGill University, Québec, Canada

**CONTACT** Martha López-Canul martha.lopezcanul@mcgill.ca

© 2019 The Author(s). Published with license by Taylor & Francis Group, LLC.

This is an Open Access article distributed under the terms of the Creative Commons Attribution License (http://creativecommons.org/licenses/by/4.0/), which permits unrestricted use, distribution, and reproduction in any medium, provided the original work is properly cited.

**Introduction/Aim**: Neuropathic pain (NP) is a major health problem characterized by high suffering, low productivity and substantial health care costs. An estimated 50% of patients with NP develop sleep disturbances (Finan et al., 2013) and only a restricted number of effective drugs are clinically available for NP associated to insomnia with several side effects. The delta-9-tetrahydrocannabinol (THC) may offer a novel approach to the chronic pain management and insomnia, but its efficacy in these two condition is not yet fully understood.

**Methods**: We induced NP (sciatic nerve ligation) in Wistar rats, 14 days later were treated with vehicle (VEH; 5%Tween 80, 5% PEG and saline) or THC (1, 1.5, 2, 2.5 and 5 mg.kg, i.p.) to assess mechanical allodynia. Then, rats with NP (sciatic nerve injury) were implanted with six stainless-steel wire electrodes in the skull and EEG/EMG was recorded for a period of 6 h (from 6 AM to 12 PM).

**Results**: THC (2.5 and 5 mg/kg) decreased mechanical allodynia in rats with NP (p < 0.001) vs VEH, 1h. after administration in a dose dependent-manner. This effect lasted about 3.5 hours. EEG/EMG analysis showed that rats with NP had a reduced time in non-rapid eyes movement (NREM) sleep (−35%, p < 0.05) and increased wakefulness (+39.6%, p < 0.05) compared to naive rats. THC (5 mg/kg) was able to restore the normal sleep-wake cycle in NP rats.

**Discussion/Conclusions**: The findings suggest that THC has a moderate analgesic effect and good hypnotic effect in a NP paradigm, similar to clinical outcomes reported in humans.

## Retrospective Review of the Use of Transdermal Buprenorphine Patches (Butrans) in a Pediatric Population

Michael Smyth^a^, Sebastian Haupt^a^, and Marie-Claude Grégoire^b^

^a^Faculty of Medicine, Dalhousie University, Halifax, Nova Scotia, Canada; ^b^Department of Pediatrics, Dalhousie University, Halifax, Nova Scotia, Canada

**CONTACT** Michael Smyth m.smyth@dal.ca

© 2019 The Author(s). Published with license by Taylor & Francis Group, LLC.

This is an Open Access article distributed under the terms of the Creative Commons Attribution License (http://creativecommons.org/licenses/by/4.0/), which permits unrestricted use, distribution, and reproduction in any medium, provided the original work is properly cited.

While indicated for use in adult populations, small studies have shown promising results in reducing pain in children with few, if any, adverse effects. The objective of this study was to evaluate the safety of a Butrans (buprenorphine) Transdermal System patch for analgesia in a palliative paediatric setting. Studies of this drug have been mostly limited to adult populations and information regarding transdermal administration of buprenorphine in a paediatric population is predominantly found in case reports. Given this, our aim was to retrospectively assess the safety of buprenorphine in a palliative paediatric care setting. In our patient population, 27.3% of patients experienced only mild adverse drug reactions in the form of skin irritation (erythema in combination with pruritus), which was resolved with topical steroid treatment. The development of skin irritation led to Butrans treatment cessation in only 9.1% of subjects. Given these promising results, Butrans was found to be appropriate for analgesic use in a palliative paediatric setting and hope to see it’s use expanded in the future.

**Introduction/Aim**: Buprenorphine is an opioid medication used for the treatment of moderate to severe pain. In Canada, buprenorphine is not indicated for use in the pediatric population and literature surrounding its use in pediatrics is limited. Our aim was to evaluate the safety of buprenorphine in a pediatric palliative care setting.

**Methods**: Our study was performed at the IWK Health Centre. Medical records of 11 patients were examined for specific clinical characteristics. The study focused primarily on descriptive results; standard data analyses were not performed.

**Results**: Buprenorphine was found to be well tolerated in our patient population. There were no adverse effects reported in 8 of 11 patients during their treatment with buprenorphine. The remaining 3 patients described mild adverse effects in the form of skin irritation which resolved with topical steroid treatment. Efficacy was reported as anecdotal quotes from patient records.

**Discussion/Conclusions**: Currently in medical literature, there is limited data on the safety of buprenorphine in children suffering from pain in a palliative care setting. In our study, the use of buprenorphine in control of pain in this setting was safe in a small group of patients (3 patients exhibited a contact dermatitis which quickly resolved upon removal of the patch; no other adverse effects noted). Other studies have also demonstrated buprenorphine to be a safe and an effective opioid for the treatment of severe pain at the end of life in a pediatric population. The implementation of buprenorphine in pediatrics may be safe and provide proper analgesia.

## Patients’ Perspectives on Methods of Assessing Pain

Allison Verge^a^, and Karim Mukhida^a^

Department of Anesthesiology, Pain Management and Perioperative Medicine, Dalhousie University, Halifax, Nova Scotia, Canada

**CONTACT** Alison Verge allisoncverge@gmail.com

© 2019 The Author(s). Published with license by Taylor & Francis Group, LLC.

This is an Open Access article distributed under the terms of the Creative Commons Attribution License (http://creativecommons.org/licenses/by/4.0/), which permits unrestricted use, distribution, and reproduction in any medium, provided the original work is properly cited.

**Introduction/Aim**: The McGill pain questionnaire serves as a basis for initial consultation assessment tools used by pain clinics throughout Canada. The effectiveness of pain evaluations using multi-page, text-heavy questionnaires has been recognized as less than ideal for patients for a number of reasons (such as reliance on literacy, the format not facilitating patient self-expression or conversation with physicians). Various novel methods of assessing pain have been suggested, including those that use pictograms and photographs and results have shown that this is a valuable area of research to pursue further. However, researchers to date have only trialed newly-designed models on patient populations and have not consulted patients in their design. Patient input on how they could best express their pain to health care professionals is a critical resource in furthering this area of research and innovation. The goal of this study was to gauge patient opinions on current pain assessment modalities and learn their opinions on future areas of improvement.

**Methods**: Thirty patients were interviewed following their initial consultation appointments at the Pain Management Unit in Halifax. Interviews were transcribed verbatim and analyzed using NVivo Software to look for themes among patients.

**Results**: The study yielded a total of twenty-five themes, such as the questionnaires were repetitive but of appropriate length. The use of a body template was valued. Participants provided perspectives on the use of technology and art for pain assessment.

**Discussion/Conclusions**: Recommendations are proposed based on the themes to help guide the creation or modification of pain assessment tools.

## Heart[♥-ART-INFORMED] Journey through Cardiac Pain: A Qualitative Artistic Interpretation

Sheila O’Keefe-McCarthy^a^, Karyn Taplay^a^, Lisa Keeping-Burke^b^, Allison Flynn-Bowman^a^, Maria Vasilaki^a^, and Jenn Salfi^a^

^a^Department of Nursing, Brock University, St. Catharines, Ontario, Canada; ^b^Department of Nursing & Health Sciences, University of New Brunswick, Saint John, New Brunswick

**CONTACT** Sheila O’Keefe-McCarthy sokeefemccarthy@brocku.ca

© 2019 The Author(s). Published with license by Taylor & Francis Group, LLC.

This is an Open Access article distributed under the terms of the Creative Commons Attribution License (http://creativecommons.org/licenses/by/4.0/), which permits unrestricted use, distribution, and reproduction in any medium, provided the original work is properly cited.

**Introduction/Aim**: Recognition of early ischemic cardiac pain symptoms is fraught with uncertainty and fear. Highlighting the experiential qualities of individual patient experiences of ischemic cardiac disease in a creative and artistic encounter, enhances the clinician’s critical awareness of the patient experience of living with heart disease.

**Methods**: We employed an arts-based creative research embodied analysis to translate the experiences of 23 individuals’ journeys through symptom recognition of coronary artery disease. Four focus groups and 12 individual qualitative semi-structured interviews were conducted, audio-recorded and transcribed verbatim. Qualitative description was used to generate categories and themes.

**Results**: Denial and disbelief, self-recrimination, and encroaching symptoms of heart disease were themes that emerged from the data. Seminal words and phrases were extracted from the interviews and constructed into poetry. Patients’ stories were further conceptualized and informed the artistic creations and painted images that metaphorically represented the participants’ cardiovascular pain and illness experiences.

**Discussion/Conclusions**: Arts-informed research provides an empathetic, compassionate and embodied introspection and reflection from the patient’s perspective. It has the power to engage clinicians at a visceral insightful level that allows meaning to be transferred differently, patient stories to be heard, visualized, and felt. The phenomena of cardiac pain as the background, creates an artful place and space to contemplate the emotional journey living with cardiac disease.

## Design of an Emergent Acute Heart Pain Application: Patient and Clinician Perspectives

Sheila O’Keefe-McCarthy 0000-0003-2909-8286^a^, Karyn Taplay^a^, Lisa Keeping-Burke^b^, Allison Flynn-Bowman^a^, Maria Vasilaki^a^, Jennifer Tsang^c^, Chris Glover^d^, Colleen Norris^e^, and Jennifer Price^f^

^a^Department of Nursing, Brock University, St. Catharines, Ontario, Canada; ^b^Department of Nursing & Health Sciences, University of New Brunswick, Saint John, New Brunswick, Canada; ^c^Niagara Health, Internal Medicine, St. Catharines, Ontario, Canada; ^d^Ottawa Heart Institute, University of Ottawa, Ottawa, Ontario, Canada; ^e^Faculty of Medicine and Dentistry, University of Alberta, Edmonton, Alberta, Canada; ^f^Women’s College Hospital, Cardiology/Rehab, Toronto, Ontario, Canada

**CONTACT** Sheila O’Keefe-McCarthy sokeefemccarthy@brocku.ca Department of Nursing, Brock University, St. Catharines, ON, Canada

© 2019 The Author(s). Published with license by Taylor & Francis Group, LLC.

This is an Open Access article distributed under the terms of the Creative Commons Attribution License (http://creativecommons.org/licenses/by/4.0/), which permits unrestricted use, distribution, and reproduction in any medium, provided the original work is properly cited.

**Introduction/Aim:** Proactive immediate treatment of acute pain is critical in the first hours of acute coronary syndrome (ACS) onset to prevent transition to persistent cardiac pain. Aim: The purpose of this research was to design the Acute-Heart PAiN-APP. There are no known digital health technologies (DHTs) (APPs) developed or evaluated to treat acute cardiac pain during an emergency hospital admission for ACS that are designed by patients and health care providers (HCPs).

**Methods:** In Phase I we conducted qualitative interviews with individuals with ACS and HCPs to determine the preferred content and format for the APP.

**Results:** 18 patients and 4 HCPs suggested that the APP consist of four interactive parts: 1) Coaching through the acute heart pain, 2) Symptom tracking of pain and anxiety, 3) SMART goals to manage the acute symptoms: a) choice of diversion: relaxation-paced breathing, b) guided imagery c) music and d) forms of gaming, and 4) immediate visual representation of pre-and-post APP effect on pain and anxiety levels.

**Discussion/Conclusions:** The Acute-Heart PAiN-APP is an interactive DHT adjuvant treatment for ACS. The APP will permit a therapeutic level of patient control, symptom monitoring and self-management of ACS-pain. More importantly, this intervention may preserve threatened ischemic myocardial muscle, which may circumvent the transition to persistent forms of cardiac pain and related disability.

## Comparison of Older and Younger Patients Referred to a University-Affiliated Community Pain Clinic in the Greater Toronto Area (GTA)

S. Fatima Lakha 0000-0002-3325-7553^a^^b^, Alex Orlovic^a^, Demetry Assimakopoulos^c^, and Angela Mailis^d^^e^^f^

^a^Research Department, Pain and Wellness Center, Vaughan, Ontario, Canada; ^b^Institute of Medical Sciences, University of Toronto, Toronto, Ontario, Canada; ^c^Comprehensive Integrated Pain Program – Rehabilitation Pain Service, University Health Network, Toronto, Ontaria, Canada; ^d^Pain and Wellness Centre, Vaughan, Ontario, Canada; ^e^Department of Medicine, Division of Physical Medicine, University of Toronto, Toronto, Ontario Canada; ^f^Toronto Rehab Institute/University Health Network, Toronto, Ontario, Canada

**CONTACT** S.Fatima Lakha sfatima.lakha@utoronto.ca Research Department, Pain and Wellness Center, Vaughan, Ontario, Canada

© 2019 The Author(s). Published with license by Taylor & Francis Group, LLC.

This is an Open Access article distributed under the terms of the Creative Commons Attribution License (http://creativecommons.org/licenses/by/4.0/), which permits unrestricted use, distribution, and reproduction in any medium, provided the original work is properly cited.

**Introduction/Aim:** To compare demographic and pain characteristics of elderly (>65) vs younger (<65) chronic non-cancer pain (CNCP) patients referred to a university-affiliated community pain clinic in GTA, Ontario.

**Methods:** This is a retrospective study of 644 consecutive new CNCP patients (elderly n = 126 and younger n = 518) seen during 2016–2017. Demographic characteristics, Brief Pain Inventory (BPI) pain ratings, and diagnosis were obtained using retrospective chart review. Patients were classified in Group I (pure biomedical pathology), Group II (mixed biomedical causes and psychological factors) and Group III (no detectable physical pathology but psychological factors were considered important).

**Results:** Male/female ratio was 1:1.3 vs 1:1.7 and mean age 74 ± 6 vs 45 ± 12 respectively for the elderly vs younger patients. About three-quarters of the elderly were foreign-born (71%) vs the younger population (37%, p < 0.001). Low back/buttocks/hips were the most prevalent site of pain; average BPI pain score (7/10) was similar between the two groups. The majority of the elderly (73%) was classified as group I vs 40% of the younger patients (p < 0.0001). Only 3% of the elderly were classified in Group III vs 16% of the younger population (p < 0.05). Opioids were consumed by 45% of elderly and 34% of younger patients, while current cannabis use for pain was minimal between the elderly (5%) vs 18% of the younger patients (<0.0001).

**Discussion/Conclusions:** Elderly CNCP patients present with greater physical and less psychosocial impairment as opposed to younger patients (similar to previous findings from an academic Toronto pain clinic). Further differences vs similarities will be discussed.

## Identifying Parent Traits that Predict Parent Heart Rate Variability Response to Child Acute Pain: Preliminary Findings

Kaytlin Constantin^a^, Rachel Moline^a^, and C. Meghan McMurtry 0000-0002-3278-1169^a^

Department of Psychology, University of Guelph, Guelph, ON, Canada

**CONTACT** Kaytlin Constantin kaytlin@uoguelph.ca

© 2019 The Author(s). Published with license by Taylor & Francis Group, LLC.

This is an Open Access article distributed under the terms of the Creative Commons Attribution License (http://creativecommons.org/licenses/by/4.0/), which permits unrestricted use, distribution, and reproduction in any medium, provided the original work is properly cited.

**Introduction/Aim:** Parent traits relate to child pain outcomes; however, less clear is how these factors affect parent responses, such as heart rate variability (HRV), which provides continuous information about parents’ emotional states. We examined how parent traits relate to changes in parent HRV before and after their child’s completion of the cold pressor task (CPT).

**Methods:** Children between 7 and 12 years of age completed the CPT in the presence of a primary caregiver (*n* = 43). Parents completed trait measures of State-Trait Anxiety Inventory, Pain Catastrophizing Scale-Parent, and the Emotion Regulation Questionnaire. Parental HRV [assessed by the high-frequency component of HRV (HF-HRV) and the root mean square of the successive differences (RMSSD)] were monitored during 3 × 2 minute time periods: 1) neutral video (resting), 2) prior to the CPT (pre-CPT), and 3) recovery after the CPT (post-CPT). Resting HRV was examined as a trait variable. HRV reactivity was calculated by subtracting resting HRV from HRV pre-CPT. HRV recovery was calculated by subtracting HRV pre-CPT from HRV post-CPT.

**Results:** four hierarchical regression analyses were conducted in which parent traits were entered as predictors of parent HRV reactivity and recovery (HF-HRV, RMSSD). Resting HRV and trait anxiety predicted HRV reactivity; while resting HRV and trait catastrophizing predicted HRV recovery.

**Discussion/Conclusions:** Findings contribute to existing social communication models of pain. This novel study examined how parent traits relate to changes in parent HRV before and after child acute pain. Clarifying the factors contributing to parent responses has implications for pain management interventions.

## Accumulating Risk: Parent Chronic Pain and Trauma Symptoms Predict Poorer Outcomes for Youth with Chronic Pain

Jaimie Beveridge^a^, Richelle Mychasiuk^a^, and Melanie Noel^a^

Psychology, University of Calgary, Calgary, Alberta, Canada

**CONTACT** Jaimie Beveridge jaimie.beveridge@ucalgary.ca

© 2019 The Author(s). Published with license by Taylor & Francis Group, LLC.

This is an Open Access article distributed under the terms of the Creative Commons Attribution License (http://creativecommons.org/licenses/by/4.0/), which permits unrestricted use, distribution, and reproduction in any medium, provided the original work is properly cited.

**Introduction/Aim:** Pediatric chronic pain is prevalent, affecting 25% of Canadian youth, and can have devastating effects on functioning and well-being. In order to improve psychological treatments for youth with chronic pain, which are often ineffective, a better understanding of factors that contribute to pediatric chronic pain, and which may be effective targets for treatment, is needed. Parent chronic pain and trauma have been found individually to contribute to chronic pain in offspring. However, chronic pain and trauma often co-occur. As such, this study aimed to examine the *cumulative* risk of parent chronic pain and trauma symptoms on pediatric chronic pain outcomes.

**Methods:** To date, 172 youth (68% girls) aged 8–18 (*M *= 13.26, *SD *= 2.59) referred for tertiary-level treatment of chronic pain and one of their parents (92% mothers) completed self-report measures of parent chronic pain status and trauma symptoms and child pain interference and health-related quality of life (HRQoL). Hierarchical multiple regressions were conducted.

**Results:** Parent chronic pain status (step 1) and trauma symptoms (step 2) predicted child pain interference (total model: *R^2^* = .08, step 1: ∆*R^2^* = .03, step 2: ∆*R^2^* = .06, all *p*s<.05) and HRQoL (total model: *R^2^ *= .11, step 1: *∆R^2^* = .05, step 2: *∆R^2^* = .06, all *p*s<.05).

**Discussion/Conclusions:** Parents’ own chronic pain and trauma symptoms predicted greater pain interference and lower HRQoL in youth with chronic pain. These findings suggest that parents’ mental and physical health may be important targets for the treatment of pediatric chronic pain. Future research should examine modifiable mechanisms (e.g., parenting behaviours) that may underlie these relationships.

## A Survey of Canadian Pediatric Chronic Pain Clinics: Views on Somatic Symptom Disorder and Related Diagnoses Among Various Health Care Professionals

Kimberly R. Edwards^a^, C. Meghan McMurtry 0000-0002-3278-1169^b^, Bruce Dick^c^, Rebecca Lewinson^d^, and Joel Katz^d^

^a^Department of Psychiatry and Behavioural Neurosciences, McMaster Children’s Hospital, Hamilton, Ontario, Canada; ^b^Clinical Child and Adolescent Psychology, University of Guelph, Guelph, Ontaria, Canada; ^c^Departments of Anesthesiology and Pain Medicine, Psychiatry and Pediatrics, University of Alberta, Edmonton, Alberta, Canada; ^d^Department of Psychology, York University, Toronto, Ontario, Canada

**CONTACT** Kimberly R. Edwards edwardkim@hhsc.ca McMaster Children’s Hospital, Hamilton, Ontario, Canada

© 2019 The Author(s). Published with license by Taylor & Francis Group, LLC.

This is an Open Access article distributed under the terms of the Creative Commons Attribution License (http://creativecommons.org/licenses/by/4.0/), which permits unrestricted use, distribution, and reproduction in any medium, provided the original work is properly cited.

**Introduction/Aim**: Youth with chronic pain (YCP) are at risk of facing co10 morbidities including mood and anxiety disorders. The most controversial diagnoses used with YCP are included within the DSM-5 Somatic symptom and related disorder (SS+) category, which focuses on the presence of one (or more) distressing bodily symptoms 15 (e.g., pain). The primary objective of this study was to examine the perspectives of health care professionals working within pediatric chronic pain clinics across Canada about the use of SS+ diagnoses in YCP.

**Methods**: Fifty participants completed online surveys designed by 20 experts within the fields of pain and somatization.

**Results**: Results indicated that 53% of the sample diagnose within their professional scope and that approximately 17% of YCP in their clinics are diagnosed with Somatic Symptom Disorder (SSD). In contrast to training in 25 chronic pain, most participants reported receiving minimal training in SS+ and were dissatisfied or very dissatisfied with the amount of training received in SS+. When clinicians were asked to rank the top five interventions they use for chronic pain, SSD, and Conversion Disorder (CD), there were no differences 30 with respect to the type of intervention (psychoeducation about the mind-body connection being endorsed as the top intervention across diagnoses). Inductive content analyses revealed substantial complexity in participants’ perceptions of the advantages and disadvan- 35 tages of using SS+ diagnoses in YCP (e.g., whether or not the diagnoses appropriately conceptualize the symptoms).

**Discussion/Conclusions**: Future research is needed to develop guidelines and training initiatives for professionals about the use of SS+ diagnoses in youth with chronic pain.

## Parent Emotional Presence during Child Pain: Examining Parent Emotion Regulation and Mindfulness during their Child’s Cold Pressor Task

Rachel Moline^a^, Kaytlin Constantin^a^, and C. Meghan McMurtry 0000-0002-3278-1169^a^

Department of Psychology, University of Guelph, Guelph, ON, Canada

**CONTACT** Rachel Moline rmoline@uoguelph.ca

© 2019 The Author(s). Published with license by Taylor & Francis Group, LLC.

This is an Open Access article distributed under the terms of the Creative Commons Attribution License (http://creativecommons.org/licenses/by/4.0/), which permits unrestricted use, distribution, and reproduction in any medium, provided the original work is properly cited.

**Introduction/Aim:** While parents can impact child pain outcomes via verbal and nonverbal communication, parents often have difficulty knowing how to respond to their child’s pain. Further, most pain management interventions focus on the child. Determining what parent responses facilitate child coping can inform interventions for parents. This study examined parent emotional and cognitive presence (mindfulness, emotion regulation), parent perceptions of child outcomes, and child pain outcomes during child pain to better understand protective parent factors.

**Methods:** Fifty-six children (7–12 years) underwent the cold pressor task (CPT) alongside their parent. Parents completed trait measures of mindfulness and emotion regulation (Cognitive Affective Mindfulness Scale-Revised; Emotion Regulation Questionnaire). Following the CPT, parents completed a tool to report how they felt during the procedure (1 = very scared; 5 = very relaxed/happy; Parent Awareness of Emotion; PAE). Child pain and fear were collected via child-self and parent-proxy report.

**Results:** Parent fear during the CPT (rated on the PAE) related to increased cognitive reappraisal (*r* = −.28*), higher perceptions of child pain (*r* = −.33*) and fear (*r* = −.47**). Parent fear also related to increased child fear (*r* = −.28*). Parent mindfulness did not relate to parent perceptions of the procedure, but related to reduced child pain (*r* = −.29*). Parent emotional suppression related to increased child fear (*r* = .27*).

**Discussion/Conclusions:** Parent emotional presence is connected to both parent and child report of child pain and fear outcomes during an acutely painful procedure. Results provide preliminary evidence for a parent intervention targeting parent mindfulness and instruction to feel emotions freely (less emotional suppression).

## A Scoping Review of Transdermal Buprenorphine Use for Non-Surgical Pain in the Paediatric Population

Sebastian Haupt^a^, Michael Smyth^a^, and Marie-Claude Grégoire^b^

^a^Faculty of Medicine, Dalhousie University, Halifax, Nova Scotia, Canada; ^b^Department of Paediatrics, Dalhousie University, Halifax, Nova Scotia, Canada

**CONTACT** Sebastian Haupt sebhaupt@dal.ca

© 2019 The Author(s). Published with license by Taylor & Francis Group, LLC.

This is an Open Access article distributed under the terms of the Creative Commons Attribution License (http://creativecommons.org/licenses/by/4.0/), which permits unrestricted use, distribution, and reproduction in any medium, provided the original work is properly cited.

We conducted a preliminary evaluation to review the scope and quality of evidence surrounding transdermal buprenorphine use in the paediatric setting for non-surgical pain. Our review revealed limited data available on the use of transdermal buprenorphine in paediatric patients. Most studies surrounding this subject involve accidental ingestion of buprenorphine and it use in treatment of neonatal abstinence syndrome. While indicated for use only in adult populations, small studies have shown encouraging results in reducing pain in children with few, if any, adverse effects. This is reassuring from a clinical perspective, as we hope to highlight the available evidence and invite researchers to expand future studies. Through this review, we have identified significant gaps in the literature surrounding the safety and use of buprenorphine in the paediatric population. To our knowledge, there are no major studies investigating this subject and it is our hope, from a patient perspective, that future studies will explore the use of transdermal buprenorphine as an alternative pain mangement technique in paediatrics. The intent of our research is to help opioid naïve patients, patients experiencing inadequate analgesia, adverse effects from traditional opioid therapies, or as an alternative route of administration when the enteral or intravenous routes are not available.

**Introduction/Aim:** In Canada and the USA, transdermal buprenorphine is not indicated in the paediatric population despite literature being devoid of any major adverse drug reactions among participants. This study aims to address current gaps in the literature and provide greater understanding of transdermal buprenorphine in the pediatric population.

**Methods:** In this scoping review, PubMed, Embase and CINAHL databases were screened. All studies including case reports (n = 1) were included. We utilized the Preferred Reporting Items for Systematic Reviews and Meta-Analyses (PRISMA), an evidence-based guideline to advance the execution of systematic and scoping reviews. We employed Covidence software, the current gold standard used for Cochrane systematic reviews to screen and filter available literature.

**Results:** Of the 2587 studies screened for transdermal buprenorphine use in the paediatric population, we found that only a small number of studies were eligible for further analysis based on our exclusion criteria. Of the studies analyzed, buprenorphine was found to be a safe and effective treatment for pain in a paediatric population. Furthermore, these studies also found that sustained release of buprenorphine was well tolerated by patients with few adverse reactions (most commonly erythema and allergic contact dermatitis). Other side effects included nausea, vomiting and headache.

**Discussion/Conclusions:** This scoping review has demonstrated that buprenorphine may be able to be safely implemented into pain management strategies in the paediatric population. Along with other analgesic treatments, it also provides an alternative for patients who do not respond well to traditional opioid therapies. Moving forward, additional research will be required in order to further demonstrate the safety and efficacy of buprenorphine in pain management.

## Experiences Participating in Physical Activity and Exercise among Adults with Chronic Pain: An Interpretive Description Qualitative Study

Kyle Vader^a^^b^, Tom Doulas^b^, Rupa Patel^c^, and Jordan Miller^a^

^a^School of Rehabilitation Therapy, Queen’s University, Kingston, Ontario, Canada; ^b^Chronic Pain Clinic, Kingston Health Sciences Centre, Kingston, Ontario, Canada; ^c^Kingston Community Health Centre, Kingston, Ontario, Canada

**CONTACT** Kyle Vader kyle.vader@queensu.ca

© 2019 The Author(s). Published with license by Taylor & Francis Group, LLC.

This is an Open Access article distributed under the terms of the Creative Commons Attribution License (http://creativecommons.org/licenses/by/4.0/), which permits unrestricted use, distribution, and reproduction in any medium, provided the original work is properly cited.

**Introduction/Aim:** The purpose of this research was to explore the experiences of adults with chronic pain when they participate in physical activity and exercise and to understand perceived barriers and facilitators to participation.

**Methods:** We conducted an interpretive descriptive qualitative study. We recruited adults who self-identified as living with chronic pain from primary care teams and a hospital-based chronic pain clinic in Kingston, Ontario. An audit trail, reflexive dialogue, and thick description were used to maintain analytical rigor.

**Results:** 16 participants took part in an interview between October 2017 and January 2018. The majority of participants identified as female (11/16) and had a median age of 53 years. Three themes and subsequent sub-themes emerged: (1) unique features of physical activity and exercise in the context of chronic pain (physical activity through daily tasks, low intensity physical activity is best, flaring up after physical activity), (2) factors influencing participation in physical activity and exercise (access to fitness equipment/facilities, motivation to participate, uncertainty/fluctuating responses, social supports, perceived benefits, competing demands for time, knowledge, fears, physical abilities, environment, pain/fatigue, and confidence, and (3) potential outcomes of participating in physical activity and exercise (improved pain management, mental and emotional changes, social connections, functional gains, and other aspects of health)" should be deleted and replaced with the following sentence "Three major themes (and sub-themes) emerged: (1) the challenge of staying active (decreased activity levels due to pain, discomfort during physical activity, and uncertain and fluctuating abilities); (2) factors influencing participation (pain, fatigue, perceived risks, beliefs about physical activity, competing demands, social support, motivation, other health conditions, and access to supports for physical activity or exercise); and (3) perceived outcomes (pain management, functional improvements, social participation, mental health, and overall wellbeing).

**Discussion/Conclusions:** A combination of factors were perceived as influencing participation in physical activity and exercise in adults with chronic pain. Potential outcomes of physical activity and exercise focused on overall health and well-being among adults with chronic pain.

## Added Value of a Conditioning to Optimize the Neuromodulatory Effect of rTMS on Heat Thresholds: A Pilot Study

Léa Proulx-Bégin^a^, Alberto Herrero Babiloni^b^, Sabrina Bouferguene^c^, Gilles Lavigne^a^, Louis De Beaumont^d^, and Caroline Arbour^e^

^a^Psychology, Research Center, Hôpital du Sacré-Coeur de Montréal, Université de Montréal, Montréal, Québec, Canada; ^b^Research Center, Hôpital du Sacré-Coeur de Montréal Université de Montréal, Montréal, Québec, Canada; ^c^Faculty of Medecine, Research Center, Hôpital du Sacré-Coeur de Montréal, Université de Montréal, Biomedical Sciences, Montréal, Québec, Canada; ^d^Surgery, Faculty of Medecine, Research Center, Hôpital du Sacré-Coeur de Montréal, Université de Montréal, Montréal, Québec, Canada; ^e^Faculty of Nursing, Research Center, Hôpital du Sacré-Coeur de Montréal, Université de Montréal, Montréal, Québec, Canada

**CONTACT** Léa Proulx-Bégin lea.proulx-begin@umontreal.ca

© 2019 The Author(s). Published with license by Taylor & Francis Group, LLC.

This is an Open Access article distributed under the terms of the Creative Commons Attribution License (http://creativecommons.org/licenses/by/4.0/), which permits unrestricted use, distribution, and reproduction in any medium, provided the original work is properly cited.

**Introduction/Aim:** Repetitive transcranial magnetic stimulation (rTMS) can modulate heat pain thresholds (HPT) in health and disease. One obstacle of rTMS however is the number of sessions required to induce a significant effect on pain. We aimed to examine whether conditioning – a physiological response elicited through unconscious association of stimuli – can be used to optimize the effect of rTMS on HPT.

**Methods:** Twenty healthy subjects were randomly assigned to: 1) conditioning (n = 10;6F;23y±2) or 2) non-conditioning (n = 10;4F;23y±3) groups and took part in two laboratory visits (one with active rTMS the other with sham). For the active visit, two rTMS sessions of 20 min with a 20 min resting period in between were conducted over the left motor cortex (10Hz at 80% RMT). For sham visit, same parameters were used with a sham coil. HPT were evaluated over the right wrist area at baseline and after each rTMS/sham session. For the conditioning procedure, heat temperature eliciting moderate pain was determined at baseline. Then, in the conditioning group, the thermode temperature was decreased by 3°C during the resting period.

**Results:** Descriptive analyses showed a tendency towards an increase in HPT from baseline (*M = *42.95;*SD = *2.93) to 1^st^(*M = *43.88;*SD = *2.04) and 2^nd^(*M = *44.68;*SD = *2.44) rTMS sessions in the conditioning group, but not in the non-conditioning (*M = *43.23;*SD = *3.18; *M = *43.87;*SD = *3.51;*M = *43.35;*SD = *4.13) with active rTMS, which was less pronounced in HDT. Sham rTMS did not lead to HPT changes regardless of conditioning.

**Discussion/Conclusions:** Preliminary results suggest the value of conditioning to optimize the effects of rTMS on HPT, potentially opening new avenues in TMS research.

## What Predicts Perceived Medical Burden among Chronic Pain Patients with Medical Comorbidities?

Marie-Eve Martel^a^, Chrystelle El-Khoury^b^, and M Gabrielle Pagé^c^

^a^Department of Psychology, Université du Québec à Trois-Rivières, Trois-Rivières, Québec, Canada; ^b^Centre de recherche, Centre hospitalier de l’Université de Montréal, Montreal, Quebec, Canada; ^c^Centre de recherche, Centre hospitalier de l’Université de Montréal, & Department of Anesthesiology and Pain Medicine, Université de Montréal, Montreal, Quebec, Canada

**CONTACT** Marie-Eve Martel Marie-Eve.Martel1@uqtr.ca ^1^John Smith, University of Toronto, Psychology, Toronto, Ontario, Canada, @johnsmithuoft, @johnsmithuoft^2^Jane Doe, Queen’s University, Nursing, Kingston, Ontario, Canada, jdoe@queensu.ca, @jdoequeens

© 2019 The Author(s). Published with license by Taylor & Francis Group, LLC.

This is an Open Access article distributed under the terms of the Creative Commons Attribution License (http://creativecommons.org/licenses/by/4.0/), which permits unrestricted use, distribution, and reproduction in any medium, provided the original work is properly cited.

**Introduction/Aim**: Many chronic pain patients suffer from other chronic health conditions, which can lead to increased burden of symptom self-management. This increased perceived burden can negatively impact on patients’ beliefs, behaviors and treatment adherence. This study aims to identify predictors of perceived medical burden among chronic pain patients suffering from ≥1 additional medical condition.

**Methods**: A transversal observational study design was used. A total of 137 individuals with chronic pain (≥3 months) and ≥1 other medical condition were recruited from patient associations and social and conventional media and completed online questionnaires. A linear regression model using backward selection process was used to identify pain characteristics (duration, interference, average and worst pain intensities), patient (age, sex, gender, self-efficacy, patient engagement in healthcare), and medical (number and severity of comorbidities) characteristics associated with perceived medical burden.

**Results**: The final regression model included 3 variables: patient engagement in healthcare (*b *= −.25, *p* < .05) and pain interference (*b *= .48, *p* < .05), but not severity of comorbidities (*p *= 0.069) were significantly associated with perceived medical burden. Results showed the less individuals are engaged in their healthcare, the greater the burden perceived. In addition, the more pain interferes with daily activities, the more their medical conditions is perceived as burdensome.

**Discussion/Conclusions**: Results revealed that objective measures of severity of conditions, as evaluated by the Self-Administered Comorbidity Questionnaire and comorbidity count, are not associated with perceived medical burden. Targeting patient and pain characteristics might prove beneficial to reduce perceived medical burden and facilitate symptom self-management.

## Age and the Experience of Chronic Pain after Moderate-To-Severe Traumatic Brain Injury

Sabrina Bouferguene^a^, Naïcha-Éveline Germélus^b^, Pierre Rainville^c^, and Caroline Arbour^d^

^a^Department of Biomedical Sciences, Faculty of Medicine, Université de Montréal, Montréal, Québec, Canada; ^b^Hôpital du Sacré-Cœur de Montréal Research Center, Montréal, Québec, Canada; ^c^Department of Stomatology, Faculty of Dental Medicine, Université de Montréal, Montréal, Québec, Canada; ^d^Faculty of Nursing, Université de Montréal, Montréal, Québec, Canada

**CONTACT** Sabrina Bouferguene sabrina.bouferguene@umontreal.ca

© 2019 The Author(s). Published with license by Taylor & Francis Group, LLC.

This is an Open Access article distributed under the terms of the Creative Commons Attribution License (http://creativecommons.org/licenses/by/4.0/), which permits unrestricted use, distribution, and reproduction in any medium, provided the original work is properly cited.

**Introduction/Aim**: Chronic pain after moderate-to-severe traumatic brain injury (TBI) could be affected by age. Still, pain presentation in adult TBI has never been investigated across a large age range. We examined whether age contributes to chronic pain presentation after moderate-to-severe TBI.

**Methods**: N = 54 adults between 18–85 years (36 males, 46 ± 19 years) were recruited 23 ± 14 months after moderate-to-severe TBI. Chronic pain was assessed with the Brief Pain Inventory. Thermal and mechanical detection/pain thresholds were gathered using Quantitative Sensory Testing (QST). Data were transformed into z-scores to account for variation in gender and pain sites.

**Results**: Participants were categorized into three groups: n = 18 young (<35 years), n = 17 middle-aged (36–55 years), n = 19 older adults (≥55 years). Chronic pain was reported by n = 30 (56%) participants with an average intensity of 5 ± 2/10. Overall, young and middle-aged participants (≤55 years) with pain presented higher cold detection z-scores when compared to same age participants without pain (*t *= 2.28, *p *= 0.03). In contrast, older participants (≥55 years) suffering from chronic pain exhibited a lower heat pain z-score when compared to pain participants of the other age groups (*F *= 8.82, *p *= 0.001), but not when compared to same age participants without pain (*t *= 0.28, *p *= 0.79). Correcting for gender and time elapsed since TBI, age was not significantly related to chronic pain status (*X^2^ *= 0.347, *OR *= 1.01, *p *= 0.57).

**Discussion/Conclusions**: Our results support previous ones showing thermal hypoesthesia in young TBI with chronic pain. For the first time however, we show that being over 55 years could predispose to thermal hyperalgesia after TBI, suggesting possible age-related differences in neuroplasticity following adults.

## Parent Perspectives on the Benefits and Limitations of a Social Media Campaign to Disseminate Evidence-Based Information on Pediatric Cancer Pain

Perri R. Tutelman^a^, Lindsay A. Jibb 0000-0001-6995-2825^b^, Christine T. Chambers 0000-0002-7138-916X^c^, Jennifer N. Stinson 0000-0002-9969-8052^d^, Jennifer A. Parker 0000-0001-9900-4703^e^, Melanie Barwick 0000-0002-2478-604X^f^, Fiona Campbell^g^, Emily K. Drake^h^, Conrad V. Fernandez^i^, Karen Irwin^j^, Paul C. Nathan^k^, Holly O. Witteman^l^, #KidsCancerPain Parent/Patient Partners^m^

^a^Department of Psychology and Neuroscience, Dalhousie University & Centre for Pediatric Pain Research, IWK Health Centre, Halifax, Nova Scotia, Canada; ^b^School of Nursing, University of Ottawa, Ottawa, Ontario, Canada; ^c^Departments of Pediatrics and Psychology and Neuroscience, Dalhousie University & Centre for Pediatric Pain Research, IWK Health Centre, Halifax, Nova Scotia, Canada; ^d^, Faculty of Nursing, University of Toronto & Child Health Evaluative Sciences, Hospital for Sick Children, Toronto, Ontario, Canada; ^e^Centre for Pediatric Pain Research, IWK Health Centre, Halifax, Nova Scotia, Canada; ^f^Child Health Evaluative Sciences, Hospital for Sick Children & Department of Psychiatry, University of Toronto, Toronto, Ontario, Canada; ^g^Department of Anesthesia, University of Toronto & Department of Anesthesia and Pain Medicine, Hospital for Sick Children, Toronto, Ontario, Canada; ^h^Faculty of Health, Dalhousie University, Halifax, Nova Scotia, Canada; ^i^Department of Pediatrics, Dalhousie University & Division of Haematology/Oncology, IWK Health Centre, Halifax, Nova Scotia, Canada; ^j^Cancer Knowledge Network, Toronto, Ontario, Canada; ^k^Faculty of Medicine, University of Toronto & Division of Haematology/Oncology, Hospital for Sick Children, Toronto, Ontario, Canada; ^l^Department of Family and Emergency Medicine, Université Laval & Population Health and Optimal Health Practices Research Unit, Research Centre of the CHU de Québec-Université Laval, Québec City, Québec, Canada; ^m^Parent/Patient Partners, USA

**CONTACT** Perri R. Tutelman ptutelman@dal.ca

© 2019 The Author(s). Published with license by Taylor & Francis Group, LLC.

This is an Open Access article distributed under the terms of the Creative Commons Attribution License (http://creativecommons.org/licenses/by/4.0/), which permits unrestricted use, distribution, and reproduction in any medium, provided the original work is properly cited.

**Introduction/Aim**: #KidsCancerPain is a social media campaign that partnered with the Cancer Knowledge Network to disseminate evidence-based information on pediatric cancer pain directly to parents. Parent perspectives on the use of social media as a tool to translate research remain unclear. The purpose of this study was to explore parent-perceived benefits and limitations of the #KidsCancerPain campaign.

**Methods**: At the conclusion of the #KidsCancerPain campaign parents of children with cancer who viewed the campaign content were recruited to complete an online survey. Parents were asked to provide open-ended responses describing their perspectives on the benefits and limitations of the campaign. Data were analyzed using content analysis.

**Results**: A total of 120 parents (94% mothers) provided responses. Parent-perceived benefits of the #KidsCancerPain campaign clustered into three categories: (1) provision of practical pain management strategies (e.g., new techniques to manage their child’s pain), (2) reinforcement of pain management practices they were already using, and (3) provision of confidence and support to manage their child’s pain, fear and distress. Parent-perceived limitations of the campaign were its applicability (e.g., child not experiencing pain or completed treatment) and visibility (e.g., some parents only recently became aware of the campaign, and some reported limited social media use).

**Discussion/Conclusions**: Parents described both benefits and limitations of the #KidsCancerPain campaign related to managing their child’s pain. Strategies to optimize the reach of social media content should be made in consultation with parents prior to future campaigns. These data will help guide broader parent-directed dissemination efforts using social media.

## Acupuncture for the Management of Chronic Diabetic Peripheral Neuropathy: A Systematic Review and Meta-Analysis of Randomized Trials

Ngai Chow 0000-0002-1800-0362^a^, Mahmood AminiLari^a^, Rachel Couban^b^, Li Wang 0000-0003-1585-8846^b^, Jason W. Busse 0000-0002-0178-8712^b^

^a^Department of Health Research Methods, Evidence, and Impact, McMaster University, Hamilton, Ontario, Canada; ^b^Department of Anesthesia, McMaster University, Hamilton, ON, Canada

**CONTACT** Ngai Chow chown7@mcmaster.ca In the present study we just report the results of 4 eligible studies published in English. In the updated study, we have also included 52 articles published in Chinese with the assistance of our Chines colleagues. We will report the results of our new analyses in the near future.

© 2019 The Author(s). Published with license by Taylor & Francis Group, LLC.

This is an Open Access article distributed under the terms of the Creative Commons Attribution License (http://creativecommons.org/licenses/by/4.0/), which permits unrestricted use, distribution, and reproduction in any medium, provided the original work is properly cited.

**Introduction/Aim**: Peripheral neuropathy is a common cause of chronic pain among patients with diabetes, and acupuncture has been suggested as a therapeutic option. To determine the effectiveness of acupuncture for chronic diabetic peripheral neuropathy (DPN).

**Methods**: We searched MEDLINE, EMBASE, CENTRAL, AMED, CINAHL, PsychINFO, trial registries, and reference lists of relevant articles up to February 2017. Pairs of reviewers, independently and in duplicate, screened articles for inclusion, assessed risk of bias and extracted data. We conducted meta-analyses when possible and used the GRADE approach to assess the quality of evidence.

**Results**: Among 4443 potentially eligible studies 4 with 244 patients proved eligible to be included for review. Overall all four studies were at high risk of bias. Compared to sham acupuncture, we found very low quality evidence that acupuncture reduces pain (weighted mean difference [WMD] −1.95cm on a 10cm visual analogue scale, 95% CI −3.27 to −0.64; minimally important difference [MID] 1cm; risk difference (RD) for achieving the MID 52%; 95% CI 19% to 63%) and improves physical functioning (WMD 3.68 points on the short form-36 [SF-36] physical component summary score; 95% CI 1.66 to 5.70; MID is 5-points; RD for achieving the MID 8%, 95% CI 3% to 18%), but does not affect emotional functioning (WMD 1.26 points on the SF-36 mental component summary score, 95% CI: −1.13 to 3.66).

**Conclusions**: Very low-quality evidence suggests that acupuncture reduces pain and improves physical functioning in patients with chronic DPN but does not affect emotional functioning.

## Online Parent-Targeted Resources for Early Childhood Vaccination: A Cross-Canada Environmental Scan

Shokoufeh Modanloo^a^, Juliana Choueiry^a^, Sandra Dunn^b^, Dawn Stacey^c^, Denise Harrison 0000-0001-7549-7742^a^

^a^Children’s Hospital of Eastern Ontario (CHEO) Research Institute, University of Ottawa, Ottawa, Ontario, Canada; ^b^Better Outcomes Registry & Network (BORN), Ottawa, Ontario, Canada; ^c^Ottawa Hospital Research Institute, Ottawa, Ontario, Canada

**CONTACT** Shokoufeh Modanloo smoda044@uottawa.ca Children’s Hospital of Eastern Ontario (CHEO) Research Institute, University of Ottawa, Ottawa, Ontario, Canada

© 2019 The Author(s). Published with license by Taylor & Francis Group, LLC.

This is an Open Access article distributed under the terms of the Creative Commons Attribution License (http://creativecommons.org/licenses/by/4.0/), which permits unrestricted use, distribution, and reproduction in any medium, provided the original work is properly cited.

**Introduction/Aim**: Early childhood vaccination is painful and distressing for infants and parents. Clinical practice guidelines recommend effective evidence-based pain management strategies during vaccination (breastfeeding, upright holding, sucrose). This study aims to identify and quality appraise publicly accessible online information regarding parent-targeted resources for pain management during vaccinations of infants.

**Methods**: An environmental scan for electronic resources containing parent-targeted information on infant vaccination were included, using internet sources in English i) Google search and ii) Social Media networks. Characteristics of the resources were collected. The quality of resources was evaluated by Center for Disease Control (CDC) Clear Communication Index (CCI) (score range 0–100%, higher than 90% are acceptable). Descriptive statistics were used to analyze the data.

**Results**: 65 eligible resources were identified. The mean resources’ CCI score was 60% (± 0.19) with most resources scoring as low to moderate quality (score 33% to 87%). Only 5% of resources were considered to be acceptable quality, almost all requiring revisions. Pain management information was presented in 30 (46%) resources; 24 (37%) included breastfeeding, 27 (42%) holding, and 22 (34%) sweet solutions. No statement regarding pain management strategies were found in over half (54%) of the resources. In resources describing pain management, distraction and holding were the most frequently suggested strategies.

**Discussion/Conclusions**: Most online parent-targeted vaccination resources are of low quality and do not include evidence-based pain management strategies. Thus, there is a need for researchers, policy makers, and educational institutes to develop improved, publicly accessible information about infant vaccination and pain management strategies.

## Characteristics of Patients with Inflammatory Bowel Disease Using Cannabis (IBD) for Pain and Mood

Lillian Du^a^, A. Hillary Steinhart^b^, Shlomit Boguslavsky^c^, Erin Murphy^d^, Kenneth Croitoru^b^, Zane Gallinger^e^, Vivian Huang^b^, Mark Silverberg^f^, Adam V. Weizman^b^, Geoffrey C. Nguyen^f^, Amol Deshpande^g^

^a^Gastroenterology, University of Toronto, Toronto, Ontario, Canada; ^b^Medicine, Mount Sinai Hospital, University of Toronto, Toronto, Ontario, Canada; ^c^Gastroenterology, Mount Sinai Hospital, Toronto, Ontario, Canada; ^d^Nursing, Trent University, Toronto, Ontario, Canada; ^e^Gastroenterology, University of Toronto, Toronto, Ontario, Canada; ^f^Mount Sinai Inflammatory Bowel Disease Centre, University of Toronto, Toronto, Ontario, Canada; ^g^Department of Family and Community Medicine, University of Toronto, Toronto, ON, Canada

**CONTACT** Amol Deshpande amol.deshpande@uhn.ca Department of Family and Community Medicine, University of Toronto, Toronto, ON, Canada

© 2019 The Author(s). Published with license by Taylor & Francis Group, LLC.

This is an Open Access article distributed under the terms of the Creative Commons Attribution License (http://creativecommons.org/licenses/by/4.0/), which permits unrestricted use, distribution, and reproduction in any medium, provided the original work is properly cited.

**Introduction/Aim**: An estimated 35% of all Inflammatory Bowel Disease (IBD) patients in remission continue to experience chronic abdominal pain. Despite the negative impact on quality of life, minimal research effort has been devoted to addressing this issue. IBD patients often seek alternative therapies to manage persistent pain including the use of cannabis, without evidence to support its use. This study describes the characteristics of pain, mood and cannabis use in IBD patients.

**Methods**: This is a cross-sectional survey evaluating a cohort of ambulatory patients, age ≥16 years with a diagnosis of IBD for over one month.

**Results**: A total of 206 surveys were collected with initial results reported on 123. Constant pain was present in 26.8%% of IBD patients with 34.9% reporting at least moderate levels of pain (NRS>4). Opioid use for more than 30 days was reported by 13% of all patients with 31.3% and 34.7% actively using cannabis in Crohn’s Disease (CD) and Ulcerative Colitis (UC), respectively.

Of active cannabis users, 67% used less than 2.5 g/d of dried cannabis. History of recreational use was greater in active cannabis users versus non-users but was only significant in UC. Average scores on the cannabis use disorder-short form were >4 in active cannabis users. The PHQ-9 and GAD-7 documented moderate depression and anxiety in all groups with significant levels of depression (18.0 vs. 14.4, p < .05) between active cannabis users versus non-users in CD. Only 14.9% of active cannabis users endorsed subjective improvement in pain or mood.

**Discussion/Conclusions**: Constant pain of at least moderate intensity and associated depression and anxiety is common in IBD. While cannabis use is prevalent, few report subjective improvement.

## Neuro-Immune Control of Post-Operative Pain via CCR4

Jaqueline Raymondi Silva^a^, Courtney A. Bannerman^a^, Julia P. Segal^b^, Francisco Gomes^c^, Thiago Mattar Cunha^c^, Ian Gilron^a^, Nader Ghasemlou^a^

^a^Department of Biomedical & Molecular Sciences, Queen’s University, Kingston, Ontario, Canada; ^b^Department of Anesthesiology and Biomedical & Molecular Sciences, Queen’s University, Kingston, Ontario, Canada; ^c^Department of Pharmacology, University of Sao Paulo, Sao Paulo, Brazil

**CONTACT** Jaqueline Silva jrs5@queensu.ca Department of Biomedical & Molecular Sciences, Queen’s University, Kingston, ON, Canada

© 2019 The Author(s). Published with license by Taylor & Francis Group, LLC.

This is an Open Access article distributed under the terms of the Creative Commons Attribution License (http://creativecommons.org/licenses/by/4.0/), which permits unrestricted use, distribution, and reproduction in any medium, provided the original work is properly cited.

Inflammatory pain is a result of complex and dynamic interactions between the immune and the nervous systems, and includes the orchestrated recruitment and activation of tissue-resident and circulating immune cells. Our previous studies have identified a central role for Ly6C-lo myeloid cells in the pathogenesis of inflammatory pain. We now show that CCL17 and CCL22, chemokines expressed preferentially by these cells, and their cognate receptor CCR4 are key mediators of this response. CCL17 and CCL22 are both upregulated significantly early after tissue injury and elicit a robust acute pain response when administered subcutaneously. Pharmacological blockade of CCR4 using a specific antagonist abrogates this effect. Acute post-surgical pain is also significantly reduced in both transgenic mice lacking CCR4 and wildtype animals treated with a CCR4 receptor antagonist. Together, these results suggest an essential role for the CCL17/CCL22:CCR4 axis in the genesis of inflammatory pain and opens new therapeutic avenues for its control.

## Hypervigilance to Pain in the Laboratory Mouse

Kevin C. Lister^a^, Sioui Maldonado Bouchard^a^, Stephania Donayre Pimentel^a^, Mariam Majeed^a^, Amanda Cdec Williams^b^, Andrea Aternali^a^, Jeffrey S. Mogil^a^

^a^Psychology Department, McGill University, Montreal, Quebec, Canada; ^b^Department of Clinical, Educational & Health Psychology, University College London, London, UK

**CONTACT** Kevin Lister kevin.lister@mail.mcgill.ca

© 2019 The Author(s). Published with license by Taylor & Francis Group, LLC.

This is an Open Access article distributed under the terms of the Creative Commons Attribution License (http://creativecommons.org/licenses/by/4.0/), which permits unrestricted use, distribution, and reproduction in any medium, provided the original work is properly cited.

**Introduction/Aim**: We designed an experiment to evaluate the potential adaptive significance of chronic pain in mammals, by measuring vigilance behaviour to predator odour. We hypothesized that mice in pain will become hypervigilant to predation threat.

**Methods**: To test threat avoidance behaviour, we developed an octagonal apparatus in which odour can be infused into a single arm. Mice were trained to seek food reward (a Fruit Loop) via the short route around the maze. Once trained, fox urine was infused such that taking the short route would entail encountering the fox odour. The number of short versus long routes chosen was recorded in five trials, in mice in the following conditions: 1) control, 2) 0.6% acetic acid, and 3) 0.6% acetic acid plus 20 mg/kg carprofen.

**Results**: Mice in all groups showed vigilance (I.e., avoidance of the short route) when fox urine was present compared to when it was not. Pain increased vigilance behaviour, and analgesia reversed this hypervigilance.

**Discussion/Conclusions**: We conclude that pain produces hypervigilance against predation. If chronic pain produces the same effect, as has been demonstrated in squid, this may represent the adaptive significance of chronic pain hypersensitivity.

## Long-Term Physical Exercise Training Program Successfully Reduces Pain Intensity and Benefits Psychological Factors in Individuals Experiencing Chronic Low Back Pain

Anna Bendas 0000-0002-2903-4015^a^^b^^c^, Kelly Cool 0000-0001-5078-6599^a^^b^^c^, Scott J. Thompson 0000-0003-3306-4513^a^^b^^c^, Florian Bobeuf 0000-0002-4798-1091^a^^b^^c^, Pierre Rainville^a^^b^^c^, Louis Bherer^a^^b^^c^, Julien Cohen-Adad 0000-0003-3662-9532^a^^b^^c^, Mathieu Roy 0000-0002-3335-445X^a^^b^^c^

^a^Department of Psychology, McGill University, Montreal, QC, Canada; ^b^Centre de recherche de l’Institut universitaire de gériatrie de Montréal, Montreal, QC, Canada; ^c^Centre de recherche de l’Institut universitaire de gériatrie de Montréal & Institut de Cardiologie de Montréal, Montreal, QC, Canada

**CONTACT** Anna Bendas anna.bendas@mail.mcgill.ca

© 2019 The Author(s). Published with license by Taylor & Francis Group, LLC.

This is an Open Access article distributed under the terms of the Creative Commons Attribution License (http://creativecommons.org/licenses/by/4.0/), which permits unrestricted use, distribution, and reproduction in any medium, provided the original work is properly cited.

**Introduction/Aim**: The Global Burden of Disease study (2015) reported that chronic low back pain (CLBP) is the most prevalent and disabling condition amongst numerous chronic illnesses. According to the guidelines provided by Airaksinen and colleagues (2006), a supervised physical exercise intervention is an important first-step treatment of nonspecific chronic low back pain. Here, we investigated the impact of a 14-week long personalized physical exercise training program on pain intensity, perceived disability and several psychological factors in people suffering from CLBP.

**Methods**: Twenty-three participants (female – 16, male – 7, age range 22–72 years old) suffering from chronic low back pain were recruited to complete 14 weeks of cardiovascular and muscle strengthening program. At the beginning and at the end of this intervention, we assessed their pain intensity following the NIH (2017) guidelines, and asked participants to complete several psychosocial questionnaires (Beck Depression Inventory, Pain Catastrophizing Scale, and Oswestry Disability Index).

**Results**: Following a long-term physical exercise intervention, perceived pain intensity decreased significantly (p = 0.001). Pain catastrophizing score (p = 0.004), Oswestry disability index (p = 0.005) and Beck depression index (p = 0.022) showed a significant decrease.

**Discussion/Conclusions**: Our findings suggest that a long-term physical exercise training regimen substantially contributes to the reduction of pain intensity levels and improves psychological factors in patients with chronic low back pain. In order to further support our hypothesis, we are looking forward to including the results from a wait-list control group in our future analysis.

## Stimulus Parameters Driving Dorsal Horn Wide Dynamic Range Neuron Responses to Mechanical Stimuli: A Systematic Review

Maham Zain^a^, Robert P. Bonin^a^

Leslie Dan Faculty of Pharmacy, University of Toronto, Toronto, Ontario, Canada

**CONTACT** Maham Zain maham.zain@mail.utoronto.ca

© 2019 The Author(s). Published with license by Taylor & Francis Group, LLC.

This is an Open Access article distributed under the terms of the Creative Commons Attribution License (http://creativecommons.org/licenses/by/4.0/), which permits unrestricted use, distribution, and reproduction in any medium, provided the original work is properly cited.

**Introduction/Aim**: Wide dynamic range (WDR) neurons of the spinal dorsal horn respond to a wide range of innocuous and noxious mechanical stimulation and encode the intensity of mechanical stimuli as changes in firing rate. However, there are inconsistent findings regarding whether WDR neuron activity is altered in pathological pain states. Additionally, there is still ambiguity regarding which stimulus parameters drive WDR firing. The aim of the current investigation was to review the available literature in order to ascertain whether WDR neuronal firing in rats is better predicted by pressure or force of a mechanical stimulus (von Frey filaments) and to assess possible mediating factors.

**Methods**: A systematic search was performed on PubMed. All papers were screened on the basis of a certain inclusion criteria and selected publications had a number of variables extracted from text, tables and figures. Forces of the von Frey filaments were converted to pressure by constructing a conversion equation based on manufacturer reported force and diameters. Statistical analyses were performed on the pooled data.

**Results**: We observed that WDR firing rate was better predicted by the calculated pressure of von Frey stimulation rather than applied filament force, as reported in all studies. The pressure-evoked firing rate of WDR neurons was not altered by any experimental pain model except for arthritis and inflammation models, where mechanical stimuli evoked a higher firing rate than controls. Conversely, there was a consistent increase the spontaneous firing rate of WDR neurons in neuropathic pain, arthritis and inflammation, and chemoneuropathy pain models.

**Discussion/Conclusions**: Evidence suggests that pressure of a mechanical stimulus, compared to force, is a better predictor of WDR neuronal firing. Additionally, these data indicate that changes in WDR encoding of applied pressure are unlikely to significantly contribute to pathological sensory processing but indicate a possible role for these neurons in spontaneous pain.

## Attachment Insecurity and Pain in Romantic Dyads: An Exploration of Actor and Partner Effects

Dyana Castillo^a^, Constance Heidt^b^, Lachlan McWilliams^a^

^a^Psychology, University of Saskatchewan, Saskatoon, SK, Canada; ^b^Department of Clinical Health Psychology, Saskatchewan Health Authority, Saskatoon, SK, Canada

**CONTACT** Dyana Castillo dyana.castillo@usask.ca

© 2019 Crown copyright. Published with license by Taylor & Francis Group, LLC.

This is an Open Access article distributed under the terms of the Creative Commons Attribution License (http://creativecommons.org/licenses/by/4.0/), which permits unrestricted use, distribution, and reproduction in any medium, provided the original work is properly cited.

**Introduction**: Attachment insecurity (i.e., anxiety and avoidance in relationships) is associated with a wide range of negative outcomes, including the experience of pain and pain-related disability. To date, research on this topic has utilized samples of individuals and has examined “actor effects” (i.e., relationships between individuals’ own attachment characteristics and their own pain variables). The current study investigates attachment and pain within dyads of romantic partners in order to investigate “partner effects” (i.e., relationships between individuals’ attachment characteristics and the functioning of their partners).

**Method**: An online survey was administered to both members of 173 heterosexual romantic couples. They provided self-reports of attachment and pain experienced over the past four weeks. The Actor-Partner Interdependence Model, a statistical method that accounts for interdependence within dyads, was used.

**Results**: Males’ attachment anxiety was associated with higher levels of self- and partner-reported pain (i.e., both actor and partner effects). Females’ attachment anxiety was associated with higher levels of self-reported pain, but there was not a significant partner effect. Attachment avoidance was generally unrelated to pain reports. However, there was a significant partner effect for females’ avoidance (i.e., attachment avoidance reported by women was positively associated with the pain reports of their male partners.

**Conclusion**: Results highlight the importance of considering dyadic relationships when investigating the potential impact of attachment insecurity on the experience of pain.

## Investigating the Relationship between Diet-Induced Obesity, Intervertebral Disc Degeneration and Back Pain

Geoffrey John Kerr^a^, Ian White^a^, Magali Millecamps^b^, Laura S. Stone^b^, Cheryle A. Séguin^a^

^a^Department of Physiology and Pharmacology, Bone and Joint Institute, University of Western Ontario, London, Ontario, Canada; ^b^Alan Edwards Centre for Research on Pain, Faculty of Dentistry, McGill University, Montreal, QC, Canada

**CONTACT** Geoffrey John Kerr gkerr7@uwo.ca

© 2019 The Author(s). Published with license by Taylor & Francis Group, LLC.

This is an Open Access article distributed under the terms of the Creative Commons Attribution License (http://creativecommons.org/licenses/by/4.0/), which permits unrestricted use, distribution, and reproduction in any medium, provided the original work is properly cited.

**Introduction:** Despite being the most common cause of disability worldwide, the etiology of back pain remains unknown, but is often associated with intervertebral disc (IVD) degeneration. Epidemiological studies have highlighted obesity as a major contributor to both IVD degeneration and back pain, yet the underlying biological mechanisms remain elusive. Our study aimed to investigate if diet-induced obesity accelerates age-induced IVD degeneration, and/or back pain.

**Methods:** Ten-week-old, male C57BL/6 mice were fed a western diet (high fat/high sugar), a high fat diet, or standard chow (control) for 12, 24 or 40 weeks. Prior to endpoint, behavioral tests were performed to assess indicators of pain. Following sacrifice, joint tissues were examined through histological, radiological and molecular analysis.

**Results:** The consumption of the high-fat and western diets significantly increased body weight over time compared to control. Behavioral analysis showed reduced thresholds to mechanical stimulus (Von Frey) in both experimental diet groups, suggesting mechanical hypersensitivity of the hind limbs. Grip force strength during axial stretch was also reduced in both experimental diet groups, suggestive of axial discomfort. The velocity and distance traveled in the open field assay were decreased in both experimental diet groups, suggesting motor impairment. Preliminary histological analysis of the lumbar spines revealed accelerated IVD degeneration in both experimental diet groups compared to control.

**Conclusions:** Our findings suggest that diet-induced obesity accelerates age-related IVD degeneration and back pain associated behaviors in the mouse. Ongoing analysis will examine neuroplastic changes to the sensory nervous system and circulating systemic factors to identify pathways involved.

## Altered Functional Network Configuration in Persons with Neuropathic Pain: A Resting-State fMRI Analysis Using Graph Theory

Scott Holmes^a^, Nadia Barakat^a^, Natalia I. Lopez^a^, Laura Simons^b^, Alyssa Lebel^c^, David Borsook^a^

^a^Anesthesiology, Perioperative & Pain Medicine, Boston Children’s Hospital, Boston, MA, USA; ^b^Anesthesiology, Perioperative & Pain Medicine, Stanford University School of Medicine, Palo Alto, CA, USA; ^c^Anesthesiology, Perioperative and Pain Medicine, Boston Children’s Hospital, Boston, MA, USA

**CONTACT** Scott Holmes scott.holmes@childrens.harvard.edu

© 2019 The Author(s). Published with license by Taylor & Francis Group, LLC.

This is an Open Access article distributed under the terms of the Creative Commons Attribution License (http://creativecommons.org/licenses/by/4.0/), which permits unrestricted use, distribution, and reproduction in any medium, provided the original work is properly cited.

**Introduction/Aim**: The neuro-functional basis of pain chronification is not yet understood. Altered brain activity associated with pain symptoms has been identified in neuropathic pain patients. The extent to which brain networks may be altered in persons with acute neuropathic pain remains unclear.

**Methods**: Participants included patients with ankle sprain (n = 24, Age = 16.1years, SD = 4.1) with clinically confirmed neuropathic pain and a group of age-matched healthy controls (n = 12, Age = 16.1, SD = 2.95). Participants completed questionnaires about pain symptoms and underwent a resting-state fMRI (rs-fMRI) scan. Graph theory was applied on rs-fMRI data using the CONN toolbox implemented in Matlab. Individual brain regions were evaluated based on global and local efficiency, betweenness centrality, cost, average path length, clustering coefficient and nodal degree.

**Results**: Patients reported higher scores than healthy controls on the Pediatric Pain Screening Tool (p < 0.001) and Functional Disability Inventory (p < 0.001) suggesting a significant impact of pain on physical and psychological functioning. No group differences were observed for global and local efficiency, cost, average path length, clustering coefficient or nodal degree (p-FDR>0.05). The ankle sprain cohort exhibited higher betweenness centrality in the posterior aspect of the left parahippocampal gyrus compared to healthy controls (t(30) = 4.20,p-FDR = 0.029).

**Discussion/Conclusions**: Findings suggest that functional brain network properties may be affected in persons with acute neuropathic pain. The increased betweenness centrality in the parahippocampal cortex in persons with neuropathic pain may pertain to changes in memory encoding due to the presence of pain. Perturbed network configuration from acute neuropathic pain relating to memory formation may underlie a component of pain chronification.

## The Effects of Exercise Therapy on Executive Functioning in Patients with Chronic Low Back Pain: Preliminary Results

Erika Gentile^a^, Florian Bobeuf 0000-0002-4798-1091^b^, Anna Bendas 0000-0002-2903-4015^a^, Kelly Cool 0000-0001-5078-6599^a^, Maxime Lussier^b^, Louis Bherer^c^, Mathieu Roy 0000-0002-3335-445X^a^

^a^Psychology, McGill University, Montreal, Quebec, Canada; ^b^Centre de recherche de l’Institut universitaire de gériatrie de Montreal, Montreal, Quebec, Canada; ^c^Centre de recherche de l’Institut universitaire de gériatrie de Montréal & l’Institut de Cardiologie de Montréal, Montreal, Quebec, Canada

**CONTACT** Erika Gentile erika.gentile@mail.mcgill.ca

© 2019 The Author(s). Published with license by Taylor & Francis Group, LLC.

This is an Open Access article distributed under the terms of the Creative Commons Attribution License (http://creativecommons.org/licenses/by/4.0/), which permits unrestricted use, distribution, and reproduction in any medium, provided the original work is properly cited.

Chronic low back pain (CLBP) is the most prevalent and problematic of all chronic pain syndromes. In addition to enduring persistent pain, they also experience a reduction in cognitive abilities. This is because CLBP patients have more difficulty summoning the cognitive resources necessary to meet professional and personal responsibilities, often providing them with a major obstacle in every day functioning. While almost all cognitive domains appear to be compromised, executive functioning seem to be one of the most affected abilities. Despite this, such cognitive deficits are rarely the focus of therapeutic interventions since they are widely viewed as secondary to chronic pain. Thus, an effective treatment for them is still unknown. Fortunately, physical exercise has been shown to be beneficial throughout the lifespan by optimizing overall physical and cognitive health and is one of few interventions that reliably improves pain management in CLBP. The present study aimed to test the impact of a physical exercise intervention on executive functioning in patients with CLBP. Twenty-three patients with non-specific CLBP completed a 14-week aerobic and muscular exercise program. Before and after the program, they performed a dual task that assessed their ability to identify different stimuli while simultaneously using their left and right hand. Our findings showed that after the exercise program, the patients’ ability to perceive multiple stimuli and coordinate the execution of two motor responses was improved. These results suggest that higher-order cognitive behavior, such as executive functioning, can be ameliorated with the help of exercise in these patients.

**Introduction/Aim**: Besides having to live in constant pain and limited mobility, patients with chronic low back pain (CLBP) have a constellation of symptoms that further contribute to their disability, such as cognitive impairment. While almost all cognitive domains appear to be compromised, executive functions seem to be one of the most affected abilities. Currently, physical exercise is one of the few interventions that reliably improves the management of pain in CLBP. However, since the cognitive deficits seen in CLBP patients are widely viewed as a secondary to pain, the impact of exercise therapy on such deficits have yet to be investigated. The present study sought to examine the effects of exercise training on dual task performance – a measure of executive functioning – in CLBP patients.

**Methods**: Twenty-three patients (15 female, 8 male, age range 22–65) with non-specific CLBP participated in a 14-week aerobic and muscular exercise program. Before and after the program, patients completed a dual task that required them to perform an identification task while simultaneously using their left and right hand. Reaction times were recorded.

**Results**: Preliminary results revealed a significant three-way interaction between time, task, and side (left and right hand), F(1,22) = 4.126, p = .054. After exercise training, patients showed a near-significant reduction in reaction time difference between the two hands suggesting that patients’ ability to perceive multiple stimuli and coordinate the execution of two motor responses improved, F(1,22) = 3.775, p = .065.

**Discussion/Conclusions**: Our findings indicate that patients’ task coordination ability required to execute multiple tasks is improved with exercise. Further, it provided insight into a feasible intervention that has the potential to enhance the cognitive health and subsequently, the functionality and quality of life of chronic pain sufferers.

## Microglia-Mediated Removal of Perineuronal Nets Contributes to the Development of Hypersensitivity in Neuropathic Pain Models

Shannon Tansley^a^, Jordyn Heal^b^, Annie Castonguay^c^, Noosha Yousefpour^d^, Antoine Godin^c^, Alfredo Ribeiro-da-Silva^e^, Yves De Koninck^f^, Luda Diatchenko^g^, Jeffrey Mogil^h^, Arkady Khoutorsky^i^

^a^Integrated Program in Neuroscience, McGill University, Montreal, Quebec, Canada; ^b^Arts & Science, McGill University, Montreal, Quebec, Canada; ^c^CERVO Brain Research Centre, Université Laval, Québec, QC, Canada; ^d^Department of Physiology, Alan Edwards Centre for Research on Pain, McGill University, Montreal, QC, Canada; ^e^Department of Physiology, Alan Edwards Centre for Research on Pain, McGill University, Montreal, QC; ^f^Université Laval, Department of Psychiatry and Neuroscience, CERVO Brain Research Centre, Québec, QC, Canada; ^g^Alan Edwards Centre for Research on Pain, McGill University, Montreal, QC, Canada; ^h^Department of Psychology, Alan Edwards Centre for Research on Pain, McGill University, Montreal, QC, Canada; ^i^Anesthesia, McGill University, Montreal, Quebec, Canada

© 2019 The Author(s). Published with license by Taylor & Francis Group, LLC.

This is an Open Access article distributed under the terms of the Creative Commons Attribution License (http://creativecommons.org/licenses/by/4.0/), which permits unrestricted use, distribution, and reproduction in any medium, provided the original work is properly cited.

**Introduction/Aim**: In the nervous system, neurons and glial cells are embedded within an extracellular matrix (ECM), whose components not only provide structural support, but also regulate synapse formation and function, and modulate neuronal excitability. The ECM restricts synaptic and structural plasticity, and enzymatic digestion of ECM affects acquisition of memories, and promotes cognitive flexibility and extinction. In this study, we investigated how remodeling of ECM components, specifically perineuronal nets (PNNs) contributes to the sensitization of spinal nociceptive circuits after injury.

**Methods**: Immunohistochemical analysis was used to assess varying components of PNNs in the spinal cord, as well as microglial engulfment. To label projection neurons, AAV2/9-CMV-CRE-eGFP virus was injected into parabrachial nuclei of TdTomato reporter mice. Behavioural studies were performed after intraspinal delivery of AAV2/9-CMV-chABC on 6–8-week-old C57BL/6 mice. Both sexes were used in all studies.

**Results**: In the dorsal horn of the spinal cord, perineuronal nets are preferentially found around projection neurons in the lamina I, and the lateral spinal nucleus. After spared nerve injury (SNI), microglia mediate removal of components of PNNs via engulfment processes. Viral delivery of an enzyme that degrades PNNs (AAV 2/9-CMV-chABC) promotes hypersensitivity in mice in both SNI and CCI (chronic constriction injury) models.

**Discussion/Conclusions**: Nerve injury causes robust microglia-mediated remodeling of the ECM in the dorsal horn of the spinal cord. Removal of perineuronal nets promotes hypersensitivity in mice, suggesting that PNNs are involved in regulation of spinal nociceptive circuits. Ongoing studies are aiming to assess the role of PNNs in chloride regulation after nerve injury.

## Characterizing Perineuronal Nets in the Dorsal Horn of the Spinal Cord

Shannon Tansley^a^, Jordyn Heal^b^, Arkady Khoutorsky^c^

^a^Integrated Program in Neuroscience, McGill University, Montreal, Quebec, Canada; ^b^Arts & Science, McGill University, Montreal, Quebec, Canada; ^c^McGill University, Anesthesia, Montreal, Quebec, Canada

**CONTACT** Shannon Tansley shannon.tansley@mcgill.ca

© 2019 The Author(s). Published with license by Taylor & Francis Group, LLC.

This is an Open Access article distributed under the terms of the Creative Commons Attribution License (http://creativecommons.org/licenses/by/4.0/), which permits unrestricted use, distribution, and reproduction in any medium, provided the original work is properly cited.

**Introduction/Aim**: Perineuronal Nets (PNNs) are net-like structures that are found around the cell soma and proximal dendrites of neurons. PNNs play a role in regulating neuronal plasticity in the cortex and hippocampus. In the spinal cord, previous studies have characterized PNNs around motor neurons in the ventral horn, however, they have not been described in the dorsal horn. In this study, we aim to characterize PNNs in the spinal cord dorsal horn and investigate developmental changes in PNNs formation. We also aim to establish the subtypes of neurons in the dorsal horn surrounded by PNNs.

**Methods**: C57BL/6 mice of different ages (P2, P42-56 and 2 years) were transcardially perfused, and their spinal cord extracted. Spinal cord sections were stained for WFA (L1516, Sigma), Aggrecan (AB1031, Millipore) and NeuN (ABN90, Millipore). Images were collected using wide-field microscope with Apotome and quantified using ZEN and ImageJ.

**Results**: In the spinal cord dorsal horn, WFA staining (chondroitin sulfate sugar chains) is present exclusively in lamina I. A small subset of neurons are labeled with Aggrecan and WFA in lamina I of the dorsal horn and lateral spinal nucleus. PNNs are not present in the spinal cord of P2 mice but were found in adult animals.

**Discussion/Conclusions**: Our study has shown that PNNs are present in spinal cord lamina 1 and the lateral spinal nucleus. These areas are involved in nociceptive processing. We propose that PNNs in the spinal cord dorsal horn are involved in regulation of spinal nociceptive circuits.

## Sustainability of the CPOT Use Three and Five Years after Its Implementation in an Adult Intensive Care Unit

Melissa Richard-Lalonde^a^, Raphaël Morisseau-Guillot^b^, Christine Echegaray-Benites^c^, Céline Gélinas^d^

^a^Ingram School of Nursing, McGill University, Montréal, Québec, Canada; ^b^Psychiatry, Université de Montréal, Montreal, Quebec, Canada; ^c^McGill University Health Centre, Emergency Department, Montreal, Quebec, Canada; ^d^Ingram School of Nursing, McGill University, and Centre for Nursing Research, CIUSSS Centre-Ouest-Ile-Montréal – Jewish General Hospital, Montréal, Québec, Canada

**CONTACT** Céline Gélinas

© 2019 The Author(s). Published with license by Taylor & Francis Group, LLC.

This is an Open Access article distributed under the terms of the Creative Commons Attribution License (http://creativecommons.org/licenses/by/4.0/), which permits unrestricted use, distribution, and reproduction in any medium, provided the original work is properly cited.

**Introduction/Aim**:Practice guidelines recommend the use of validated behavioral scales such as the Critical-Care Pain Observation Tool (CPOT) for pain assessment in the nonverbal critically ill. While evidence has shown the sustainable use of the CPOT up to 12 months post-implementation, long-term use of the tool had never been studied. This study aimed to evaluate the sustainability of the CPOT use at three- and five-years post-implementation.

**Methods**: Medical charts documenting a 24-hour period within the first 48 hours of the ICU stay were reviewed. Included patients were 18 years or older with a Glasgow Coma Scale of 4–12, without spinal cord injury or neuromuscular blockade. Data collected included documentation of pain assessments with the CPOT, administration of opioids, and pain reassessments.

**Results**: A total of 60 files were included (30 for each time point). The mean age was 63.35 (20–89) years, with 38.3% being male (no difference between samples). Pain assessments done using CPOT were reported on average 7–8 times per 24 hours, ranging from 0–20 assessments (median of 1.00 to 3.00 per shift). There was an average of 1.0–2.5 bolus of opioids administered over the same period. Following an opioid bolus, pain was reassessed <60% of the time. Based on pain reassessments, 59.1% (three years) and 29.4% (five years) of administered opioids were effective in reducing pain scores by two points on the CPOT.

**Discussion/Conclusions**: These findings support that CPOT is still used on a regular basis three and five years after its implementation. Pain reassessments should be improved.

## Investigating the Consequences of Living with A Parent with Chronic Pain on Their Children: A Qualitative Study

Somayyeh Mohammadi^a^^b^, Christine T. Chambers 0000-0002-7138-916X^a^^b^^c^, Margot Latimer^d^, Lindsay L. Richter^a^^e^, and Lukas Propper^f^

^a^Centre for Pediatric Pain Research, IWK Health Centre, Halifax, Canada; ^b^Department of Pediatrics, Dalhousie University, Halifax, Canada; ^c^Department of Psychology and Neuroscience, Dalhousie University, Halifax, Canada; ^d^School of Nursing, Faculty of Health Professions, Dalhousie University, Halifax, Nova Scotia, Canada; ^e^Department of Obstetrics and Gynecology, University of British Columbia, Vancouver, Canada; ^f^Department of Psychiatry, Dalhousie University, Halifax, Nova Scotia, Canada

**CONTACT** Somayyeh Mohammadi somayyeh.mohammadi@iwk.nshealth.ca

© 2019 The Author(s). Published with license by Taylor & Francis Group, LLC.

This is an Open Access article distributed under the terms of the Creative Commons Attribution License (http://creativecommons.org/licenses/by/4.0/), which permits unrestricted use, distribution, and reproduction in any medium, provided the original work is properly cited.

**Introduction**: There is a higher risk of behavioral and emotional symptoms (e.g., anxiety) in children of parents with chronic pain compared to their peers. This qualitative study investigated the challenges that children of parents with chronic pain face in their daily life and the coping strategies that they use to overcome these challenges.

**Method**: Fifteen children (9 females; 12–17 years old) who live with parents with chronic pain (pain > 3 months) participated in an individual semi-structured interview over the phone. Children answered questions regarding their understanding of their parent’s pain, its impacts on their daily life and their coping strategies. The interviews were transcribed verbatim and analyzed using the phenomenologic approach.

**Result**: Five categories emerged from the data: 1) child’s incomplete understanding about their parent’s chronic pain, 2) noticing something is different when the parent is in pain, 3) growth of personal and interpersonal skills because of living with a parent with pain, 4) parent’s pain limits child’s social life, and 5) experiencing emotional distress because of parents’ pain.

**Discussion**: The results enhance our knowledge on the impact of parental chronic pain on their children and the variety of coping strategies that children use to overcome these challenges. Moreover, the interviews revealed certain positive impacts of living with parents with chronic pain including the development of good personal and interpersonal skills. These findings can be used to develop interventions to support parents and their children to better understand the pain and its multidimensional consequences on the children.

## *Blastocystis* Infection is Associated with Colonic Hypersensitivity and Intestinal Dysbiosis

Manon Defaye^a^, Céline Nourrisson^b^, Elodie Baudu^c^, Amandine Lashermes^d^, Ivan Wawrzyniak^a^, Virginie Bonnin^c^, Nicolas Barnich^c^, Denis Ardid^d^, Mathilde Bonnet^c^, Frédéric Delbac^a^, Frédéric Carvalho^d^, Philippe Poirier^b^

^a^Université Clermont Auvergne, CNRS U6023 LMGE, Clermont-Ferrand, France; ^b^Université Clermont Auvergne, CNRS U6023 LMGE, CHU, Clermont-Ferrand, France; ^c^Université Clermont Auvergne, Inserm U1071, USC INRA 2018, M2iSH, Clermont-Ferrand, France; ^d^Université Clermont Auvergne, Inserm U1107 NeuroDol, Clermont-Ferrand, France

**CONTACT** Manon Defaye manon.defaye1@ucalgary.ca

© 2019 The Author(s). Published with license by Taylor & Francis Group, LLC.

This is an Open Access article distributed under the terms of the Creative Commons Attribution License (http://creativecommons.org/licenses/by/4.0/), which permits unrestricted use, distribution, and reproduction in any medium, provided the original work is properly cited.

**Introduction/Aim:** Infectious gastroenteritis is a risk factor for development of Irritable Bowel Syndrome (post-infectious-IBS). Recent clinical studies described a high prevalence of *Blastocystis* in IBS patients. Here we investigated using an animal model the link between *Blastocystis*, colonic hypersensitivity (CHS) and microbiome changes.

**Methods:** Rats were orally infected with *Blastocystis* subtype 4 cysts. Colonic sensitivity was evaluated by colorectal distension one month post-infection. Animal behavior was assessed using a behavioral recognition system (PhenoTyper®), Elevated Plus Maze and Forced Swimming test. Feces were collected to study microbiota composition by 16S Illumina® sequencing and for metabolite analyses.

**Results:** We found that *Blastocystis* ST4 induced non inflammatory CHS in infected rats. We have demonstrated an increase in fecal serine protease activity in infected rats which may explain development of CHS. In addition infected rats developed anxiety- and depressive-like behaviors correlated with CHS. Infection induced intestinal dysbiosis was characterized by increased bacterial richness and decreased *Firmicutes/Bacteroidetes* ratio. Interestingly, we correlated the CHS with the increase in the relative abundance of the genus of *Bacteroides* and the decrease in the relative abundance of the family of *Clostridiaceae*, some bacteria producing Short Chain Fatty Acids (SCFAs). Indeed, fecal SCFAs levels were decreased in infected rats.

**Discussion/Conclusions:** Our data suggest that *Blastocystis* infection in rat mimics IBS with the establishment of a CHS linked to microbiota and metabolic shifts. Thus, this new infectious model could therefore contribute to a better diagnosis and development of new therapeutic strategies for chronic gastrointestinal disorders.

### Acknowledgments

This work was achieved by obtaining a co-financing Region Auvergne-Rhône-Alpes and FEDER in 2015 (“Thématiques émergentes”).

## Antibiotic Treatment Slows Recovery of Mechanical Hypersensitivity for Males but Not Females in an Incision Model of Pain

Katherine Halievski^a^, Michael W. Salter^a^^b^

^a^The Hospital for Sick Children, Neurosciences and Mental Health, Toronto, Ontario, Canada; ^b^Physiology, University of Toronto, Toronto, Ontario, Canada

**CONTACT** Katherine Halievski k.halievski@gmail.com

© 2019 The Author(s). Published with license by Taylor & Francis Group, LLC.

This is an Open Access article distributed under the terms of the Creative Commons Attribution License (http://creativecommons.org/licenses/by/4.0/), which permits unrestricted use, distribution, and reproduction in any medium, provided the original work is properly cited.

**Introduction/Aim:** Treatment options for chronic pain are limited owing in large part to individual allelic, sex, and microbiota differences. Indeed it is likely that multiple factors together determine individual differences.

**Methods:** To explore whether a sex difference exists in the role of microbiota in different painful conditions, we subjected male and female mice to broad-spectrum oral antibiotics for three weeks prior to either a hindpaw incision or a spared nerve injury (SNI). We then assessed pain using a battery of nociceptive behavioural tests: mechanical hypersensitivity, dynamic weight bearing, and thermal hypersensitivity.

**Results:** While all mice responded to the antibiotics as each developed a grossly enlarged caecum, antibiotic treatment alone did not alter mechanical or thermal sensitivity. However, after hindpaw incision we found that mechanical withdrawal thresholds of male mice showed slower recovery to baseline than those of vehicle-treated males. Antibiotic-treated males also showed an increased rear-load in dynamic weight bearing testing compared to vehicle-treated males. Thermal hypersensitivity did not differ between vehicle- and antibiotic-treated males. After SNI, vehicle- and antibiotic-treated male mice showed no behavioural differences using this battery of tests. Antibiotic-treated female mice were not different than vehicle-treated females in any assay after either hindpaw incision or SNI.

**Discussion/Conclusions:** Thus, long-term antibiotic treatment affects behavioural responses in an acute pain model in a sex-specific manner.

## A Comparison of the Directional and Concurrent Relationships between Behavioural and Cardiac Indicators of Vaccination Pain at 12 Months

Jordana Waxman^a^, Miranda DiLorenzo^a^, Rebecca Pillai Riddell^a^, Hartley Garfield^b^

^a^Psychology, York University, Toronto, ON, Canada; ^b^Pediatrics, University of Toronto, Toronto, ON, Canada

**CONTACT** Jordana Waxman waxmanja@yorku.ca

© 2019 The Author(s). Published with license by Taylor & Francis Group, LLC.

This is an Open Access article distributed under the terms of the Creative Commons Attribution License (http://creativecommons.org/licenses/by/4.0/), which permits unrestricted use, distribution, and reproduction in any medium, provided the original work is properly cited.

**Introduction/Aim:** The aim of this study is to provide preliminary comparative data on the relationship between healthy infants’ pain behaviour (FLACC) and commonly used cardiac indicators (i.e. heart rate [HR], respiratory sinus arrhythmia [RSA]) during 12-month vaccinations.

**Methods:** Caregiver-infant dyads were part of an ongoing cohort followed at 12-, 18- and 24-month vaccinations. Behavioural and cardiac data were simultaneously collected, and later coded/analyzed for FLACC and HR/RSA during four 30-s epochs (30-s pre-needle, immediately post-needle, 1-minute and 2-minutes post-needle). Two cross-lagged path models examined directional and reciprocal relationships between FLACC and HR or RSA during the 12-month vaccination (n = 82).

**Results:** Results indicated that: 1) Previous FLACC scores (or HR) predict future FLACC scores (or HR) across epochs (FLACC *b* = .23–1.12; HR *b* = .62-.7). Previous RSA only predicts future RSA in post-needle epochs (*b* = .22–42); 2) FLACC scores immediately (*b = *3.40) and 1-minute post-needle (*b = −1*.13) predict subsequent HR post-needle. Pre-needle FLACC scores predict RSA immediately post-needle (*b = −.2*4); and 3) Concurrent relationships between FLACC and HR were found in all but the immediate post-needle epoch (*b* = 13.86–20.55), while negative relations between FLACC and RSA were found in all epochs except 2-minutes post-needle (*b = −1*.53- [−.73]).

**Discussion/Conclusions:** Preliminary analyses suggest behaviour and cardiac indicators converge during most concurrent epochs. There were also strong indications of within-measure prediction and cross-lagged relationships between behaviour and cardiac indicators. Results suggest that behavioural and cardiac indicators may be capturing unique aspects of the nociceptive response.

## Associations between Parental Psychopathology and Infant Pain-Related Distress Behaviour: Is Infant Temperament a Moderator?

Shaylea Badovinac^a^, Hannah Gennis^a^, Rebecca Pillai Riddell^a^, Hartley Garfield^b^

^a^Clinical-Developmental Psychology, York University, Toronto, Ontario, Canada; ^b^Pediatrics, University of Toronto, Toronto, Ontario, Canada

**CONTACT** Shaylea Badovinac sdbadov@yorku.ca

© 2019 The Author(s). Published with license by Taylor & Francis Group, LLC.

This is an Open Access article distributed under the terms of the Creative Commons Attribution License (http://creativecommons.org/licenses/by/4.0/), which permits unrestricted use, distribution, and reproduction in any medium, provided the original work is properly cited.

**Introduction/Aim:** Previous studies have shown that infant temperament (Stevens et al., 2013) and parental psychopathology (Moscardino, Axia, & Altoe, 2006) are independently associated with infants’ pain-related distress. The present study aimed to examine whether infant temperament moderates the association between parental psychopathology and infant pain-related distress reactivity and regulation during routine vaccinations.

**Methods:** The study included parent-infant dyads (*n* = 58) from the 12-month wave of an ongoing longitudinal study in which dyads were videotaped during infants’ routine vaccinations. Infant pain-related distress was coded from 15-second epochs of video footage collected immediately post-needle (reactivity) and at one minute (regulation 1) and two minutes (regulation 2) post-needle using the Face, Legs, Activity, Cry, Consolability Scale (FLACC; Merkel, Voepel-Lewis, Shayevitz, & Malviya, 1997). Infant negative affectivity and parental psychopathology were assessed using the Early Childhood Behavior Questionnaire (ECBQ; Putnam, Jacobs, Gartstein, & Rothbart, 2010) and the Brief Symptom Inventory 18 (Derogatis, 2001), respectively. Moderated regression models examined the contribution of infant negative affectivity, parental psychopathology, and their interaction, to the prediction of infant pain-related distress.

**Results:** The regression model did not account for a significant proportion of variance in infant pain-related distress behaviour during the reactivity or regulation phases. No main effects or moderation effects were found.

**Discussion/Conclusions:** Parent reports of psychopathology and infant temperament did not predict infants’ pain-related distress behaviours. This may be due to limitations associated with using a normative sample. It is critical to replicate this study in a higher risk or clinical sample.

## Does Integrative Medicine Reduce Prescribed Opioid Use for Chronic Pain? A Systematic Literature Review

Samah Hassan^a^^b^^c^, Qingping Zheng^a^, Erica Rizzolo^a^, Evrim Tezcanli^a^, Sukriti Bhardwaj^a^, Kieran Cooley^a^^d^^e^

^a^Canadian College of Naturopathic Medicine, ON, Canada; ^b^Institute of Medical Science, University of Toronto, ON, Canada; ^c^Toronto Rehabilitation Institute, University Health Network, ON, Canada; ^d^Australian Research Centre in Complementary and Integrative Medicine – University Technology Sydney, Ultimo, Australia; ^e^Pacific College of Oriental Medicine, San Diego, CA, USA

**CONTACT** Samah Hassan samahhassan@ccnm.edu

© 2019 The Author(s). Published with license by Taylor & Francis Group, LLC.

This is an Open Access article distributed under the terms of the Creative Commons Attribution License (http://creativecommons.org/licenses/by/4.0/), which permits unrestricted use, distribution, and reproduction in any medium, provided the original work is properly cited.

Chronic pain (CP) is a major public-health problem. Many patients with CP are increasingly prescribed opioids, which have led to an opioid crisis. Integrative Medicine (IM) which combines pharmacological and complementary and alternative medicine (CAM) has been proposed as opioid alternative for CP treatment. Nevertheless, many CAM therapies have been viewed with prejudice and skepticism based on claims of limited or low-quality evidence. To explore the true effectiveness of the IM approach or any of the CAM therapies to reduce or cease opioid use in CP patients

An online search of MEDLINE and Embase, CINAHL, PubMed supp. and Allied and Complementary Medicine Database (AMED) for studies published in English from inception until February 15th, 2018 was conducted. The Mixed Methods Appraisal Tool (MMAT) was used to critically appraise selected studies.

\The electronic search yielded 5200 citations. Twenty-three studies met all eligibility criteria and underwent data extraction. The majority of studies showed that opioid use was reduced significantly after using IM. Cannabinoids were among the most commonly investigated approaches in reducing opioid use followed by multidisciplinary approaches, cognitive-behavioural model and acupuncture. The majority of the studies had limitations with sample size, duration and study designs.

There is a small, but defined body of literature demonstrating positive preliminary evidence that IM approach including CAM therapies can help in reducing opioid use. As the opioid crisis continues to grow, it’s vital that clinicians and patients be adequately informed regarding the evidence and opportunities for IM/CAM therapies for CP.

**Introduction/Aim:** Chronic pain (CP) is a major public-health problem. Many patients with CP are increasingly prescribed opioids, which have led to an opioid crisis. Integrative Medicine (IM) which combines pharmacological and complementary and alternative medicine (CAM) has been proposed as opioid alternative for CP treatment. Nevertheless, many CAM therapies have been viewed with prejudice and skepticism based on claims of limited or low-quality evidence. To explore the true effectiveness of the IM approach or any of the CAM therapies to reduce or cease opioid use in CP patients

**Methods:** An online search of MEDLINE and Embase, CINAHL, PubMed supp. and Allied and Complementary Medicine Database (AMED) for studies published in English from inception until February 15th, 2018 was conducted. The Mixed Methods Appraisal Tool (MMAT) was used to critically appraise selected studies.

**Results:** The electronic search yielded 5200 citations. Twenty-three studies met all eligibility criteria and underwent data extraction. The majority of studies showed that opioid use was reduced significantly after using IM. Cannabinoids were among the most commonly investigated approaches in reducing opioid use followed by multidisciplinary approaches, cognitive-behavioural model and acupuncture. The majority of the studies had limitations with sample size, duration and study designs.

**Discussion/Conclusions:** There is a small, but defined body of literature demonstrating positive preliminary evidence that IM approach including CAM therapies can help in reducing opioid use. As the opioid crisis continues to grow, it’s vital that clinicians and patients be adequately informed regarding the evidence and opportunities for IM/CAM therapies for CP.

## Cannabis and the Opioid Crisis: Scoping the Literature to Understand the Relationship between Cannabis and Opioid Use

Nancy Carnide^a^, Morgane Le Pouésard^a^, Emma Irvin^a^, Dwayne Van Eerd^a^, Heather Johnston^a^, Quenby Mahood^a^, Margaret Tiong^a^, Zoe Sinkins^b^, Christa Orchard^c^, Sudaba Popal, Nimish Mittal^d^, Siobhan Cardoso^a^, Andrea D. Furlan^a^^e^^d^

^a^Institute for Work & Health, Toronto, Ontario, Canada; ^b^McMaster University, Hamilton, Ontario, Canada; ^c^Dalla Lana School of Public Health, University of Toronto, Toronto, Ontario, Canada; ^d^Toronto Rehab-University Health Network, Toronto, Ontario, Canada; ^e^Department of Medicine, University of Toronto

**CONTACT** Nancy Carnide ncarnide@iwh.on.ca

© 2019 The Author(s). Published with license by Taylor & Francis Group, LLC.

This is an Open Access article distributed under the terms of the Creative Commons Attribution License (http://creativecommons.org/licenses/by/4.0/), which permits unrestricted use, distribution, and reproduction in any medium, provided the original work is properly cited.

**Introduction/Aim**: Canada is in the middle of a public health crisis related to opioids that shows no signs of abating. The social and legislative landscape around cannabis in Canada is evolving. A synthesis of the data on the potential intersection between cannabis use and opioid outcomes is urgently needed at this pivotal time. The aim of this study was to conduct a scoping review to develop a broad understanding of the relationship between cannabis and opioid use and related outcomes

**Methods**: We used scoping review methods to search, select, and summarize published studies conducted in the past 20 years.

**Results**: We included 81 studies that reported on the relationship between a variety of cannabis measures and a range of opioid-related outcomes. Within the Prevention Pillar, a total of 30 studies showed no association for prescription synthetic cannabinoids, as well as mixed results for medical cannabis, cannabis legislation, and non-medical cannabis use. Within the Treatment Pillar, a total of 17 studies showed mixed results for prescription synthetic cannabinoids, medical cannabis use, and non-medical cannabis use. Within the Prevention/Harm Reduction Pillar, a total of 34 studies showed no studies for synthetic cannabinoids or cannabis legislation, and mixed results for medical cannabis and non-medical cannabis use.

**Discusson/Conclusions**: As this is a scoping review, we cannot make policy or practice recommendations; however, the findings of this review can point to gaps in research that should be addressed in order to make future recommendations based on high-quality evidence.

## Prospective Analysis of Sensitivity to Physical Activity among Adults with Recent Onset Low Back Pain

Arthur Woznowski-Vu^a^, Daniel Flegg^a^, Rebekah Wickens^b^, Andrea Aternali^c^, Karen Meyer^d^, Michael Sullivan 0000-0002-4228-1678^e^, Timothy H. Wideman^a^

^a^School of Physical and Occupational Therapy, McGill University, Montreal, Quebec, Canada; ^b^Department of Psychology, Université du Québec à Montréal (UQAM), Montreal, Quebec, Canada; ^c^Department of Psychology, York University, Montreal, Quebec, Canada; ^d^CBI Health Group (Concordia Physio Sport), Montreal, Quebec, Canada; ^e^Department of Psychology, McGill University, Montreal, Quebec, Canada

**CONTACT** Arthur Woznowski-Vu arthur.woznowskivu@mail.mcgill.ca

© 2019 The Author(s). Published with license by Taylor & Francis Group, LLC.

This is an Open Access article distributed under the terms of the Creative Commons Attribution License (http://creativecommons.org/licenses/by/4.0/), which permits unrestricted use, distribution, and reproduction in any medium, provided the original work is properly cited.

**Introduction/Aim:** Recently developed measures for sensitivity to physical activity (SPA) use brief physical tasks and monitor evoked pain-related responses. However, these measures have not yet been studied prospectively. The purpose of this study was to estimate the extent to which SPA predicts pain and disability among adults with recent onset low back pain (<6 months), cross-sectionally and prospectively (3-month follow-up).

**Methods:** SPA was assessed using a repeated lifting task with difficulty tailored to personal pain responses. SPA-related measures included evoked pain (single lift, 10 repeated lifts), pre-post pressure pain threshold (hands, lower back), and task-specific questions (catastrophizing, fear, pain self-efficacy). Otherwise, participants answered questionnaires (Demographic, Pain Catastrophizing Scale, 11-item Tampa Scale of Kinesophobia, Brief Pain Inventory, Pain Disability Index). 3-month follow-up was completed by telephone (pain and disability questionnaires).

**Results:** Hierarchical regression analysis on 72 participants (preliminary analysis). Pain evoked with 10 lifts uniquely predicted pain at initial visit (β = .383,*t* = 3.587,p = .001) and 3-month follow-up (β = .268,*t* = 2.049,p = .044), beyond pain evoked with single lift. Pain evoked with 10 lifts uniquely predicted disability at initial visit (β = .283,*t* = 2.570,p = .012) beyond pain evoked with single lift, but not at 3-month. Task-specific catastrophizing uniquely predicted disability at 3-month (β = .298,*t* = 2.701,p = .009) beyond general pain catastrophizing, but not cross-sectionally. The pre-post change in pressure pain threshold (lower back) uniquely predicted disability at 3-month follow-up (β = −.271,*t* = −2.357,p = .021), but not cross-sectionally.

**Discussion/Conclusions:** This is the first study which prospectively analyzed the predictive value of SPA. Also, this is among the first studies to consider task-specific evoked psychological responses as part of a physical task-based SPA measure.

## Using Virtual Reality to Reduce Procedural Pain and Distress in Children with Cancer: A Feasibility Pilot Randomized Controlled Trial

Celia Cassiani^a^, Abla Oussama^b^, Kathryn Birnie 0000-0002-8223-8834^a^, Lindsay Jibb 0000-0001-6995-2825^c^, Karyn Positano^b^, Sarah Lloyd^a^, Jennifer Stinson^a^

^a^Child Health Evaluative Sciences, The Hospital for Sick Children, Toronto, Ontario, Canada; ^b^Haematology/Oncology, The Hospital for Sick Children, Toronto, Ontario, Canada; ^c^Faculty of Health Science, University of Ottawa, Ottawa, Ontario, Canada

**CONTACT** Celia Cassiani celia.cassiani@sickkids.ca

© 2019 The Author(s). Published with license by Taylor & Francis Group, LLC.

This is an Open Access article distributed under the terms of the Creative Commons Attribution License (http://creativecommons.org/licenses/by/4.0/), which permits unrestricted use, distribution, and reproduction in any medium, provided the original work is properly cited.

**Introduction/Aim**: Children with cancer have reported pain and distress during needle insertion into a subcutaneous port (SCP) despite numbing with a topical anesthetic.1–4 Virtual reality (VR) has shown to reduce acute pain in adults and children.5,6 Research is limited surrounding the use and safety of VR for procedural pain management for children with cancer.^7^ This study aims to determine the feasibility (i.e., accrual rate, reasons for non-participation, safety, acceptability) of implementing a randomized controlled trial (RCT) of VR for SCP needle insertion in children with cancer and provide estimates of the impact on pain and distress.

**Methods**: A pilot RCT comparing an intervention (VR headset) to an active control group (iPad) during SCP needle insertion in children 8–18 with cancer. Feasibility data will be analyzed through descriptive statistics.

**Results**: Recruitment and data analysis are ongoing. Currently, 119 patients were screened for eligibility, 48 were eligible, 36 were approached, and 22 enrolled. Participants cited “not interested”, or “comfortable without distraction” as reasons for non-participation. Average age of participants is 12.8 years (SD = 3.2). Nine (75%) participants enjoyed using the VR, 7 (58%) found the VR headset a helpful distraction, and 11(91.7%) nurses were satisfied with the overall experience of using VR during port access. No safety issues were reported.

**Conclusion**: Preliminary results suggest that VR during port insertion for children with cancer is feasible, safe, and acceptable for children with cancer and their nurses. Next steps include analyzing feasibility and effectiveness outcomes before completing a definitive trial.

References1.
Ljungman
G, Kreuger
A, Gordh
T, Berg
T, Sörensen
S, Rawal
N. Treatment of pain in pediatric oncology: a Swedish nationwide survey. Pain. 1996;68:385–94. doi:
10.1016/S0304-3959(96)03193-4.91218282.
Ljungman
G, Gordh
T, Sörensen
S, Kreuger
A. Pain variations during cancer treatment in children: a descrip-tive survey. Pediatr Hematol Oncol. 2000;17:211–21. doi:
10.1080/088800100276389.107799873.
Collins
JJ. Cancer pain management in children. Eur J Pain. 2001;5(Suppl A):37–41. doi:
10.1053/eujp.2001.0278.117982164.
Lüllmann
B, Calleja
N, Decelis
A. Pain reduction in children during port-à-cath catheter puncture using local anaesthesia with EMLA™. Eur J Pediatr. 2010;169:1465–69. doi:
10.1007/s00431-010-1185-8.206232335.
Law
EF, Dahlquist
LM, Sil
S, Weiss
KE, Herbert
LJ, Wohlheiter
K, Horn
SB. Videogame distraction using virtual reality technology for children experiencing cold pressor pain: the role of cognitive processing. J Pediatr Psychol. 2011;36:84–94. doi:
10.1093/jpepsy/jsq063.20656761PMC31075856.
Hua
Y, Qiu
R, Yao
W-Y, Zhang
Q, Chen
X-L. The effect of virtual reality distraction on pain relief during dressing changes in children with chronic wounds on lower limbs. Pain Manag Nurs. 2015;16:685–91. doi:
10.1016/j.pmn.2015.03.001.259720747.
Jibb
L, Nathan
P, Stevens
B, Seto, Cafazzo
J, Stephens
N, Yohannes
L, Stinson
J. Psychological and physical interventions for the management of cancer-related pain in pediatric and young adult patients: an integrative review. Oncol Nurs Forum. 2015;42:E339–E357. doi:
10.1188/15.ONF.E339-E357.26488841

## Updates from the Chronic Pain Network

Norm Buckley^a^, Kim Begley^b^, Megan Groves^b^

^a^Department of Anesthesia, Michael G DeGroote School of Medicine, McMaster University, Hamilton, Ontario, Canada; ^b^Department of Anesthesia, Chronic Pain Network, Hamilton, Ontario, Canada

**CONTACT** Norman Buckley buckleyn@mcmaster.ca

© 2019 The Author(s). Published with license by Taylor & Francis Group, LLC.

This is an Open Access article distributed under the terms of the Creative Commons Attribution License (http://creativecommons.org/licenses/by/4.0/), which permits unrestricted use, distribution, and reproduction in any medium, provided the original work is properly cited.

**Introduction/Aim:** The CIHR Strategy for Patient Oriented Research engages patients in research from priority setting to knowledge translation. Chronic Pain Network’s (CPN) mission is to change the way pain is managed in Canada. The Network supports, coordinates and synchronizes leading, innovative and high impact patient-oriented research and creates opportunities for collaboration across the country.

**Methods:** Projects are aligned with patient derived priorities (Poulin et al., 2018) and are reviewed twice yearly by the Patient Oriented Research committee for milestone achievement, patient engagement, training and mentoring and knowledge translation. Patient partners are joining project teams and serve on governance committees. The 12 Clinical Research Network sites have 10 full time research coordinators in place.

**Results:** The Network currently has twenty-seven projects that align with identified patient priorities in the areas of:

- Clinical trials testing new ways to prevent, detect or manage diseases (9)
- Basic science encompassing the fundamental work to advance knowledge (8)
- Population Studies addressing groups who share a common characteristic such as sex or health conditions (6)
- Behavioural Science in multiple contexts (4)

**Discussion/Conclusions:** Some projects have modified due to budget restrictions; all are on track logistically; new initiatives have arisen including providing a strategy for pain management in hemophilia (underway), and a national discussion on biomarkers (Feb 2019). CPN patient groups have been recognized as a national resource for Health Canada in its Opioid Response, and support for furthering the Canadian Pain Strategy has arisen from CPN activities.

## Pain Assessment Documentation in Adult Intensive Care Units: A Lot of Room for Improvement!

Céline Gélinas^a^, Melissa Richard-Lalonde^a^, Francis Bernard^b^, Mélanie Bérubé^c^, Emmanuel Charbonney^d^, Jean-Nicolas Dubé^e^, Julie Houle^f^, Suzanne Morin^g^, Marc Perreault^h^, Nathalie Thiffault^i^, Yannick Tousignant-Laflamme^j^, Darina M. Tsoller^k^, Virginie Williams^l^, David Williamson^m^, Manon Choinière 0000-0001-9593-8883^n^

^a^Ingram School of Nursing, and Jewish General Hospital - CIUSSS Centre-Ouest-Ile-Montreal, Centre for Nursing Research and Lady Davis Institute, McGill University, Montréal, Québec, Canada; ^b^CIUSSS du Nord-de-l’Île-de-Montréal, Hôpital du Sacré-Cœur de Montréal, Département des soins intensifs, Université de Montréal, Montréal, Québec, Canada; ^c^CIUSSS du Nord-de-l’Île-de-Montréal, Hôpital du Sacré-Coeur de Montréal, Montréal, Québec, Canada; ^d^CIUSSS de la Mauricie-et-Centre-du-Québec, CIUSSS du Nord-de-l’Île-de-Montréal, Université de Montréal, Montréal, Québec, Canada; ^e^Centre hospitalier affilié universitaire régional - CIUSSS de la Mauricie-et-Centre-du-Québec, Université de Montréal, Trois-Rivières, Québec, Canada; ^f^Département des sciences infirmières, CIUSSS de la Mauricie-et-Centre-du-Québec, Université du Québec à Trois-Rivières, Trois-Rivières, Québec, Canada; ^g^Department of Medicine, McGill University, Montréal, Québec, Canada; ^h^Faculty of Pharmacy, McGill University Health Center, and Université de Montréal, Montréal, Québec, Canada; ^i^Centre hospitalier affilié universitaire régional - CIUSSS de la Mauricie-et-Centre-du-Québec, Trois-Rivières, Québec, Canada; ^j^School of Rehabilitation, Université de Sherbrooke, Sherbrooke, Québec, Canada; ^k^Jewish General Hospital - CIUSSS Centre-Ouest-Ile-Montreal, Centre for Nursing Research and Lady Davis Institute, Montréal, Québec, Canada; ^l^Département des soins intensifs, CIUSSS du Nord-de-l’île-de-Montréal, Hôpital du Sacré-Cœur de Montréal, Montréal, Québec, Canada; ^m^Faculty of Pharmacy, CIUSSS Nord-de-l’Ile-de-Montréal, Research Centre and Hôpital du Sacré-Coeur de Montréal, Université de Montréal, Montréal, Québec, Canada; ^n^Department of Anesthesiology and Pain Medicine, Research Centre of the Centre hospitalier de l’Université de Montréal and Université de Montréal, Montréal, Québec, Canada

**CONTACT** Céline Gélinas celine.gelinas@mcgill.ca

© 2019 The Author(s). Published with license by Taylor & Francis Group, LLC.

This is an Open Access article distributed under the terms of the Creative Commons Attribution License (http://creativecommons.org/licenses/by/4.0/), which permits unrestricted use, distribution, and reproduction in any medium, provided the original work is properly cited.

**Introduction/Aim**: Practice guidelines recommend that pain be assessed on a regular basis (q 2-3 h) in the intensive care unit (ICU) We aimed to describe pain assessment documentation in adult ICUs.^1^

**Methods**: A retrospective cohort design was used in this multi-site study of four ICU settings in the province of Quebec. Pain assessment tools (i.e., 0–10 Numeric Rating Scale and Critical-Care Pain Observation Tool) were available in all ICUs. Medical charts of patients admitted to the ICU for a minimum of 48 hours in 2017 and 2018 were reviewed and we extracted data from the second 24-hour period following ICU admission. Information related to socio-demographic (age, sex), clinical (diagnosis, ICU length of stay, mechanical ventilation duration), pain assessments, and opioid administration was collected.

**Results**: A total of 297 patient charts (31% male, mean age = 60 y, SD = 18.1) were reviewed. Admitting ICU diagnosis was medical (38%), surgical (26%) and trauma (36%). Median ICU length of stay was 6.6 days and median duration of mechanical ventilation (80% of patients were mechanically ventilated) was 74.7 hours. Pain assessments were documented in only 51% of reviewed charts, and when present the median number was 4 assessments/24 hours. Pain reassessments following opioid administration were documented in only 22% of patients who received opioids.

**Discussion/Conclusions**: Documentation of pain assessments in the ICU is suboptimal and opioid administration is not monitored with regular pain reassessments. Improvement strategies must be deployed to improve optimal documentation and to ensure pain management practices for relieving pain in the ICU.

**Disclosure statement**

No potential conflict of interest was reported by the authors.

Reference1.
Devlin
JW, Skrobik
Y, Gelinas
C, Needham
DM, Slooter
AJC, Pandharipande
PP, … Alhazzani
W. Clinical practice guidelines for the prevention and management of pain, agitation/ sedation,delirium, immobility, and sleep disruption in adult patients in the ICU. Crit Care Med. 2018;46(9):e825–e873. doi:
10.1097/CCM.0000000000003299.30113379

## A Cumulative Impact of Psychological and Sensitization Risk Factors on Pain-Related Outcomes

Zakir Uddin^a^, Arthur Woznowski-Vu^a^, Daniel Flegg^a^, Andrea Aternali^b^, Timothy H. Wideman^a^

^a^School of Physical and Occupational Therapy, McGill University, Montreal, Quebec, Canada; ^b^Psychology, McGill University, Montreal, Quebec, Canada

© 2019 The Author(s). Published with license by Taylor & Francis Group, LLC.

This is an Open Access article distributed under the terms of the Creative Commons Attribution License (http://creativecommons.org/licenses/by/4.0/), which permits unrestricted use, distribution, and reproduction in any medium, provided the original work is properly cited.

**Introduction/Aim**: The relationship between pain catastrophizing (PC) and pressure pain threshold (PPT) is not clear as of existing controversy and insufficient studies. Although, both are evidence-based risk factors measuring tools in research and clinical practice. A cumulative impact evaluation may help us to understand this relationship and their clinical usability. The aim of this study is to evaluate the cumulative impact of psychological and sensitization risk factors on pain-related outcomes (activity avoidance, pain severity and disability). We hypothesized people with both high catastrophizing and high sensitivity most vulnerable.

**Methods**: We included 109 participants (70.60% women; mean ± SD age 53.6 ± 12.3 years) with chronic musculoskeletal pain for data analysis who completed all measures of this study.

Participants completed a single testing session that included measures of risk factors (PC and PPT) and pain-related outcomes (self-reported avoidance, functional avoidance, disability and pain severity). Subgroups were constructed by dichotomizing (median split) of PC and PPT scores, resulting in 4 groups: 1. high catastrophizing and low sensitivity (N = 27), 2. high catastrophizing and high sensitivity (N = 31), 3. low catastrophizing and low sensitivity (N = 26), and 4. low catastrophizing and high sensitivity (N = 25).

**Results**: Onaway ANOVA revealed significant group differences (p < .05, η^2^ = .08 to .14) on all outcomes of this study (except functional avoidance) and post hoc analysis indicated the significance differences are between group 2 and 3 (p < .05). ANCOVA identified gender as the significate covariant for the functional avoidance (p < .01, ηp^2^ = .19).

**Discussion/Conclusions**: The study suggests both higher level of pain catastrophizing and pressure sensitivity has a cumulative impact in risk screening for pain-related outcomes, considering gender in functional avoidance (task related outcome). This finding has important clinical and theoretical implications.

## Validating Skin Conductance for Assessing Pain and Stress in Mechanically Ventilated Infants

Jiale Hu 0000-0003-1993-8704^a^, Joann Harrold^b^, Janet Squires^c^, Shokoufeh Modanloo^a^, Denise Harrison 0000-0001-7549-7742^a^

^a^School of Nursing and Children’s Hospital of Eastern Ontario Research Institute, University of Ottawa, Ottawa, Ontario, Canada; ^b^Children’s Hospital of Eastern Ontario and Ottawa Hospital, Faculty of Medicine & Neonatology, University of Ottawa, Ottawa, Ontario, Canada; ^c^School of Nursing and Ottawa Hospital Research Institute, University of Ottawa, Ottawa, Ontario, Canada

**CONTACT** Jiale Hu jhu081@uottawa.ca

© 2019 The Author(s). Published with license by Taylor & Francis Group, LLC.

This is an Open Access article distributed under the terms of the Creative Commons Attribution License (http://creativecommons.org/licenses/by/4.0/), which permits unrestricted use, distribution, and reproduction in any medium, provided the original work is properly cited.

**Introduction/Aim**: Measuring pain in mechanically ventilated infants is challenging. The measurement of skin conductance (SC) is based on the sympathetic nervous system response to stress. The study aimed to evaluate the validity of SC for assessing pain and stress in mechanically ventilated infants.

**Methods:** A prospective cross-sectional observational design to study SC and its relation to: 1) type of procedure (painful or non-painful), 2) phase of procedure (before, during and after procedure), and 3) referent pain measures (the Premature Infant Pain Profile-Revised (PIPP-R) and Neonatal Facial Coding System (NFCS)). Eligibility criteria: Infants up to 12 months, in the intensive care units, who were mechanically ventilated, and required a painful and non-painful procedure.

**Results:** From October 2017 to November 2018, 130 eligible infants were identified and 55 (30 male and 25 female) infants were studied. SC (number of waves per second) was statistically significantly different between during painful (median 0.27; interquartile range 0.20–0.40) and non-painful procedures (0; 0–0.09). These values during procedures were statistically significantly higher than the SC before (0; 0–0.07) and after painful (0; 0–0.07) or before (0; 0–0.02) and after non-painful procedures (0; 0–0.07). SC statistically significantly correlated with PIPP-R (Spearman’s rho = 0.73) and NFCS (0.67) during painful procedures, and with PIPP-R (0.50) and NFCS (0.44) during non-painful procedures.

**Discussion/Conclusions:** The study showed the validity of SC in relation to the type of procedure, the phase of procedure and the referent pain measures. SC is a potential approach to assessing pain and stress in sick infants requiring machinal ventilation.

## EEG-Based Functional Connectivity – A Possible Biomarker for Neuropathic Pain in DPN

L. Shafran Topaz^a^, A. Frid^a^, Y. Granovsky^a^^b^, S. Shapira^a^, L. Meir -Yalon^a^, C. Buxbaum^b^, N. Bosak^b^, E. Domany^b^, R. Hadad^b^, M. Khamaisi^a^^b^, D. Yarnitsky^a^^b^

^1^The Bruce Rappaport Faculty of Medicine, Technion, Israel Institute of Technology, Haifa, Israel; ^2^Neurology Department, Rambam Health Care Campus, Haifa, Israel

**CONTACT** L. Shafran Topaz leah0883@gmail.com

© 2019 The Author(s). Published with license by Taylor & Francis Group, LLC.

This is an Open Access article distributed under the terms of the Creative Commons Attribution-NonCommercial License (http://creativecommons.org/licenses/by-nc/4.0/), which permits unrestricted non-commercial use, distribution, and reproduction in any medium, provided the original work is properly cited.

**Background**: The reason some diabetic polyneuropathy (DPN) patients develop neuropathic pain while others present with painless symptoms is unknown. Altered central pain processing has been associated with neuropathic pain. Previous EEG studies found unique cortical patterns in patients with neuropathic pain however these studies were limited to univariate analysis of locally activated areas of cortex. It has been proposed that pain is encoded by complex activity and connectivity patterns which can be explored by application of Machine Learning techniques on the EEG signal. We aimed to investigate whether EEG based functional connectivity can be a potential biomarker for distinction between painful and non-painful DPN.

**Methods**: We recorded contact-heat pain evoked potentials (CHEPs) of 120 painful DPN patients (33F, 63.2 ± 9.9 yrs) and 38 non-painful DPN patients (7F, 64.3 ± 9.5 yrs). Connectivity analysis was conducted based on a measure of synchrony (Phase Locking Value) between the recording sites. We then applied a data-driven analysis scheme to identify the most differentiating functional connections in each frequency band. These connections were validated and used to predict neuropathic pain.

**Results**: We found overall higher cortical functional connectivity between areas of the pain matrix among painful DPN patients. In alpha and theta bands, the connectivity values were significantly different, and differentiated between the two groups with fair-to-excellent specificity (0.797) and sensitivity (0.9).

**Conclusion**: Patients with DPN can be successfully classified into painful and non-painful based solely on EEG functional connectivity data. Increased connectivity between areas of the pain matrix in alpha and tetha band might be a biomarker for neuropathic pain.

### Acknowledgment

European Union’s Horizon 2020 research and innovation programme No. 633491.

## Interleukin-1β is a Therapeutic Target of Pain Hypersensitivity in a Model of Non-Compressive Disc Herniation

Milind M. Muley^a^, Yu Shan Tu^a^, Benjamin E. Steinberg^a^, Michael W. Salter^a^

Program in Neuroscience & Mental Health, Hospital for Sick Children, Toronto, Ontario, Canada

**CONTACT** Milind M. Muley milind.muley@sickkids.ca

© 2019 The Author(s). Published with license by Taylor & Francis Group, LLC.

This is an Open Access article distributed under the terms of the Creative Commons Attribution License (http://creativecommons.org/licenses/by/4.0/), which permits unrestricted use, distribution, and reproduction in any medium, provided the original work is properly cited.

**Introduction/Aim**: Low back pain secondary to disc herniation is a major health problem. Proinflammatory cytokines have been implicated in the pathogenesis of disc herniation. Here, we assessed the contribution of interleukin-1β (IL-1β) to the development of pain behaviours in a recently developed model of non-compressive disc herniation.

**Methods**: Nucleus pulposus (NP), collected from littermate tail intravertebral discs, was placed on the sciatic nerve of male C57BL/6 mice. In sham animals, only the sciatic nerve was exposed. von Frey algesiometry was used to assess mechanical allodynia. In separate cohorts, immunohistochemistry and PCR were performed on day 7 to respectively study the infiltration of inflammatory cells and IL-1β gene expression in nerve. The effect of the caspase-1 inhibitor, VX-765 (200 mg/kg i.p; days 0–3), on NP-induced pain hypersensitivity was studied. To gain mechanistic insight, we developed an in vitro assay to test the effect of VX-765 on IL-1β secretion from cultured human macrophages.

**Results**: We found that mechanical allodynia appeared on day 1 and persisted to day 7 following NP exposure to the sciatic nerve. We detected an increase in macrophage (F4/80) infiltration in and around the nerve at 1-week post-surgery in NP animals, compared with sham controls. We identified increased *IL-1β* gene expression in the sciatic nerves treated with NP compared with sham controls. Treatment of NP animals with VX-765 prevented mechanical allodynia as compared to vehicle-treated animals. It was observed that VX-765 prevented the release of IL-1β from cultured macrophages.

**Discussion/Conclusions**: These results indicate an important role for IL-1β secretion from macrophages during the development of pain associated with non-compressive disc herniation. Blocking IL-1β may be a viable strategy in treating pain associated with disc herniation.

## A Complement-Microglia Pathway Drives Spinal Inhibitory Synapse Loss in Neuropathic Pain

Noosha Yousefpour^a^, Shannon Tansley^b^, Samantha Locke^a^, Valerie Cabana^a^, Chengyang Wang^c^, Arkady Khoutorsky^d^, Yves De Koninck^e^, Alfredo Ribeiro-da-Silva^a^

^a^Pharmacology and Therapeutics, McGill University, Montreal, Quebec, Canada; ^b^Integrated Program in Neuroscience, McGill University, Montreal, Quebec, Canada; ^c^Psychology and Anesthesia, McGill University, Montreal, Quebec, Canada; ^d^Anesthesia, McGill University, Montreal, Quebec, Canada; ^e^Psychiatry and Neuroscience, Laval University, Quebec City, Quebec, Canada

**CONTACT** Sabrina Noosha Yousefpour noosha.yousefpour@mail.mcgill.ca

© 2019 The Author(s). Published with license by Taylor & Francis Group, LLC.

This is an Open Access article distributed under the terms of the Creative Commons Attribution-NonCommercial License (http://creativecommons.org/licenses/by-nc/4.0/), which permits unrestricted non-commercial use, distribution, and reproduction in any medium, provided the original work is properly cited.

**Introduction/Aim:** Inhibitory synapse loss in the dorsal horn of the spinal cord strongly correlates with pain hypersensitivity in animal models of neuropathic pain. This selective synapse loss potentially contributes to disinhibition of spinal cord nociceptive circuits and maintenance of neuropathic pain. The present study aims to elucidate the key mechanism underlying this inhibitory synapse loss. Specifically, we investigate the role of microglia and the complement system, a well characterized synaptic pruning pathway, in neuropathic pain.

**Methods:** In a mouse model of neuropathic pain, using immunohistochemistry in combination with high resolution light and electron microscopy, we analyzed the integrity of inhibitory and excitatory synapses and their colocalization with complement factors. To investigate microglial involvement in phagocytosing inhibitory presynaptic inputs we performed an engulfment assay. Lastly, we depleted spinal microglia and complement factors in neuropathic and control mice then assessed the effect of these manipulations on dorsal horn synapse loss and pain-related behaviour.

**Results:** In neuropathic mice, we found a reduction in the number of intact inhibitory synaptic structures. A significant proportion of the remaining synapses colocalized with complement factors C1q and C3. The engulfment assay showed that microglia phagocytosed inhibitory presynaptic compartments. Furthermore, microglia and complement depletion prevented inhibitory synapse loss and pain hypersensitivity.

**Discussion/Conclusions:** Together, these findings suggest that microglia contribute to disinhibition of spinal nociceptive circuits in neuropathic pain through engulfment of inhibitory synapses in the spinal dorsal horn. The selectivity of microglia-mediated synapse pruning in neuropathic pain is likely dependent on complement factors.

### TSD Symptoms as a Mediator in the Relationship between Pre-Sleep Arousal and Chronic Pain in Youth

Cara Nania^a^, Tessa Wihak^a^, Richelle Mychasiuk^a^^b^, Melanie Noel^a^^c^

^a^Psychology, University of Calgary, Calgary, Alberta, Canada; ^b^Neuroscience, Monash University, Melbourne, Australia; ^c^Anesthesia, Alberta Children’s Hospital Research Institute, Mathison Centre for Mental Health Research and Education, The Hotckiss Brain Institute, Calgary, Alberta, Canada

**CONTACT** Cara Nania cgnania@ucalgary.ca

© 2019 The Author(s). Published with license by Taylor & Francis Group, LLC.

This is an Open Access article distributed under the terms of the Creative Commons Attribution License (http://creativecommons.org/licenses/by/4.0/), which permits unrestricted use, distribution, and reproduction in any medium, provided the original work is properly cited.

**Introduction/Aim:** Pediatric chronic pain co-occurs at a high rate with both PTSD symptoms (PTSS) and sleep impairments. Noel et al. (2017) showed that sleep quality partially mediated the relationship between PTSS and pain intensity and interference in youth with chronic pain. To date, little is known about this co-occurrence and the potential mechanisms that underlie it. In a recent model, Holley et al. (2016) proposed that hyperarousal, a symptom of PTSD, is incompatible with sleep and may lead to worse pain outcomes in children with chronic pain. Thus, the aim of this study was to examine the mediating role of PTSS in the relationship between pre-sleep arousal and pain outcomes in children with chronic pain.

**Methods:** Eighty-four adolescents (10–18 years; 73% female) receiving tertiary-level treatment for chronic pain completed questionnaires that assessed PTSS, pre-sleep arousal, pain intensity, and pain unpleasantness. Mediation analyses were conducted using bootstrapping.

**Results:** PTSS mediated the relationship between pre-sleep arousal and pain intensity (*n* = 81, *ab* = 0.03, CI_BCa_ = 0.01 to 0.06). PTSS also mediated the relationship between pre-sleep arousal and pain unpleasantness (*n* = 81, *ab* = 0.03, CI_BCa_ = 0.01 to 0.04).

**Discussion/Conclusions:** PTSS mediated the relationship between pre-sleep arousal and pain intensity and unpleasantness. These findings suggest that PTSS may be a factor underlying the sleep-pain relationship in pediatric chronic pain. Therefore, PTSS is an important target for future interventions involving children with chronic pain. Longitudinal examination of these relationships is needed with the use of objective sleep measures.

### Spinal Inhibitory Synapse Loss in Arthritis via a Microglial-Complement Pathway

Samantha Locke^a^, Noosha Yousefpour^b^, Valerie Bourassa^a^, Alfredo Ribeiro-Da-Silva^c^

^a^Integrated Program in Neuroscience, McGill University, Montreal, Quebec, Canada; ^b^Pharmacology and Therapeutics, McGill University, Montreal, Quebec, Canada; ^c^Pharmacology and Therapeutics, McGill University, Montreal, Quebec, Canada

**CONTACT** Samantha Locke samantha.locke@mail.mcgill.ca

© 2019 The Author(s). Published with license by Taylor & Francis Group, LLC.

This is an Open Access article distributed under the terms of the Creative Commons Attribution License (http://creativecommons.org/licenses/by/4.0/), which permits unrestricted use, distribution, and reproduction in any medium, provided the original work is properly cited.

**Introduction/Aim:** There is an imperfect correlation between joint changes and pain in arthritis, suggesting that there may be mechanisms other than just overt inflammation or damage involved in arthritis pain. In neuropathic pain disinhibition and microglial effects have been well-described. Recently, complement-mediated microglial synapse removal has been described in development and CNS disorders. Here, we aim to demonstrate in models of arthritis that complement-mediated synaptic pruning of inhibitory terminals by microglia occurs.

**Methods:** We used monoiodoacetatate (MIA) and complete Freund’s adjuvant (CFA) to induce ankle-joint arthritis in rats combined with immunohistochemistry and microscopy.

**Results:** We observed: microgliosis in the ipsilateral dorsal horn, upregulated complement factors, decreased number of inhibitory terminals and more inhibitory terminal signal in the cytoplasm of phagocytic microglia in tissue from arthritis animals compared to control. Interestingly, complement initiating factor C1q co-localized at more inhibitory terminals than excitatory terminals.

**Discussion/Conclusions:** These data demonstrate that there are spinal changes in arthritis models that may alter the balance of spinal excitation and inhibition and contribute to pain in this disease.

### Anxiety Mediates the Relationship between Insomnia and Pediatric Chronic Pain over Time

Tessa Wihak^a^, Allison McPeak^b^, Richelle Mychasiuk^a^^c^, Melanie Noel^b^^d^

^a^Psychology, University of Calgary, Calgary, AB, Canada; ^b^Anesthesia, University of Calgary, Calgary, AB, Canada; ^c^Neuroscience, Monash University, Melbourne, Australia; ^d^Psychology, Alberta Children’s Hospital Research Institute, Mathison Centre for Mental Health Research and Education, The Hotchkiss Brain Institute, University of Calgary, Calgary, AB, Canada

**CONTACT** Tessa Wihak tessa.wihak@ucalgary.ca

© 2019 The Author(s). Published with license by Taylor & Francis Group, LLC.

This is an Open Access article distributed under the terms of the Creative Commons Attribution License (http://creativecommons.org/licenses/by/4.0/), which permits unrestricted use, distribution, and reproduction in any medium, provided the original work is properly cited.

**Introduction/Aim:** Youth with chronic pain are at risk of experiencing co-occurring internalizing mental health symptoms and insomnia which are associated with poorer quality of life and increased healthcare utilization. Preliminary research suggests that sleep problems precede chronic pain and that anxiety may mediate this relationship. However, little is known about the relationship over time. This study aimed to examine anxiety as a mediator in the insomnia-pain relationship in youth with chronic pain at baseline and 3-month follow-up.

**Methods:** Eighty-four youth (10–18 years; 73% female) with chronic pain completed self-report measures of anxiety symptoms, insomnia, and pain interference at baseline and 3-month follow-up. Participants also underwent 7 days of actigraphy monitoring to assess sleep efficiency at baseline. Three mediation models were tested: Model 1 (baseline insomnia, anxiety, and pain), Model 2 (3-month insomnia, anxiety, and pain), and Model 3 (baseline insomnia, anxiety, and sleep efficiency).

**Results:** Model 1 revealed that the relationship between insomnia and pain interference was mediated by anxiety (*n* = 84, *ab* = 0.22, CI_BCa_ = 0.05 to 0.44). Anxiety also mediated the relationship between insomnia and pain interference in Model 2 (*n* = 65, *ab* = 0.26, CI_BCa_ = 0.09 to 0.53). Anxiety did not mediate the relationship between sleep efficiency and pain interference in Model 3.

**Discussion/Conclusions:** Findings suggest that insomnia may worsen pain through alterations in anxiety symptoms and that this relationship is maintained over time. Further investigation using self-report versus actigraphy is warranted to better understand the co-occurrence of pain, sleep impairment, and anxiety.

### Non-Ionotropic NMDA Receptor Signaling Mediates the Reversal of Hyperalgesia by Spinal Reconsolidation

David He^a^, Abigail J. D’Souza^b^, Robert P. Bonin^b^

^a^Department of Anesthesia, University of Toronto, Toronto, ON, Canada; ^b^Department of Pharmaceutical Sciences, University of Toronto, Toronto, ON, Canada

**CONTACT** David He davidymhe@gmail.com

© 2019 The Author(s). Published with license by Taylor & Francis Group, LLC.

This is an Open Access article distributed under the terms of the Creative Commons Attribution License (http://creativecommons.org/licenses/by/4.0/), which permits unrestricted use, distribution, and reproduction in any medium, provided the original work is properly cited.

**Background and aim:** Pathological pain can arise from plastic changes in nociceptive networks of the spinal dorsal horn. We have previously shown that the reactivation of sensitized nociceptive networks triggers a process that parallels memory reconsolidation: a protein synthesis-dependent process in which memory traces are rendered labile and modifiable. However, it is unclear how this memory trace disruption is initiated or what its underlying mechanisms are. Here, we examine the role of non-canonical, non-ionotropic NMDA (NI-NMDA) receptor in the regulation of spinal synaptic plasticity and hyperalgesia, and whether NI-NMDA contributes to pain reconsolidation.

**Methods:** Mechanosensitivity was assessed in mice using von Frey filaments. In vitro electrophysiological studies of afferent input to spinal dorsal horn was assessed by measuring field post-synaptic potentials induced by electrical stimulation of dorsal roots in an isolated lumbar spinal cord preparation.

**Results:** The induction of NI-NMDA signalling reversed hyperalgesia induced by plantar injection of capsaicin or CFA. Similarly, in vitro electrophysiological studies of afferent input to the spinal dorsal horn showed that NI-NMDA signaling could reverse spinal LTP but had no effect in the absence of LTP. Finally, we linked NI-NMDA signaling to pain reconsolidation by demonstrating that the reversal of hyperalgesia and LTP through reconsolidation blockade and NI-NMDA involve similar downstream mechanisms to reverse hyperalgesia.

**Conclusions:** These findings reveal a novel role for NI-NMDA signalling in the regulation of spinal sensitization and hyperalgesia. We further demonstrate intriguing links between NI-NMDA signalling and pain reconsolidation that indicate a role of NI-NMDA signalling in pain reconsolidation.

### Pain and Post-Traumatic Stress Disorder Symptoms in Parents of Childhood Cancer Survivors

Michaela Patton^a^, Melanie Noel^a^, Melanie Khu^b^, Brooke Russell^a^, Alexandra Neville^a^, Fiona Schulte^c^

^a^Psychology, University of Calgary, Calgary, Alberta, Canada; ^b^Hematology, Oncology, and Transplant Program, Alberta Children’s Hospital, Calgary, Alberta, Canada; ^c^Cumming School of Medicine, University of Calgary, Calgary, Alberta, Canada

**CONTACT** Michaela Patton michaela.patton@ucalgary.ca

© 2019 The Author(s). Published with license by Taylor & Francis Group, LLC.

This is an Open Access article distributed under the terms of the Creative Commons Attribution License (http://creativecommons.org/licenses/by/4.0/), which permits unrestricted use, distribution, and reproduction in any medium, provided the original work is properly cited.

**Introduction/Aim:** Parents of children with cancer are at heightened risk for developing post-traumatic stress disorder symptoms (PTSS), which may co-occur with chronic pain. However, research has not yet explored the prevalence of chronic pain among parents of childhood cancer survivors and the impact of parental chronic pain on children. The current study aims to explore the prevalence of and relationships between PTSS and pain among parents of childhood cancer survivors.

**Methods:** Survivors of Acute Lymphoblastic Leukemia and one parent (n = 16, 100% female; current mean age [range] = 45.81 [35–57] years) were recruited from Alberta Children’s Hospital. Data collection is ongoing. Parents completed the Pain Questionnaire, Pain Catastrophizing Scale-Parent Proxy, and PTSD Checklist for DSM-5.

**Results:** Preliminary analyses found that 46.7% of parents reported having chronic pain. Parents reported a 2.26 (SD = 1.21) mean pain frequency (scale of 0–4), mean pain severity of 4.27 (SD = 1.49) (scale of 0–7), and mean PTSS score of 8.55 (scale of 0–80). Bivariate correlations showed that parents’ chronic pain was not related to parent PTSS (r = .249, *p *= .371) nor was parent PTSS related to parents’ pain frequency (r = .190, *p *= .480), severity (r = .344, *p *= .301), or interference (r = .230, *p *= .410). Parent PTSS was related to parent catastrophizing about their child’s pain (r = .813, *p *< .001); greater PTSS was related to greater catastrophizing.

**Discussion/Conclusions:** The relationship between parent pain, PTSS, and how this might influence children’s pain experiences requires further elucidation. Future research should also investigate trajectories of pain and PTSS beginning at child’s diagnosis into survivorship.

### A Serious Immersive Virtual Reality Game for Promoting Chronic Arthritis Pain Patients’ General Physical Activity and Range of Motion

Xin Tong^a^, Diane Gromala^a^, Frederico Machuca^a^, Pam Squire^b^, Daehan Kim^c^, Yan Li^d^, Kunlin Wei^e^

^a^School of Interactive and Technology, Simon Fraser University, Vancouver, British Columbia, Canada; ^b^School of Medicine, University of British Columbia, Vancouver, British Columbia, Canada; ^c^University of Saskatchewan, Saskatchewan, Canada; ^d^Xuanwu Hospital, Capital Medical University, Beijing, China; ^e^School of Psychology, Peking University, Beijing, China

**CONTACT** Xin Tong tongxint@sfu.ca

© 2019 The Author(s). Published with license by Taylor & Francis Group, LLC.

This is an Open Access article distributed under the terms of the Creative Commons Attribution License (http://creativecommons.org/licenses/by/4.0/), which permits unrestricted use, distribution, and reproduction in any medium, provided the original work is properly cited.

**Introduction/Aim:** Virtual Reality (VR) shows great promise in creating testing and treatment environments where virtual representations can be precisely controlled and guided according to therapy needs. Therefore, the goal of this pilot study was to explore how arthritis pain patients like our VR environment and how effective it was at promoting physical activity (PA).

**Methods:** A mixed-method study was conducted. The inclusion criteria are arthritis patients who are older than 19 years old. Five participants with limitations of physical movement were recruited via convenient sampling, including 3 females (M = 61.5 years old, SD = 11.01). First, the participants were given a tutorial and then they explored the VR game for 15– 20 mins. The participants’ real-time Heart Rate (HR) was measured, and they were asked to fill in the Rating of Perceived Exertion Scale questionnaire.

**Results:** We found there was an increase in HR variation as participant’s age increases. Patients reported perceived physical exertion (M = 75.78, SD = 8.45) is lower than their real exertion (M = 82.25, SD = 7.05). This indicated that our VR game was able to immerse and distract the patients from the amount of PA without noticing they were in an aerobic state. Besides, all participants had their average HR above or close to their 50% threshold.

**Discussion/Conclusions:** Results presented a great promise of using our VR game to promote patients’ movement and enlarge their RoM. Our future work includes a longitudinal study to measure the effect on chronic pain patients’ PA level and pain.

### Investigating Canadian Parents’ Knowledge and Use of Evidence-Based Pain Management Strategies for Infants: An Analysis of Social Media Posts

Lindsay L. Richter^a^^b^, Christine T. Chambers 0000-0002-7138-916X^a^^c^, Erica Ehm^d^, Justine Dol^a^, Jennifer A. Parker^a^

^a^Centre for Pediatric Pain Research, IWK Health Centre, Halifax, Nova Scotia, Canada; ^b^Department of Obstetrics and Gynecology, University of British Columbia, Vancouver, British Columbia, Canada; ^c^Departments of Pediatrics & Psychology and Neuroscience, Dalhousie University, Halifax, Nova Scotia, Canada; ^d^YummyMummyClub.ca/YMC, Ehm & Co., Toronto, Ontario, Canada

**CONTACT** Lindsay Richter lrichter@bcchr.ca

© 2019 The Author(s). Published with license by Taylor & Francis Group, LLC.

This is an Open Access article distributed under the terms of the Creative Commons Attribution License (http://creativecommons.org/licenses/by/4.0/), which permits unrestricted use, distribution, and reproduction in any medium, provided the original work is properly cited.

**Introduction/Aim**: Breastfeeding, skin-to-skin care, and oral sucrose are extensively studied interventions for effectively managing procedural pain in newborns. We aimed to assess Canadian parents’ knowledge and use of these strategies.

**Methods**: In partnership with an award-winning Canadian digital publisher (YummyMummyClub.ca/YMC), we shared an evidence-based YouTube video about breastfeeding, skin-to-skin contact, and sucrose via Facebook as part of the #ItDoesntHaveToHurt initiative. Participants were prompted with the following questions: 1) have you ever used any of these strategies before, and 2) which new strategy did you learn that you’ll use in the future. Responses were summed and categorized for analysis.

**Results**: Within 24 hours, the post received 136 engagements (50 likes, 25 shares, and 61 comments). Of commenting parents, 25% had never used the strategies, 75% used at least one, and 27% used more than one. Breastfeeding (64%) and skin-to-skin contact (52%) were most common, while few respondents used sucrose in previous pain management (21%). 30% of parents expressed that they learned new information and that they will share or try at least one of the strategies. Sucrose generated the most interest (89%), followed by breastfeeding (44%), and skin-to-skin (39%). Parents commented on their experiences with pain management education from health professionals, alternative strategies for management of their infant’s pain, and questions regarding clinical use of sucrose for procedural pain.

**Discussion/Conclusions**: Parents’ knowledge of evidence-based pain management strategies for infants was enhanced through social media engagement. Health professionals should utilize science-media partnerships to reach parents and benefit parental involvement in pediatric pain management.

### Contribution of Acute Post-Operative Pain Towards Neuropathic Pain after Breast Cancer Surgery- 3-Month Prospective Cohort Study

N. Arora^a^, M. Gornitsky^b^, R. Hovey^c^, D. Hickey^d^, M. Basik^e^, F. Boileau^e^, H. Sigman^e^, D. Sinziana^e^, A.M. Velly^b^

^a^Department of Dentistry, McGill University, Montreal, Quebec, Canada; ^b^Department of Dentistry, Jewish General Hospital, McGill University, Montreal, Quebec, Canada; ^c^Faculty of Dentistry, McGill University, Montreal, Quebec, Canada; ^d^Department of Anesthesia, Jewish General Hospital, McGill University, Montreal, Quebec, Canada; ^e^Department of Surgery, Jewish General Hospital, McGill University, Montreal, Quebec, Canada

**CONTACT** Navpreet Arora navpreet.arora@mail.mcgill.ca; Ana Miriam Velly ana.velly@mcgill.ca

© 2019 The Author(s). Published with license by Taylor & Francis Group, LLC.

This is an Open Access article distributed under the terms of the Creative Commons Attribution License (http://creativecommons.org/licenses/by/4.0/), which permits unrestricted use, distribution, and reproduction in any medium, provided the original work is properly cited.

**Introduction/Aim:** The aim of this study is to determine the contribution of acute post-operative pain towards the development of neuropathic pain (NP) 3 months after breast cancer surgery (BCS).

**Methods:** In this 3-month prospective cohort study, female breast cancer patients (≥18 years) who underwent first BCS were recruited from Segal Cancer Center, Jewish General Hospital, Montreal. The study outcome was NP occurrence at 3 months post-surgery.

Collected data was acute post-operative pain, anxiety, depression, type of surgery, axillary status, and NP. Presence of NP was assessed at 3 months by telephone using the Douleur Neuropathique-4 (DN-4) questionnaire. Linear and logistic regression analysis were used to assess risk factors for the outcome.

**Results:** At 3 months post-surgery, 45 patients (24%) reported NP. The most frequent DN4 terms describing NP were: burning (31%), electric shock (21%), itching (31%), numbness (24%), pin and needles (24%). NP at 3 months was positively associated with *current pain intensity* (β = 0.11, P = 0.003), and *pain during movement* (β = 0.59, P = 0.006), both assessed at 7 days after surgery. Furthermore, numbness was associated with *current pain intensity* (β = 0.03, P = 0.02), and *acute pain* (β = 0.13, P = 0.04), both assessed at 7 days after surgery.

Finally, *pins and needles* were associated with *pain during movement* at 7 days (β = 0.14, P 0.02), and *tingling* (β = 0.14, P = 0.008). This association was not confounded by emotional state (β = 0.15, P = 0.01).

**Discussion/Conclusions:** NP at 3 months is associated with acute post-operative pain and its intensity. *Pins and needles* is associated acute pain, whereas *numbness* is associated with acute pain and its intensity.

### Non-Dermatomal Sensory Deficit, a Prevalent but Missing Phenomenon, in Chronic Pain Patients Attending Chronic Pain Management Program in Norther Ontario

Hadi Shojaei^a^, Bryan MacLeod^b^, Mary Donaghy^c^, Stacey Gleeson^d^, Victoria Ewen^c^, Arman Shojaei^e^

^a^Northern Ontario School of Medicine, Thunder Bay, ON, Canada; ^b^NOSM, Thunder Bay, ON, Canada; ^c^Lakehead University, Thunder Bay, ON, Canada; ^d^Psychotherapist, SJCG, Thunder Bay, ON, Canada; ^e^Faculty of Science, York University, Thunder Bay, Ontario, Canada

**CONTACT** Hadi Shojaei shojaeih@tbh.net

© 2019 The Author(s). Published with license by Taylor & Francis Group, LLC.

This is an Open Access article distributed under the terms of the Creative Commons Attribution License (http://creativecommons.org/licenses/by/4.0/), which permits unrestricted use, distribution, and reproduction in any medium, provided the original work is properly cited.

About 25% of the population in Canada and at least 70 million people in North America are suffering from chronic non cancer pain (CNCP). Treating patients with CNCP has always been a major challenge. One of the important and useful findings in CNCP patients is Non-Dermatomal Sensory Deficit (NDSD), which is a functional deficit in contrast to structural sensory deficit that can be caused from a clear peripheral or central nervous system lesion. NDSD has shown to be associated with maladaptive neuroplasticity. The phenomenon has been described extensively since 2001 by Dr. Angella Mailis and her team at the university of Toronto in numerous publications. Study of 100 consecutive patients referred to CPMP by community physicians in 2018 showed NDSD in 23%. 78% of NDSD patients were suffering from severe and moderate Depression, 65% were suffering from moderate and severe anxiety, and 91% suffered from Insomnia.

**Introduction/Aim:** It has been shown that NDSD is often present in individuals experiencing both a physical trauma as well as a psychological trauma, thus requiring further assessments. In Southern Ontario, there have been studies examining patients’ demographics and pain characteristics of patients with NDSDs. However, such studies have not been conducted for patients in Northern Ontario. We have been focused on studying not only chronic pain patients’ demographics, inciting events, pain characteristics, psychological and physical, but also we have added questionnaires for evaluating depression, anxiety, and perceived injustice.

**Methods:** Retrospective cross-sectional study of 100 consecutive patients referred to CPMP by community physicians in 2018 showed 23% NDSD. Demographics, inciting event, sleep, fatigue as well as PHQ-9, GAD-7, and IEQ scores were collected and analyzed using charts review of the 23 patients with NDSDs.

**Results:** 23% of CNCP patients attending CPMP at SJCG had NDSD. 82% of them were female (F = 19), with mean = 47.73 and Range = 16–78 years old. 74% were referred for assessment of multisite, diffuse, or widespread body pain (17 cases). 22% (5 cases) reported Motor Vehicle Accident as the inciting event, 22% reported work-related injury, 13% reported sport-related injury, 13% reported a fall, 4% reported Head Trauma, 4% post-appendectomy infection, 4% sudden onset of low back, neck, ankle pain each. Based on PHQ-9 questionnaire, 35% of patients indicated severe Depression (8 cases), 26% moderately severe Depression, 17% moderate Depression. Regarding GAD-7, 43% of patients scored for severe Anxiety (10 cases), 22% moderate Anxiety (5 cases). Regarding the Injustice Experience Questionnaire, 48% of patients scored at high levels of perceived injustice (11 cases), and 22% scored at moderate levels of perceived injustice. 91% of patients complained of difficulty falling asleep, and 65% complained of constant Fatigue (15 cases).

**Discussion/Conclusions:** NDSDs are prevalent clinical phenomena in chronic pain patients attending CPMP in Northern Ontario. Vast majority of the chronic pain patients (82%) developed sudden onsets of pain following a clear inciting event. NDSDs are commonly associated with Insomnia, Fatigue, Anxiety, Depression, and High/Moderate Perceived Injustice.

### Rasch Analysis of the Patient-Rated Wrist Evaluation Questionnaire

S Esakki^a^, Jc MacDermid^a^^b^^c^, Ji Vincent^a^, Tl Packham^c^, D Walton^a^, R Grewal^b^^d^

^a^School of Physical Therapy, Western University, London, ON, Canada; ^b^The Hand and Upper Limb Centre, St Joseph’s Health Centre, London, ON, Canada; ^c^School of Rehabilitation Science, McMaster University, Hamilton, ON, Canada; ^d^Department of Surgery, University of Western Ontario, London, ON, Canada

**CONTACT** Saravanan Esakki sesakki@uwo.ca

© 2019 The Author(s). Published with license by Taylor & Francis Group, LLC.

This is an Open Access article distributed under the terms of the Creative Commons Attribution License (http://creativecommons.org/licenses/by/4.0/), which permits unrestricted use, distribution, and reproduction in any medium, provided the original work is properly cited.

**Introduction/Aim**: The Patient-Rated Wrist Evaluation (PRWE) was developed as a wrist joint specific measure of pain and disability. Rasch analysis (RA) has been endorsed as a newer method for analyzing the clinical measurement properties of self-report outcome measures.

The purpose of this study was to evaluate the PRWE using Rasch modeling.

**Methods**: We employed the Rasch model to assess overall fit, response scaling, individual item fit, differential item functioning (DIF), local dependency, unidimensionality, and person separation index (PSI). A convenience sample of 382 patients with distal radius fracture was recruited from the hand and upper limb clinic at a large academic healthcare organization, London, Ontario. RA was conducted on the 3 subscales (pain, specific activities, and usual activities) of the PRWE separately.

**Results**: The pain subscale adequately fit the Rasch model when item 4 was deleted to eliminate non-uniform DIF by age group, and item 5 was rescored by collapsing into 8 intervals to eliminate disordered thresholds. After background rescoring of 2 items in pain subscale, 2 items in specific activities and 3 items in usual activities, all three subscales of the PRWE were well targeted and had high reliability (PSI = 0.86). These changes provided a unidimensional, interval-level scaled measure.

**Discussion/Conclusions**: Like a previous analysis of the Patient-Rated Wrist and Hand Evaluation, this study found the PRWE could be fit to the Rasch model with rescoring of multiple items. However, the modifications required to achieve fit were not the same across studies, our fit statistics also suggested one of the pain items should be deleted.

### Characterization of Key Sexually Dimorphic Regulators in Pain Processing

Shahrzad Ghazisaeidi^a^, Arun Ramani^b^, Parisa Shooshtari^b^, Amy Tu^c^, Katherine Halievski^c^, David Finn^d^, Sofia Assi^a^, Milind Muley^c^, Vivian Wang^c^, Ameet Sengar^c^, Rosanna Weksberg^e^, Michael Brudno 0000-0001-7947-2243^b^, Michael W Salter^c^

^a^Physiology, University of Toronto, Toronto, Ontario, Canada; ^b^Centre for Computational Medicine, The Hospital for Sick Children, Toronto, Ontario, Canada; ^c^Neurosciences and Mental Health, The Hospital for Sick Children, Toronto, Ontario, Canada; ^d^Pharmacology & Therapeutics, National University of Ireland, Galway, Ireland; ^e^Genetics & Genome Biology, The Hospital for Sick Children, Toronto, Ontario, Canada

**CONTACT** Shahrzad Ghazisaeidi shahrzad.ghazisaeidi@mail.utoronto.ca

© 2019 The Author(s). Published with license by Taylor & Francis Group, LLC.

This is an Open Access article distributed under the terms of the Creative Commons Attribution License (http://creativecommons.org/licenses/by/4.0/), which permits unrestricted use, distribution, and reproduction in any medium, provided the original work is properly cited.

**Introduction/Aim**: Chronic pain affects 1 in 5 Canadians and costs over $43B annually, yet effective and safe treatment options remain elusive. Recent discoveries have brought to the forefront sex differences in mechanisms of pain as a potential explanation why novel pre-clinical therapeutics have not translated into successfully in clinical trials.

**Methods**: To begin understanding how males and females differ in pain processing, we analyzed gene expression, using RNA sequencing, and DNA methylation, using reduced representation bisulfite sequencing (RRBS), in rodent models of neuropathic pain.

**Results**: Across both sexes, our data reveals peripheral nerve injury (PNI) caused upregulation of 61 genes involved in innate immune responses in spinal cord. In females specifically, we observed PNI-induced downregulation of 5 genes involved in neuronal function and upregulation of two classes of Cathepsins. (C and E). On the other hand, in males, we observed upregulation of 14 genes including those involved in metabolism of purines and glutathione. Additionally we found that PNI leads to methylome remodeling in a sexually dimorphic manner: 125 promoters in rat spinal cord that were differentially methylated in injured males versus females.

**Discussion/Conclusions**: Our data shows robust sex specific DNA methylation and transcriptome signature after PNI. Additionally, our findings leads to the hypothesis that remapping of DNA methylation, with subsequent alterations in the transcriptome, are critically involved in the development of neuropathic pain. We anticipate that future research directed at understanding these differences may lead to effective drug development to combat chronic pain.

### eHealth Interventions for Improving Evidence-Based Pain Practices among Healthcare Professionals: A Scoping Review

Shelly-Anne Li^a^, Suman Virdee^b^, Mariana Bueno^c^, Bonnie Stevens^d^

^a^Lawrence S Bloomberg Faculty of Nursing, University of Toronto, Toronto, ON, Canada; ^b^Global Health Program, McMaster University, Hamilton, ON, Canada; ^c^Child Health Evaluative Sciences, The Hospital for Sick Children, Toronto, ON, Canada; ^d^Lawrence S Bloomberg Faculty of Nursing, University of Toronto; Research Institute, The Hospital for Sick Children, Toronto, ON, Canada

**CONTACT** Shelly-Anne Li shellyanne.li@mail.utoronto.ca

© 2019 The Author(s). Published with license by Taylor & Francis Group, LLC.

This is an Open Access article distributed under the terms of the Creative Commons Attribution License (http://creativecommons.org/licenses/by/4.0/), which permits unrestricted use, distribution, and reproduction in any medium, provided the original work is properly cited.

**Introduction/Aim**: eHealth interventions provide a promising approach to promote the use of evidence-based pain practices among healthcare professionals (HCPs). The objectives of this scoping review were to (1) identify scientific peer-reviewed and publicly available eHealth interventions (including electronic toolkits, web-based resources) that aim to improve pain assessment and treatment practices, and (2) evaluate the validity, effectiveness and comprehensiveness of these eHealth interventions.

**Methods**: MEDLINE, EMBASE, CINAHL, AMED, ERIC, PsycINFO, Cochrane, and Web of Science databases were searched from inception to May 2018 to uncover any peer-reviewed citations; Google engine was searched for publicly available eHealth interventions. Eligible eHealth interventions were targeted at HCPs and focused on an eHealth knowledge translation strategy (1) to support the integration of evidence into pain practice, and (2) to facilitate sharing of knowledge, building/promoting awareness and changing practice.

**Results**: Of 2979 retrieved citations, 22 (18 published reports, 4 conference abstracts) were included. Thirty-six publicly available eHealth interventions were identified. eHealth interventions aimed to improve pain across different healthcare practices including mental health, pediatrics, dental health, rehabilitation, primary care and neurology. Of the peer-reviewed citations, 75% of the eHealth interventions were not evaluated for effectiveness; of those that were, none reported on improving pain practice among HCPs, nor patient pain outcomes.

**Discussion/Conclusions**: Researchers should consider examining the relationship between use of eHealth interventions and whether HCP’s pain practices improve. Investigation on why published eHealth interventions do not tend to move beyond the research phases of usability and feasibility testing to evaluate intervention effectiveness, is warranted.

### Sex and Gender-Based Analysis in Studies of Thumb Osteoarthritis

Joy MacDermid

Western University, London, Ontario, Canada

**CONTACT** Joy MacDermid jmacderm@uwo.ca

© 2019 The Author(s). Published with license by Taylor & Francis Group, LLC.

This is an Open Access article distributed under the terms of the Creative Commons Attribution License (http://creativecommons.org/licenses/by/4.0/), which permits unrestricted use, distribution, and reproduction in any medium, provided the original work is properly cited.

**Introduction/Aim:** Arthritis of the hand, particularly the carpometacarpal (CMC) joint is one of the most sex/gender differentiated health conditions. Therefore, incorporating sex and gender-based analysis in research is critical. Further standards for sex and gender-based reporting are increasingly being mandated in research. This study evaluated the extent to which clinical trials relating to the CMC arthritis were consistent with the SAGER guidelines for sex/gender considerations in design and analysis.

**Methods:** A systematic search was conducted to identify randomized clinical trials on CMC arthritis (surgery or rehabilitation). The methods used were compared to SAGER guidelines to assess compliance with quality criteria for design/reporting in relation to sex/gender. The evaluated quality criteria addressed sex/gender definitions, sampling, analysis and interpretation

**Results:** Amongst the 21 clinical trials conducted on CMC arthritis, including surgery and rehabilitation interventions none adequately considered sex and gender in sampling, analysis or discussion of findings. Women form the majority of the sample in most studies, but studies typically included less than 100 people. None were explicit in how recruitment was designed to obtain adequate gender representation; stated how non-binary sex or gender were managed or specifically stated whether the results were equally valid for men and women.

**Discussion/Conclusions:** Many of the studies addressing CMC arthritis are small and too underpowered to address sex and gender as a disaggregated analysis. Nevertheless, dramatic improvements are needed in how sex and gender are considered in sampling, collection of sex and gender data, analysis and reporting.

**Disclosure statement**

No potential conflict of interest was reported by the author.

Reference1.
Heidari
S, Babor
TF, De Castro
P, Tort
S, Curno
M. Sex and gender equity in research: rationale for the SAGER guidelines and recommended use. Res Integr Peer Rev. 2016;1(1):2. doi:
10.1186/s41073-016-0007-6.29451543PMC5793986

### How Much Does Pain or Fear of Needles Contribute to Vaccine Hesitancy in Parents - a Systematic Review and Meta-Analysis

Jonathan Sgro^a^, Yixin Qu^a^, Anna Guo^a^, Jason Stacey^a^, Many Fung^a^, Derek Stephens^b^, Elizabeth Uleryk^c^, Anna Taddio^a^^b^

^a^Leslie Dan Faculty of Pharmacy, University of Toronto, Toronto, ON, Canada; ^b^Child Health Evaluative Sciences, The Hospital for Sick Children, Toronto, ON, Canada; ^c^Uleryk Consulting, Mississauga, ON, Canada

**CONTACT** Anna Taddio anna.taddio@utoronto.ca Leslie Dan Faculty of Pharmacy, University of Toronto, Toronto, Canada

© 2019 The Author(s). Published with license by Taylor & Francis Group, LLC.

This is an Open Access article distributed under the terms of the Creative Commons Attribution License (http://creativecommons.org/licenses/by/4.0/), which permits unrestricted use, distribution, and reproduction in any medium, provided the original work is properly cited.

**Background and Objectives**: Pain or fear associated with needles is a cited barrier to vaccination. There has been no quantitative synthesis of the research evidence regarding the impact of pain or fear of needles on vaccination compliance. Our objective was to determine the prevalence of pain or fear as a parent-reported barrier to vaccination of children.

**Methods/Description**: A literature search was conducted for articles published January 1946 to July 2017. The search strategy was developed and executed with the assistance of an academic librarian using relevant medical subject headings (MeSH terms) and free-text terms. We included studies that: i) addressed vaccine hesitancy and attitudes towards needles, ii) identified vaccinated and partially vaccinated/non-vaccinated groups, iii) identified reasons for partial and non-vaccination (barriers), and iv) inquired specifically about pain or fear of needles and reported information to enable determination of the proportion of parents that selected this response. Data was extracted, and meta-analysis was carried out.

**Results**: The overall prevalence of pain or fear as a barrier to vaccination was 21.25% (95% CI 11.35 to 33.25) for surveys using closed-ended questions to elicit reasons for vaccine non-compliance and 2.60% (95% CI 0.58 to 6.00) for those using open-ended questions.

**Conclusion/Implications**: Pain and fear contribute to parents’ decisions to not vaccinate their children. We recommend interventions be utilized more widely to reduce pain and fear during vaccinations in children. Limitations include a small number of included studies and lack of consistency with regards to definitions and procedures.

### Lost for Primary Care. The Lessons from the TRAST Project

Irina Kudrina^a^, Isabelle Vedel^a^, Perry Adler^a^, Joyce Chen^a^, Vanessa Pasztor^a^, Michael Soueidan^a^, Brian Bradley^b^, Krista Brecht^b^, Louisette Larochelle^b^, Sabrina Morin Chabane^b^^c^, Gillian Bartlett^a^, Gabrielle Pagé^b^^d^, Manon Choinière 0000-0001-9593-8883^d^, Yoram Shir^b^

^a^Family Medicine Department, McGill University, Montreal, Quebec, Canada; ^b^Anesthesia Department, McGill University Health Center, Montreal, Quebec, Canada; ^c^Physiotherapy Department, McGill University Health Center, Montreal, Quebec, Canada; ^d^Psychologie, Centre hospitalier de l’Université de Montréal, Montréal, Québec, Canada

**CONTACT** Irina Kundrina irina.kudrina@mcgill.ca

© 2019 The Author(s). Published with license by Taylor & Francis Group, LLC.

This is an Open Access article distributed under the terms of the Creative Commons Attribution License (http://creativecommons.org/licenses/by/4.0/), which permits unrestricted use, distribution, and reproduction in any medium, provided the original work is properly cited.

**Introduction/Aim**: ~17% of young Quebecers aged 18–35 suffer from a chronic non-cancer pain (CNCP) condition, ~12–17% of sufferers report no pain relief. Transition of youth to adult care without primary care continuing guidance, extinct connection to children services, and new adulthood responsibilities could lead to missed medical appointments, poor pain management, self-medication practices, and increased risk of psychosocial co-morbidities. Primary care practitioners (PCPs), following patients from the cradle to the grave and supported by specialized pain services, represent a safety net for this population. *Objectives*. 1. To evaluate existing McGill University pain services communication practices, patients’ and PCPs’ roles in pain management, as viewed by the young patients, their caregivers, and referring PCPs. 2. To offer a formative feedback to the primary and specialized care teams and propose realistic mitigation strategies.

**Methods**: Participatory design. Three-phase sequential–consensual qualitative: 1. Consultations with patient-experts. 2. Individual semi-structured interviews with PCPs. 3. Stakeholder focus groups (care providers, administration, allied professionals, patient-partners). *Participants*. McGill (AEPMU) and University of Montreal pain team members, consulting PCPs, patients. *Analysis*. Thematic deductive-inductive analysis.

**Results**: 1. PCPs referring to AEPMU lack training in adolescent/youth, pain and addiction medicine. 2. PCPs are seldom involved in interdisciplinary pain management. 3. Pain in youth is highly stigmatized. 4. Young pain patients are viewed as too complex, vulnerable, and not fitting PCP practice profiles. 5. Overwhelming lack of resources, insufficient communication prevent appropriate pain care provision.

**Discussion/Conclusions**: Major gaps in the management of CNCP in young adults exist. Realistic mitigations strategies were proposed.

### Phenotyping Chronic Musculoskeletal by Pain Catastrophizing and Pressure Pain Threshold Level and Comparing on Constructs of the Fear-Avoidance Model of Pain

Zakir Uddin^a^, Arthur Woznowski-Vu^a^, Daniel Flegg^a^, Andrea Aternali^a^, Timothy H. Wideman^a^

School of Physical and Occupational Therapy, McGill University, Montreal, Quebec, Canada

**CONTACT** Zakir Uddin zakir.uddin@mail.mcgill.ca

© 2019 The Author(s). Published with license by Taylor & Francis Group, LLC.

This is an Open Access article distributed under the terms of the Creative Commons Attribution License (http://creativecommons.org/licenses/by/4.0/), which permits unrestricted use, distribution, and reproduction in any medium, provided the original work is properly cited.

**Introduction/Aim:** Pain catastrophizing scale (PCS) and pressure pain threshold (PPT) are two evidence-based outcome measures for both research and clinical practice. The relationship between PCS and PPT is not clear with existing controversy and insufficient studies. The aim of this study is to evaluate whether subgroups of chronic musculoskeletal pain patients defined by the relative congruence of PCS and PPT differed on the major constructs (predictor and outcomes) of the fear-avoidance model (FAM) after controlling for covariates.

**Methods:** A total of 125 participants (68% women; mean ± SD age 53.5 ± 12.77 years) with chronic musculoskeletal pain participated in the study. Of these individuals who met the inclusion criteria, 103 participants completed all measures included in this study. Participants completed a single testing session that included measures of all of the major constructs of the FAM, including pain catastrophizing, pain-related fear, activity avoidance (self-report and functional measures), pain-related disability, depression and pain severity, as well as pressure pain threshold. Subgroups were constructed by dichotomizing (median split) of PCS and PPT scores, resulting in 4 groups: high-PCS/high-PPT (n = 26), high-PCS/low-PPT (n = 29), low-PCS/high-PPT (n = 24), and low-PCS/low-PPT (n = 24).

**Results:** Onaway ANOVA revealed significant group differences (p < .05) on all the FAM constructs of this study (except functional avoidance) and post hoc analysis indicated the most of the significance differences are for the high-PCS/low-PPT group (p < .05). Multivariate analyses also revealed significant group differences (p < .01) after adjusting for differences on all covariates (age, ethnicity, education level, comorbidity, BMI), except gender.

**Discussion/Conclusions:** The study suggest higher level of pain catastrophizing and lower level of pressure pain threshold are an indicator of higher level FAM constructs in predicting pain-related fear and outcomes considering gender and functional avoidance. This finding has important clinical and theoretical implications.

### Medical Cannabis-Opioid Reduction Program (MCORP): Results from 6-Month Longitudinal Multi-Disciplinary Pain Program

Kevin Rod

Department of Community and Family Medicine, University of Toronto, Toronto, ON, Canada; Toronto Poly Clinic, Toronto, ON, Canada

**CONTACT** Kevin Rod krod@tpclinic.com

© 2019 The Author(s). Published with license by Taylor & Francis Group, LLC.

This is an Open Access article distributed under the terms of the Creative Commons Attribution License (http://creativecommons.org/licenses/by/4.0/), which permits unrestricted use, distribution, and reproduction in any medium, provided the original work is properly cited.

**Background**: Patients referred to Toronto Poly Clinic multi-disciplinary pain program are often receiving opioid therapy at high-doses (≥90 mg Morphine Equivalent Dose [MED]) than recommended by Canadian opioid prescribing guidelines. These patients are encouraged to gradually taper the dose to avoid risks associated with opioid therapy. Tapering is successful when the patient is carefully monitored and provided with appropriate pharmacological and psychological support. In this study, we observed the effect of medical cannabis and psychological support on opioid reduction and pain management.

**Methods**: Patients with chronic pain (N = 600) receiving high-dose opioids (range: 90–240 MED/daily) were included in this program. The rate of tapering opioid dose was individualised per patient (average reduction: 10% every 1–2 weeks). For each reduction, patients were authorized to increase medical cannabis dose by 0.5 g/day up to a maximum of 3 g/day, if needed. ZENDOSE, a validated web-based mental health and wellness tool, was used to track physical, and mental well-being of patient. Physicians monitored patients regularly, assessing their pain, sleep, function, and quality of life (QoL), every 1–2 weeks.

**Results**: After six months, 329 (55%) patients were able to reduce their daily opioid intake by 30%; 156 (26%) patients discontinued opioids. All patients expressed satisfaction with their pain control, sleep and QoL. Opioid dose remained unchanged and increased in 114 (19%) and 1 patient(s), respectively. Detailed results will be presented.

**Conclusion**: Results from this study warrants further investigation on medical cannabis as a potential alternative to prescription opioids for treating chronic pain and opioid-dose reduction

### Arthroscopic versus Mini-Open Rotator Cuff Repair: A Randomized Trial and Meta-Analysis

Joy MacDermid^a^, Diane Bryant^b^, Richard Holtby^c^, Helen Razmjou and JOINTS Canada^d^

^a^Physical Therapy, Western University, London, Ontario, Canada; ^b^Departments of Surgery,and Physical Therapy, University of Western Ontario, London, Ontario, Canada; ^c^FRCSC Department of Orthopaedic Surgery, Holland Orthopaedic & Arthritic Centre, Toronto, Canada; ^d^Department of Rehabilitation, Sunnybrook Research Institute

**CONTACT** Joy MacDermid jmacderm@uwo.ca

© 2019 The Author(s). Published with license by Taylor & Francis Group, LLC.

This is an Open Access article distributed under the terms of the Creative Commons Attribution License (http://creativecommons.org/licenses/by/4.0/), which permits unrestricted use, distribution, and reproduction in any medium, provided the original work is properly cited.

**Background:** This randomized trial compared mini-open (MO) versus all-arthroscopic (AA) rotator cuff repair.

**Methods:** People with rotator cuff tears were screened and consented to randomization to MO or AA repair at 9 centres by 23 surgeons. Random allocation was revealed in the operating room. The primary outcome – The Western Ontario Rotator Cuff Index (WORC); secondary outcomes: ASES, SPADI pain scale, SF-12, MRI imaging of cuff integrity, range of motion, strength, medication use and adverse events were assessed by a blinded evaluator 1-month before surgery; and 2 and 6-weeks, and 3, 6, 12, 18, and 24 months later. A single blinded radiologist evaluated MRIs at baseline and 2-years. Intention-to-treat ANCOVA with preoperative WORC score, age and tear size as covariates, assessed continuous outcomes; disaggregated by sex. A meta-analysis synthesized the current trial outcomes with prior studies.

**Results:** From 954 patients screened, 474 were eligible and consented, 449 were screened; 138 received MO and 134 arthroscopic AA repair. The groups (AA/MO) were similar prior to surgery on age, sex, WORC scores, BMI, and third party compensation. WORC scores improved from 40 pre-op to 89 (AA) and 93 (MO) at 2-years; for effect sizes of 2.8 and 3.0. No significantly differences(NS) occurred between AA and MO at any time-point. All secondary patient report measures were NS between groups, except the 2-year SPADI pain (MO = 12 vs AA = 8; p = 0.02). Similar and significant recovery in motion and strength occurred in both groups over time (pre-op/2-years) in ROM and strength. MRI indicated minimal improvement in muscle relative to fat (AA = 3; MO = 2), with most worsening (AA = 25; MO = 24) or remaining unchanged (AA = 70; MO = 70). Opioid and NSAID use significantly reduced after surgery. The meta-analysis indicated NS standardized mean group differences on the primary outcome across all pooled studies (SMD −0.06, 95%CI −0.34 to 0.22).

**Discussion/Conclusions:** Both AA and MO rotator cuff repair provide large clinical benefits, with few adverse events.

### Thematic Synthesis of Parental Participation for Procedural Pain Management in the NICU

Carol McNair^a^, Nevart Chinian^b^, Vibhuti Shah^b^, Mary McAllister^c^, Linda S. Franck^d^, Bonnie Stevens^e^, Lisa Burry^f^, Anna Taddio^f^

^a^Neonatology, SickKids, Toronto, Ontario, Canada; ^b^Neonatology, Mount Sinai Health System, Toronto, Ontario, Canada; ^c^Department of Nursing, SickKids, Toronto, Ontario, Canada; ^d^Family Health Care Nursing, University of California San Francisco, Francisco, CA, USA; ^e^Research Institute, SickKids, Toronto, Ontario, Canada; ^f^Leslie Dan Faculty of Pharmacy, University of Toronto, Toronto, Ontario, Canada

**CONTACT** Carol McNair carol.mcnair@sickkids.ca

© 2019 The Author(s). Published with license by Taylor & Francis Group, LLC.

This is an Open Access article distributed under the terms of the Creative Commons Attribution License (http://creativecommons.org/licenses/by/4.0/), which permits unrestricted use, distribution, and reproduction in any medium, provided the original work is properly cited.

**Introduction/Aim:** Emerging data from qualitative studies demonstrates that parents wish to be involved in all aspects of their infant’s care in the NICU, including pain management. The aim of this study was to complete a meta-synthesis evaluating the factors affecting parent participation in pain management.

**Methods:** A literature search was conducted of studies published from 1976 to 2017 using MeSH terminology. All studies that utilized a qualitative methodology and evaluated parental participation and education in NICU were included. The Critical Appraisal Skills Programme (CASP) qualitative tool was used to assess the qualitative studies by two researchers. A thematic synthesis technique was used to collate the qualitative data.

**Results:** A total of 29,903 articles were returned. Once duplicates were removed, and the studies were limited to qualitative methodology, 47 studies remained. Forty studies were excluded for not being conducted with neonates, were evaluating comfort measures in neonatal palliative care, or were review/opinion articles. This left seven studies applicable for inclusion.

Four themes were identified from the seven studies reviewed. How to parent, parental stress and anxiety, health care providers as gate keepers and NICU environment were identified as the key themes across the studies.

**Discussion/Conclusions:** Four main themes were identified in this qualitative meta-synthesis that impact parents ability to participate in their infant’s pain management. Further research to evaluate how to improve parental participation in pain management in the NICU is needed to better understand this phenomenon.

### Characteristics of Canadians Likely to Try or Increase Cannabis Use following Legalization for Recreational Use

Harman S. Sandhu^a^, Jason W. Busse 0000-0002-0178-8712^b^

^a^Department of Health Research Methods, Evidence and Impact, McMaster University, Hamilton, Ontario, Canada; ^b^Department of Anesthesia, McMaster University, Hamilton, Ontario, Canada

**CONTACT** Harman.S Sandhu sandhhs3@mcmaster.ca

© 2019 The Author(s). Published with license by Taylor & Francis Group, LLC.

This is an Open Access article distributed under the terms of the Creative Commons Attribution-NonCommercial License (http://creativecommons.org/licenses/by-nc/4.0/), which permits unrestricted non-commercial use, distribution, and reproduction in any medium, provided the original work is properly cited.

**Introduction/Aim:** On October 17 2018, the Government of Canada legalized the non-medical use of cannabis. Effects of this policy on rates of use remain uncertain.

**Methods:** We used data from the National Cannabis Survey, Wave 1 (collected from February to March 2018) to explore the rate of, and characteristics associated with, initiating or increasing cannabis use due to legalization among the Canadian population aged 15 years and over. We applied bootstrap weights provided by Statistics Canada to extrapolate findings to the entire Canadian population. We constructed logistic regression models to calculate adjusted odds ratios (aORs) for predictors of new or increased use, and present adjusted risk increases (ARIs) for all significant associations.

**Results:** Our findings suggest that 21% of Canadians may initiate or increase cannabis consumption after legalization. Canadians who were aged 15-35 years (aOR: 4.4; ARI: 22.4%), had used cannabis in the past 3 months (aOR: 2.9; ARI: 19.4%), had higher income (aOR: 1.05; ARI: 6.5%), and reported poorer mental health (aOR: 1.7; ARI: 9.0%) were more likely to intend on trying or increasing their cannabis use compared to referent categories.

**Discussion/Conclusions:** Cannabis use may increase following legalization for recreational use in Canada. Potential higher risk populations (adolescents and young adults, those with mental illness) should be carefully monitored post-cannabis legalization, and targeted for public health education.

### Exploration of Body Ownership, Agency and Heat Pain Perception in Virtual Reality (VR)

Xin Tong^a^, Henan Diao^b^, Kunlin Wei^b^, Li Hu^c^, Diane Gromala^a^

^a^School of Interactive and Technology, Simon Fraser University, Vancouver, British Columbia, Canada; ^b^School of Psychology, Peking University, Beijing, China; ^c^Chinese Academy of Sciences, Institute of Psychology, Beijing, China

**CONTACT** Xin Tong tongxint@sfu.ca

© 2019 The Author(s). Published with license by Taylor & Francis Group, LLC.

This is an Open Access article distributed under the terms of the Creative Commons Attribution License (http://creativecommons.org/licenses/by/4.0/), which permits unrestricted use, distribution, and reproduction in any medium, provided the original work is properly cited.

**Introduction/Aim**: Prior studies have shown that the representation and movement of a body in VR can increase heat pain thresholds. For instance, a reddened arm significantly decreased the pain threshold compared with normal/blue skin color. Seeing one’s arm alone in VR has significant analgesic affect and increase heat pain thresholds. We hypothesize comparing to a still hand in VR, synced arm movement can increase pain thresholds. This top-down modulation of pain through visual and motor input may suggest a potential use of altering embodied virtual body image for pain therapy and management.

**Methods**: Ten subjects (8 females, 19 – 29, mean = 25.2, SD = 5.4) were recruited. All participants went through the same three visual conditions and they were asked to perform the same movement task (1) no virtual body (2) a virtual body with right arm still, and (3) a virtual body with synced right arm movement. Heat pain and self-reported sense of ownership and sense of agency were measured in VAS. The heat pain stimulus was generated by a TSA-II Neuro Sensory Analyzer.

**Results**: Comparing the still arm condition with synced arm group, significant differences (p < 0.01) were found in question (I felt the virtual arm was my own arm) and question (I felt my real arm was becoming virtual). The conditions with hands in VR had significant higher pain thresholds than the no-hand condition (p < 0.05). Nonetheless, we did not find significant differences in pain thresholds between the still and synced movement conditions.

**Discussion/Conclusions**: We will continue the study with another 10–15 participants as 25 is a standard amount for pain thresholds investigation studies which will be done by Dec 2018.

### Urinary Volatiles from Pregnant Mice Produce Stress-Induced Analgesia in Male Mice

Lucas V. Lima^a^, Sarah Rosen^b^, Janet Zhao^a^, Susana G. Sotocinal^a^, Jeffrey S. Mogil^a^

^a^Alan Edwards Centre for Research on Pain, McGill University, Montreal, Quebec, Canada; ^b^Washington University, St. Louis, USA

**CONTACT** Lucas Lima lima.lvl@outlook.com

© 2019 The Author(s). Published with license by Taylor & Francis Group, LLC.

This is an Open Access article distributed under the terms of the Creative Commons Attribution License (http://creativecommons.org/licenses/by/4.0/), which permits unrestricted use, distribution, and reproduction in any medium, provided the original work is properly cited.

**Introduction/Aim**: We observed while performing behavioral experiments on pregnant mice that male mice in the same testing room had elevated pain thresholds. The male mice also displayed more aggressive behaviors (e.g., biting an experimenter during handling). Based on this observation we hypothesized that male mice display stress-induced analgesia in the presence of pregnant mice.

**Methods**: We tested whether exposure to female mice in different stages of their reproductive cycle (naïve, early pregnancy, late pregnancy, lactating and post-weaning) would affect thermal pain thresholds and systemic corticosterone levels in male mice. Further, we exposed male mice to different volumes of urinary volatiles known to be increased during pregnancy in mice (pentyl acetate and 4-heptanone) and compared the pain thresholds and corticosterone levels to a control group. Aggression levels were tested by using the Resident-Intruder paradigm test in mice exposed to pentyl acetate.

**Results**: Male mice exposed to both late pregnant and lactating mice showed increased pain thresholds, while increased levels of systemic corticosterone were only observed in males exposed to late pregnant mice. Exposure to pentyl acetate and 4-heptanone induced a dose-dependent reduction of pain threshold. Resident-intruder test revealed a trend for pentyl-acetate exposed mice to be more aggressive than control (reduced attack latency).

**Discussion/Conclusions**: We show for the first-time evidence for a chemical signal, released from pregnant and lactating mice, which may act as a stressor for male mice and affect their pain behaviors.

### Canadian Surveillance of Complex Regional Pain Syndrome in Children and Youth – Results from Year 1 of Surveillance

Krista Baerg 0000-0001-5192-7285^a^, Susan Tupper 0000-0003-3736-357X^b^, G. Allen Finley 0000-0003-4579-7749^c^

^a^Department of Pediatrics, University of Saskatchewan, Saskatoon, Saskatchewan, Canada; ^b^School of Rehabilitation Science, University of Saskatchewan, Saskatoon, Saskatchewan, Canada; ^c^Department of Anesthesia, Pain Management & Perioperative Medicine, Dalhousie University, Halifax, Nova Scotia, Canada

**CONTACT** Krista Baerg dr.kbaerg@usask.ca

© 2019 The Author(s). Published with license by Taylor & Francis Group, LLC.

This is an Open Access article distributed under the terms of the Creative Commons Attribution-NonCommercial License (http://creativecommons.org/licenses/by-nc/4.0/), which permits unrestricted non-commercial use, distribution, and reproduction in any medium, provided the original work is properly cited.

**Introduction**: Complex Regional Pain Syndrome (CRPS), previously *regional sympathetic dystrophy*, is a chronic severe pain condition characterized by continuing pain disproportionate in time or degree to the usual course of known trauma or lesion. The pain is regional and has a distal predominance of abnormal sensory, motor, sudomotor, vasomotor, and/or trophic findings. In adults, annual incidence is 5.5–26.2 new cases per 100,000 annually. Pediatric incidence is unknown and diagnostic criteria have not been validated.

**Methods**: The Canadian Paediatric Surveillance Program (CPSP) surveys approximately 2,700 pediatricians and pediatric subspecialists monthly. CPSP enables detection of CRPS cases that present in pediatric primary care, subspecialty care, and inpatient care. For the 2-year study period, Canadian pediatric pain clinics are included. Participating physicians signal cases to the CPSP then complete a follow-up questionnaire. *Case definition*: Any patient aged 2–18 years with a new diagnosis of CRPS, meeting the International Association for the Study of Pain clinical diagnostic criteria.

**Results**: During year 1, 87 potential cases have been identified, 38 detailed surveys were received and 29 cases confirmed. Confirmed cases range from 7–16 years; 76% are female (22/29) and 66% report severe pain (19/29). Most commonly, one lower limb is affected (69%, 20/29) and the inciting event is trauma/injury (55%, 16/29). Of 15 cases that missed school, 60% missed less than 2 weeks (9/15).

**Conclusions**: Study results will help determine the minimum incidence of CRPS, patient demographics, and risk factors in Canada. Results will be used to promote early recognition and treatment to benefit patient recovery.

#### Acknowledgments

Collaborating Canadian Pediatric Pain clinics (BC Children’s Complex Pain Service, Alberta Children’s Hospital, Stollery Children’s Hospital, Saskatchewan Health Authority – Saskatoon, Children’s Hospital of Eastern Ontario, London Health Sciences Centre, The Hospital for Sick Children, McMaster Children’s Hospital, CHU Ste-­Justine, McGill University Health Centre, IWK Health Centre), the Canadian Paediatric Surveillance Program (CPSP) and CPSP participants.

### Nonpharmacological Self-Management of Migraine across Social Locations in the Southeast United States: An Equity-Oriented, Qualitative Analysis

Deanna Befus^a^, Sharon Hull^b^, Justine Strand de Oliveira^c^, Gillian Sanders^d^, Morris Weinberger^e^, Remy Coeytaux^f^

^a^Arthur Labatt Family School of Nursing, Western University, London, Ontario, Canada; ^b^Community and Family Medicine, Duke University, Durham, North Carolina, USA; ^c^Physician Assistant Programme, Queen Mary University of London, London, UK; ^d^Department of Medicine, Duke University, Durham, North Carolina, USA; ^e^Health Policy and Management, University of North Carolina-Chapel Hill, Chapel Hill, North Carolina, USA; ^f^Wake Forest School of Medicine, Center for Integrative Medicine, Winston-Salem, North Carolina, USA

**CONTACT** Deanna Befus dbefus@uwo.ca

© 2019 The Author(s). Published with license by Taylor & Francis Group, LLC.

This is an Open Access article distributed under the terms of the Creative Commons Attribution License (http://creativecommons.org/licenses/by/4.0/), which permits unrestricted use, distribution, and reproduction in any medium, provided the original work is properly cited.

**Introduction/Aim**: Migraine – a disabling neurological pain disorder and the sixth biggest cause of disability worldwide – was declared a major public health problem by the WHO due to a paucity of knowledge about cause and effective treatment options. In incidence and severity, migraine disproportionately affects people occupying marginalized social locations (SL). Cost-prohibitive, ineffective, and unsustainable pharmacological treatment options have contributed to high levels of interest in complementary approaches by people with migraine. Little is known about their motivations, patterns of use or access, or whether these vary by SL.

**Methods**: Thematic qualitative content analysis of focus groups (n = 30) exploring most meaningful outcomes and goals in migraine treatment.

**Results**: Four themes: a more holistic, collaborative, long-term treatment approach; medication as a short-term solution; high personal and economic costs of medication, and; desire for more information and access to natural approaches. Across SL, participants expressed keen interest in integrative approaches and better access to complimentary modalities. Participants in marginalized SL described reliance on traditional/folk remedies, including engagement with family and community healers, who they described as more affordable and culturally accessible.

**Discussion/Conclusions**: Holistic and integrative approaches were preferred over medication as long-term migraine management strategies. However, people in marginalized SL, while disproportionately disabled by migraine, did not feel as comfortable accessing integrative approaches in their current forms. Engaging these communities and using a critical lens to explore barriers to access can develop options to make complimentary modalities more approachable, while also attending to systemic blind spots that may unintentionally alienate socially marginalized groups.

### External Cold and Vibration for Pain Management of Children Undergoing Needle-Related Procedures in the Emergency Department: A Randomized Controlled Non-Inferiority Trial

Ariane Ballard^a^, Christelle Khadra^a^, Samara Adler^b^, Emilie Parent^c^, Evelyne D.Trottier^d^, Benoit Bailey^d^, Naveen Poonai^e^, Sylvie Le May^a^

^a^Faculty of Nursing, Université de Montréal, Montreal, Quebec, Canada; ^b^Faculty of Medicine, Université de Montréal, Montreal, Quebec, Canada; ^c^Faculty of Medicine, Université de Sherbrooke, Chicoutimi, Quebec, Canada; ^d^Division of Emergency Medicine, Department of Pediatrics, CHU Sainte-Justine, Montreal, Quebec, Canada; ^e^Division of Paediatric Emergency Medicine, Department of Paediatrics, London Health Sciences Centre, London, Ontario, Canada

**CONTACT** Ariane Ballard ariane.ballard@umontreal.ca; ariane.ballard@hotmail.com

© 2019 The Author(s). Published with license by Taylor & Francis Group, LLC.

This is an Open Access article distributed under the terms of the Creative Commons Attribution License (http://creativecommons.org/licenses/by/4.0/), which permits unrestricted use, distribution, and reproduction in any medium, provided the original work is properly cited.

**Introduction/Aim**: Needle-related procedures are the most important source of pain in children. Time constraints and heavy workload are barriers to the use of available interventions for pain management during these procedures. Therefore, the use of a rapid and easy-to-use intervention could improve pain management practices. This study aimed to determine if a device combining cold and vibration (Buzzy) was non-inferior to a topical anesthetic (Maxilene) for pain management of children undergoing needle-related procedures in the Emergency Department (ED).

**Methods**: This study was a randomized, controlled, non-inferiority trial. We enrolled children aged between 4–17 years presenting to the ED and requiring a needle-related procedure. The primary outcome was the mean difference in pain intensity during the procedure. The secondary outcomes were procedural distress and success of the procedure at first attempt.

**Results**: A total of 352 participants were enrolled and 346 were randomized (Buzzy = 172; Maxilene = 174). Mean difference in procedural pain scores between groups was 0.64 (95%CI −0.1 to 1.3), showing that the Buzzy device was not non-inferior to Maxilene according to a non-inferiority margin of 0.70. No significant differences were observed for procedural distress (p = .370) and success of the procedure at first attempt (p = .602).

**Discussion/Conclusions**: Non-inferiority of the Buzzy device over a topical anesthetic was not demonstrated for pain management of children during a needle-related procedure in the ED. However, considering that topical anesthetics are underused in the ED setting and require an application time, the Buzzy device seems to be a promising alternative as it is rapid, reusable, and easy-to-use.

### The Role of Human Chemosignals in Eliciting a Stress Response

Laila Chaudhry^a^, Sioui Maldonado^a^, Gabrielle Dutra^a^, Jeffrey S. Mogil^a^

Psychology, McGill University, Montreal, QC, Canada

**CONTACT** Laila Chaudhry laila.chaudhry@mail.mcgill.ca

© 2019 The Author(s). Published with license by Taylor & Francis Group, LLC.

This is an Open Access article distributed under the terms of the Creative Commons Attribution License (http://creativecommons.org/licenses/by/4.0/), which permits unrestricted use, distribution, and reproduction in any medium, provided the original work is properly cited.

**Introduction/Aim:** Our laboratory recently demonstrated that mere olfactory exposure to male experimenters can trigger stress-induced analgesia in rodents. Male sweat, and certain volatile chemosignaling compounds found therein–(E)-3-methyl-2-hexenoic acid (3M2H), androstenone, and androstadienone–were shown to cause reduction in pain behaviours and increases in corticosterone. This stress response to male chemosignals was also found to be concentration-dependent and particularly robust in female rodents. Androstenone and androstadienone have also been shown to raise cortisol levels in human women but not in men, for approximately one hour following olfactory exposure. 3M2H’s concentration in sweat is far higher than that of both androstadienone and androstenone, and although it has been studied far less than the other two compounds, it is reasonable to predict that 3M2H will produce a similar reaction effect: stress, and stress-induced analgesia in humans, particularly in women.

**Methods:** In the current stage of our study, we are confirming that 3M2H does indeed alter stress levels in a sex-dependent manner. Participants are exposed to 3M2H via an olfactometer, their mood and anxiety reported via visual analog scales, and their cortisol collected via saliva samples.

**Results:** We have observed significant sex differences, with males showing a drop in cortisol levels after exposure to high concentrations of 3M2H, while women’s cortisol levels remain unchanged.

**Discussion/Conclusions:** We believe that the robust sex differences displayed in stress responses might have important implications for the design of all human laboratory experiments, and possible clinical relevance to a number of mental disorders ranging from anxiety to depression to post traumatic stress disorder, and thus its explication may make unique contributions to the pain field.

### Validation of the 13-Item Pain Stages of Change Questionnaire (PSOCQ-13) in a Pediatric Chronic Pain Clinic

Kathy Xie^a^^b^^c^, Nez Elik^a^^b^^c^, Eleni Hapidou^a^^b^^c^

^a^Health Sciences, McMaster University, Hamilton, ON, Canada; ^b^Psychiatry and Behavioural Neurosciences, McMaster Children’s Hospital, McMaster University, Hamilton, ON, Canada; ^c^Psychiatry and Behavioural Neurosciences, Michael G. DeGroote Institute for Pain Research and Care, McMaster University, Hamilton, ON, Canada

**CONTACT** Kathy Xie xiek5@mcmaster.ca 

© 2019 The Author(s). Published with license by Taylor & Francis Group, LLC.

This is an Open Access article distributed under the terms of the Creative Commons Attribution License (http://creativecommons.org/licenses/by/4.0/), which permits unrestricted use, distribution, and reproduction in any medium, provided the original work is properly cited.

**Introduction/Aim**: While the use of the Pain Stages of Change Questionnaire (PSCOQ) to assess readiness for self-management is established in adults with chronic pain, there is paucity of research in children and youth. As quality of life can be severely impacted by chronic pain, developing a pediatric version of the PSOCQ can have significant clinical implications. This study aims to provide validation of the recently developed 13-item adolescent PSCOQ by Guite and colleagues in a pediatric sample.

**Methods**: Children and youth 10 to 18 years old enrolled in the Pediatric Chronic Pain Program at McMaster Children’s Hospital complete a set of intake questionnaires including the PSOCQ-13, chronic pain acceptance (CPAQ), pain self-efficacy (PSE), and pain coping (PCQ). Correlations will examine the internal reliability (Cronbach’s alpha) and concurrent validity of PSOCQ-13.

**Results**: Data from 60 children and youth will be obtained. It is expected there will be significant positive correlations between the action/maintenance subscale of the PSCOQ-13 and the CPAQ, PSE, and PCQ scores. There will be negative correlations between the pre-contemplation subscale of the PSCOQ-13 and the CPAQ, PSE, and PCQ scores. A subgroup of participants aged 10–12, when analyzed separately, is expected to produce results similar to the older (age 13–18) group.

**Discussion/Conclusions**: Initial validation of an adolescent PSOCQ by Guite and colleagues was not conclusive. Results from our study (underway) will help us determine the suitability of the 13-item adolescent version for use in children and youth, and provide directions for improvement.

### Predictors of Patient Satisfaction in a Four-Week Interdisciplinary Chronic Pain Management Program

Cindy Li^a^, Eleni G. Hapidou^b^

^a^Faculty of Science (continuing education), McMaster University, Hamilton, ON, Canada; ^b^Michael G. DeGroote Pain Clinic, Michael G. DeGroote Institute for Pain Research and Care, & Psychiatry and Behavioral Neurosciences, McMaster University, Hamilton, ON, Canada

**CONTACT** Cindy Li liyl2@mcmaster.ca

© 2019 The Author(s). Published with license by Taylor & Francis Group, LLC.

This is an Open Access article distributed under the terms of the Creative Commons Attribution License (http://creativecommons.org/licenses/by/4.0/), which permits unrestricted use, distribution, and reproduction in any medium, provided the original work is properly cited.

**Introduction/Aim**: Patient satisfaction (PS) is an important outcome in health care because it can indicate treatment effectiveness. Few studies have evaluated PS with chronic pain management. The purpose of this study was to examine PS as an outcome measure in a chronic pain program. Several psychosocial variables at pre- and post- treatment were examined as predictors.

**Methods**: The study sample consisted of 471 (52% female) individuals of 20– 79 years (M = 44, SD = 10.3) who had sustained heterogeneous injuries in motor vehicle accidents or the workplace. Average time since injury was 61.0 months (SD = 69.4), with a range of 6 to 684. Participants completed pre- and post-treatment measures of pain, disability, depression, catastrophizing, anxiety, stages of change, and pain acceptance. PS was measured post-treatment. The latter was developed in this program and previously reported on.

**Results**: We used multiple regression to examine predictors of PS. Pre- and post-treatment variables explained 53.0% of the variance (R^2^ = .53, F (24, 470) = 20.96, p < .001). Significant predictors included depression (β = −0.17, p < .005), acceptance (β = 0.11, p < .05), pre-contemplation, contemplation, and maintenance stages of change (β = −0.25, 0.16, 0.25, p < .001, respectively).

**Discussion/Conclusions**: Patients are more satisfied with pain management when they integrate self-management techniques in their lives, accept their pain more, and have better mood. They also recognize their ability to continue using these techniques post-treatment. Results are discussed within the patient satisfaction and pain program outcomes literature.

### Effectiveness of MEDi® for the Management of Children’s Pain and Fear during IV Induction: Help from a Humanoid Robot

Rachelle C.W. Lee^a^, Jacqueline R. Pearson^b^, Adam Spencer^c^, Melanie Noel^d^, Lisa Bell-Graham^b^, Tanya N. Beran^a^

^a^Department of Community Health Sciences, University of Calgary, Calgary, Alberta, Canada; ^b^Department of Child Life, Alberta Children’s Hospital, Calgary, Alberta, Canada; ^c^Department of Anesthesiology, University of Calgary, Calgary, Alberta, Canada; ^d^Department of Psychology, University of Calgary, Calgary, Alberta, Canada

**CONTACT** Rachelle Lee rcwlee@ucalgary.ca

© 2019 The Author(s). Published with license by Taylor & Francis Group, LLC.

This is an Open Access article distributed under the terms of the Creative Commons Attribution License (http://creativecommons.org/licenses/by/4.0/), which permits unrestricted use, distribution, and reproduction in any medium, provided the original work is properly cited.

**Introduction/Aim:** Intravenous (IV) induction can be a distressing experience for children receiving surgery. This randomized, controlled, two-armed study explored the effectiveness of a humanoid robot (MEDi®) programmed to deliver cognitive-behavioral strategies and teach deep breathing techniques to help children cope with IV induction.

**Methods:** Children (n = 137) ages 4–12 years were recruited from a tertiary pediatric hospital, and were randomly assigned to obtain induction according to standard protocol or with preparation from MEDi® prior to induction. Procedural pain and fear ratings were collected from children, parents, anesthesiologists, and researchers. Follow-up interviews were conducted to assess the recall of pain-related memories.

**Results:** Although no group differences were found for pain or fear (*ps* > 0.05), children who received preparation were more likely to complete the induction without an inhalation mask, compared to standard care, Fisher’s Exact Test *X2*(1) = 4.85, *p* = 0.04, φ*c* = 0.22. Specifically, the odds ratio indicates that patients with MEDi® preparation were 5.04 times more likely to complete the IV procedure than children without MEDi®. Many children recalled interacting with the robot (n = 12, 46.2% families interviewed) and had positive memories about the IV procedure. Among them, 33.3% of children remembered MEDi® and breathing exercises performed during the procedure.

**Discussion/Conclusions:** Despite being a safe, effective sedation method, IV induction is a painful and fear provoking procedure for many pediatric surgical patients. This study was the first to examine how a robot can assist children in learning strategies to cope with IV induction and suggests that it may help them tolerate IV procedures.

### The Role of mTORC2 in the Peripheral Nervous System in the Development of Chronic Pain

Calvin Wong^a^, Shannon Tansley^a^, Noosha Yosefpour^b^, Jieyi Yang^a^, Alfredo Ribeiro-Da-Silva^b^, Arkady Khoutorsky^a^

^a^Anesthesia, McGill University, Montreal, Quebec, Canada; ^b^Pharmacology & Therapeutics, McGill University, Montreal, Quebec, Canada

**CONTACT** Calvin Wong calvin.wong3@mail.mcgill.ca

© 2019 The Author(s). Published with license by Taylor & Francis Group, LLC.

This is an Open Access article distributed under the terms of the Creative Commons Attribution License (http://creativecommons.org/licenses/by/4.0/), which permits unrestricted use, distribution, and reproduction in any medium, provided the original work is properly cited.

**Introduction/Aim:** Changes associated with the development of pain involve the reorganization of pain circuitry, and alterations in gene expression. mTOR is a highly evolutionarily conserved serine/threonine kinase that regulates cell homeostasis through key cellular processes, including cell growth and proliferation, translation, autophagy, and cytoskeleton organization. mTOR is present in two structurally and functionally distinct multiprotein complexes: mTORC1 (mTOR Complex 1) and mTORC2. mTORC1 regulates the rate of mRNA translation. Much less is known about mTORC2, which has recently emerged as a key signaling molecule in a variety of cellular processes.

**Methods:** To study the role of mTORC2 in pain, we selectively ablated rictor, a key protein within the mTORC2, in Nav1.8-positive nociceptors. To ensure that behavioral effects are not a result of aberrant developmental changes from the conditional knockout of Rictor, immunohistochemistry and western blot analysis were performed. We also studied the effect of rictor conditional knockout (cKO) on intracellular signaling following inflammation and tissue injury. Furthermore, we used a drug compound, A-443654, that activates mTORC2.

**Results:** Our behavioral experiments demonstrate that rictor cKO mice exhibit reduced hypersensitivity in a model of inflammatory pain, complete Freund’s adjuvant, but not in the model of neuropathic pain, spared nerve injury. Western blotting and immunohistochemistry confirmed that developmental effects do not contribute to the observed phenotype. Administration of the mTORC2 activator A-443654 induced mechanical and thermal hypersensitivity.

**Discussion/Conclusions:** Our study demonstrates for the first time the central role of mTORC2 in nociceptors in the development of pain hypersensitivity in response to inflammation.

### Therapeutic Interventions for Acute Pain after Hand Injury or Surgery: An Evidence Synthesis Overview

Tara Packham^a^, Ruheena Sangrar^a^

School of Rehabilitation Sciences, McMaster University, Hamilton, Ontario, Canada

**CONTACT** Tara Packham packhamt@mcmaster.ca 

© 2019 The Author(s). Published with license by Taylor & Francis Group, LLC.

This is an Open Access article distributed under the terms of the Creative Commons Attribution License (http://creativecommons.org/licenses/by/4.0/), which permits unrestricted use, distribution, and reproduction in any medium, provided the original work is properly cited.

**Introduction/Aim**: Pain is a common feature in persons presenting with hand conditions including acute injuries, emergent post-traumatic and planned reconstructive surgeries. Effective pain management promotes recovery by facilitating rehabilitation participation. This evidence synthesis compares interventions addressing acute pain to support research-informed decision-making for conservative pain management in hand therapy practice.

**Methods**: Systematic reviews (SR) addressing effects on acute pain in hand or wrist injury and randomized clinical trials (RCTs) not captured previously were identified. The primary outcome of interest was pain reduction across short (<1), medium (1–6), and long-term outcomes (>6 months).

**Results**: Thirteen SRs and 16 RCTs met inclusion criteria. We grouped interventions into those using mobilization/immobilization, non-prescription medication, and modalities or supervised therapy. Robust findings were lacking to support exercise or early motion vs immobilization via casting or splinting for pain reduction. Pooled moderate quality studies supported topical NSAIDs compared to placebo. Few modalities were supported by more than a single trial, with the exception of transcutaneous electrical nerve stimulation, which demonstrated superiority to sham in pooled trials for acute and neuropathic pain. Supervised therapy attendance was not superior to tailored home exercise programs.

**Discussion/Conclusions**: No single treatment or approach emerged with high quality evidence to support effectiveness for acute pain management in hand therapy. How and when pain is measured in clinical studies varies greatly, and often lacks discussion around potential mechanisms of effect guiding treatment selection for individual clients. Clinicians should consider multi-modal approaches to reducing targeted pain outcome, as well as the multiple actions each intervention might accomplish.

### Her Heart, Her Story: A Grassroots Approach to Understanding Cardiac Pain in Women with Arthritis

Monica Parry^a^, Ann Kristin Bjørnnes^b^, Lihi Eder^c^, Paula Harvey^c^, Hafsa Ansari^a^, Jennifer La^a^, Jennifer Price^d^, Lynn Cooper^e^, Chitra Lalloo^f^, Dawn P. Richards^g^, Jennifer Stinson^f^, Judy Watt-Watson^a^, Linda Wilhelm^h^

^a^Lawrence S. Bloomberg Faculty of Nursing, University of Toronto, Toronto, Ontario, Canada; ^b^Department of Nursing and Health Promotion, Oslo Metropolitan University, Oslo, Norway; ^c^Department of Medicine, Women’s College Hospital, Toronto, Ontario, Canada; ^d^Women's College Research Institute, Women’s College Hospital, Toronto, Ontario, Canada; ^e^Patient Partner, Oshawa, Ontario, Canada; ^f^Peter Gilgan Centre for Research and Learning, Toronto, Ontario, Canada; ^g^Canadian Arthritis Patient Alliance, Toronto, Ontario, Canada; ^h^Canadian Arthritis Patient Alliance, Midland, Kings County, New Brunswick, Canada

**CONTACT** Monica Parry monica.parry@utoronto.ca

© 2019 The Author(s). Published with license by Taylor & Francis Group, LLC.

This is an Open Access article distributed under the terms of the Creative Commons Attribution License (http://creativecommons.org/licenses/by/4.0/), which permits unrestricted use, distribution, and reproduction in any medium, provided the original work is properly cited.

**Introduction/Aim**: Inflammatory arthritis (IA) is associated with an increased risk of coronary artery disease (CAD) morbidity and mortality but little is known about the clinical presentations associated with cardiac pain in women with IA. The aim of this study was to understand how women with IA understood, discussed, recognized and managed cardiac pain related to CAD.

**Methods**: We utilized a mixed method design to combine evidence from a comprehensive review of the literature on the self-management of cardiac pain in women with IA with the results of interviews with women with IA and cardiac pain.

**Results**: In total, 519 studies were included for full text assessment. However, *no study* described the self-management of cardiac pain or associated cardiac pain equivalents in women with IA. A total of five women were recruited for interviews; 80% (n = 4) had mild/moderate or high stress at home, reduced physical function (X = 4.6, SD = 2.1), moderate pain scores (X = 6.0, SD = 2.0, range 4.0– 8.5), and had high IA disease activity (n = 3, 60%). Most women (n = 3, 60%) were also the primary person responsible for housework in the home, completing an average of 18.2 hours (SD = 8.0) of housework per week (range 8– 30 hours per week).

**Discussion/Conclusions**: Our results suggest women with IA have a high burden of disease activity related to IA. More research is needed to better target treatment and follow-up support for these women.

### Evaluating the Impact of a Pain Management Group for Individuals with Neurological Diagnoses and Chronic Pain – A Pilot Study

Sarah Sheffe^a^, Bonnie Cai-Duarte^b^, Bronwen Moore^b^, Cara Kircher^b^, Janelle Panday^c^, Allison Freeman^d^, Deborah Hebert^a^

^a^Toronto Rehabilitation Institute, University Health Network, Ontario, Canada; ^b^Toronto Rehabilitation Institute, Living Engaged and Actively with Pain Service, University Health Network, Ontario, Canada; ^c^School of Rehabilitation Science, Toronto Rehabilitation Institute & McMaster University, Ontario, Canada; ^d^Toronto Rehabilitation Institute, ABI Program Services, University Health Network, Ontario, Canada

**CONTACT** Sarah Sheffe sarah.sheffe@uhn.ca

© 2019 The Author(s). Published with license by Taylor & Francis Group, LLC.

This is an Open Access article distributed under the terms of the Creative Commons Attribution License (http://creativecommons.org/licenses/by/4.0/), which permits unrestricted use, distribution, and reproduction in any medium, provided the original work is properly cited.

**Introduction/Aim:** Individuals with neurological diagnoses often have high rates of persistent pain; pain which does not subside over time. Psychological and physical interventions aimed at improving coping and acceptance of pain are successful at reducing pain and enhancing general well-being. The Living Engaged and Actively with Pain (LEAP) service provides therapy to clients with both a neurological diagnosis and chronic pain, offering a nine-week Pain Management Group (PMG). This pilot study was designed to explore the impact of the PMG on psychological and functional outcomes using mixed methods.

**Methods:** A pre- and post-test design was used, with participants (n = 40) completing measures of subjective pain, quality of life, and occupational goal progress. Measures were completed at three time points (pre-PMG, post-PMG and follow-up) along with a qualitative exit interview post-PMG. Scores were analyzed using paired t-tests. Constant comparative analysis was conducted on qualitative data.

**Results:** Participants showed significant positive changes in measures of pain catastrophizing, pain acceptance, quality of life, and goal performance and satisfaction. Three themes emerged from qualitative analysis: “Pros and Cons of PMG Format,” “The Importance of Peer Dynamics” and “Applying Group Content to Daily Life & Goals.”

**Discussion/Conclusions:** Results support that the PMG is a beneficial treatment plan for improving psychological status, functional abilities, and chronic pain management in persons with neurological diagnoses. Qualitative results highlight (1) that participants applied strategies learned in PMG to managing pain and meeting goals, and (2) potential areas of improvement to better tailor the group to client needs.

### Does Sex Influence Pain-Related Treatment Effects of the ABCD’s of Pain Management Psychoeducational Video?

Hannah Gennis^a^, Monica O’Neill^a^, Rebecca Pillai Riddell^a^, Anna Taddio^b^, Saul Greenberg^c^, Hartley Garfield^c^

^a^Psychology, York University, Toronto, Ontario, Canada; ^b^Pharmacy, University of Toronto, Toronto, Ontario, Canada; ^c^Pediatrics, University of Toronto, Toronto, Ontario, Canada

**CONTACT** Hannah Gennis hgennis@yorku.ca

© 2019 The Author(s). Published with license by Taylor & Francis Group, LLC.

This is an Open Access article distributed under the terms of the Creative Commons Attribution License (http://creativecommons.org/licenses/by/4.0/), which permits unrestricted use, distribution, and reproduction in any medium, provided the original work is properly cited.

**Introduction/Aim:** Exposing parents to the ABCD’s of Pain Management psychoeducational video is effective at reducing children’s pain-related distress in toddlers one- and two-minutes post-needle (Pillai Riddell et al., 2017). The effect of sex on these findings has yet to be explored. The aim of this analysis is to assess the interaction of treatment, age, and sex on young children’s pain related distress post-needle.

**Methods:** Parents of 6- and 18-month-olds (*n* = 64 each) were randomized to a video treatment – The ABCD’s (**A**ssess anxiety, **B**elly breathe, **C**alm close cuddle, and **D**istraction) of pain management or a placebo video. Pain-related distress was measured using the Modified Behaviour Pain Scale (MBPS; Taddio et al., 1995) at four distinct time points: immediately following the needle (1–15s), one-minute post-needle (61–75s), two minutes post-needle (121–135s), and three minutes post-needle (181–195s). Child sex was also recorded.

**Results:** Using a 2 (Treatment: ABCD vs. Control) X 2 (Age: 6- vs. 18-month) X 2 (Child Sex: Male vs. Female) MANOVA, a Treatment X Age interaction was found (*F* = 3.41, *p* = .01), such that 18-month-old toddlers in the treatment condition displayed less pain-related distress one- and two-minutes post-needle. However, there was no interaction found between treatment, age, and sex (*F* = 1.84, *p* = .13).

**Discussion/Conclusions:** Although the ABCD’s of pain management video appears to reduce pain-related distress post-needle in toddlers, this does not appear to be influenced by sex. Future research should assess the impact of treatment, age, and sex or gender in older children.

### Predictors of Long-Term Opioid Use in Chronic Non-Cancer Pain Patients: A Quebec Pain Registry Study

Jean-Luc Kaboré^a^, Hichem Saïdi^a^, Lise Dassieu^b^, Gabrielle Pagé^a^, Manon Choinière 0000-0001-9593-8883^a^

^a^Carrefour de l’innovation et de l’évaluation en santé, Centre de Recherche du Centre hospitalier de l’Université de Montréal, Montréal, Québec, Canada; ^b^Faculté de Médecine et des Sciences de la Santé, Chaire de Recherche en toxicomanie, Université de Sherbrooke, Longueuil, Québec, Canada

**CONTACT** Jean-Luc Kaboré benewende.jean.luc.kabore@umontreal.ca

© 2019 The Author(s). Published with license by Taylor & Francis Group, LLC.

This is an Open Access article distributed under the terms of the Creative Commons Attribution License (http://creativecommons.org/licenses/by/4.0/), which permits unrestricted use, distribution, and reproduction in any medium, provided the original work is properly cited.

**Introduction/Aim:** This study aimed at identifying biopsychosocial characteristics associated with long-term opioid treatment in chronic non-cancer pain patients.

**Methods:** This was a retrospective study from the Quebec Pain Registry. Consenting patients aged ≥ 18 years completed self-reported and nurse-administered questionnaires before their first visit (baseline) at one of five multidisciplinary pain treatment clinics and at 6- and 12-months follow-up. Three opioid use profiles (OUP) were defined: non-opioid users (not using opioids during the follow-up period), non-lasting opioid users (using opioids but not during the whole follow-up period) and lasting opioid users (using opioids during the whole follow-up period). Multivariate multinomial logistic regression analysis was used to identify predictors of OUP.

**Results:** A total of 788 patients participated (mean age of 52.0 ± 14.0 years, 38.2% male): 399 were non-opioid users, 245 were non-lasting opioid users and 144 were lasting opioid users. Severe pain intensity (OR = 1.8 (1.2– 2.8), p = 0.004) and moderate to severe depression (OR = 1.8 (1.2– 2.7), p = 0.003) at baseline were associated with lasting opioid use. Neuropathic pain (OR = 1.6 (95% CI: 1.0– 2.5), p = 0.045) and severe pain intensity (OR = 1.4 (95% CI: 1.0– 2.0), p = 0.038) predicted non-lasting opioid use.

**Discussion/Conclusions:** More severe pain and higher depression level were predictors of long-term OUP. Presence of neuropathic pain was associated with non-lasting opioid use. Further research is needed to identify which patients are most likely and least likely to benefit from long-term opioid use.

### An Examination of the Concurrent and Directional Relationships between Caregiver and Infant Cardiac Indicators of Pain-Related Distress during Vaccination

Miranda G. DiLorenzo^a^, Jordana A. Waxman^a^, Rebecca Pillai Riddell^a^, Hartley Garfield^b^

^a^Department of Psychology, York University, Toronto, ON, Canada; ^b^Pediatrics, University of Toronto, Toronto, ON, Canada

**CONTACT** Miranda DiLorenzo mgdilo@yorku.ca

© 2019 The Author(s). Published with license by Taylor & Francis Group, LLC.

This is an Open Access article distributed under the terms of the Creative Commons Attribution License (http://creativecommons.org/licenses/by/4.0/), which permits unrestricted use, distribution, and reproduction in any medium, provided the original work is properly cited.

**Introduction/Aim**: The coordination of distress responding and recovery with one’s caregiver plays an important role in structuring and consolidating an infant’s ability to regulate their distress (Feldman, 2012). The aim of this study was to provide preliminary evidence of coordination between caregiver and infant cardiac indicators of pain-related distress (heart rate (HR), respiratory sinus arrhythmia (RSA)) during vaccination.

**Methods**: Caregiver-infant dyads for this study were followed as part of an ongoing longitudinal study at 12-, 18-, and 24-month vaccinations. Only 12-month cardiac data (n = 73) will be reported in the current investigation. HR and RSA were analyzed during sequential 30-second epochs (30 seconds before the needle, immediately after the needle, 1-minute post-needle, and 2-minutes post-needle). Cross-lagged path models were estimated to examine concurrent and directional relationships between caregiver and infant cardiac indicators across epochs.

**Results**: Results indicated: 1) Across the 4 epochs, caregivers’ and infants’ RSA (and HR) are predicted by their preceding RSA (and HR); 2) there are no concurrent relationships between caregiver and infant cardiac indicators across epochs; and 3) generally, caregiver RSA (and HR) did not predict subsequent infant RSA (and HR) and vice versa.

**Discussion/Conclusions**: Results suggest coordination of caregiver and infant pain-related distress in a vaccination context does not manifest at 12 months. Statistical modeling (e.g. growth curve modelling) that discern groups of caregiver-dyads and their response trajectories should be used to better elucidate patterns of coordination (or lack of coordination) between caregivers and their infants, and the factors that influence patterns of coordination.

### Exploration of the Nociception Level (NOL)^TM^ Index for Pain Assessment during Endotracheal Suctioning in Mechanically Ventilated Patients in the Intensive Care Unit

Shiva Shahiri^a^, Melissa Richard-Lalonde^a^, Philippe Richebé^b^, Céline Gélinas^a^

^a^Ingram School of Nursing, Montreal, Canada, Jewish General Hospital - CIUSSS Centre-Ouest-Ile-Montreal, Centre for Nursing Research and Lady Davis Institute, McGill University, Montreal, Canada; ^b^Département d’anesthésiologie et de médecine de la douleur, Montreal, Canada, Hôpital Maisonneuve-Rosemont - CIUSSS Est-Ile-Montreal, Anesthésie, Université de Montréal, Montreal, Canada

**CONTACT** Shiva Shahiri shiva.shahiri@mail.mcgill.ca

© 2019 The Author(s). Published with license by Taylor & Francis Group, LLC.

This is an Open Access article distributed under the terms of the Creative Commons Attribution License (http://creativecommons.org/licenses/by/4.0/), which permits unrestricted use, distribution, and reproduction in any medium, provided the original work is properly cited.

**Introduction/Aim**: Pain is a common symptom in intensive care unit (ICU) patients. Alternative pain measures are necessary as many patients are non-communicative. Although behavioral pain measures (e.g., Critical-Care Pain Observation Tool or CPOT) are available, physiological measures are lacking The Nociception Level (NOL)^TM^ index incorporates simultaneously multiple physiological parameters (i.e., heart rate and its variability, pulse plethysmograph amplitude, skin conductance level, number of skin conductance fluctuations) to measure pain, but its use in the ICU is new. We explored the NOL for pain assessment in mechanically ventilated ICU patients during endotracheal suctioning.

**Methods**: A prospective cohort study was performed in a medical-surgical ICU in Montreal, Quebec. Data were collected at rest (T1), during endotracheal suctioning (T2), and 15 minutes post-procedure (T3). The NOL index ranges from 0 to 100 with values >25 indicative of pain The patient’s self-report of 0–10 pain intensity and 0–8 CPOT scores were also obtained. Friedman tests were used.

**Results**: Sixteen patients (56% males, mean age = 65) were included. The NOL (median, [interquartile range]) was significantly increased at T2 (34.86 [23.57–45.57]) compared with T1 (11.21 [4.48–24.56]) and T3 (13.04 [6.12–20.41]) (p < 0.001). Pain intensity and CPOT scores were higher at T2 (medians of 5 and 4, respectively) compared with T1 and T3 (medians of 0 for both scores) (p < 0.001).

**Discussion/Conclusions**: Consistent with pain intensity and CPOT scores, the NOL values were higher during endotracheal suctioning compared to pre/post-procedure. The NOL is an interesting pain assessment method requiring further validation testing in the ICU.

References1.
Gélinas
C. Pain assessment in the critically ill adult: recent evidence and new trends. Intensive Crit Care Nurs. 2016;34(5):1–11. doi:
10.1016/j.iccn.2016.03.001.270677452.
Devlin
JW, Skrobik
Y, Gélinas
C, Needham
DM, Slooter
AJC, Pandharipande
PP, … Alhazzani
W. Clinical practice guidelines for the prevention and management of pain, agitation/ sedation,delirium, immobility, and sleep disruption in adult patients in the ICU. Crit Care Med. 2018;46(9):e825–e873. doi:
10.1097/CCM.0000000000003299.301133793.
Ben-Israel
N, Kliger
M, Zuckerman
G, Katz
Y, Edry
R. Monitoring the nociception level: A multi-parameter approach. Int J Clin Monit Comput. 2013;27(6):659–68. doi:
10.1007/s10877-013-9487-9.23835792

### An Assessment of Opioid Prescribing Behaviors in Ontario Family Physicians before and after Participation in ECHO Chronic Pain/Opioid Stewardship

Santana Díaz^a^, Andrea Furlan^b^, Claire Bombardier^c^, Susan Jaglal^d^, Shawna Cronin^e^, Jane Zhao^f^

^a^Institute of Medical Sciences, University of Toronto, Toronto, Ontario, Canada; ^b^Toronto Rehab Institute UHN, Medicine/MSK, University of Toronto, Toronto, Ontario, Canada; ^c^Medicine/Rheumatology, University of Toronto, Mount Sinai Hospital UNH, Toronto, Ontario, Canada; ^d^Department of Physical Therapy, University of Toronto, Toronto, Ontario, Canada; ^e^IHPME, University of Toronto, Toronto, Ontario, Canada; ^f^MSK, Toronto Rehab, Toronto, Ontario, Canada

**CONTACT** Santana Díaz santana.diaz@uhn.ca

© 2019 The Author(s). Published with license by Taylor & Francis Group, LLC.

This is an Open Access article distributed under the terms of the Creative Commons Attribution License (http://creativecommons.org/licenses/by/4.0/), which permits unrestricted use, distribution, and reproduction in any medium, provided the original work is properly cited.

**Introduction/Aim**: Canada is in the middle of an opioid crisis. The Ontario Ministry of Health and Long-Term Care funded a demonstration project of the first replication of Project ECHO in Canada to tackle the opioid crisis. ECHO Ontario Chronic Pain/Opioid Stewardship started in June 2014. Participants attending ECHO acquire knowledge related to chronic pain management with or without opioids. This study aims to assess prescribing behaviours among family physicians who attended ECHO compared to those who did not.

**Methods**: We conducted an observational study with two control groups: a matched cohort and a random sample of 3,000 primary care physicians in Ontario using the Narcotics Monitoring System.

**Results**: We found that the ECHO group significantly reduced (p = 0.0047) high-dose opioid prescriptions by 20.2% after attending ECHO, compared to a non-significant increase of 0.8% in the matched cohort, and a non-significant reduction of 1.5% in the Ontario group during the same comparable periods.

**Discussion/Conclusions**: Ontario family physicians who participated in ECHO Chronic Pain/Opioid Stewardship had a greater reduction in the number of patients on high dose of opioids than those who did not participate in ECHO.

### A Canadian Perspective on the Availability and Access to Non-Pharmacological Treatments for Chronic Non-Cancer Pain

Colleen Donder^a^, Sarah Ndegwa^b^, Bert Dolcine^c^, Nina Frey^d^, Teo Quay^e^

^a^Knowledge Mobilization, CADTH, Winnipeg, MB, Canada; ^b^Health Research Consultant, Edmonton, AB, Canada; ^c^Program Development, CADTH, Ottawa, ON, Canada; ^d^Research Information Services, CADTH, Ottawa, ON, Canada; ^e^Manager Program Development – Medical Devices, CADTH, Toronto, ON, Canada

**CONTACT** Colleen Donder colleend@cadth.ca

© 2019 Canadian Agency for Drugs and Technologies in Health (CADTH). Published with license by Taylor & Francis Group, LLC.

This is an Open Access article distributed under the terms of the Creative Commons Attribution License (http://creativecommons.org/licenses/by/4.0/), which permits unrestricted use, distribution, and reproduction in any medium, provided the original work is properly cited.

**Introduction/Aim**: Non-pharmacological therapies are recommended first line by Canadian guidelines as a part of the multi-modal approach for managing chronic non-cancer pain. Non-pharmacological approaches are burdened by long wait times, cost, and limited access to those who reside outside of urban settings. This poster presents the information regarding available services, factors affecting access, strategies to improve access and funding practices related to non-pharmacological therapies for chronic non-cancer pain.

**Methods**: A survey, direct stakeholder consultations, and a literature review were conducted to gather information on non-pharmacological treatment availability, funding practices, access, and strategies to improve availability and access.

**Results**: Most non-pharmacological treatments for chronic non-cancer pain are available in Canada, mainly in urban settings with lower availability in rural settings and the lowest in remote settings. While treatments are available, access is limited due to long wait times for publically funded services and lack of funding for community based-services. Survey respondents indicated various barriers and facilitators to providing treatment and, also reported a need for more guidance on providing non-pharmacological treatments for non-cancer pain.

Creating a national pain strategy helped other countries create plans for equity of access to non-pharmacological interventions. Efforts are underway to create a pain strategy in Canada.

**Discussion/Conclusions**: Non-pharmacological treatments for chronic non-cancer pain are available in Canada; however, access is impeded by wait times and funding availability. More guidance is needed to direct treatment.

### Cognitive Depressive Symptoms and Helplessness Predict Pain at One Year in Women with Interstitial Cystitis/Bladder Pain Syndrome (IC/BPS)

Alison Crawford^a^, Dean A. Tripp^b^, J. Curtis Nickel^c^, Lesley Carr^d^, Robert Moldwin^e^, Robert Mayer^f^, Laura Katz^g^, Abi Muere^a^

^a^Department of Psychology, Queen’s University, Kingston, Ontario, Canada; ^b^Departments of Psychology, Urology, & Anesthesiology, Queen’s University, Kingston, Ontario, Canada; ^c^Department of Urology, Queen’s University, Kingston, Ontario, Canada; ^d^Department of Surgery, University of Toronto, Toronto, Ontario, Canada; ^e^Department of Urology, Hosftra University School of Medicine, New Hyde Park, New York, USA; ^f^Asante Physician Partners, Grants Pass, Oregon, USA; ^g^Michael G DeGroote Pain Clinic, McMaster University Hospital, Hamilton, Ontario, Canada

**CONTACT** Alison Crawford alison.crawford@queensu.ca

© 2019 The Author(s). Published with license by Taylor & Francis Group, LLC.

This is an Open Access article distributed under the terms of the Creative Commons Attribution License (http://creativecommons.org/licenses/by/4.0/), which permits unrestricted use, distribution, and reproduction in any medium, provided the original work is properly cited.

**Introduction/Aim:** Interstitial cystitis/bladder pain syndrome (IC/BPS) is a chronic pelvic pain condition characterized by urinary urgency, frequency, and dysuria. As the etiology of this condition is unknown, treatment focuses on symptom management. Previous research has demonstrated that helplessness mediates the relationship between depression and pain. The aim of this study was to understand the types of depressive symptoms driving the relationship, and whether this relationship was dependent on type of patient pain descriptors.

**Methods:** 135 women diagnosed with IC/BPS recruited from tertiary care clinics completed demographic, depressive symptoms, catastrophizing, and pain questionnaires at baseline, six months, and one year. Six mediation models were run using baseline cognitive/somatic depressive symptoms to helplessness at six months to affective/acute sensory/chronic sensory descriptors of pain at one year.

**Results:** Baseline cognitive depressive symptoms led to helplessness at six months, which led to descriptions of affective pain, acute sensory pain, and chronic sensory pain at one year while controlling for baseline somatic depressive symptoms, catastrophizing and pain. When controlling for cognitive symptoms, baseline somatic symptoms did not significantly predict helplessness at six months or affective pain, acute sensory pain, and chronic sensory pain descriptions at one year.

**Discussion/Conclusions:** Cognitive symptoms of depression are the driving factor of the longitudinal relationship between depression, helplessness, and pain in women with IC/BPS. This relationship does not differ according to patient affective, acute sensory, or chronic sensory pain descriptors. These findings highlight the importance of addressing patient cognitions through psychological intervention for effective pain management.

### Development of Interdisciplinary Pain Education in an Undergraduate Health Sciences Program

Jennifer V. Nash^a^, Eric P. Seidlitz^a^

Faculty of Health Sciences, Bachelor of Health Sciences (Honours) Program, McMaster University, Hamilton, ON, Canada

**CONTACT** Jennifer Nash nashjv@mcmaster.ca

© 2019 The Author(s). Published with license by Taylor & Francis Group, LLC.

This is an Open Access article distributed under the terms of the Creative Commons Attribution License (http://creativecommons.org/licenses/by/4.0/), which permits unrestricted use, distribution, and reproduction in any medium, provided the original work is properly cited.

**Introduction/Aim**: Recent attention to the opioid crisis has identified the need for increased discussion, communication, and collaboration in health care and at the community level. Significant gaps currently exist in interdisciplinary pain education. Increasing opportunities for learners to explore topics related to pain at the undergraduate level may help address this deficiency.

**Methods**: A new inquiry-based course was created within the Bachelor of Health Sciences (Honours) Program at McMaster University in 2014–2015. The objectives of this limited-enrolment course are: to develop an understanding of theories and mechanisms of pain, to gain an appreciation for the wide range of pain management techniques, to gain an appreciation for what it means to live with pain (e.g. the psychosocial context), and to work collaboratively with peers and utilize community resources in learning.

**Results**: Since its inception, this course has enrolled 96 students over 5 years. Each year, students work independently and in groups on pain topics relating to their own interests. In addition to in-class and online discussions, guest speakers from a variety of disciplines introduce their own perspectives on pain.

**Discussion/Conclusions**: This popular course provides an example of how interdisciplinary pain education can be integrated at the undergraduate level. It offers a tremendous opportunity to start conversations, introduce interdisciplinary collaboration, and inspire learners to pursue interests in pain research, policy, and practice. Bringing together faculty, students, professionals, and the community, this course addresses the need to promote pain as a topic for discussion and further study.

### Waiting Time for Multidisciplinary Pain Treatment: Associations with Improvement in Pain Interference for Patients with Rheumatic Conditions

Simon Deslauriers^a^, Jean-Sébastien Roy^b^, Sasha Bernatsky^c^, Debbie E. Feldman^d^, Anne Marie Pinard^e^, François Desmeules^f^, Mary-Ann Fitzcharles^g^, Kadija Perreault^b^

^a^Center for Interdisciplinary Research in Rehabilitation and Social Integration (CIRRIS), Université Laval, Faculty of Medicine, Québec, Québec, Canada; ^b^Department of Rehabilitation, CIRRIS, Université Laval, Québec, Québec, Canada; ^c^McGill University Health Centre (MUHC), McGill University, Research Institute of the McGill University Health Centre (RI-MUHC), Montréal, Québec, Canada; ^d^Centre for Interdisciplinary Research in Rehabilitation of Greater Montreal, Public Health Research Institute of Université de Montréal, Université de Montréal, Faculty of medicine, Montréal, Québec, Canada; ^e^Centre hospitalier universitaire (CHU) de Québec, Université Laval, Faculty of Medicine, Québec, Québec, Canada; ^f^Maisonneuve-Rosemont Hospital (CRHMR) Research Center, Université de Montréal, School of Rehabilitation, Montréal, Québec, Canada; ^g^McGill University Health Centre (MUHC), McGill University, Montréal, Québec, Canada

**CONTACT** Simon Deslauriers simon.deslauriers.1@ulaval.ca

© 2019 The Author(s). Published with license by Taylor & Francis Group, LLC.

This is an Open Access article distributed under the terms of the Creative Commons Attribution License (http://creativecommons.org/licenses/by/4.0/), which permits unrestricted use, distribution, and reproduction in any medium, provided the original work is properly cited.

**Introduction/Aim**: Over 25% of persons with rheumatic conditions reports frequent and severe joint pain. Access to multidisciplinary pain treatment facilities (MPTF) is critical. However, access to MPTF in Canada is limited by extensive waiting lists. Our aim was to assess, in patients with rheumatic conditions, associations between waiting time for MPTF and pain interference progression between the first MPTF appointment and the 6-month follow-up.

**Methods**: We conducted a retrospective study using the Quebec Pain Registry, a database of patients who received services within five MPTF in Québec between 2008 and 2014. Waiting time between referral and the initial appointment was categorized as < 2 months, 2–6 months and > 6 months. The outcome was change in Brief Pain Inventory (BPI) scores from baseline to 6 months. Multivariate analyses included generalized estimating equations.

**Results**: Of the 3665 patients studied, 26% waited < 2 months, 28% waited 2–6 months and 34% waited > 6 months (missing n = 437). Overall, there was a significant improvement in the BPI score from baseline to 6 months (p < 0.001). Patients who waited < 2 months had a significantly larger improvement in their BPI scores (−11.6, 95% confidence intervals [CI]: −9.6 – −13.5) versus patients who waited 2–6 months (−7.0, 95%CI: −5.5 – −8.5) or > 6 months (−5.1, 95%CI: −3.8 – −6.3).

**Discussion/Conclusions**: These results suggest that waiting time for MPTF is associated with improvement in pain interference, with a greater improvement for patients with rheumatic conditions who waited < 2 months versus patients who waited > 2 months.

### Lesional Trigeminal Neuralgia: Neuroimaging and Clinical Characterization of a New Trigeminal Pain Syndrome

Sarasa Tohyama^a^, Peter S. P. Hung^a^, Joshua C. Cheng^b^, Jia Y. Zhang^c^, Mojgan Hodaie^c^

^a^Institute of Medical Science, University of Toronto, Toronto, Ontario, Canada; ^b^Medicine, Stony Brook University, Stony Brook, New York, USA; ^c^Division of Brain, Imaging, and Behaviour – Systems Neuroscience, Krembil Research Institute, Toronto, Ontario, Canada

**CONTACT** Sarasa Tohyama sarasa.tohyama@mail.utoronto.ca

© 2019 The Author(s). Published with license by Taylor & Francis Group, LLC.

This is an Open Access article distributed under the terms of the Creative Commons Attribution License (http://creativecommons.org/licenses/by/4.0/), which permits unrestricted use, distribution, and reproduction in any medium, provided the original work is properly cited.

**Introduction/Aim**: Trigeminal neuralgia (TN) is a chronic facial pain condition distinguished by episodic attacks of severe, electrical pain. Patients with TN typically have an unremarkable MRI, with no identifiable brain lesions. Here, we report an unusual group of TN patients that present with a single lesion in the brainstem. We aim to define this new pain syndrome, which we term lesional trigeminal neuralgia (LTN), using a clinical and neuroimaging approach.

**Methods**: We retrospectively identified 25 LTN patients (10F, ages 43–87). To assess treatment response, 20 patients with sufficient follow-up data (i.e. > 6m) were examined. To assess the white matter properties of the brainstem lesions, a further subgroup of 12 LTN patients (5F, ages 43–79) and 12 matched healthy controls (5F, ages 39–78) with DTI scans were analyzed. White matter metrics of fractional anisotropy (FA), mean, radial, and axial diffusivities (MD, RD, and AD) were extracted from (1) the LTN brainstem lesions, (2) the contralateral, unaffected side, (3) and healthy controls.

**Results**: 19/20 LTN patients were non-responders to surgical treatment (mean no. of surgeries ± SD: 3.95 ± 2.33, range: 1–9), with the majority having undergone >3 procedures. The brainstem lesions demonstrated abnormal white matter microstructure, characterized by lower FA, and higher MD, RD, and AD.

**Discussion/Conclusions**: This study identifies a new, unique group of TN patients with a single brainstem lesion not in keeping with multiple sclerosis. Understanding their clinical and DTI features may help strategize therapeutic options, especially since these patients are non-responders to conventional surgical treatment.

### Prevalence of Falls and Associated Risk Factors in Adults Living with Chronic Pain

Etienne J. Bisson 0000-0002-0649-3550^a^^b^^c^, Jen Gemmell^b^, Sarah Kelly^b^, Adam Marsala^b^, Elizabeth Brown^a^, Mary Anne Good^a^, Rosemary Wilson 0000-0003-3262-243X^a^^d^^c^, Scott Duggan^a^^c^

^a^Chronic Pain Clinic, Kingston Health Sciences Centre-Hotel Dieu Hospital site, Kingston, Ontario, Canada; ^b^School of Rehabilitation Therapy, Queen’s University, Kingston, Ontario, Canada; ^c^Department of Anesthesiology and Perioperative Medicine, Queen’s University, Kingston, Ontario, Canada; ^d^School of Nursing, Queen’s University, Kingston, Ontario, Canada

**CONTACT** Etienne J. Bisson etienne.bisson@kingstonhsc.ca

© 2019 The Author(s). Published with license by Taylor & Francis Group, LLC.

This is an Open Access article distributed under the terms of the Creative Commons Attribution License (http://creativecommons.org/licenses/by/4.0/), which permits unrestricted use, distribution, and reproduction in any medium, provided the original work is properly cited.

**Introduction/Aim**: Falls and associated risk factors related to chronic pain (CP) have only been examined in older adults, yet falls are common regardless of age in this population. This study aimed to determine the prevalence of falls and associated risk factors in all adults living with CP.

**Methods**: This cross-sectional study used baseline data from the Kingston Health Sciences Centre Chronic Pain Registry extracted between November 2017 and October 2018, including sociodemographics, history of falls, and biopsychosocial measures of pain. Descriptive and bivariate analyses were performed to determine the prevalence of falls and to phenotype adults with CP who experienced a fall in the previous year. Using multivariate logistic regression, we examined age- and gender-adjusted factors associated with falls.

**Results**: Of the 317 adults with CP included in this study, 145 (45%) reported a total of 564 falls in the previous year. Among fallers, 81 (65%) had a recent fall (<3 months) and 91 (67%) had multiple falls. Prevalence of falls was independent of age. Fallers had greater pain severity and interference, lower physical function, greater depression and more pain sites compared to non-fallers. Pain severity (OR = 1.19 (95% CI: 1.03–1.38)) and physical function (OR = 0.960 (0.932–0.988)) were independently associated with falls.

**Discussion/Conclusions**: High prevalence of falls was found regardless of age for adults living with CP. Risk of falls increased with greater pain severity and lower physical function. A better understanding of circumstances, predictors and consequences of falls in all adults with CP is warranted.

### Acceptability and Feasibility of Quantitative Sensory Testing in Children: A Pilot Study

Alexandra Reda^a^, Christine T. Chambers 0000-0002-7138-916X^b^, Perri R. Tutelman^c^, Jennifer A. Parker 0000-0001-9900-4703^d^, Javeria Hashmi^e^

^a^Faculty of Medicine, Dalhousie University, Halifax, NS, Canada; ^b^Department of Pediatrics and Psychology and Neuroscience, Dalhousie University, Halifax, NS, Canada; ^c^Department of Psychology and Neuroscience, Dalhousie University, Halifax, NS, Canada; ^d^Centre for Pediatric Pain Research, IWK Health Centre, Halifax, NS, Canada; ^e^Department of Anesthesia, Pain Management & Perioperative Medicine, Dalhousie University, Halifax, NS, Canada

**CONTACT** Alexandra Reda Alexandra.reda@dal.ca

© 2019 The Author(s). Published with license by Taylor & Francis Group, LLC.

This is an Open Access article distributed under the terms of the Creative Commons Attribution License (http://creativecommons.org/licenses/by/4.0/), which permits unrestricted use, distribution, and reproduction in any medium, provided the original work is properly cited.

**Introduction/Aim**: Quantitative sensory testing (QST) refers to a group of procedures that assess perceptual responses to systematically applied and quantifiable sensory stimuli. QST has primarily been used to study pain in adults, but recently has also been used in pediatrics. The purpose was to examine the acceptability of QST from children’s and parents’ perspectives and to determine the feasibility of its use in future pediatric studies.

**Methods**: Children participated in 8 QST tasks (thermal detection and pain threshold, heat pain tolerance threshold, mechanical pain sensitivity, dynamic mechanical allodynia, and wind up ratio) and self-reported the discomfort they experienced (DISCOmfort in Research with Children questionnaire; DISCO-RC). To assess acceptability, parents and children reported on their satisfaction with the visit and experience with the QST protocol and questionnaires. The length of the visit and study completion rate were used to assess feasibility.

**Results**: Ten children aged 8–15 years (*M* = 10.8 years, *SD *= 2.15; 70% girls) and a parent (70% mothers) participated. On the DISCO-RC, children rated the tasks as “slightly painful” (thermal: *M = *2.20, *SD = *0.63, range = 1–5; mechanical: *M = *2.00, *SD *= 0.67, range = 1–5), and “slightly boring” (thermal: *M = *2.2, *SD *= 1.48, range = 1–5; mechanical: M = 1.6, *SD = *1.23, range = 1–5). Children and parents reported their experience as positive, indicated they were happy to participate, and said they would participate in a similar study again. They rated length of the visit as “just right” (children: *M = *4.8, *SD = *1.75, range = 0–10; parents: *M = *6.4, *SD = *1.65, range = 0–10). Study completion rate was 100%.

**Discussion/Conclusions**: The current protocol was acceptable and feasible for studying experimental pain and was associated with minimal discomfort in healthy children aged 8–15 years and their parents.

### Predictors of Prolonged Opioid Use following Prescription for Acute Musculoskeletal Injury

John J. Riva^a^, Salmi T. Noor^b^, Li Wang 0000-0003-1585-8846^c^, Vahid Ashoorion^c^, Farid Foroutan^b^, Behnam Sadeghirad^c^, Rachel Couban^c^, Gordon H. Guyatt 0000-0003-2352-5718^b^, and Jason W. Busse 0000-0002-0178-8712^c^

^a^Department of Family Medicine, McMaster University, Hamilton, ON, Canada; ^b^Department of Health Research Methods, Evidence and Impact, McMaster University, Hamilton, ON, Canada; ^c^Department of Anesthesia, McMaster University, Hamilton, ON, Canada

**CONTACT** John Riva rivaj@mcmaster.ca

© 2019 The Author(s). Published with license by Taylor & Francis Group, LLC.

This is an Open Access article distributed under the terms of the Creative Commons Attribution License (http://creativecommons.org/licenses/by/4.0/), which permits unrestricted use, distribution, and reproduction in any medium, provided the original work is properly cited.

**Introduction/Aim**: Opioids are prescribed for acute musculoskeletal injuries (<4 weeks), and some patients progress to long-term opioid use that extends beyond the anticipated duration of healing. Understanding factors that predict long-term opioid use would be helpful to identify high-risk patients at the time of prescribing.

**Methods**: We searched MEDLINE, EMBASE, Web of Science, and Google Scholar to identify studies that explored risk factors for prolonged opioid use after prescription for acute musculoskeletal injuries. When possible, we pooled estimates of association for all independent variables reported by more than one study.

**Results**: We screened 8,223 citations, of which 16 observational studies were eligible for review. Prevalence of prolonged opioid use in these studies ranged from 18% to 40%.

Factors associated with prolonged use of opioids were: (1) older age, (2) receipt of disability benefits, (3) lower levels of formal education, (4) substance use disorder, (5) previous opioid use, (6) anxiety disorder, (7)post-traumatic stress disorder, (8) history of suicide attempt, (9) receiving a higher dose of opioids (>90 mg morphine equivalent dose/day), (10) receiving opioid for >5 days, and (11) receiving treatment from high-intensity opioid prescribers. Attending a physical therapist was associated with a lower risk of prolonged opioid use after prescription for an acute injury.

**Discussion/Conclusions**: Our study identified patient and prescribing characteristics that can help inform policies to optimize opioid prescribing for acute musculoskeletal injuries.

### The Effect of Childhood Trauma on IBD Symptom Severity is Mediated by Pain

Valentina Mihajlovic^a^, Abi Muere^a^, and Dean Tripp^a^

Department of Psychology, Queen’s University, Kingston, Ontario, Canada

**CONTACT** Valentina Mihajlovic valentina.mihajlovic@queensu.ca

© 2019 The Author(s). Published with license by Taylor & Francis Group, LLC.

This is an Open Access article distributed under the terms of the Creative Commons Attribution License (http://creativecommons.org/licenses/by/4.0/), which permits unrestricted use, distribution, and reproduction in any medium, provided the original work is properly cited.

**Introduction/Aim**: Childhood trauma has been linked to increased incidence of inflammatory bowel disease (IBD) in adulthood, yet the mechanism by which trauma leads to IBD remains unknown. Considering that IBD is often accompanied by pain, and there is a relationship between childhood trauma and chronic pain, it is predicted that pain will mediate the relationship between childhood trauma and IBD symptom severity.

**Methods**: 103 individuals with IBD completed an online self-report survey. Childhood trauma was assessed using the Childhood Traumatic Events Scale. IBD symptom severity was assessed with a short version of the IBD Symptom Inventory. Pain was assessed using the pain body diagram from the McGill Pain Questionnaire; pain locations were quantified using a grid system.

**Results**: A simple mediation analysis revealed that childhood trauma indirectly influenced severity of IBD symptoms through its effects on body pain. Individuals who reported more severe childhood trauma rated their pain higher (*a* = .0672), and individuals with higher levels of pain report more severe IBD symptoms (*b* = 5.1279). A bootstrap confidence interval for the indirect effect (*ab* = .3447) based on 10,000 bootstrap samples was entirely above zero (.0868 to .6642). There was no evidence that childhood trauma influenced IBD symptom severity independent of its effect on body pain (*c’ *= .2623, *p* = .2739).

**Discussion/Conclusions**: This finding highlights the importance of screening for childhood trauma and the need for future studies to identify appropriate strategies to better manage pain in individuals with IBD so as to decrease their symptom severity.

### Comparative Analysis of Impairment Ratings from the 5th and 6th Editions of the AMA Guides

Jason W. Busse 0000-0002-0178-8712^a^, Marieke M. De Vaal^b^, S. John Ham^b^, Behnam Sadeghirad^a^, Loes W.A.H. Van Beers^b^, Rachel J. Couban^a^, Sun Makosso Kallyth^a^, and Rudolf W. Poolman^b^

^a^Anesthesia, McMaster University, Hamilton, ON, Canada; ^b^Department of Orthopedic Surgery, Onze Lieve Vrouwe Gasthuis, Amsterdam, The Netherlands

**CONTACT** Jason W. Busse bussejw@mcmaster.ca

Received 19 November 2018Accepted 14 January 2019

© 2019 The Author(s). Published with license by Taylor & Francis Group, LLC.

This is an Open Access article distributed under the terms of the Creative Commons Attribution License (http://creativecommons.org/licenses/by/4.0/), which permits unrestricted use, distribution, and reproduction in any medium, provided the original work is properly cited.

**Introduction/Aim**: The American Medical Association Guides to the Evaluation of Permanent Impairment (AMA Guides) are commonly used for assigning impairment ratings to injured workers; however, the association of AMA Guides edition (there are 6) with impairment ratings is uncertain.

**Methods**: We used data from a consecutive sample of 249 injured workers referred for an independent evaluation, 10 months before and after assessors switched from the 5^th^ to the 6^th^ edition of the AMA Guides. We also surveyed all Workers’ Compensation Boards (WCBs) in North America to determine what impairment rating system they used.

**Results**: Multivariable analysis showed a 36.4% relative reduction (95% confidence interval [CI] 17.2% to 57.3%) in impairment rating with the 6^th^ edition of the Guides vs. the 5^th^ edition. The majority of WCBs in North America (46 of 64; 71.9%) mandate use of the AMA Guides, including the 6th (n = 21; 32.8%), 5th (n = 11; 17.2%), 4th (n = 7; 10.9%), and 3rd (n = 3; 4.7%) editions. Two WCBs (3.1%) allow assessors to use any edition of the AMA Guides, and 2 (3.1%) allow assessors to use either the 5th or 6th edition. Ten Compensation Boards (15.6%) have developed their own impairment rating guides, and 8 WCBs (12.5%) allow assessors to use the impairment rating system of their choice

**Discussion/Conclusions**: The 6^th^ edition of the AMA Guides provides systematically lower impairment ratings for injured workers than the 5^th^ edition. Impairment rating systems should be standardized across WCBs.

### Testing the Effectiveness of A Pain Training for Respite Workers Supporting Children with Developmental Disabilities: A Randomized Controlled Trial Protocol

Lara M. Genik^a^, and C. Meghan McMurtry 0000-0002-3278-1169^a^

Department of Psychology, University of Guelph, Guelph, Ontario, Canada

**CONTACT** Lara M. Genik lgenik@uoguelph.ca

© 2019 The Author(s). Published with license by Taylor & Francis Group, LLC.

This is an Open Access article distributed under the terms of the Creative Commons Attribution License (http://creativecommons.org/licenses/by/4.0/), which permits unrestricted use, distribution, and reproduction in any medium, provided the original work is properly cited.

**Introduction/Aim**: Pain is common for children with developmental disabilities. Secondary caregivers (e.g., respite workers) who are less familiar with a given child and their unique pain expression may be at particular risk for underestimating pain. The objective of this poster is to present the research protocol for a randomized controlled trial which will investigate the effectiveness of a pain assessment and management training for respite workers who support children with developmental disabilities.

**Methods**: Participating children’s respite organizations in Ontario will be randomly assigned to a pain or control (family centered care) training condition. Immediately prior to the training, participants will complete: (a) a demographics questionnaire, (b) ratings of feasibility and their perceived skill/confidence in pain assessment and management, (c) a knowledge questionnaire, and (d) a vignette. Following completion of the 3 to 3.5 hour long training, they will complete (b) and (c) described above. Follow up will occur approximately one month after completion of the training, and will include completion of (b), (c), and (d) above, as well as participation in a focus group.

**Results**: The research protocol will be presented in detail. Data collection for this randomized controlled trial is complete, and analyses are ongoing.

**Discussion/Conclusions**: A number of methods and protocol-related decision points need to occur when conducting a randomized controlled trial. This is particularly the case when collaborating with multiple sites for data collection. The pros and cons of aspects of the presented protocol will be reviewed (e.g., feasibility, follow up time points).

### Conducting GENDER-Based Analysis of Existing Databases When Self-Reported GENDER Data are Unavailable: The GENDER Index

Anaïs Lacasse 0000-0002-3992-5145^a^, Gabrielle Pagé^b^, Manon Choinière 0000-0001-9593-8883^b^, Marc Dorais^c^, Bilkis Vissandjée^d^, Hermine Lore Nguena Nguefack^a^, Joel Katz 0000-0002-8686-447X^e^, Oumar Mallé Samb^a^, and Alain Vanasse^f^

^a^Département des sciences de la santé, Université du Québec en Abitibi-Témiscamingue (UQAT), Rouyn-Noranda, Québec, Canada; ^b^Centre de recherche du Centre hospitalier de l’Université de Montréal (CRCHUM); Département d’anesthésiologie et de médecine de la douleur, Faculté de médecine, Université de Montréal, Montréal, Québec, Canada; ^c^StatSciences Inc., Notre-Dame-de-l’Île-Perrot, Québec, Canada; ^d^Faculté des sciences infirmières, Université de Montréal, Montréal, Québec, Canada; ^e^Department of Psychology, Faculty of Health, York University, Toronto, Ontario, Canada; ^f^Centre de recherche du Centre hospitalier universitaire de Sherbrooke (CRCHUS), Département de médecine de famille et de médecine d’urgence, Faculté de médecine et des sciences de la santé, Université de Sherbrooke, Sherbrooke, Québec, Canada

**CONTACT** Anaïs Lacasse anais.lacasse@uqat.ca

© 2019 The Author(s). Published with license by Taylor & Francis Group, LLC.

This is an Open Access article distributed under the terms of the Creative Commons Attribution License (http://creativecommons.org/licenses/by/4.0/), which permits unrestricted use, distribution, and reproduction in any medium, provided the original work is properly cited.

**Introduction/Aim**: Growing attention has been given to considering sex and gender in pain clinical and epidemiological research. However, this remains a challenge in the context of retrospective studies where self-reported gender measures are often unavailable. We aimed to create a new gender index using data from the Canadian Community Health Survey (CCHS).

**Methods**: The GENDER Index was created using potentially gender-related variables available in the CCHS (selection based on scientific literature and expert opinion). Among workers aged 15–75 years who had no missing data for our variables of interest (n = 29 470 participants), a propensity score was derived from a logistic regression model that included gender-related variables as covariates and where sex served as the dependent variable. Face and construct validity of the index were examined.

**Results**: When looking at the distribution of the gender index in men and women, gender scores appeared related but partly independent (e.g., incomplete histogram overlap, variability of gender scores within each sex group). Differences also appeared in the proportion of women between groups categorized according to gender scores tertiles (p < .0001). Construct validity was established through associations between gender index scores and gender-related variables identified a priori such as choosing/avoiding certain foods because of weight concerns (p < .0001), caring for children as the most important thing contributing to stress (p = .0309), and ability to handle unexpected/difficult problems (p = .0375).

**Discussion/Conclusions**: According to our results, the GENDER Index could be useful to enhance the capacity of researchers using CCHS data to conduct gender-based analysis.

### Opioid Utilization and Perception of Pain Control in Hospitalized Patients: A Cross-Sectional Study of 11 Sites in 8 Countries

Marisha Burden^a^, Angela Keniston^b^, Mary Anderson Wallace^b^, Jason W. Busse 0000-0002-0178-8712^c^, Jordi Casademont^d^, Smitha R Chadaga^e^, Sumitra Chandrasekaran^e^, Marco Cicardi^f^, John M. Cunningham^g^, David Fiella^d^, Daniel Hoody^h^, David Hilden^h^, Ming-Ju Hsieh^i^, Yoon-Seon Lee^j^, Daniel D Melley^k^, Anna Munoa^g^, Francesca Perego^l^, Chin-Chung Shu^m^, Chang Hwan Sohn^j^, Jeffrey Spence^g^, Lindsay Thurman^b^, Cindy R Towns^n^, John You^o^, Luca Zocchi^p^, Richard K. Albert^b^

^a^Division of Hospital Medicine, University of Colorado, Denver, Colorado, USA; ^b^University of Colorado, Denver, Colorado, USA; ^c^Anesthesia, McMaster University, Hamilton, Ontario, Canada; ^d^Hospital de la Santa Creu i Sant Pau, Universitat Autònoma de Barcelona, Barcelona, Spain; ^e^Legacy Health, Legacy Emanuel Medical Center, Portland, Oregon, USA; ^f^Dipartimento di Medicina Interna, Università degli Studi di Milano, Milan, Italy; ^g^Denver Health, University of Colorado, Denver, Colorado, USA; ^h^Hennepin Healthcare Medical Center, Minneapolis, Minnesota, USA; ^i^National Taiwan University, Taipei City, Taiwan; ^j^Emergency Department, Asan Medical Center, Seoul, Korea; ^k^Imperial College, Chelsea and Westminster Hospital, London, UK; ^l^Department of Biomedical and Clinical Sciences, Ospedale Luigi Sacco-Polo Universitario, Milan, Italy; ^m^National Taiwan University Hospital, Taipei City, Taiwan; ^n^Wellington Hospital, Bioethics Centre, University of Otago, Dunedin, New Zealand; ^o^McMaster University, Hamilton, Ontario, Canada; ^p^Angelo Bellini Hospital (Somma Lombardo); Internal Medicine and Cardiac Rehab, Lombardia, Italy

**CONTACT** Marisha Burden MARISHA.BURDEN@ucdenver.edu

© 2019 The Author(s). Published with license by Taylor & Francis Group, LLC.

This is an Open Access article distributed under the terms of the Creative Commons Attribution License (http://creativecommons.org/licenses/by/4.0/), which permits unrestricted use, distribution, and reproduction in any medium, provided the original work is properly cited.

**Introduction/Aim**: Hospitalized patients are frequently treated with opioids for pain control and receipt of opioids at hospital discharge may increase risk of chronic opioid use. We aimed to explore inpatient analgesic prescribing patterns and patients’ perception of pain control between US and non-US hospitals.

**Methods**: Cross-sectional observational study conducted at 11 academic hospitals in the US and 7 other countries, including medical inpatients presenting with pain and analyzing the percent of patients given opioid and non-opioid analgesics during the first 24 to 36 hours of their hospitalization and at discharge and assessments of pain and beliefs about pain control

**Results**: We acquired completed surveys 503 of 719 patients in the US and 478 of 590 patients in other countries (75% response rate). Compared with patients in other countries, US patients who did or did not report taking opioids prior to admission were given opioids more frequently during their hospitalization (92% vs 70% and 71% vs 41%, respectively, P < 0.05). Patients who did not report taking opioids prior to admission were prescribed opioids more frequently at discharge (34% vs 15%, P < 0.05). These findings were not associated with improved satisfaction with pain control for US patients.

**Discussion/Conclusions**: Physicians in the US prescribe opioids more frequently during hospitalization than physicians in other countries, but this was not associated with improvement in patients’ satisfaction with pain control. Efforts to curb the US opioid epidemic should include inpatient analgesic prescribing practices as well as patients’ expectations regarding pain control.

### The Moderating Role of Toddler Effortful Control on Parent-Toddler Co-Regulation during Vaccinations

Oana Bucsea^a^, Miranda DiLorenzo^a^, Jordana Waxman^a^, and Rebecca Pillai Riddell^a^

Psychology, York University, Toronto, Ontario, Canada

**CONTACT** Oana Bucsea obucsea@yorku.ca

© 2019 The Author(s). Published with license by Taylor & Francis Group, LLC.

This is an Open Access article distributed under the terms of the Creative Commons Attribution License (http://creativecommons.org/licenses/by/4.0/), which permits unrestricted use, distribution, and reproduction in any medium, provided the original work is properly cited.

Previous research has revealed the presence of caregiver-toddler co-regulation (temporal coordination of biological states) during acute procedures. However, the impact of various dimensions of toddlers’ temperament, such as effortful control, on the relationship between caregivers’ and toddlers’ co-regulation has not yet been explored. The aim of the current study was to determine the moderating effect of toddlers’ effortful control on this relationship. The relationships between toddlers’ and caregivers’ heart rate variability (HRV), as well as toddlers’ effortful control, were measured during children’s 12- (n = 64) and 18-month- (n = 38) vaccinations. HRV, corresponding to respiratory sinus arrhythmia (RSA), was analyzed using the MindWare analysis system and it was determined for four different 30-second epochs: immediately before the needle, immediately after the needle, 1-minute post-needle (i.e. 60–89 seconds post needle), and 2-minutes post-needle (i.e .120 to 149 seconds post-needle). Parents reported on their toddlers’ effortful control using the Early Childhood Behaviour Questionnaire (ECBQ; Effortful Control subscale). At 12-months of age, there was no link between parent-toddler co-regulation and toddlers’ effortful control. At 18-months of age, toddler effortful control significantly moderated their co-regulation with their caregivers during the pain-related distress regulation phases at 1-minute (*b* = −.07, *p* < .01) and 2-minutes (*b* = −.07, *p* < .05) post-needle. Specifically, parent-toddler co-regulation only emerged in toddlers with low levels of effortful control. It appears that caregiver-toddler co-regulation may be achieved more readily in children exhibiting difficulties with effortful control during acute procedures, potentially due to their increased need for external sources of regulation.

**Introduction/Aim**: Previous research has revealed the presence of caregiver-toddler co-regulation (temporal coordination of biological states) during acute procedures. However, the impact of various dimensions of toddlers’ temperament, such as effortful control, on the relationship between caregivers’ and toddlers’ co-regulation has not yet been explored. The aim of the current study was to determine the moderating effect of toddlers’ effortful control on this relationship.

**Methods**: The relationships between toddlers’ and caregivers’ heart rate variability (HRV), as well as toddlers’ effortful control, were measured during children’s 12- (n = 64) and 18-month- (n = 38) vaccinations. HRV, corresponding to respiratory sinus arrhythmia (RSA), was analyzed using the MindWare analysis system and it was determined for four different 30-second epochs: immediately before the needle, immediately after the needle, 1-minute post-needle (i.e. 60–89 seconds post needle), and 2-minutes post-needle (i.e .120 to 149 seconds post-needle). Parents reported on their toddlers’ effortful control using the Early Childhood Behaviour Questionnaire (ECBQ; Effortful Control subscale).

**Results**: At 12-months of age, there was no link between parent-toddler co-regulation and toddlers’ effortful control. At 18-months of age, toddler effortful control significantly moderated their co-regulation with their caregivers during the pain-related distress regulation phases at 1-minute (*b* = −.07, *p* < .01) and 2-minutes (*b* = −.07, *p* < .05) post-needle. Specifically, parent-toddler co-regulation only emerged in toddlers with low levels of effortful control.

**Discussion/Conclusions**: It appears that caregiver-toddler co-regulation may be achieved more readily in children exhibiting difficulties with effortful control during acute procedures, potentially due to their increased need for external sources of regulation.

### The Effect of Smoking on Patients Attending a Tertiary Pain Management Center: A Propensity-Weighted Analysis on the Collaborative Health Outcomes Information Registry

James S. Khan^a^, Jennifer M. Hah^b^, and Sean C. Mackey^b^

^a^Department of Anesthesiology, University of Toronto, Toronto, Ontario, Canada; ^b^Department of Anesthesiology, Perioperative, and Pain Medicine, Stanford University, Palo Alto, California, USA

**CONTACT** James Khan james.khan@medportal.ca

© 2019 The Author(s). Published with license by Taylor & Francis Group, LLC.

This is an Open Access article distributed under the terms of the Creative Commons Attribution License (http://creativecommons.org/licenses/by/4.0/), which permits unrestricted use, distribution, and reproduction in any medium, provided the original work is properly cited.

**Introduction/Aim**: Tobacco smoking is associated with adverse health effects. Its relationship to pain is complex and previous studies have shown a bi-directional relationship. The longitudinal effect of smoking on patients attending a tertiary pain management center is not well-established.

**Methods**: Using the Collaborative Health Outcomes Information Registry (CHOIR) of patients attending the Stanford Pain Management Center from 2013 to 2017, we conducted a propensity-weighted analysis to determine the independent effects of smoking on chronic pain patients. We adjusted for covariates including age, gender, body mass index, depression and anxiety history, ethnicity, alcohol use, marital status, disability, and education. We compared smokers and non-smokers on pain intensity, functional, sleep, and psychological and mood variables using self-reported NIH PROMIS outcomes at consultation and over time.

**Results**: A total of 12,368 patients completed the CHOIR questionnaire of which 8,584 patients had complete data for propensity analysis. Smokers at time of pain consultation reported significantly worse pain intensities, pain interference, pain behaviors, physical functioning, fatigue, sleep-related impairment, sleep disturbance, anger, emotional support, depression, and anxiety symptoms than non-smokers (all p < 0.001). In the mixed model analysis, smokers tended to have worse pain interference, fatigue, sleep-related impairment, anger, emotional support, depression, and anxiety over time compared to non-smokers.

**Discussion/Conclusions**: Chronic pain patients who smoke tobacco have worse pain, functional, sleep, and psychological and mood outcomes compared to non-smokers. Smoking also has prognostic importance for poor recovery and improvement over time. Further research is needed on tailored strategies and therapies to assist chronic pain patients who smoke.

### A Systematic Review of Interventions to Promote Safer and More Effective Opioid Prescribing for Chronic Non-Cancer Pain

Jason W. Busse 0000-0002-0178-8712^a^, Zack van Allen^b^, Andrea Patey^b^, Behnam Sadeghirad^a^, Natasha Kithulegoda^c^, Rachel Couban^a^, Brittany Sauvé^d^, Norm Buckley^a^, Ramesh Zacharias^a^, Jeremy M. Grimshaw 0000-0001-8015-8243^e^, Noah M. Ivers 0000-0003-2500-2435^c^, David N. Juurlink^f^, John N. Lavis^g^, Per Olav Vandvik^h^, and Justin Presseau 0000-0002-2132-0703^b^

^a^Anesthesia, McMaster University, Hamilton, ON, Canada; ^b^Clinical Epidemiology Program, Ottawa Hospital Research Institute, Ottawa, Ontario, Canada; ^c^Department of Family and Community Medicine, Women’s College Hospital, University of Toronto, Toronto, Ontario, Canada; ^d^Opioid Response Team, Health Canada, Ottawa, Ontario, Canada; ^e^Department of Medicine, University of Ottawa, Ottawa, Ontario, Canada; ^f^Department of Medicine, Sunnybrook Health Sciences Centre, Toronto, Ontario, Canada; ^g^McMaster Health Forum, McMaster University, Hamilton, ON, Canada; ^h^Institute of Health and Society, Faculty of Medicine, University of Oslo, Oslo, Norway

**CONTACT** Jason Busse bussejw@mcmaster.ca

© 2019 The Author(s). Published with license by Taylor & Francis Group, LLC.

This is an Open Access article distributed under the terms of the Creative Commons Attribution License (http://creativecommons.org/licenses/by/4.0/), which permits unrestricted use, distribution, and reproduction in any medium, provided the original work is properly cited.

**Introduction/Aim**: Canada is the 2nd highest per-capita prescriber of opioids in the world. A number of interventions have been explored to promote evidence-based opioid prescribing, but there is uncertainty regarding features of effective strategies. We conducted a systematic review of studies exploring interventions to improve opioid prescribing for chronic non-cancer pain. We used the Behaviour Change Techniques (BCTs) Taxonomy to identify the active components of effective interventions.

**Methods**: We conducted systematic literature searches of MEDLINE, Cochrane Central, Google Scholar, OCLC OAISter database, and 3 trials registries (ISRCTN, ICTRP, and Clinical Trials.gov), until March 16, 2018. Articles eligible for our review were randomized, or observational studies that explored an intervention targeted at physicians to optimize opioid prescribing for chronic non-cancer pain. Trained teams of reviewers screened citations for eligible studies, abstracted data, and coded each trial according to the BCTs Taxonomy.

**Results**: Our search identified 5,746 citations, of which 42 proved eligible for our review. Education or electronic medical records alone were often ineffective, whereas, strategies that targeted clinician actions at the point-of-care, gave prescribers feedback, or practice support from pharmacists was more likely to improve opioid prescribing. Regarding BCTs, more effective interventions were more likely to include active strategies (e.g. problem solving, behavior substitution), peer support, material incentive and multiple components vs. simply providing instruction or information.

**Discussion/Conclusions**: Multicomponent interventions that involve providing alternatives to opioid prescribing for clinicians may be effective strategies to promote evidence-based opioid prescribing for chronic noncancer pain.

### The Relationship between Parent Attitudes Towards Vaccines and Use of a Knowledge Translation Resource for Children’s Vaccination Pain Management

Nicole E. MacKenzie^a^, Christine T. Chambers 0000-0002-7138-916X^a^, Melanie Barwick 0000-0002-2478-604X^b^, Kathryn A. Birnie^c^, Katelynn E. Boerner^d^, Vera Granikov^e^, Noni MacDonald^f^, C. Meghan McMurtry 0000-0002-3278-1169^g^, Jennifer A. Parker 0000-0001-9900-4703^h^, Pierre Pluye^e^, Anna Taddio^i^, and Perri R. Tutelman^a^

^a^Department of Psychology and Neuroscience, Dalhousie University, Halifax, Nova Scotia, Canada; ^b^Department of Psychiatry, Research Institute, Hospital for Sick Children and University of Toronto, Toronto, Ontario, Canada; ^c^Lawrence S. Bloomberg Faculty of Nursing, University of Toronto, Toronto, Ontario, Canada; ^d^Department of Psychiatry, BC Children’s Hospital & University of British Columbia, Vancouver, British Columbia, Canada; ^e^Department of Family Medicine, McGill University, Montreal, Quebec, Canada; ^f^Department of Pediatrics, Dalhousie University, Halifax, Nova Scotia, Canada; ^g^Psychology, University of Guelph, Guelph, Ontario, Canada; ^h^Centre for Pediatric Pain Research, IWK Health Centre, Halifax, Nova Scotia, Canada; ^i^Leslie Dan Faculty of Pharmacy, University of Toronto, Toronto, Ontario, Canada

**CONTACT** Nicole MacKenzie nmackenzie@dal.ca

© 2019 The Author(s). Published with license by Taylor & Francis Group, LLC.

This is an Open Access article distributed under the terms of the Creative Commons Attribution License (http://creativecommons.org/licenses/by/4.0/), which permits unrestricted use, distribution, and reproduction in any medium, provided the original work is properly cited.

**Introduction/Aim**: Parent-directed knowledge translation (KT) resources can inform and promote the use of evidence-based practices to manage children’s vaccination pain. This study used a KT resource to inform and potentially influence parents’ use of evidence-based pain management strategies for children’s vaccination pain. This study aimed to assess the relationship between parent’s attitudes toward childhood vaccines and intentions to use information from a vaccination pain management KT resource.

**Methods**: Parents of children aged 0–17 years viewed the KT resource about vaccination pain management strategies, developed in partnership with a parenting magazine. Parents completed the Parent Attitudes about Childhood Vaccines survey (PACV) to measure vaccine hesitancy (vaccination delay/refusal), and the content-validated Information Assessment Method for Parents to measure relevance and intention to use the KT resource.

**Results**: 155 parents completed the online survey (91% mothers, *n = *142). Parents demonstrated little to no vaccine hesitancy (83.1%, *n =* 128). PACV scores were negatively correlated with relevance of the KT resource (*r_s_* = −.185, *p* < .05) and intention to use the information (*r_s_* = −.221, *p* < .01). Parents with more positive attitudes towards vaccinations were more likely to be receptive to the KT resource and intended to use the strategies.

**Discussion/Conclusions**: In this primarily vaccine-accepting sample, positive attitudes towards childhood vaccination were associated with parents’ intention to use the pain management strategies in the KT resource. Further research is needed to inform whether KT resources can influence vaccine-hesitant parents toward acceptance and use of evidence-based pain management strategies.

### The Effects of Transcranial Direct Current Stimulation Associated with Graded Motor Imagery on Central Post-Stroke Pain: A Case Report

Rodrigo Deamo Assis^a^, Dat Nhut Nguyen^b^, Josée Boucher^b^, and Jacques Charest^c^

^a^Chronic Pain, Centre Intégré de Santé et des Services Sociaux – Abitibi Témiscamingue (CISSSAT), Rouyn-Noranda, Quebec, Canada; ^b^Chronic Pain, CISSSAT, Rouyn-Noranda, Quebec, Canada; ^c^Chronic Pain, Université du Québec en Abitibi-Témiscamingue, Rouyn-Noranda, Quebec, Canada

**CONTACT** Rodrigo Assis rodrigodeamoassis@yahoo.ca Regional Clinic of Pain, Abitibi-Témiscamingue, Québec, Canada

© 2019 The Author(s). Published with license by Taylor & Francis Group, LLC.

This is an Open Access article distributed under the terms of the Creative Commons Attribution-NonCommercial License (http://creativecommons.org/licenses/by-nc/4.0/), which permits unrestricted non-commercial use, distribution, and reproduction in any medium, provided the original work is properly cited.

**Introduction/Aim**: Central post-stroke pain (CPSP) is characterized by constant or intermittent pain associated with sensory abnormalities. The utilisation of transcranial direct current stimulation (tDCS) for the management of chronic pain and the utilisation of graded motor imagery (GMI) for the improvement of the motor function are well documented, but the association of tDCS+GMI on CPSP remains unclear. The presented case investigates the effectiveness of a tDCS+GMI protocol in the treatment of CPSP to decrease pain and to improve motor function.

**Methods**: A consenting male patient with right upper-extremity impairment and with the diagnosis of CPSP performed the tDCS+GMI protocol. This protocol involves placing an anodal tDCS over the left M1 and a cathodal tDCS over the right M1 while using graded motor imagery for 20 minutes during 5 consecutive-days. We used the visual analogue scale (VAS) and the McGill pain questionnaire – short form, for the pain measurement and the Nine Hole Peg Test (NHPT) for the motor function. The measures were taken at the beginning of the protocol, at the end of the protocol and 1 week after.

**Results**: Beginning of tDCS+GMI: VAS 6/10; McGill Pain 11/45 and NHPT 55,23 seconds. End of tDCS+GMI: VAS 1/10; McGill Pain 7/45 and NHPT 39,12 seconds. 1 week after tDCS+GMI: VAS 3/10; McGill Pain 7/45 and NHPT 33,36 seconds.

**Discussion/Conclusions**: For this patient the protocol combining tDCS+GMI was effective in decreasing pain and improving motor function.

### Sociodemographic Factors in Alberta’s Pediatric Pain Rehabilitation Program

Vishal Varshney^a^, Allison McPeak^a^, Jillian Vinall^a^, Nivez Rasic^b^, and Melanie Noel^c^

^a^Department of Anesthesia, University of Calgary, Calgary, Alberta, Canada; ^b^Department of Anesthesia, Vi Riddell Children’s Pain and Rehabilitation Service, Alberta Children’s Hospital, University of Calgary, Calgary, Alberta, Canada; ^c^Department of Psychology, Alberta Children’s Hospital Research Institute (Behaviour and the Developing Brain Theme), Hotchkiss Brain Institute, Mathison Centre for Mental Health Research and Education, University of Calgary, Calgary, AB, Canada

**CONTACT** Vishal Varshney vishal.varshney@ahs.ca

© 2019 The Author(s). Published with license by Taylor & Francis Group, LLC.

This is an Open Access article distributed under the terms of the Creative Commons Attribution License (http://creativecommons.org/licenses/by/4.0/), which permits unrestricted use, distribution, and reproduction in any medium, provided the original work is properly cited.

**Introduction/Aim**: Sociodemographic factors in children and families, such as gender, ethnicity and socioeconomic status, are associated with a higher prevalence of chronic pain, and may affect equitable access to tertiary or resource-intensive care. Pediatric pain rehabilitation programs, which offer intensive outpatient interdisciplinary treatments based on the biopsychosocial model of pain, demonstrate cost-effectiveness and functional improvement for youth with pain and significant pain-related disability. However, few exist in North America, and their resource-intensive nature limits access to many children with chronic pain. We sought to examine sociodemographic data of youth enrolled in our intensive pain rehabilitation program (IPRP), and compare it to published sociodemographic characteristics of children with chronic pain.

**Methods**: A retrospective analysis of patients admitted to the IPRP over 3 years (N = 27, mean age 16.2 years) was performed.

**Results**: Enrolled patients were predominantly Caucasian (81.5%), female (81.5%), and 65% reported an annual household income greater than $90,000. Mean pain duration was 38.1 ± 28.1 months. Primary pain complaints in the IPRP were varied, with 44.4% listing neuropathic pain.

**Discussion/Conclusions**: Our findings reflect literature data that pediatric chronic pain is more prevalent in females than males, but show underrepresentation of low-income households despite lower socioeconomic status being associated with a higher prevalence of chronic pain. The findings also suggest underrepresentation of minorities, despite minority status being a risk factor for functional limitation from physical symptoms. These are similar to other North American pain rehabilitation programs. Continued focus in this area may involve diversifying referral bases and broadening exposure of our IPRP to more at-risk demographics.

### The Effect of Opioid Schedule, Formulation, Rotation and Tapering among Patients with Chronic Non-Cancer Pain: A Systematic Review and Meta-Analysis

Vahid Ashoorion^a^, Madison Zhang^b^, Li Wang^a^, Samantha Craigie^b^, Paul Bruno^c^, Luciane C. Lopes^d^, Yechan Kim^e^, Yaping Chang^f^, Nicole Vogel^g^, Sureka Pavalagantharajah^h^, Mehdi Ghasemi^i^, Kayli Culig^j^, Kyle De Oliveira^k^, Arnav Agarwal^l^, Rachel Couban^a^, Patrick J. Hong^m^, Stephanie Ross^b^, Regina Kunz^n^, Yung Lee^o^, Norman Buckley 0000-0002-1031-6813^a^, Daniel I. Sessler^p^, Gordon H. Guyatt 0000-0003-2352-5718^b^, and Jason W. Busse 0000-0002-0178-8712^a^

^a^Department of Anesthesia, McMaster University, Hamilton, ON, Canada; ^b^Department of Health Research Methods, Evidence and Impact, McMaster University, Hamilton, Ontario, Canada; ^c^Kinesiology and Health Studies, University of Regina, Regina, SK, Canada; ^d^Pharmaceutical Science, University of Sorocaba, Soracaba, Sao Paolo, Brazil; ^e^Firestone Institute for Respiratory Health, McMaster University, Hamilton, ON, Canada; ^f^OrthoEvidence Inc., Burlington, Ontario, Canada; ^g^Hirslanden Klinik Birshof, Leonardo, Münchenstein, Switzerland; ^h^Faculty of Medicine, McMaster University, Hamilton, Ontario, Canada; ^i^Anesthesia, Isfahan University of Medical Sciences, Isfahan, Iran; ^j^Faculty of Medicine, University of Toronto, Toronto, Ontario, Canada; ^k^Anesthesiology and Pain Medicine, Anesthesiology and Pain Medicine, University of Ottawa, Ottawa, Ontario, Canada; ^l^Department of Medicine, University of Toronto, Toronto, Ontario, Canada; ^m^Faculty of Medicine, University of Ottawa, Ottawa, Ontario, Canada; ^n^Department of Clinical Research, University Hospital, Basel, Switzerland; ^o^Michael G. DeGroote School of Medicine, McMaster University, Hamilton, ON, Canada; ^p^Department of Outcomes Research, Anesthesiology Institute, Cleveland Clinic, Cleveland, OH, USA

**CONTACT** Vahid Ashoorion ashooriv@mcmaster.ca

© 2019 The Author(s). Published with license by Taylor & Francis Group, LLC.

This is an Open Access article distributed under the terms of the Creative Commons Attribution License (http://creativecommons.org/licenses/by/4.0/), which permits unrestricted use, distribution, and reproduction in any medium, provided the original work is properly cited.

**Introduction/Aim**: The impact of opioid dosing approaches for chronic non-cancer pain are uncertain, as is the effect of multidisciplinary support for those patients attempting to reduce their opioid dose.

**Methods**: We searched Medline, EMBASE and Cochrane CENTRAL to the end of August 2018 for trials that explored the impact of opioid dosing, formulations, or supported tapering on patients with chronic noncancer pain.

**Results**: We identified 24 eligible studies for our review (5 randomized and 19 observational studies). Moderate to very low-quality evidence showed that immediate vs controlled release opioids, or as needed vs. scheduled dosing does not affect pain relief, physical functioning or gastrointestinal-related side effects. Very low quality of evidence showed that opioid rotation may reduce pain (weighted mean difference [WMD] −27.1 on a 100 mm VAS for pain, 95%CI −37.1 to −17.1). Low and very-low quality evidence showed that multidisciplinary tapering support is associated with reduced pain (WMD −14.3 mm, 95%CI −20 to −8.6), increased physical functioning (WMD 10.38 points on the 100-point physical functioning subscale of the SF-36, 95%CI 2.65 to 18.1), and improved mental health (WMD 8.71 points on the 100-point SF-36 mental health subscale of the SF-36, 95%CI 7.61 to 9.8). Our pooled analysis showed 88.0% (95%CI 83.0 to 93.0) of chronic pain patients successfully completed opioid withdrawal with multidisciplinary support.

**Discussion/Conclusions**: Limited evidence found no difference in outcomes for chronic pain patients when opioids were administered in different ways. Multidisciplinary support may be effective for assisting patients with chronic non-cancer pain with voluntary opioid tapering efforts.

### The Neural Mechanisms behind Conditioned Analgesia in Chronic Neuropathic Pain

Chulmin Cho^a^, Vassilia Michailidis^b^, Areej Fatima^a^, Hyun Been Park^c^, Batul Presswala^a^, Natalia Dziekonski^a^, and Loren J. Martin^a^

^a^Psychology, University of Toronto, Toronto, ON, Canada; ^b^Cell Systems Biology, University of Toronto, Toronto, ON, Canada; ^c^Molecular Genetics, University of Toronto, Toronto, ON, Canada

**CONTACT** Chulmin Cho chulmin.cho@utoronto.ca

© 2019 The Author(s). Published with license by Taylor & Francis Group, LLC.

This is an Open Access article distributed under the terms of the Creative Commons Attribution License (http://creativecommons.org/licenses/by/4.0/), which permits unrestricted use, distribution, and reproduction in any medium, provided the original work is properly cited.

**Introduction/Aim**: Placebo analgesia is mediated and strengthened by learning such as conditioning. However, the neurobiological correlates of learning for pain remain elusive. Towards that goal, the central objective of the study is to delineate the neural pathways that contribute to conditioned analgesia using a mouse model of chronic neuropathic pain.

**Methods**: Mechanical pain thresholds were measured using von Frey filaments in 6-8wk old male CD-1 mice, before and following spared nerve injury (SNI). The SNI mice then underwent a four-day conditioning phase where contextual (Plexiglas cubicles) and tactile (intraperitoneal injection) stimuli were coupled with an unconditioned drug stimulus (morphine, 10mg/kg). Following the conditioning period, the SNI mice were administered either saline or one of the opioid receptor antagonists. Following behavioral testing, neuronal activity was mapped in the spinal cord and brain by probing for c-fos expression using immunoblotting and immunohistochemistry.

**Results**: After pharmacological conditioning, saline administration was analgesic comparable to that of morphine, which was reversed by naloxone. Upon immunoblotting, the observed conditioned analgesia was negatively correlated with neuronal activity in the dorsal horn of the spinal cord. Furthermore, immunohistochemical analyses revealed significant changes in neuronal activity in pain processing regions of the brain.

**Discussion/Conclusions**: Here, we demonstrate a novel animal model of conditioned analgesia within the context of chronic neuropathic pain. The changes in neuronal activity in the brain and spinal cord corresponds to those observed in humans, warranting further investigations in order to elucidate the underlying neural basis for conditioned analgesia.

### Sociodemographic Factors in Alberta’s Pediatric Pain Rehabilitation Program

Vishal Varshney^a^, Allison McPeak^a^, Jillian Vinall^a^, Nivez Rasic^b^, and Melanie Noel^c^

^a^Department of Anesthesia, University of Calgary, Calgary, Alberta, Canada; ^b^Department of Anesthesia, Vi Riddell Children’s Pain and Rehabilitation Service, Alberta Children’s Hospital, University of Calgary, Calgary, Alberta, Canada; ^c^Department of Psychology, Alberta Children’s Hospital Research Institute (Behaviour and the Developing Brain Theme), Hotchkiss Brain Institute, Mathison Centre for Mental Health Research and Education, University of Calgary, Calgary, AB, Canada

**CONTACT** Vishal Varshney vishal.varshney@ahs.ca

© 2019 The Author(s). Published with license by Taylor & Francis Group, LLC.

This is an Open Access article distributed under the terms of the Creative Commons Attribution License (http://creativecommons.org/licenses/by/4.0/), which permits unrestricted use, distribution, and reproduction in any medium, provided the original work is properly cited.

**Introduction/Aim**: Sociodemographic factors in children and families, such as gender, ethnicity and socioeconomic status, are associated with a higher prevalence of chronic pain, and may affect equitable access to tertiary or resource-intensive care. Pediatric pain rehabilitation programs, which offer intensive outpatient interdisciplinary treatments based on the biopsychosocial model of pain, demonstrate cost-effectiveness and functional improvement for youth with pain and significant pain-related disability. However, few exist in North America, and their resource-intensive nature limits access to many children with chronic pain. We sought to examine sociodemographic data of youth enrolled in our intensive pain rehabilitation program (IPRP), and compare it to published sociodemographic characteristics of children with chronic pain.

**Methods**: A retrospective analysis of patients admitted to the IPRP over 3 years (N = 27, mean age 16.2 years) was performed.

**Results**: Enrolled patients were predominantly Caucasian (81.5%), female (81.5%), and 65% reported an annual household income greater than $90,000. Mean pain duration was 38.1 ± 28.1 months. Primary pain complaints in the IPRP were varied, with 44.4% listing neuropathic pain.

**Discussion/Conclusions**: Our findings reflect literature data that pediatric chronic pain is more prevalent in females than males, but show underrepresentation of low-income households despite lower socioeconomic status being associated with a higher prevalence of chronic pain. The findings also suggest underrepresentation of minorities, despite minority status being a risk factor for functional limitation from physical symptoms. These are similar to other North American pain rehabilitation programs. Continued focus in this area may involve diversifying referral bases and broadening exposure of our IPRP to more at-risk demographics.

### Chronic Pain in High Frequency Users of the Thunder Bay Regional Health Sciences Centre Emergency Department

Barbara Gunka^a^, Ocean Nenadov^a^, Samantha Biggs^a^, and Bryan MacLeod^a^

Medicine, Northern Ontario School of Medicine, Thunder Bay, Ontario, Canada

**CONTACT** Barbara Gunka bgunka@nosm.ca

© 2019 The Author(s). Published with license by Taylor & Francis Group, LLC.

This is an Open Access article distributed under the terms of the Creative Commons Attribution License (http://creativecommons.org/licenses/by/4.0/), which permits unrestricted use, distribution, and reproduction in any medium, provided the original work is properly cited.

**Introduction/Aim**: Chronic pain (CP) is defined as pain that persists for more than 3 months or beyond the expected healing time, CP affects 20-30% of the population worldwide.^1^ Lack of community resources and primary care access can lead to frequent, costly, and avoidable emergency department (ED) visits and hospital admissions. In Ontario, utilization of the ED is highest in the North West LHIN, with the highest usage at the Thunder Bay Regional Health Sciences Centre (TBRHSC).^2^ Approximately 70% of ED visits are prompted by pain.^3^

**Methods**: Phase I included a retrospective analysis of 283 randomly selected high frequency users (HFU) of the ED, defined as having 8 or more visits to the TBRHSC ED in the 2014–2015 fiscal year.

**Results**: Preliminary results demonstrate that of the sample, 28% of individuals visiting the TBRHSC ED met the criteria as chronic pain high frequency users. Only 4.5% of these CP HFU were involved with other pain management services. CP HFU had a mean of 17 visits versus 12 for those without CP. Additionally, CP HFU were discharged from ED more often than non CP sufferers.

**Discussion/Conclusions**: Patients presenting to the TBRHSC ED often have complex concerns that are not fully addressed. A very small population of CP sufferers have access to interdisciplinary pain management. Improving community resources is a requirement to reduce the costly demand on the ED and CP sufferers. This poster presents the analysis of data derived from CP sufferers of the ED at the TBRHSC.

References1.
Schopflocher
D, Taenzer
P, Jovey
R. The prevalence of chronic pain in Canada. Pain Res Manag. 2011;16:445–50.2218455510.1155/2011/876306PMC32980512.
LLP
K. North WEST LHIN Regional Emergency Department Study.
North West Local Health Integration Network; 2009.3.
Neighbor
ML, Puntillo
KA, Homel
P, Todd
K. Chronic pain in the emergency department. Acad Emerg Med. 2007;14(Suppl):S112. doi:
10.1197/j.aem.2006.07.032.

### The Global Biomechanical and Morphological Assessment: Understanding Back Pain in Adolescent Idiopathic Scoliosis

Maxime St-Georges^a^, Alisson R. Teles^b^, Neil Saran^c^, Jean A. Ouellet^c^, and Catherine E. Ferland^d^

^a^Experimental Surgery, McGill University, Montréal, Québec, Canada; ^b^Integrated Program in Neuroscience, McGill University, Montréal, Québec, Canada; ^c^Orthopaedic Surgery, McGill University, Montréal, Québec, Canada; ^d^Anesthesia, McGill University, Montréal, Québec, Canada

**CONTACT** Maxime St-Georges maxime.st-georges@mail.mcgill.ca

© 2019 The Author(s). Published with license by Taylor & Francis Group, LLC.

This is an Open Access article distributed under the terms of the Creative Commons Attribution License (http://creativecommons.org/licenses/by/4.0/), which permits unrestricted use, distribution, and reproduction in any medium, provided the original work is properly cited.

**Introduction/Aim:** Half of patients with Adolescent Idiopathic Scoliosis (AIS) experience pain. It is still unknown if corrective spinal surgery alleviates pain. This study’s aim was to create a 3-dimensional biomechanical and morphological assessment to better quantify perioperative back pain in AIS patients.

**Methods:** Twenty patients with AIS scheduled for spinal fusion surgery were recruited. X-rays of the patient wearing sensory insoles standing upon a Cartesian plane were taken using EOS Imagery before and after surgery. Plantar pressure distribution was measured using Moticon insoles. Self-reported pain intensity and location were collected using a grid divided back diagram. Regression analyses to assess variables’ correlations, and paired t-tests to identify timepoints differences were performed.

**Results:** Pain intensity diminished after surgery (NRS: 4.728 versus 2.722, t = 2.350, p = 0.031). The thoracolumbar/lumbar region pain intensity was significantly reduced (NRS: 3.425 versus 0.814 after, t = 4.04 p = 0.001). Greater pressure difference between feet was associated with greater curve severity (p = 0.018 r^2^ = 0.395 before and p = 0.013 r^2^ = 0.347 after). All curve angles, the thoracic apical vertebral rotation, the torsion of the spine and the lordosis were reduced after surgery (p < 0.001, p = 0.009; p < 0.001; p = 0.021).

**Discussion/Conclusions:** Preliminary results suggest that surgical correction of AIS causes a normalization of the spine without affecting the plantar pressure distribution. Surgical correction alleviates pain, especially in the thoracolumbar/lumbar region possibly due to the reduction of lordosis.

### Management of Acute, Non-Back, Musculoskeletal Pain: A Systematic Review and Network Meta-Analysis of Randomized Trials

Jason W. Busse 0000-0002-0178-8712^a^, Behnam Sadeghirad^a^, Yvgeniy Oparin^b^, Eric Chen^b^, Anna Goshua^c^, Curtis May^d^, Patrick J Hong^e^, Arnav Agarwal^f^, Yaping Chang^g^, Peter Emary^h^, Stephanie Ross^h^, Ivan D. Florez^h^, Salmi Noor^h^, William Yao^b^, Syed Hussain Ali^b^, Samantha Craigie^h^, Rachel Couban^a^, Rebecca L. Morgan^h^, Kayli Culig^i^, Sonia Brar^j^, Khashayar Akbari-Kelachayeh^k^, Laxsanaa Sivananthan^l^, Bahareh Zihayat^m^, Aninditee Das^b^, Alex Pozdnyakov^b^, and Gordon H. Guyatt^h^

^a^Department of Anesthesia, McMaster University, Hamilton, Ontario, Canada; ^b^Michael G. DeGroote School of Medicine, McMaster University, Hamilton, Ontario, Canada; ^c^School of Medicine, Stanford University, Stanford, California, USA; ^d^Faculty of Medicine, University of British Columbia, Vancouver, British Columbia, Canada; ^e^Faculty of Medicine, University of Ottawa, Ottawa, Ontario, Canada; ^f^Department of Medicine, University of Toronto, Toronto, Ontario, Canada; ^g^OrthoEvidence Inc., Burlington, Ontario, Canada; ^h^Department of Health Research Methods, McMaster University, Hamilton, Ontario, Canada; ^i^Faculty of Medicine, Evidence and Impact, University of Toronto, Toronto, Ontario, Canada; ^j^Jacobs School of Medicine & Biomedical Sciences, University at Buffalo, Buffalo, New York, USA; ^k^Faculty of Health Sciences, McMaster University, Hamilton, Ontario, Canada; ^l^Graduate Entry Medical School, University of Limerick, Limerick, Ireland; ^m^Faculty of Pharmacy, Kerman University of Medical Sciences, Kerman, Iran

**CONTACT** Jason W. Busse bussejw@mcmaster.ca

© 2019 The Author(s). Published with license by Taylor & Francis Group, LLC.

This is an Open Access article distributed under the terms of the Creative Commons Attribution License (http://creativecommons.org/licenses/by/4.0/), which permits unrestricted use, distribution, and reproduction in any medium, provided the original work is properly cited.

**Introduction/Aim**: Clinicians have access to multiple treatment options for acute musculoskeletal (MSK) pain. We assessed the comparative effectiveness and harms of available treatments for acute, non-low-back related MSK injuries.

**Methods**: We searched MEDLINE, EMBASE, CINAHL, PEDro and CENTRAL, for trials that enrolled adult patients with acute, non-low-back related MSK pain, and randomized them to any intervention directed at pain relief or a control group (active or placebo/sham). We assessed certainty of evidence using the GRADE approach. We estimated summary odds ratios (ORs) using pairwise and network meta-analysis using random effects models.

**Results**: We identified 22,919 citations, of which 181 trials were eligible for review. Moderate to high quality evidence showed that, compared to placebo, transbuccal fentanyl, topical and oral NSAIDs, and acetaminophen alone or in combination with diclofenac provided significant pain relief at 30min to 120min post-treatment, ranging from 4.1cm on a 10cm VAS (95% CI: 2.6 to 5.6) for fentanyl to 0.9cm (95% CI: 0.4 to 1.5) for acetaminophen. Oral and topical NSAIDs were associated with significant improvement in physical function, patient satisfaction, and symptom relief (moderate to high quality evidence). Moderate to high quality evidence showed that opioids alone or in combination with acetaminophen were associated with gastro-intestinal and neurologic related adverse events, while oral NSAIDs were associated with gastro-intestinal related adverse events.

**Discussion/Conclusions**: We found moderate to high quality evidence that oral and topical NSAIDs are among the most effective treatments for patients with acute, non-low-back related MSK pain, and that topical NSAIDs are the safest option.

### Realist Review of Multidisciplinary Care for Opioid Dose Reduction in Patients with Chronic Non-Cancer Pain

Abhimanyu Sud^a^^b^, Michelle L.A. Nelson^b^^c^, Alana Armas^c^, Heather Cunningham^d^, Shawn Tracy^c^, Kirk Foat^e^, Navindra Persaud^a^, Fardous Hosseiny^f^, Leyna Lowe^f^, Sylvia Hyland^g^, and Ross Upshur^a^^c^^h^

^a^Department of Family and Community Medicine, University of Toronto, Toronto, Ontario, Canada; ^b^Institute of Health Policy, Management and Evaluation, University of Toronto, Toronto, Ontario, Canada; ^c^Bridgepoint Collaboratory for Research and Innovation, Lunenfeld-Tanenbaum Research Institute, Toronto, Ontario, Canada; ^d^Gerstein Science Information Centre, University of Toronto, Toronto, Ontario, Canada; ^e^London, Ontario, Canada; ^f^Canadian Mental Health Association National, Toronto, Ontario, Canada; ^g^Institute for Safe Medication Practices Canada, Toronto, Ontario, Canada; ^h^Dalla Lana School of Public Health, University of Toronto, Toronto, Onatrio, Canada

**CONTACT** Abhimanyu Sud abhimanyu.sud@utoronto.ca

© 2019 The Author(s). Published with license by Taylor & Francis Group, LLC.

This is an Open Access article distributed under the terms of the Creative Commons Attribution License (http://creativecommons.org/licenses/by/4.0/), which permits unrestricted use, distribution, and reproduction in any medium, provided the original work is properly cited.

**Introduction/Aim**: Lowering opioid dosages in people with chronic non-cancer pain can be challenging and potentially hazardous. Recent Canadian guidelines suggest multidisciplinary care (MDC) can help with opioid tapering. However, MDC for this purpose is not well characterized. We therefore conducted a systematic realist review to understand what constitutes MDC for opioid tapering and by what mechanisms these programs operate.

**Methods**: We searched 5 academic databases (Ovid MEDLINE, PsycINFO, AMED, CINAHL Plus, and Cochrane Library) to identify studies that evaluated MDC and reported on changes in opioid doses. We also searched the grey literature, conducted iterative hand searches and consulted experts to identify the broadest possible literature. We identified 12,872 records and 96 studies were included in the final review.

**Results**: The studies spanned five decades and included 97 evaluations on 77 distinct programs from 12 different countries. Sustained dose reduction (≥ 12 months post intervention) is achieved in programs with the following key mechanisms: required opioid tapering as part of the program; included pain relief *and* behaviour change approaches; were at least 3 weeks in duration; and had group therapy and family involvement.

**Discussion/Conclusions**: The outcome of sustained opioid dose reduction in multidisciplinary care is facilitated by a small number of distinct mechanisms that are usually operationalized by specific members of an MDC team. Reconceptualizing medication taking and prescribing as behaviours are important drivers of change. Further research should focus on why these mechanisms typically operate in specialized care but not in primary care.

### Pain Rating Concordance among Youth with Sickle Cell Disease and Their Caregivers

Alexandra Gilbert^a^, Guolian Kang^b^, Hui Li^b^, Jason Hodges^c^, James Klosky^d^, Jane Hankins^c^, and Nicole M. Alberts^e^

^a^Department of Psychology, University of Mississippi, Oxford, Mississippi, USA; ^b^Department of Biostatistics, St. Jude Children’s Research Hospital, Memphis, Tennessee, USA; ^c^Department of Hematology, St. Jude Children’s Research Hospital, Memphis, Tennessee, USA; ^d^Department of Psychology, Emory University School of Medicine, Atlanta, Georgia, USA; ^e^Department of Psychology, St. Jude Children’s Research Hospital, Memphis, Tennessee, USA

**CONTACT** Alexandra Gilbert Alexandra.Gilbert@stjude.org; Nicole M. Alberts nicole.alberts@stjude.org

© 2019 St. Jude. Published with license by Taylor & Francis Group, LLC.

This is an Open Access article distributed under the terms of the Creative Commons Attribution License (http://creativecommons.org/licenses/by/4.0/), which permits unrestricted use, distribution, and reproduction in any medium, provided the original work is properly cited.

**Introduction/Aim**: Pain is often prevalent, severe, and chronic among youth with sickle cell disease (SCD). Proper assessment of SCD pain is essential to the provision of adequate pain management. Previous research examining pain rating concordance among youth with SCD and their caregivers, however, has been limited by small sample sizes, and has rarely examined the influence of child age on concordance. Therefore, we examined concordance between pain reports made by youth with SCD and their caregivers, while exploring age-related differences in the degree of correspondence across three developmental stages: school-aged children (5–7 years), preadolescents (8–12 years), and adolescents (13–18 years).

**Methods**: Participants, 386 youth with SCD and their parents, completed the Pediatric Quality of Life Inventory Sickle Cell Disease Module (PedsQL- SCD).

**Results**: Utilizing intraclass correlation coefficients (ICCs), moderate agreement was found on the Pain and Hurt (PH) scale for preadolescents and adolescents and their caregivers (ICCs = 0.6; 0.47, respectively), and on the Pain Impact (PI) scale among preadolescents and their caregivers (ICC = 0.56). Poor to fair agreement was found on all other combinations of scales and age groups. On the PH and PI scales, analysis of covariances showed that smaller correlations between youth and parent ratings tended to be observed among younger in comparison to older children (*p*s ranging from < .05 to <.001).

**Discussion/Conclusions**: Concordance of pain rating scales among youth with SCD and caregivers was moderate at best, highlighting the importance of continued research on pain assessment strategies, particularly among younger children.

### Investigating the Neural Basis for Music Modulation of Pain in the Brain and Brainstem Using Functional MRI

Jocelyn M. Powers^a^, Gabriela Ioachim^a^, and Patrick W. Stroman^a^

Centre for Neuroscience Studies, Queen’s University, Kingston, Ontario, Canada

**CONTACT** Jocelyn Powers jocelyn.powers@queensu.ca

© 2019 The Author(s). Published with license by Taylor & Francis Group, LLC.

This is an Open Access article distributed under the terms of the Creative Commons Attribution License (http://creativecommons.org/licenses/by/4.0/), which permits unrestricted use, distribution, and reproduction in any medium, provided the original work is properly cited.

**Introduction/Aim:** Music has been used to treat pain for thousands of years, and its effect has been characterized in behavioural studies and with functional magnetic resonance imaging (fMRI). The aim of this study was to investigate the neural basis of music analgesia.

**Methods:** FMRI was carried out at 3 tesla and data spanned the brain and brainstem in order to investigate the effects of music on a network of pain-related areas. Twenty healthy participants (23 ± 3 years of age) each underwent a 1-hour training session in a sham MRI lab followed by a 1.5-hour session of repeated fMRI acquisitions while a noxious heat stimulation paradigm was applied to the hand. During 6 fMRI runs participants listened to music, and in 6 runs there was no music.

**Results:** Behavioural results show that the music induced a significant decrease in pain unpleasantness scores (p < 0.006, paired t-test), but not pain intensity. Structural equation modeling (SEM) demonstrated networks of brain/brainstem regions with significant connectivity and revealed differences between runs with and without music. The results indicate that music influences pain perception via multiple brain regions which project to the thalamus which in turn provides input to the periaqueductal gray matter.

**Discussion/Conclusions:** This work will help to provide a new view of how human pain is regulated in healthy people. Moreover, it will provide a critical baseline of research for future studies into how pain processing is altered in chronic pain conditions.

### Acute Postoperative Pain Trajectories Predicted by Preoperative Psychological and Functional Outcomes in Adolescents Undergoing Spine Surgery

Don Daniel Ocay^a^, Mandy Li^b^, Diana-Luk Ye^a^, Jean A. Ouellet^c^, Gabrielle Pagé^d^, and Catherine E. Ferland^e^

^a^Department of Experimental Surgery, McGill University, Montreal, Quebec, Canada; ^b^Department of Medicine, McGill University, Montreal, Quebec, Canada; ^c^Department of Pediatric Orthopaedics, McGill University, Montreal, Quebec, Canada; ^d^Department of Anesthésiologie, Université de Montréal, Montreal, Quebec, Canada; ^e^Department of Anesthesia, McGill University, Montreal, Quebec, Canada

**CONTACT** Don Daniel Ocay don.ocay@mail.mcgill.ca

© 2019 The Author(s). Published with license by Taylor & Francis Group, LLC.

This is an Open Access article distributed under the terms of the Creative Commons Attribution License (http://creativecommons.org/licenses/by/4.0/), which permits unrestricted use, distribution, and reproduction in any medium, provided the original work is properly cited.

**Introduction/Aim**: In the adult patient population, acute pain trajectories are associated with long-term outcomes such as pain and functional disability. However, there is limited data that examines acute postoperative pain trajectories in the pediatric population. The aim of this study was to identify pain trajectories in the acute postoperative period, predictors of pain trajectory membership and associations between pain trajectories and long-term outcomes.

**Methods**: Patients with adolescent idiopathic scoliosis (AIS) and scheduled to undergo spinal fusion were assessed before surgery for their medication use, pain, physical functioning, mental health, pain catastrophizing, anxiety and pain anticipation after surgery. Average 6-hour self-reported pain intensity scores were recorded in the medical charts and extracted for the entire hospital stay (5 days). At 6 weeks and 6 months after surgery, baseline variables were reassessed. Growth mixture modeling was used to conduct acute postoperative pain trajectory analysis.

**Results**: One hundred and one patients were included in the best fitted acute pain trajectory model (AIC = 6627.9, BIC = 6732.5) including 4 classes. Pain trajectory membership was significantly predicted by preoperative pain medication use, physical functioning, back pain and pain elsewhere in the body, mental health, helplessness and trait anxiety. Acute postoperative pain trajectories were associated with physical functioning (*p* = 0.0113) and pain (*p* = 0.00039) 6 months after surgery.

**Discussion/Conclusions**: Preoperative assessment of surgical AIS patients and analyzing their progression of pain in the acute postoperative period is clinically significant to predict the long-term outcomes and develop personalized pain management.

### Evaluation of a 6 Week Interprofessional Collaborative Chronic Pain Management Program

Andrew Koscielniak^a^, Victoria Ewen^b^, Mary Donaghy^a^, Matt Schmidt^a^, and Jaye Walker^a^

^a^Chronic Pain Management Program, St. Joseph’s Care Group, Thunder Bay, Ontario, Canada; ^b^Department of Pschology, Lakehead University, Thunder Bay, Ontario, Canada

**CONTACT** Andrew Koscielniak kosciela@tbh.net

© 2019 The Author(s). Published with license by Taylor & Francis Group, LLC.

This is an Open Access article distributed under the terms of the Creative Commons Attribution License (http://creativecommons.org/licenses/by/4.0/), which permits unrestricted use, distribution, and reproduction in any medium, provided the original work is properly cited.

**Introduction/Aim**: Interprofessional collaboration (IPC) occurs when learners/practitioners, patients/clients/families and communities develop and maintain interprofessional working relationships that enable optimal health outcomes. The St. Joseph’s Care Group Chronic Pain Management program (CPMP) uses an IPC approach to help clients achieve their pain management and functional goals. The objective of this evaluation was to determine the effectiveness of an IPC approach to improving impairment associated with chronic pain.

**Methods**: Of the 155 clients attending the CPMP after November 2015 who completed an assessment, 96 clients (Mean age = 49.0, *SD *= 13.1; 64.6% female) completed measures related to their pain catastrophizing, pain interference, and kinesiophobia before and after attending the CPMP. Eighty-three clients (Mean age = 49.0, *SD* = 13.1; 67.5% female) also completed a measure of psychological functioning and 80 clients (Mean age = 50.9, *SD* = 14.1; 68.8% female) completed a fitness assessment before and after engaging in the program.

**Results**: Clients experienced reductions in pain catastrophizing, (*t*(94) = 2.26, *p* = .026), pain interference, (*t*(95) = 5.42, *p* < .001), and kinesiophobia (*t*(95) = −2.75, *p* = .007) after attending the 6 week CPMP. Clients also improved their physical fitness (e.g., 6 minute walk test, *t*(79) = 7.64, *p* < .001; chair sit to stand, *t*(79) = 7.82, *p* < .001) after attending.

**Discussion/Conclusions**: The current findings support the effectiveness of a 6 week IPC CPMP for improving impairment associated with chronic pain.

### Increased Central Sensitization in Women with Chronic Pelvic Pain and Painful Bladder Syndrome

Natasha L. Orr^a^, Kate Wahl^a^, Heather Noga^b^, Arianne Albert^b^, Kelly B. Smith^c^, and Paul J. Yong^a^

^a^Obstetrics and Gynecology, University of British Columbia, Vancouver, British Columbia, Canada; ^b^Obstetrics and Gynecology, Women’s Health Research Institute, Vancouver, British Columbia, Canada; ^c^Obstetrics and Gynecology, Vancouver General Hospital, Vancouver, British Columbia, Canada

**CONTACT** Paul Yong paul.yong@vch.ca F2-4500 Oak street, Vancouver British Columbia, Canada, V6H3N1

© 2019 The Author(s). Published with license by Taylor & Francis Group, LLC.

This is an Open Access article distributed under the terms of the Creative Commons Attribution License (http://creativecommons.org/licenses/by/4.0/), which permits unrestricted use, distribution, and reproduction in any medium, provided the original work is properly cited.

**Introduction/Aim**: A common symptom of endometriosis is chronic pelvic pain (CPP; ≥6 months; not including painful periods, sexual pain, or painful bowel movements). The objective of this study was to determine if CPP and painful bladder syndrome (PBS) were associated with central sensitization.

**Methods**: A cross-sectional study examining 256 women with endometriosis between the age of 18–50 years from a prospective patient registry (ClinicalTrials.gov#NCT02911090). Exclusion criteria: missing outcome variables and menopausal. Primary outcome: Central Sensitization Inventory scores (CSI; 0–100). Main variables of interest: 1) CPP (high [≥5], low [<5]), and 2) PBS (yes, no) using diagnostic criteria. Bivariate analyses identified variables significantly associated with the primary outcome. The cohort was divided into groups: 1) low CPP with or without PBS, 2) high CPP and no PBS, and 3) high CPP and PBS. ANOVA and post hoc testing identified differences between groups.

**Results**: The mean CSI score was 41.9 ± 18.5; 49.2% of the sample had PBS; 71.9% had a high severity of CPP. Mean CSI score was significantly associated with PBS (T = −7.40, p < .001) and severity of CPP (T = −6.14, p < .001). Group 3 had a significantly higher mean CSI score compared to the other two groups (p < .05).

**Discussion/Conclusions**: The group with high severity of CPP and PBS had the greatest CSI score, suggesting a potential role for central sensitization in this subgroup.

### Improvement in Outcomes of Parents of Youth with Chronic Pain following Intensive Pain Rehabilitation at the Alberta Children’s Hospital

Laura Rayner^a^, Nivez Rasic^b^, Jillian Vinall^b^, Allison McPeak^b^, and Melanie Noel^c^

^a^The Vi Riddell Children’s Pain & Rehabilitation Centre, Alberta Children’s Hospital, Calgary, AB, Canada; ^b^Department of Anesthesia, University of Calgary, Calgary, AB, Canada; ^c^Department of Psychology, University of Calgary, Calgary, AB, Canada

**CONTACT** Laura Rayner laura.rayner@ahs.ca

© 2019 The Author(s). Published with license by Taylor & Francis Group, LLC.

This is an Open Access article distributed under the terms of the Creative Commons Attribution License (http://creativecommons.org/licenses/by/4.0/), which permits unrestricted use, distribution, and reproduction in any medium, provided the original work is properly cited.

**Introduction/Aim**: Chronic pain is a growing epidemic with substantial impact on the individual, family and society. The Intensive Pain Rehabilitation Program (IPRP) at the Alberta Children’s Hospital (ACH) was developed to target youth with chronic pain and consequent functional disability who are not progressing in conventional outpatient pain therapies. IPRP involves participation in 3- to 6-weeks of day treatment rehabilitation, provided by a multidisciplinary team. While there is growing evidence that IPRP is effective in improving functioning for youth with chronic pain, little is known about its effectiveness for improving functioning in parents of youth in these programs. Therefore, the present study assessed parent outcomes following IPRP at ACH.

**Methods: T**wenty-four parents and their child participated in the IPRP. Measures assessing family functioning, pain catastrophizing, parent responses to child pain, pain acceptance and parent anxiety and depressive symptoms were administered to parents at baseline (before IPRP), discharge (end of IPRP) and 3-months post-IPRP. Repeated measures ANOVAs with Bonferonni correction were used to examine changes in parent outcomes between time points.

**Results: F**ollowing IPRP, parents reported significantly less pain catastrophizing (Wilks’ Lambda = 0.58, F(2,13) = 4.71, p = 0.03), less monitoring behavior (Wilks’ Lambda = 0.41, F(2,12) = 8.67, p = 0.005), and greater acceptance of their child’s pain (Wilks’ Lambda = 0.52, F(2,12) = 5.60, p = 0.02). No other significant main effects were observed.

**Discussion/Conclusions: F**ollowing IPRP, parents reported fewer behaviors that have been previously shown to contribute to the development and maintenance of chronic pain in youth. However, both parents and youth may benefit from more parent-directed interventions in IPRP.

### Parent Protective Behaviours Influence Youth Pain-Related Outcomes Following Intensive Pain Rehabilitation

Allison McPeak^a^, Nivez Rasic^a^, Jillian Vinall^a^, Laura Rayner^b^, and Melanie Noel^c^

^a^Department of Anesthesia, University of Calgary, Calgary, Alberta, Canada; ^b^The Vi Riddell Children’s Pain & Rehabilitation Centre, Alberta Children’s Hospital, Calgary, Alberta, Canada; ^c^Department of Psychology, University of Calgary, Calgary, Alberta, Canada

**CONTACT** Allison McPeak aemcpeak@ucalgary.ca

© 2019 The Author(s). Published with license by Taylor & Francis Group, LLC.

This is an Open Access article distributed under the terms of the Creative Commons Attribution License (http://creativecommons.org/licenses/by/4.0/), which permits unrestricted use, distribution, and reproduction in any medium, provided the original work is properly cited.

**Introduction/Aim**: The Intensive Pain Rehabilitation Program (IPRP) provides multidisciplinary 3-to-6-weekday-treatment rehabilitation for youth with chronic pain who are not progressing in traditional pain therapies. This program focuses on self-management skills, but also addresses the role of the parent in pain management. Parental protective behaviours, as assessed by the Adult Responses to Children’s Symptoms (ARCS) measure, have been shown to improve following cognitive-behavioral therapy (CBT) and influence child functioning. However, change in protective behaviours has not been well examined in relation to child pain acceptance or fear of pain, two factors that influence disability-related outcomes. The present study examined how changes in parent protective behaviours influence changes in child pain acceptance and fear of pain over the course of IPRP.

**Methods**: Twenty-four adolescents completed measures of chronic pain acceptance and fear of pain, and their parents completed a measure of their protective responses to their child’s pain symptoms at baseline (pre-IPRP), discharge (post-IPRP) and follow-up (3-months post-IPRP). Generalized estimating equations were used to examine change over time in parent behaviours and child outcomes.

**Results**: Greater acceptance of chronic pain (β = −0.505, p < 0.001) and lower fear of pain (β = 0.357, p = 0.017) in youth over the course of and following IPRP were associated with fewer parent protective behaviours, after accounting for child age, gender, and weeks in IPRP.

**Discussion/Conclusions**: Decreases in parental protectiveness was associated with greater child pain acceptance and less fear of pain over the course of IPRP, providing further evidence for the importance of targeting these operant parent behaviours in treatment.

### Acute Postoperative Opioid Consumption Trajectories and Long-Term Outcomes in Paediatric Patients after Spine Surgery

Mandy Li^a^, Don Daniel Ocay^b^, Alisson R. Teles^c^, Pablo M. Ingelmo^d^, Jean A. Ouellet^e^, Gabrielle Pagé^f^, and Catherine E. Ferland^d^

^a^Faculty of Medicine, McGill University, Montreal, Quebec, Canada; ^b^Department of Experimental Surgery, McGill University, Montreal, Quebec, Canada; ^c^Integrated Program in Neurosciences, McGill University, Montreal, Quebec, Canada; ^d^Department of Anesthesia, McGill University, Montreal, Quebec, Canada; ^e^Division of Orthopaedic Surgery, McGill University, Montreal, Quebec, Canada; ^f^Département d’anesthésiologie, Université de Montréal, Montreal, Quebec, Canada

**CONTACT** Mandy Li mandy.li@mail.mcgill.ca Faculty of Medicine, McGill University, Montreal, Quebec, Canada

© 2019 The Author(s). Published with license by Taylor & Francis Group, LLC.

This is an Open Access article distributed under the terms of the Creative Commons Attribution License (http://creativecommons.org/licenses/by/4.0/), which permits unrestricted use, distribution, and reproduction in any medium, provided the original work is properly cited.

**Introduction/Aim**: It is currently unknown as to whether opioid consumption throughout the acute postoperative period is associated with long-term outcomes in paediatric patients. The aims of this study were to characterize opioid consumption trajectories in the acute postoperative period, identify predictors of trajectory membership and determine associations between acute opioid consumption and long-term patient outcomes.

**Methods**: Medication use, pain and functional activity were assessed at baseline in adolescents with idiopathic scoliosis scheduled for spinal fusion surgery. Cumulative 6-hour opioid consumption was recorded for up to 5 days after surgery. At 6 months after surgery, medication use, pain and functional activity were re-evaluated. Growth mixture modelling was used to identify opioid trajectories.

**Results**: A total of 106 patients were included in the study. Mean cumulative opioid consumption 120 hours after surgery was 13.23 ± 5.20 mg/kg. The model with the best fit contained 5 acute postoperative opioid trajectories and a quadratic term (AIC = 6703.26, BIC = 6767.19). Two types of patient behaviours were identified: high opioid consumers (trajectories 4 and 5) and low opioid consumers (trajectories 1, 2 and 3). Intraoperative epimorphine dose was a predictor of trajectory membership (p = 0.0498). Opioid consumption during the acute postoperative period was not significantly associated with pain, functional activity or pain medication use 6 months after surgery.

**Discussion/Conclusions**: In paediatric patients, intraoperative epimorphine dose predicts opioid consumption in the acute postoperative period. Importantly, opioid consumption after surgery is not associated with long-term pain, functional activity or opioid and non-opioid pain medication use.

### Impact of E-Learning Modules on Self-Efficacy regarding Chronic Regional Pain Syndrome (CRPS) in Family Physicians

Vicky Fournier^a^, and Anne Marie Pinard^a^

Anesthesiology and Intensive Care Unit Department, Laval University, Québec, Canada

**CONTACT** Vicky Fournier vicky.fournier.2@ulaval.ca

© 2019 The Author(s). Published with license by Taylor & Francis Group, LLC.

This is an Open Access article distributed under the terms of the Creative Commons Attribution License (http://creativecommons.org/licenses/by/4.0/), which permits unrestricted use, distribution, and reproduction in any medium, provided the original work is properly cited.

**Introduction/Aim**: CRPS is a rare and largely unknown disease to family physician. Nevertheless, diagnosis is the key to patient recovery Continuing professional development (CPD) is a requirement for physicians and *e-learning* constitute an excellent option to promote asynchronous learning Aim of this study is to demonstrate self-efficacy improvement regarding CRPS in family physician by completing short e-learning modules, Modules’ acceptability will also be evaluated.

**Methods**: 30 family physicians answered surveys about self-efficacy regarding CRPS before (T_0_), immediately after (T_1_) and 3 months after (T_2_) completion of modules and about modules’ acceptability at T_1_. Surveys were inspired from the CPD-reaction tool and LORI (Learning Object Review Instrument) A 5-points Likert scale was used to answer each statement. Data was dichotomously analyzed: the first 3 points were associated with a negative self-efficacy feeling; the 4th and 5th points with a positive feeling. The same structure was applied for modules’ acceptability.

**Results**: Initially, self-efficacy was low. As the physician complete the module, it tends to improve. Modules were well received and therefore acceptable. Our analysis is currently in progress. T_2_ questionnaires will be completed and analysed upon presentation.

**Discussion/Conclusions**: The modules were generally appreciated.

Short e-learning modules on rare but serious chronic pain conditions such as CRPS might be a simple and effective method to improve self-efficacy and in turn, patient care.

**Disclosure statement**

No potential conflict of interest was reported by the authors.

References1.
Harden
RN, Oaklander
AL, Burton
AW, Perez
RS, Richardson
K, Swan
M, Bruehl
S. Complex regional pain syndrome: practical diagnostic and treatment guidelines. Pain med. 2013;14(2):180–229. doi:
10.1111/pme.12033.233319502.
Ellaway
R, Masters
K. AMEE guide 32: e-learning in medical education Part 1: learning, teaching and assessment. Med Teach. 2008;30(5):455–73. doi:
10.1080/01421590802108331.185761853.
Légaré
F, Borduas
F, Freitas
A, Jacques
A, Godin
G, Luconi
F, Grimshaw
J. Development of a simple 12-item theory-based instrument to assess the impact of continuing professional development on clinical behavioral intentions. PLoS One. 2014;9(3):e91013. doi:
10.1371/journal.pone.0091013.24643173PMC39583454.
Leacock
TL, Nesbit
JC. A framework for evaluating the quality of multimedia learning resources. J Educ Technol Soc. 2007;10(2):44–59.

### Exploring the Relationship between Pain Catastrophizing, Pain-Related Fear and Trunk Biomechanics in Chronic Low Back Pain: A Scoping Review

Patrick Ippersiel^a^, and Shawn M. Robbins 0000-0002-8108-0561^a^

School of Physical and Occupational Therapy, McGill University, Montreal, Quebec, Canada

**CONTACT** Patrick Ippersiel Patrick.ippersiel@mail.mcgill.ca

© 2019 The Author(s). Published with license by Taylor & Francis Group, LLC.

This is an Open Access article distributed under the terms of the Creative Commons Attribution License (http://creativecommons.org/licenses/by/4.0/), which permits unrestricted use, distribution, and reproduction in any medium, provided the original work is properly cited.

**Introduction/Aim**: Pain changes the way people move. In chronic low back pain (CLBP), these changes are maintained in the long-term and contribute to ongoing pain and disability. Psychosocial factors have been identified as predictors of future disability and poor treatment outcomes in CLBP, but how such factors relate to movement is unclear. The purpose of this scoping review is to examine the relationship of pain catastrophizing and pain-related fear with trunk biomechanics, among adults with CLBP performing functional tasks.

**Methods**: Scoping review methodology was conducted based on the Arksey and O’Malley (2005) framework. Literature searches were performed in MEDLINE, PubMed, EMBASE, PsycINFO and CINAHL. Keywords and MeSH terms were included to capture relevant articles relating to pain-related fear, pain catastrophizing, and trunk biomechanics, in individuals with CLBP .

**Results**: Twenty-three studies met selection criteria, with a total of 26 biomechanical outcomes. 12 studies reported an association between pain catastrophizing and/or pain-related fear with trunk biomechanics during: the flexion-relaxation phenomenon (n = 4), gait cycle (n = 3), in response to perturbations (n = 3), and spinal mobility (n = 2). The remaining studies were classified as either inconclusive (n = 5/26) or demonstrating no association (n = 9/26).

**Discussion/Conclusions**: These findings might suggest an association between pain-related fear and/or pain catastrophizing with: (i) greater trunk activity, (ii) reduced spinal mobility and (iii) reduced trunk reflexes, which may indicate a form of protective guarding or bracing. Due to lack of study appraisal, our inferences should be interpreted with caution. A systematic review on this subject would be of great value to the field.

### Trajectory Modelling Techniques Useful to Pain Research: A Narrative Comparison of Approaches

Hermine Lore Nguena Nguefack^a^, M. Gabrielle Pagé^b^, Manon Choinière 0000-0001-9593-8883^b^, and Anaïs Lacasse 0000-0002-3992-5145^a^

^a^Département des sciences de la santé, Université du Québec en Abitibi-Témiscamingue (UQAT), Rouyn-Noranda, Québec, Canada; ^b^Centre de recherche du Centre hospitalier de l’Université de Montréal (CRCHUM); Département d’anesthésiologie et de médecine de la douleur, Faculté de médecine, Université de Montréal, Montréal, Québec, Canada

**CONTACT** Anaïs Lacasse anais.lacasse@uqat.ca

© 2019 The Author(s). Published with license by Taylor & Francis Group, LLC.

This is an Open Access article distributed under the terms of the Creative Commons Attribution-NonCommercial License (http://creativecommons.org/licenses/by-nc/4.0/), which permits unrestricted non-commercial use, distribution, and reproduction in any medium, provided the original work is properly cited.

**Introduction/Aim**: Trajectory modelling approaches have been developed to determine subgroups within a given population and are increasingly used to better understand pain outcomes. With the purpose of enabling pain researchers to choose the technique that best suits their research questions, the objective of this narrative review was to explore various trajectory modelling approaches used in health research and discuss about their applications.

**Methods**: To establish and identify relevant peer-reviewed literature, PubMed, Psych-Info and Google Scholar were used with no date of restriction. Approaches were compared in terms of definitions, rationale of use, assumptions, concrete clinical applications, and availability of statistical software programs.

**Results**: Three common approaches of trajectory modelling were identified: Latent class modelling (LCM) approaches (e.g. Growth mixture modelling-GMM, Group-based trajectory modelling-GBTM, Latent class analysis-LCA), cluster analysis (CA) and sequence analysis (SA).

LCM are based on a probabilistic modelling approach with a finite mixture distribution that describes an observed life course sequence of categorical values as resulting from the conditional probabilities that define membership of a latent class. LCM provides the contribution of every observed variable on the definition of classes.

CA is a tool to explore groups within a data set. When variables under study are continuous, CA is sometimes called latent profile analysis. When the variables are categorical, CA is sometimes called LCA.

SA is a fully nonparametric approach based on algorithmic, approaches aimed at making use of measures of distance between individual trajectories.

**Discussion/Conclusions**: Depending on the research question and the available data particularities, one or another of these approaches can be used for trajectory modelling.

### Understanding Pain Management Information Needs in Caregivers of Children with Arthritis

Yvonne Brandelli^a^, Christine Chambers^b^, Perri Tutelman^a^, Jennifer Stinson^c^, Adam Huber^d^, and Jennifer Wilson^e^

^a^Psychology and Neuroscience, Dalhousie University, Halifax, Nova Scotia, Canada; ^b^Psychology and Neuroscience & Pediatrics, Dalhousie University & IWK Health Centre, Halifax, Nova Scotia, Canada; ^c^Lawrence S. Bloomberg Faculty of Nursing, University of Toronto and Research Institute at The Hospital for Sick Children, Toronto, Ontario, Canada; ^d^Pediatrics & Rheumatology, Dalhousie University & IWK Health Centre, Halifax, Nova Scotia, Canada; ^e^Cassie and Friends, Vancouver, British Columbia, Canada

**CONTACT** Yvonne Brandelli Yvonne.Brandelli@dal.ca

© 2019 The Author(s). Published with license by Taylor & Francis Group, LLC.

This is an Open Access article distributed under the terms of the Creative Commons Attribution License (http://creativecommons.org/licenses/by/4.0/), which permits unrestricted use, distribution, and reproduction in any medium, provided the original work is properly cited.

**Introduction/Aim**: Juvenile Idiopathic Arthritis (JIA) affects approximately 24,000 children throughout Canada, many of whom report pain as the predominant symptom. Caregivers play an important role in managing JIA-related pain; not only are they the primary support, many are also tasked with administering potentially painful treatments at home. This study explored caregivers’ confidence and information needs in managing their child’s pain.

**Methods**: Recruitment took place worldwide by engaging partner organizations and sharing across online and social media platforms. 216 caregivers of children with JIA aged 0–17 participated in an online survey, completing questions about their child’s arthritis and pain, and their own information needs. Participants were predominantly mothers (96%) residing in North America (77%).

**Results**: Caregivers reported lacking confidence in alleviating their child’s arthritis-related pain (*M* = 4.11, *SD *= 2.60, range = 0–10). Although all participants reported wanting some information, regressions demonstrate that lower caregiver confidence related to a need for more information [*t* = −2.91, *β *= −0.34 (−0.57, −0.12)]. The most frequently identified needs included managing emotions related to painful experiences (60%) and differentiating arthritis pain from normal pain (56%). Caregivers strongly valued developing partnerships between researchers and parent organizations wherein evidence-based information related to arthritis pain could be shared (*M* = 4.25, *SD *= 1.05, range = 1–5).

**Discussion/Conclusions**: As caregivers of children with JIA are tasked with helping to manage their child’s pain, it is important that they have the necessary tools to do so. This research demonstrates that although parents’ lack confidence in pain management, they are keen to learn more. Implications will be discussed for knowledge translation within this community.

### Intensive Care Pupillometry as A Predictor of Opioid Consumption following Intubation: A Preliminary Study in Moderate-To-Severe Traumatic Brain Injury

Chloé Martineau, Sabrina Bouferguene, Léa Proulx-Bégin, Francis Bernard, and Caroline Arbour

**CONTACT** Chloé Martineau chloe.martineau-lessard@umontreal.ca

© 2019 The Author(s). Published with license by Taylor & Francis Group, LLC.

This is an Open Access article distributed under the terms of the Creative Commons Attribution License (http://creativecommons.org/licenses/by/4.0/), which permits unrestricted use, distribution, and reproduction in any medium, provided the original work is properly cited.

**Introduction/Aim**: Opioid use following intubation is associated to poorer outcomes after moderate-to-severe traumatic brain injury (TBI), but is difficult to predict. Emerging evidence in anesthesiology suggest the pupillary dilation reflex (PDR) can provide some insights about opioids consumption post-intubation. Yet, the utility of this metric in critically ill TBI patients is unknown. We investigated the preliminary utility of PDR during the first three days of ICU-admission to predict opioid consumption following intubation in the context of TBI.

**Methods**: Pupil measurements were performed once a day at fixed times and on both eyes during the first three days of ICU-admission using video-pupillometry. PDR was elicited by pressure algometry applied to the fingernails. Cumulative doses of opioids received within 72h post-intubation were collected.

**Results**: Twenty adults (15 male, 18–64 yr) with moderate or severe TBI participated. Overall, n = 9 (45%) TBI patients showed low pupillary reactivity (PDR <5%) and n = 11 (55%) TBI patients showed high reactivity (PDR ≥5%) during fingernail pressure. Subgroups were found similar in terms of TBI presentation and pharmacological management in the first three days of ICU-admission. Cumulative doses of Hydromorphone administered to TBI patients with low versus high pupillary reactivity within 72h post-intubation were as follow: 4 ± 3mg vs. 15 ± 8mg (t = 3.20; p = 0.03). Correcting for age and gender, pupil variation in critically ill TBI was found a significant predictor of opioid consumption post-intubation (adjusted R square = 87.5).^1^In the present study we just report the results of 4 eligible studies published in English. In the updated study, we have also included 52 articles published in Chinese with the assistance of our Chines colleagues. We will report the results of our new analyses in the near future.

**Discussion/Conclusions**: Our results show for the first time that ICU pupillometry can provide accurate insights on TBI patients opioid requirements following intubation.

### Acupuncture for the Management of Chronic Diabetic Peripheral Neuropathy: A Systematic Review and Meta-Analysis of Randomized Trials

Ngai Chow 0000-0002-1800-0362^a^, Mahmood AminiLari^a^, Rachel Couban^b^, Li Wang 0000-0003-1585-8846^b^, and Jason W. Busse 0000-0002-0178-8712^b^

^a^Department of Health Research Methods, Evidence, and Impact, McMaster University, Hamilton, Ontario, Canada; ^b^Department of Anesthesia, McMaster University, Hamilton, Ontario, Canada

**CONTACT** Mahmood AminiLari aminilam@mcmaster.ca

© 2019 The Author(s). Published with license by Taylor & Francis Group, LLC.

This is an Open Access article distributed under the terms of the Creative Commons Attribution License (http://creativecommons.org/licenses/by/4.0/), which permits unrestricted use, distribution, and reproduction in any medium, provided the original work is properly cited.

**Introduction/Aim**: Peripheral neuropathy is a common cause of chronic pain among patients with diabetes, and acupuncture has been suggested as a therapeutic option. To determine the effectiveness of acupuncture for chronic diabetic peripheral neuropathy (DPN).

**Methods**: We searched MEDLINE, EMBASE, CENTRAL, AMED, CINAHL, PsychINFO, trial registries, and reference lists of relevant articles up to February 2017. Pairs of reviewers, independently and in duplicate, screened articles for inclusion, assessed risk of bias and extracted data. We conducted meta-analyses when possible and used the GRADE approach to assess the quality of evidence.

**Results**: Among 4443 potentially eligible studies 4 with 244 patients proved eligible to be included for review.^1^ Overall all four studies were at high risk of bias. Compared to sham acupuncture, we found very low quality evidence that acupuncture reduces pain (weighted mean difference [WMD] −1.95cm on a 10cm visual analogue scale, 95% CI −3.27 to −0.64; minimally important difference [MID] 1cm; risk difference (RD) for achieving the MID 52%; 95% CI 19% to 63%) and improves physical functioning (WMD 3.68 points on the short form-36 [SF-36] physical component summary score; 95% CI 1.66 to 5.70; MID is 5-points; RD for achieving the MID 8%, 95% CI 3% to 18%), but does not affect emotional functioning (WMD 1.26 points on the SF-36 mental component summary score, 95% CI: −1.13 to 3.66).

**Conclusions**: Very low-quality evidence suggests that acupuncture reduces pain and improves physical functioning in patients with chronic DPN but does not affect emotional functioning.

### Chronic Pain Experience and Management among People Who Use Illicit Drugs: A Qualitative Study in Montreal (QC)

Lise Dassieu^a^^b^, Jean-Luc Kaboré^b^^c^, Manon Choinière 0000-0001-9593-8883^b^^d^, and Élise Roy^a^

^a^ Université de Sherbrooke, Faculté de Médecine et des Sciences de la Santé, Département des Sciences de la Santé Communautaire, Chaire de Recherche en toxicomanie, Longueuil, Québec, Canada; ^b^Centre de Recherche du Centre hospitalier de l’Université de Montréal, Carrefour de l’innovation, Montréal, Québec, Canada; ^c^Université de Montréal, Département de Pharmacologie, Faculté de Médecine, Montréal, Québec, Canada; ^d^Université de Montréal, Département d’Anesthésiologie et de Médecine de la Douleur, Faculté de Médecine, Montréal, Québec, Canada

**CONTACT** Lise Dassieu lise.dassieu@umontreal.ca

© 2019 The Author(s). Published with license by Taylor & Francis Group, LLC.

This is an Open Access article distributed under the terms of the Creative Commons Attribution License (http://creativecommons.org/licenses/by/4.0/), which permits unrestricted use, distribution, and reproduction in any medium, provided the original work is properly cited.

**Introduction/Aim**: Chronic non-cancer pain (CNCP) is both highly prevalent and undertreated among people who use illicit drugs (PWUD). To address the current opioid crisis, several health authorities have produced guidelines recommending not to prescribe opioid painkillers to patients with substance abuse risk factors. This could jeopardize pain relief for PWUD suffering from CNCP. This study aims to describe: (1) PWUD’s daily CNCP experiences; (2) barriers of access to adequate CNCP management for PWUD in the opioid crisis context; (3) PWUD’s stated needs for improving their CNCP and healthcare experiences.

**Methods**: This was a qualitative study. In-depth semi-structured interviews were conducted with 25 PWUD (illicit opioids and/or cocaine) suffering from CNCP (≥ 3 months) recruited in downtown Montreal (2017/07 to 2018/05). Interviews were analyzed using Grounded Theory method.

**Results**: Participants faced multiple health problems (e.g. HIV, hepatitis C) and social issues (e.g. homelessness, precarious employment) concomitantly with CNCP and substance use. These problems contributed to heighten their pain intensity. Physicians’ increased defiance against PWUD in the opioid crisis context led to major barriers for participants’ pain management. Participants experienced stigma and discrimination in the healthcare system. They wished they could access non-pharmacological therapies (e.g. physiotherapy) as alternatives to opioids, but these therapies were unaffordable. Some participants reported self-medicating their CNCP with street drugs when no other solution was available.

**Discussion/Conclusions**: PWUD suffering from CNCP are a population with several comorbidities and high health care needs. Public policies should be revisited to improve access to appropriate CNCP management for this population.

### CX3CR1 Expression Is Required for the Development of Pain like Behavior in a Mouse Model of Autoimmune Peripheral Neuropathy

Oladayo Oladiran^a^, Mu Yang^a^, Xiang Qun Shi^a^, Sylvie Fournier^b^, and Ji Zhang^a^

^a^The Alan Edwards Centre for Research on Pain, McGill University, Montreal, Quebec, Canada; ^b^Microbiology and Immunology, McGill University, Montreal, Quebec, Canada

**CONTACT** Dr. Ji Zhang ji.zhang@mcgill.ca McGill University, The Alan Edwards Centre for Research on Pain, Montreal, Canada.

© 2019 The Author(s). Published with license by Taylor & Francis Group, LLC.

This is an Open Access article distributed under the terms of the Creative Commons Attribution License (http://creativecommons.org/licenses/by/4.0/), which permits unrestricted use, distribution, and reproduction in any medium, provided the original work is properly cited.

**Introduction/Aim**: Guillain-Barré syndrome and chronic inflammatory demyelinating polyradiculoneuropathy are prototypic autoimmune peripheral neuropathy (APN) which represent a serious neurological emergency. Although current treatment options have proven effective, many patients still present with a severe disease course, pain and residual deficits. We have reported that B7.2 transgenic L31 mice spontaneously develop inflammatory peripheral neuropathy. We also showed that an injury to peripheral nerve in L31 mice facilitates the development of APN. Both effector/memory CD8 T cells and B7.2+ macrophages are required for disease initiation. Here, we highlighted the importance of CX3CR1 expression in disease pathogenesis and in the development of pain like behavior in L31 mice.

**Methods**: L31 mice were crossed with CX3CR1KO mice, and the effect on macrophage phagocytic ability, activated CD8 T cells function and pathology were assessed.

**Results**: CX3CR1 expression was strongly upregulated in sciatic nerve of symptomatic L31 mice which correlated with increased phagocytic ability. Enhanced phagocytosis in L31 mice was impeded in L31/CX3CR1^−/-^ mice. Also, injury failed to trigger APN in L31 mice deficient of CX3CR1 expression. APN associated sensory deficits positively correlated with the amount of CX3CR1 expression. In addition, CX3CR1 deficiency led to a high number of dying monocytes, macrophages and activated CD8 T cells suggesting that CX3CR1 signaling may be crucial for their survival. Lastly, sciatic nerve histology showed no myelin and axon damage in L31/CX3CR1^−/-^ mice.

**Discussion/Conclusions**: These data suggest that CX3CR1 expression are critical for the development of APN in L31 mice. CX3CR1 expressing macrophages contribute to disease pathogenesis via enhanced antigen phagocytosis and presentation.

### Coordinated Networks in the Human Brainstem and Spinal Cord during the Expectation of Pain

Gabriela Ioachim^a^, Jocelyn M. Powers^a^, and Patrick W. Stroman^a^

Centre for Neuroscience Studies, Queen’s University, Kingston, Ontario, Canada

**CONTACT** Gabriela Ioachim ioachim.gabriela@queensu.ca

© 2019 The Author(s). Published with license by Taylor & Francis Group, LLC.

This is an Open Access article distributed under the terms of the Creative Commons Attribution-NonCommercial License (http://creativecommons.org/licenses/by-nc/4.0/), which permits unrestricted non-commercial use, distribution, and reproduction in any medium, provided the original work is properly cited.

**Introduction/Aim**: Spontaneous variations in activity of brainstem (BS) and spinal cord (SC) regions may arise from a number of functions such as autonomic regulation, sensory, and motor functions. Recent evidence suggests that changes in a person’s cognitive/emotional state are linked to changes in identified BS and SC resting-state networks, indicating that these networks likely play a role in the integration of homeostatic autonomic functions. The aim of this study was to investigate how these networks change when participants are specifically expecting pain.

**Methods**: Previously, data were obtained from the cervical SC and brainstem in 17 healthy participants during a stimulation paradigm that involved a predictable noxious heat stimulus. Blood oxygenation-level dependent (BOLD) fMRI data were obtained at 3 Tesla, with T2-weighted single-shot fast spin-echo imaging. For the current study we investigated functional connectivity in the entire 3D region with structural equation modelling (SEM) during the first two minutes of each run (baseline period, and after participants were told whether to expect a painful stimulus).

**Results**: SEM results showed extensive connectivity within and across BS and SC regions both when participants were expecting pain, and when they were expecting no pain. Furthermore, significant differences in connectivity between regions of the BS and SC were also identified across study conditions.

**Discussion/Conclusions**: The results indicate that connectivity across BS/SC networks is influenced by the expectation of pain in specific ways. The known functions of the regions involved support the conclusion that these networks likely serve to integrate autonomic regulation functions with pain processing.

### Development and Validation of a Pain Competence Assessment Tool (PCAT) Based on the IASP Core Competencies for Pain Management

Samah Hassan^a^, Andrea Furlan^b^, Bonnie Stevens^c^, Judy Watt-Watson^c^, Sharon Switzer-Mcintyre^d^, and John Flannery^b^

^a^Institute of Medical Science, Faculty of Medicine, University of Toronto, Toronto, ON; ^b^Toronto Rehabilitation Institute, University Health Network, Toronto, ON, Canada; ^c^Lawrence S. Bloomberg Faculty of Nursing, University of Toronto, Toronto, ON, Canada; ^d^Department of Physical Therapy, Faculty of Medicine, University of Toronto, Toronto, ON, Canada

**CONTACT** Samah Hassan samah.hassam@uhn.ca

© 2019 The Author(s). Published with license by Taylor & Francis Group, LLC.

This is an Open Access article distributed under the terms of the Creative Commons Attribution License (http://creativecommons.org/licenses/by/4.0/), which permits unrestricted use, distribution, and reproduction in any medium, provided the original work is properly cited.

Several interprofessional education programs have been developed to enhance primary care providers’ (PCPs) competency in pain management. This project aims to develop a valid and reliable competency-based assessment tool, suitable for all professions, to evaluate the impact of these programs.

To develop a new pain competence assessment tool (PCAT), six clinical vignettes were created. Each vignette is followed by questions addressing one or more competencies derived from the core pain competencies developed by Fishman et al. in 2013. Experts in pain management representing different professions were asked to review and rate the appropriateness of the PCAT questions to confirm face validity through a modified Delphi study. The selected PCAT questions were mapped against the core competencies to ensure content validity. A pilot sample of PCPs was then asked to answer the PCAT through a cognitive interview study to assess feasibility. Currently, the PCAT was sent to a large sample of PCPs to assess its reliability through a cross-sectional study. The PCAT was also sent to healthcare prelicensure students to test for construct validity.

Based on the Delphi results, nineteen questions were rated as appropriate confirming its face and content validity. The PCAT also showed adequate feasibility based on the cognitive interview study. Psychometric testing is still in process.

Future implementation of a valid and reliable tool is necessary to reduce measurement error and produce results with high degree of credibility.

**Introduction/Aim**: Several interprofessional education programs have been developed to enhance primary care providers’ (PCPs) competency in pain management. This project aims to develop a valid and reliable competency-based assessment tool, suitable for all professions, to evaluate the impact of these programs.

**Methods**: To develop a new pain competence assessment tool (PCAT), six clinical vignettes were created. Each vignette is followed by questions addressing one or more competencies derived from the core pain competencies developed by Fishman et al. in 2013. Experts in pain management representing different professions were asked to review and rate the appropriateness of the PCAT questions to confirm face validity through a modified Delphi study. The selected PCAT questions were mapped against the core competencies to ensure content validity. A pilot sample of PCPs was then asked to answer the PCAT through a cognitive interview study to assess feasibility. Currently, the PCAT was sent to a large sample of PCPs to assess its reliability through a cross-sectional study. The PCAT was also sent to healthcare prelicensure students to test for construct validity.

**Results**: Based on the Delphi results, nineteen questions were rated as appropriate confirming its face and content validity. The PCAT also showed adequate feasibility based on the cognitive interview study. Psychometric testing is still in process.

**Discussion/Conclusions**: Future implementation of a valid and reliable tool is necessary to reduce measurement error and produce results with high degree of credibility.

### Isolating Brain Regions Implicated in the Affective Components of Neuropathic Pain

Natalia I. Lopez^a^, Scott Holmes^a^, Nadia Barakat^a^, Alyssa Lebel^a^, Laura Simons^b^, and David Borsook^a^

^a^Anesthesiology, Perioperative & Pain Medicine, Boston Children’s Hospital, Boston, MA, USA; ^b^Anesthesiology, Perioperative & Pain Medicine, Stanford University School of Medicine, Palo Alto, CA, USA

**CONTACT** Natalia I. Lopez natalia.lopez@childrens.harvard.edu

© 2019 The Author(s). Published with license by Taylor & Francis Group, LLC.

This is an Open Access article distributed under the terms of the Creative Commons Attribution License (http://creativecommons.org/licenses/by/4.0/), which permits unrestricted use, distribution, and reproduction in any medium, provided the original work is properly cited.

**Introduction/Aim**: The psychosocial factors implicated in neuropathic pain are profound. Pain symptoms can lead to prolonged manifestations of affective and other psychological disorders (e.g., negative mood; avoidance behaviors). In this investigation, we sought to (1) evaluate the extent to which psychological factors are elevated in an acute neuropathic pain cohort, and (2) evaluate brain changes implicated in the affective components of acute neuropathic pain.

**Methods**: Participants included ankle sprain patients with acute neuropathic pain (n = 24, Age = 16.1 years, SD = 4.1), and age- and gender-matched healthy controls (n = 12, Age = 16.1, SD = 2.95). Participants were evaluated using self-report questionnaires of psychological and pain-related symptoms: Multidimensional Anxiety Scale for Children-MASC; Children’s Depression Inventory-CDI; Fear of Pain Questionnaire-FOPQ, Functional Disability Inventory-FDI, Pediatric Pain Screening Tool-PPST, and Pain Catastrophizing Scale-PCS. Cortical thickness and sub-cortical volumes were extracted from Freesurfer.

**Results**: The neuropathic pain cohort exhibited elevated scores on the CDI, FOPQ, FDI, PPST, and PCS relative to controls (p < 0.05). K-means clustering was used to create groups within the patient cohort with lower vs. higher psychological questionnaire scores. In patients with elevated questionnaire scores, greater cortical thickness was found in regions implicated in pain processing, including the orbitofrontal gyrus and caudal anterior cingulate cortex compared to patients with lower scores (p < 0.01).

**Discussion/Conclusions**: Neuropathic pain implicates many brain regions. Here, we demonstrated that persons with acute injury-induced neuropathic pain and greater levels of psychological manifestations display increased cortical thickness in regions involved in processing pain and its psychological components. Findings inform the diversity of changes observed in neuropathic pain patients.

### Promoting an Interprofessional Approach to Chronic Pain Management in Primary Care Using Project ECHO

Samah Hassan^a^, Leslie Carlin^b^, Jwane Zhao^c^, Paul Taenzer^d^, and Andrea D. Furlan^e^

^a^Institute of Medical Science, University of Toronto; ^b^Department of Physical Therapy, Faculty of Medicine, University of Toronto, Toronto, Ontario, Canada; ^c^Outpatient Services -Project ECHO, Toronto Rehabilitation Institute, University Health Network, Toronto, Ontario, Canada; ^d^Department of Physical Medicine and Rehabilitation, Queen’s University; ^e^Toronto Rehabilitation Institute, University Health Network, Toronto, Ontario, Canada

**CONTACT** Samah Hassan samah.hassam@uhn.ca

© 2019 The Author(s). Published with license by Taylor & Francis Group, LLC.

This is an Open Access article distributed under the terms of the Creative Commons Attribution License (http://creativecommons.org/licenses/by/4.0/), which permits unrestricted use, distribution, and reproduction in any medium, provided the original work is properly cited.

Chronic pain is a complex multidimensional condition that requires management with multiprofessional expertise. Healthcare professional training programs tend to adhere to curricula within their own discipline with very few interactions with other professions. Project ECHO (Extension for Community Healthcare Outcomes) is a model for interprofessional education, using tele-mentoring, case-base discussions and didactics. The goals are to improve competency and confidence in managing complex cases in primary care. This qualitative study was conducted among primary care providers from various disciplines who participated in Project ECHO Ontario Chronic Pain/Opioid stewardship. The results show that participating in ECHO resulted in personal and professional benefit, and increased understanding about their own roles and limitations, as well as other healthcare professionals’ roles. The participants described changes in their attitudes toward patients with chronic pain, and their colleagues from other professions. Non-physician participants were more likely to approach physicians to discuss their assessment and diagnosis as well as medications prescriptions. The interprofessional nature of the program was seen as positive, contributing to a modest increase in collaboration between healthcare professional groups. These results show that Project ECHO is successful in enhancing interprofessional care in primary care settings to manage complex cases of chronic pain.

**Introduction/Aim**: Chronic pain is a complex multidimensional condition that requires management with multiprofessional expertise. Healthcare professional training programs tend to adhere to curricula within their own discipline with very few interactions with other professions. Project ECHO (Extension for Community Healthcare Outcomes) is a model for interprofessional education, using tele-mentoring, case-base discussions and didactics. The goals are to improve competency and confidence in managing complex cases in primary care.

**Methods**: This qualitative study was conducted among primary care providers from various disciplines who participated in Project ECHO Ontario Chronic Pain/Opioid stewardship.

**Results**: The results show that participating in ECHO resulted in personal and professional benefit, and increased understanding about their own roles and limitations, as well as other healthcare professionals’ roles. The participants described changes in their attitudes toward patients with chronic pain, and their colleagues from other professions. Non-physician participants were more likely to approach physicians to discuss their assessment and diagnosis as well as medications prescriptions.

**Discussion/Conclusions**: The interprofessional nature of the program was seen as positive, contributing to a modest increase in collaboration between healthcare professional groups. These results show that Project ECHO is successful in enhancing interprofessional care in primary care settings to manage complex cases of chronic pain.

### Child and Parent Predictors of Healthcare Utilization Amongst Children and Adolescents with Chronic Pain

Kathryn A. Birnie^a^, Nivez Rasic^b^, and Melanie Noel^c^

^a^Vi Riddell Children’s Pain and Rehabilitation Centre, Alberta Children’s Hospital, Calgary, AB, Canada; ^b^Anesthesiology and Vi Riddell Children’s Pain and Rehabilitation Centre, University of Calgary and Alberta Children’s Hospital, Calgary, AB, Canada; ^c^Psychology, University of Calgary, Calgary, AB, Canada

**CONTACT** Kathryn A. Birnie katie.birnie@ahs.ca

© 2019 The Author(s). Published with license by Taylor & Francis Group, LLC.

This is an Open Access article distributed under the terms of the Creative Commons Attribution License (http://creativecommons.org/licenses/by/4.0/), which permits unrestricted use, distribution, and reproduction in any medium, provided the original work is properly cited.

**Introduction/Aim**: Pediatric chronic pain has a high economic burden. It is unknown what role parent factors have on healthcare utilization for pediatric chronic pain. Our aim was to identify child and parent factors that predict greater healthcare visits for pediatric chronic pain.

**Methods**: 111 8- to 17-year-olds (*M* = 13.28; *SD *= 2.73; 67% female) and caregivers (89% female) attending an outpatient multidisciplinary pediatric chronic pain program. Children self-reported pain catastrophizing (PCS-C), and mobility, anxiety, depression, fatigue, and pain interference (PROMIS). Parents self-reported parenting responses to their child’s pain (ARCS), pain catastrophizing (PCS-P), and their own chronic pain, physical function, anxiety, depression, fatigue, sleep, ability to participate in social roles, and pain interference (PROMIS). Healthcare utilization was parent-reported child visits to general practitioners, specialists, non-physician providers, emergency, and/or inpatient stays over the past 3 months due to pain. A linear regression assessed child (Step 1) and parent factors (Step 2) as predictors of healthcare utilization.

**Results**: Number of pain-related healthcare visits over the past 3 months was 0 to 46 (*M* = 7.82; *SD *= 8.00). Healthcare utilization was significantly predicted by poorer child mobility (ß = .258; *p* < .05) and fatigue (ß = .362; *p* < .01; Step 1: R = .523; R^2^ = .273; F(10,100) = 3.76; *p* < .01). Parent factors did not add significantly overall (Step 2: R^2^ = .436; F(14,86) = 1.77; *p* = .06), although greater protective (ß = .299; *p* < .01) and less monitoring (ß = −.341; *p* < .05) behaviours, and greater ability to participate in social roles (ß = .258; *p* < .05) were significant predictors.

**Discussion/Conclusions**: Greater healthcare utilization for pediatric chronic pain was primarily driven by child symptoms, with additional relations to parenting responses.

### Assessment of a Chronic Pain Clinic’s Compliance with Guidelines on Intrathecal Therapy for the Management of Pain

Monakshi Sawhney^a^, Katie Root-Clarke^b^, Elizabeth Brown^b^, Scott Duggan^b^, and Mary Anne Good^b^

^a^School of Nursing, Queen’s University, Kingston, Ontario, Canada; ^b^Chronic Pain Clinic, Kingston Health Science Center – Hotel Dieu Site, Kingston, Ontario, Canada

**CONTACT** Monakshi Sawhney mona.sawhney@queensu.ca

© 2019 The Author(s). Published with license by Taylor & Francis Group, LLC.

This is an Open Access article distributed under the terms of the Creative Commons Attribution License (http://creativecommons.org/licenses/by/4.0/), which permits unrestricted use, distribution, and reproduction in any medium, provided the original work is properly cited.

**Introduction/Aim**: Intrathecal therapy (IT) is accepted as an effective way of treating chronic or cancer pain. The Polyanalgesic Consensus Conference (PACC, 2017) guidelines provide recommendations for: patient selection; assessment and psychological considerations; medication selection and starting doses; and educational requirements for implanting and managing IT. This study examined the PACC recommendations regarding IT delivery and evaluated if these recommendations are being followed in a tertiary chronic pain setting.

**Method**: This study utilized a retrospective chart audit. A checklist was created using the PACC guideline consensus points to assess for compliance with the recommendations.

**Results**: Charts from patients (n = 8 eligible; n = 7 with consent) were reviewed in the clinic. The majority of PACC recommendations were met. Patients had a diagnosis of either palliative cancer pain (n = 2) or a chronic non-cancer pain (n = 5) including failed back syndrome (n = 4), and radicular extremity pain (n = 1). There were 5 different drug combinations administered to patients: morphine + bupivacaine (n = 2); hydromorphone (n = 2); morphine (n = 1); baclofen + morphine (n = 1); baclofen + bupivacaine (n = 1). Patients’ original opioids were weaned after the 1^st^ year of IT. Psychological screening was only conducted for the cancer patients. All health care professionals who refilled pumps had the appropriate training and all programming was conducted using an independent double check.

**Discussion/Conclusions**: Chronic non-cancer pain was the main reason for IT delivery at our clinic. Although the majority of PACC recommendations were met there is room for improvement, for example psychological screening is not routinely done but should be considered for current and future patients.

### Evaluating the Novel Added Value of Neurophysiological Pain Sensitivity within the Fear-Avoidance Model of Pain

Zakir Uddin^a^, Arthur Woznowski-Vu^a^, Daniel Flegg^a^, Andrea Aternali^b^, Rebekah Wickens^c^, and Timothy H. Wideman^a^

^a^School of Physical and Occupational Therapy, McGill University, Montreal, Quebec, Canada; ^b^Psychology, McGill University, Montreal, Quebec, Canada; ^c^School of Physical and Occupational Therapy, Constance Lethbridge Rehabilitation Centre/McGill University, Montreal, Quebec, Canada

**CONTACT** Zakir Uddin zakir.uddin@mail.mcgill.ca

© 2019 The Author(s). Published with license by Taylor & Francis Group, LLC.

This is an Open Access article distributed under the terms of the Creative Commons Attribution License (http://creativecommons.org/licenses/by/4.0/), which permits unrestricted use, distribution, and reproduction in any medium, provided the original work is properly cited.

**Introduction/Aim**: The Fear-Avoidance Model (FAM) is a leading theoretical paradigm for explaining persistent pain following musculoskeletal injury. The model suggests that as injuries heal, pain-related outcomes are increasingly determined by psychological, rather than physiological factors. Increasing literature, however, suggests that neurophysiological processes related to pain sensitivity also play an important role in chronicity. To date, there has been limited research that has specifically explored the role of pain sensitivity within the FAM. This study addresses this gap by evaluating whether clinical measures of pain sensitivity help explain FAM-related outcomes, beyond model-relevant psychological predictors.

**Methods**: The study sample consisted of 80 adults with chronic and widespread musculoskeletal pain (pain was distributed across 4.96 ± 2.01 number of body sites including limbs and spine). Participants completed a single testing session that included measures of all of the major constructs of the FAM, including pain catastrophizing, pain-related fear, activity avoidance (self-report and functional measures), pain-related disability, depression and pain severity, as well as a battery of quantitative sensory testing that included measures of pressure pain threshold and temporal summation of mechanical pain across 8 body sites.

**Results**: A series of hierarchical regression analyses revealed that after controlling for the psychological predictors of the FAM, indices of pain sensitivity significantly predicted 4 of the 5 FAM-related outcomes (p < .05). Depression was the only outcome not significantly predicted by pain sensitivity. Interestingly, measures of pain sensitivity, but not FAM psychological factors, predicted the functional measure of activity avoidance (β = .249, t = 2.281, p < .05).

**Discussion/Conclusions**: These findings provide further evidence for the importance of neurophysiological factors within the FAM and have important clinical and theoretical implications.

### Parent-Targeted Education Regarding Infant Pain Management: A Scoping Review

Brianna Richardson^a^, Allyson Falconer^a^, Joshna Shrestha^a^, Christine Cassidy^a^, Marsha Campbell-Yeo^a^, and Janet Curran^a^

School of Nursing, Dalhousie University, Halifax, Nova Scotia, Canada

**CONTACT** Brianna Richardson brianna.richardson@dal.ca

© 2019 The Author(s). Published with license by Taylor & Francis Group, LLC.

This is an Open Access article distributed under the terms of the Creative Commons Attribution License (http://creativecommons.org/licenses/by/4.0/), which permits unrestricted use, distribution, and reproduction in any medium, provided the original work is properly cited.

**Introduction/Aim**: Procedural pain is a prevalent issue that parents recognize, reporting a strong desire for more information on all aspects of infant pain. The aim of this review was to explore the current evidence of parent-targeted education about infant pain, delivered throughout the perinatal period.

**Methods**: Using the Methodology for Joanna Briggs Institute Scoping Reviews, records were identified through PubMed, CINAHL, EMBASE, and ERIC databases, as well as by hand searching in PAIN, BMC Pediatrics, and the Journal of Perinatal Neonatal Nursing. Articles in English that describe or evaluate educational interventions on infant pain management targeted to parents during the perinatal period were included.

**Results**: The initial search yielded 6946 articles, with nine eligible. Included studies specified interventions for procedural pain management such as breastfeeding (n = 7), skin to skin contact (n = 3), facilitated tucking (n = 2), sucrose (n = 2), topical anesthetic (n = 2), non-nutritive sucking (n = 2), holding (n = 3), and deep breathing & distraction (n = 2). Educational intervention delivery methods included written components only (n = 2), video components only (n = 1) and multimodal (n = 6). Outcomes measured across studies included parental knowledge, self-efficacy, parental involvement in procedural pain management, stress, anxiety, postnatal depression, role attainment, pain assessment documentation, and measure of social support.

**Discussion/Conclusions**: Despite an area of high concern for parents of newborns, few studies addressed parent targeted educational interventions regarding infant pain. Of those that did, the educational interventions appear to improve parental knowledge, self-efficacy, and increased involvement in pain management activities. Future research examining the impact and efficacy of these educational interventions on parental and neonatal outcomes is warranted.

### Characteristics of Patients with Pain Admitted to a Regional Hospital in Victoria-Australia

M. F. Jeddi^a^, A. Gunadi^b^, L. Muns^b^, K. Rodda^c^, K. Kaur^a^, F. Cavestany-Ricker^a^, V. Jain^a^, J. Degtiareva^a^, L. K. Whitfort^d^, A. Maundy^e^, and M. Budge^f^

^a^Medical, Bendigo Health, Bendigo, Australia; ^b^Nursing, Bendigo Health, Bendigo, Australia; ^c^Physiotherapy, Bendigo Health, Bendigo, Australia; ^d^Bendigo Health, Bendigo, Australia; ^e^Library Sciences, Bendigo Health, Bendigo, Australia; ^f^Medical, Bendigo Health, Monash University, Bendigo, Australia

CONTACT M. F. Jeddi fjeddi@bendigohealth.org.au

© 2019 The Author(s). Published with license by Taylor & Francis Group, LLC.

This is an Open Access article distributed under the terms of the Creative Commons Attribution License (http://creativecommons.org/licenses/by/4.0/), which permits unrestricted use, distribution, and reproduction in any medium, provided the original work is properly cited.

**Introduction/Aim**: Australians living in rural and remote areas tend to have lower life expectancy, higher rates of disease and injury, and poorer access to and use of health services than people living in cities. We aim to document the demographic characteristics of patients and the comparison of pre and post admission functional gains, admitted to a Regional Victorian hospital in Australia.

**Methods**: This is a retrospective data-analysis of Australian Rehabilitation Outcomes Centre (AROC) data was undertaken with the data collected from 31/01/2005 till 4/05/2018. All patients had a primary diagnosis of acute pain were included. Mann-Whitney and correlations tests were performed to compare variable in the data including Functional Independence Measure.

**Results**: 464 patients {n = 154 (33.2%) were males} were included in the review with a mean age of 74.3 years. 33% patients were between 81–90 years of age. FIM Admission scores ranged from 20–126 with a median score of 87. Mean FIM change was 13.77. Mean length of Stay was 21.23 days. Mean FIM Admission Motor score was 56.65 with median of 59 and Mean FIM Discharge Motor score 69.38 with a median of 74. Mean FIM Admission cognitive score was 26.98 and Mean FIM Discharge cognitive score 28.29. Back pain reported by most of the patients, n = 272 (58.6%).

**Discussion/Conclusions**: There is a significant paucity of data around inpatient rehabilitation programs in rural/regional Australia. This study will provide a baseline for future prospective analyses/studies. Our findings also have implication for developing rehabilitation services for patients with pain in terms of functional improvement.

### Gender and Sex Differences in Self-Management of Chronic Pain in the Presence of Medical Comorbidities

Chrystelle El-Khoury^a^, and M Gabrielle Pagé 0000-0002-7742-2717^a^^b^

^a^Centre de recherche, Centre hospitalier de l’Université de Montréal, Montréal, Quebec, Canada; ^b^Department of Anesthesiology and Pain Medicine, Centre hospitalier de l’Université de Montréal, Montreal, Quebec, Canada

**CONTACT** Chrystelle El-Khoury chrystelle.el-khoury@umontreal.ca

© 2019 The Author(s). Published with license by Taylor & Francis Group, LLC.

This is an Open Access article distributed under the terms of the Creative Commons Attribution License (http://creativecommons.org/licenses/by/4.0/), which permits unrestricted use, distribution, and reproduction in any medium, provided the original work is properly cited.

**Introduction/Aim**: Many chronic pain patients also suffer from other medical conditions, leading to increased self-management demands. This study examines the association between sex, gender, patients’ confidence in their abilities to manage pain and their level of engagement in pain self-management in the context of comorbidities.

**Methods**: A transversal observational study design was used. One hundred twenty-seven individuals suffering from chronic non-cancer pain and ≥ 1 medical comorbidity were recruited from patient associations and social and conventional media. They completed online self-report questionnaires on gender role, medical burden, engagement in self-management and pain self-efficacy.

**Results**: Participants were predominantly of female sex (n = 103; 81%) and uniformly distributed in terms of gender (feminine = 33 (25%); masculine = 27 (21%); androgynous = 33 (26%); undifferentiated = 36 (28%)). Patients had been suffering from pain on average for 15.7 ± 13.4 years and had 3.6 ± 2.2 comorbidities. Results showed a significant interaction between sex and gender in levels of self-efficacy (F (3,111) = 4.25, *p* = 0.007). More specifically, patients of male sex and feminine gender had the highest levels of self-efficacy in managing pain (mean = 236.00 ± 65.1) compared to all other groups (overall mean = 195.63 ± 52.0). There were no significant interactions or main effects of sex and/or gender on levels of engagement in pain management (*p* < 0.05).

**Discussion/Conclusions**: Results show an interactive effect of sex and gender on how confident patients are to manage their pain. Research and clinical interventions geared at optimizing pain self-management should take into account both constructs.

### Relative Frequency and Risk Factors for Prolonged Opioid Therapy after Surgery and Trauma: A Systematic Review and Meta-Analysis

M Gabrielle Pagé 0000-0002-7742-2717^a^^b^, Irina Kudrina^c^, Patrice Ngangue^d^, Maude Fortier^a^, Elisabeth Martin^a^, Esthelle Ewusi-Boisvert^a^, Hervé T V Zomahoun^e^^f^, Jordie Croteau^f^, Daniela Ziegler 0000-0002-7210-5296^g^, Pierre Beaulieu^b^^h^, Céline Charbonneau^i^, Jennifer Cogan^b^^j^, Raoul Daoust^k^, Marc O Martel^l^, Andrée Néron^m^, Philippe Richebé^b^^n^, and Hance Clarke^o^^p^

^a^Carrefour de l'innovation et évaluation en santé, Centre de recherche, Centre hospitalier de l’Université de Montréal, Montreal, Quebec, Canada; ^b^Department of Anesthesiology and Pain Medicine, Faculty of Medicine, Université de Montréal, Montreal, Quebec, Canada; ^c^Department of Family Medicine, Faculty of Medicine, McGill University, Montreal, Quebec, Canada; ^d^Faculty of medicine and health sciences, Université de Sherbrooke, Sherbrooke, Quebec, Canada; ^e^Department of social and preventive medicine, Faculty of medicine, Université Laval, Québec, Quebec, Canada; ^f^Knowledge Translation and Implementation component of the Quebec SPOR-SUPPORT Unit, Health and Social Services Systems, Québec, Quebec, Canada; ^g^Direction de l’enseignement et l’Académie, Centre hospitalier de l’Université de Montréal, Montreal, Quebec, Canada; ^h^Department of anesthesiology, Centre Hospitalier de l’Université de Montréal, Montreal, Quebec, Canada; ^i^Association Québécoise de la douleur chronique (AQDC), Montreal, Quebec, Canada; ^j^Department of anesthesiology, Montreal Heart Institute, Montreal, Quebec, Canada; ^k^Emergency Medicine, Hôpital du Sacré-Coeur de Montréal, Montreal, Quebec, Canada; ^l^Faculty of Dentistry & Department of Anesthesia, McGill University, Montreal, Quebec, Canada; ^m^Pain clinic, Department of pharmacy, Centre Hospitalier de l’Université de Montréal, Montreal, Quebec, Canada; ^n^Department of anesthesiology, Hôpital Maisonneuve-Rosemont, Montreal, Quebec, Canada; ^o^Department of anesthesia and pain management, Toronto General Hospital, University Health Network, Toronto, Ontario, Canada; ^p^Transitional Pain Service, Toronto General Hospital, University Health Network, Toronto, Ontario, Canada

**CONTACT** M. Gabrielle Pagé gabrielle.page@umontreal.ca

© 2019 The Author(s). Published with license by Taylor & Francis Group, LLC.

This is an Open Access article distributed under the terms of the Creative Commons Attribution License (http://creativecommons.org/licenses/by/4.0/), which permits unrestricted use, distribution, and reproduction in any medium, provided the original work is properly cited.

**Introduction/Aim**: The objectives of this systematic review and meta-analysis were to examine the relative frequency and risk factors (patient, surgical, medical, clinical) for prolonged opioid therapy among surgical and trauma patients.

**Methods**: Studies published in English and French between 1998 and April 2018 examining risk factors for *prolonged* (3–6 months) or *chronic* (>6 months) opioid use after surgery/trauma were included. Literature search: seven databases were queried, empirical studies were identified via direct and back citation search, grey literature was also included. A minimum of two independent reviewers assessed studies for inclusion, extracted data and assessed studies quality.

**Results**: Thirty-five out of 10,003 screened articles were included. The median relative frequency of prolonged (50.9%) and chronic (58.5%) opioid therapy among pre-event patients already on opioid therapy was much higher compared to pre-event opioid naïve patients (4.1% and 2.6%, respectively). Tobacco use, depressive disorder and antidepressants use were significant risk factors for prolonged and/or chronic opioid therapy among pre-event opioid naïve patients. Tobacco use, depressive disorder and history of migraines were risk factors for prolonged opioid therapy among pre-event opioid-treated patients.

**Discussion/Conclusions**: Prevention initiatives to reduce the risk of prolonged opioid therapy after surgery or trauma should target specific health behaviors and psychiatric disorders; these interventions should be tailored based on patients’ pre-event opioid status.

### Capacity to Describe Inner Experiences Predicts Lower Pain-Related Mind-Wandering during a Smartphone-Based Mindfulness Task in People with Chronic Pain

Muhammad Abid Azam^a^, Vered Valeria Latman^a^, Helia Ghazinejad^a^, Amir Zarie^a^, Fatma Al-Rubeye^a^, Natasha Aguanno^a^, Zahra Mohamedbhai^a^, Myra Massey^a^, and Joel Katz 0000-0002-8686-447X^a^

Psychology, York University, Toronto, Ontario, Canada

**CONTACT** Muhammad Abid Azam abidazam@yorku.ca

© 2019 The Author(s). Published with license by Taylor & Francis Group, LLC.

This is an Open Access article distributed under the terms of the Creative Commons Attribution License (http://creativecommons.org/licenses/by/4.0/), which permits unrestricted use, distribution, and reproduction in any medium, provided the original work is properly cited.

**Introduction/Aim**: Pain-related mind-wandering (PRMW) may facilitate pain and disability in people with chronic pain. This study explored mindfulness skills that predict lower PRMW during mindfulness meditation (MM)

**Methods**: 133 participants (Age_M_ = 20.5 years, SD = 3.74; Male = 45) were classified into groups: 1-Chronic pain (CP; n = 35) based on self-reported CP diagnoses, 2-Depressive-anxious (DA; n = 67) based on severe symptoms on the Center for Epidemiological Studies-Depression subscale (≥21) or Beck Anxiety Inventory (≥36), or 3-Controls (n = 31) if neither CP nor DA criteria applied. Participants completed the Five Facet Mindfulness Questionnaire (FFMQ) assessing mindfulness skills: observing experiences, describing internal experiences (Describe), acting with awareness, non-judgement and non-reactivity. Participants practiced breath-focused MM on a smartphone (~12-minutes) and pressed “breath” or “other” buttons at the sound of tones if awareness was on breathing or another experience, respectively. The Mind-Wandering Inventory was completed post-MM using 3 items: awareness of bodily pain, thoughts about pain, and other unpleasant sensations. Pearson correlations were conducted between FFMQ and PRMW.

**Results**: Amongst all correlations, only Describe significantly predicted PRMW for CP (r = −0.46, p < 0.01), DA (r = 0.25, p < 0.05), and controls (r = 0.37, p < 0.05). CP participants had significantly higher % breath responses than the other groups.

**Discussion/Conclusions**: The mindfulness skill of describing inner experiences may be a vital component of mindfulness-based pain treatments.

### The Temporal Relationship between Catastrophizing and Chronic Pain

Abi Muere^a^, Laura Katz^b^, Alison Crawford^a^, J Curtis Nickel^c^, Lesley Carr^d^, Robert Moldwin^e^, Robert Mayer^f^, and Dean A Tripp^g^

^a^Department of Psychology, Queen’s University, Kingston, Ontario, Canada; ^b^Michael G DeGroote Pain Clinic, McMaster University Hospital, Hamilton, Ontario, Canada; ^c^Urology & Medicine, Queen’s University, Kingston, Ontario, Canada; ^d^Department of Surgery, University of Toronto, Toronto, Ontario, Canada; ^e^Department of Medicine, Hofstra University, New Hyde Park, New York, USA; ^f^Asante Physician Partners, Grants Pass, OR, USA; ^g^Department of Psychology, Urology & Anesthesiology, Queen’s University, Kingston, Ontario, Canada

**CONTACT** Abi Muere abigail.muere@queensu.ca

© 2019 The Author(s). Published with license by Taylor & Francis Group, LLC.

This is an Open Access article distributed under the terms of the Creative Commons Attribution License (http://creativecommons.org/licenses/by/4.0/), which permits unrestricted use, distribution, and reproduction in any medium, provided the original work is properly cited.

**Introduction/Aim**: Interstitial Cystitis/Bladder Pain Syndrome (IC/BPS) is a chronic pelvic pain syndrome characterized by persistent pain localized to the bladder and urologic symptoms of urgency, frequency, and dysuria (Nickel et al., 2009). While the temporal relationship between catastrophizing and chronic pain has been examined in other chronic pain populations (e.g., Campbell et al., 2012), the present study is the first to investigate this temporal relationship in an IC/BPS sample.

**Methods**: 151 women diagnosed with IC/BPS were recruited from tertiary care urology clinics and completed the Short Form McGill Pain Questionnaire and the Pain Catastrophizing Scale at baseline (Time 1), 6 months post-baseline (Time 2), and 12-months post-baseline (Time 3). Two cross-lagged panel analysis were conducted using residualized change scores.

**Results**: Increases in catastrophizing between Time 1 and Time 2 (Early Catastrophizing Change) predicted increases in pain between Time 2 and Time 3 (Later Pain Change), after controlling for early changes in pain and later changes in catastrophizing. In contrast, increases in pain between Time 1 and Time 2 (Early Pain Change) did not predict increases in catastrophizing between Time 2 and Time 3 (Later Catastrophizing Change), after controlling for early changes in catastrophizing and later changes in pain.

**Discussion/Conclusions**: Early increases in catastrophizing predict later increases in pain levels among patients with IC/BPS, but not vice versa. These findings add to the growing body of research emphasizing the importance of catastrophizing in the development and maintenance of chronic pain. Clinical implications include targeting catastrophizing for the management of IC/BPS pain.

### Exposure to Stressful Life Events Predicts Reduction in Placebo Analgesic Response

Terry K Borsook^a^, and Shelly-Anne Li^b^

^a^Department of Psychology, University of Toronto Mississauga, Mississauga, Ontario, Canada; ^b^Lawrence S Bloomberg Faculty of Nursing, University of Toronto, Toronto, Ontario, Canada

**CONTACT** Terry K Borsook terry.borsook@utoronto.ca

© 2019 The Author(s). Published with license by Taylor & Francis Group, LLC.

This is an Open Access article distributed under the terms of the Creative Commons Attribution License (http://creativecommons.org/licenses/by/4.0/), which permits unrestricted use, distribution, and reproduction in any medium, provided the original work is properly cited.

**Introduction/Aim**: Descending pain inhibitory system (DPIS) activation can moderate ascending pain signals and thus performs a critical role in regulating pain. Evidence from animal studies suggests that stress can lead to pain regulation dysfunction as indicated by DPIS changes. We investigated whether results observed in animal models generalize to humans using placebo analgesia (PA) as a means of assessing DPIS function.

**Methods**: We measured stressful life events (Stress Life Event Scale, Student Stress Scale) and perceived life stress (Perceived Stress Scale) for 50 healthy participants using an online questionnaire. Subsequently, during a lab session, a hand cream was applied to participants’ middle three fingers, who then experienced pressure induced pain stimuli before and after receiving a message regarding the cream. Participants who were randomized to the placebo condition (n = 23) were told that the cream contained a painkiller, promoting the expectation of reduced pain sensitivity; the control group (n = 27) was informed that the cream was a moisturizer.

**Results**: For pain tolerance, but not for pain threshold, stressful life experiences predicted 30% of the variance in PA effects, with greater exposure to stressful life events associated with diminished PA. Perceived stress, however, was not predictive of PA effects, suggesting a disconnect between stressor exposure and the subjective perception surrounding one’s ability to cope with challenges.

**Discussion/Conclusions**: Exposure to stressful life events can account for a considerable proportion of inter-individual differences in subsequent PA effects, suggesting DPIS dysfunction. Given the critical role played by the DPIS in regulating pain, these findings have important clinical implications.

### Can Sensitivity to Physical Activity Predict Objectively Measured Activity Levels Better than Psychological Factors?

Daniel Flegg^a^, Arthur Woznowski-Vu^a^, Andrea Aternali^b^, Rebekah Wickens^c^, Ryan Reid^d^, and Timothy H. Wideman^a^

^a^School of Physical and Occupational Therapy, McGill University, Montreal, Quebec, Canada; ^b^Psychology, McGill University, Montreal, Quebec, Canada; ^c^School of Physical and Occupational Therapy, Constance Lethbridge Rehabilitation Centre/McGill University, Montreal, Quebec, Canada; ^d^École de kinésiologie et des sciences de l’activité physique, CHU Sainte Justine, University of Montreal

**CONTACT** Daniel Flegg daniel.flegg@mail.mcgill.ca

© 2019 The Author(s). Published with license by Taylor & Francis Group, LLC.

This is an Open Access article distributed under the terms of the Creative Commons Attribution License (http://creativecommons.org/licenses/by/4.0/), which permits unrestricted use, distribution, and reproduction in any medium, provided the original work is properly cited.

**Introduction/Aim:** Low physical activity level is a leading public health problem due to the negative associated health conditions. Previous research has demonstrated difficulty in predicting objectively measured activity levels using psychological factors. The primary aim of this study is to explore whether sensitivity to physical activity (SPA) can predict objectively measured activity levels over psychological factors.

**Methods:** 73 participants with chronic (>3 months) musculoskeletal pain performed the 6-Minute Walk Test (6-MWT) and answered self-report questionnaires. SPA was measured by evaluating self-report pain before, during and after the 6-MWT using a numeric scale of 0 (no pain) to 100 (most pain imaginable). Participants wore a triaxial accelerometer (GT3X) on their hip for 9 days following the testing session. A bi-variate correlation was run to assess the relationship between objectively measured activity level outcomes, pain ratings, psychological factors and demographic data. A hierarchical regression was used to quantify the predictive capability of SPA and the self-report questionnaires while controlling for significant co-variates.

**Results:** SPA was significantly correlated with steps/minute, moderate-vigorous physical activity (MVPA) and vector count per minute. The Tampa Scale of Kinesiophobia and the Pain Catastrophizing Scale were not correlated with any objectively measured activity levels. SPA was able to explain a significant portion of the variance in steps per minute and average MVPA after controlling for significant co-variates.

**Discussion/Conclusions:** The analysis indicates that SPA is able to significantly predict certain outcome measures recorded by an accelerometer while psychological factors could not.

### Wide Variation in Opioid Prescribing Practices after Discharge in a Pediatric Teaching Hospital

Naiyi Sun^a^, Benjamin Steinberg^a^^b^, Jacqueline Hanley^a^, and Lisa Isaac^a^^b^

^a^Department of Anesthesia and Pain Medicine, Hospital for Sick Children, Toronto, Ontario, Canada; ^b^Hospital for Sick Children, University of Toronto, Toronto, Ontario, Canada

**CONTACT** Naiyi Sun naiyi.sun@sickkids.ca

© 2019 The Author(s). Published with license by Taylor & Francis Group, LLC.

This is an Open Access article distributed under the terms of the Creative Commons Attribution License (http://creativecommons.org/licenses/by/4.0/), which permits unrestricted use, distribution, and reproduction in any medium, provided the original work is properly cited.

**Introduction/Aim**: Opioid analgesics are frequently used for the treatment of acute pain in children following hospital discharge. In adults, opioid use can lead to overdose, diversion, and dependency. However, data on opioids prescription practices in the pediatric population is limited. The aim of this study is to characterize the amount and duration of opioids prescribed at time of discharge in children.

**Methods**: We retrospectively identified all patients who were discharged from the hospital with an opioid prescription over an 18-month period in a pediatric teaching hospital. For each prescription, we recorded the type of opioid, dosing frequency, number of prescribed doses, and prescribing service. The patient’s age, gender, weight, associated diagnosis, and hospital length of stay were also recorded.

**Results**: During the 18-month period, we identified a total of 3784 patients who received 3871 discharge opioid prescriptions. The average age was 8.6 years (range: 0–18). Median oral morphine equivalent dose prescribed was 1.0 mg/kg/day (range: 0.043–11.146). Median duration of outpatient opioid therapy was 3.75 days (range: 0.17–50). The most common diagnoses were supracondylar fracture (n = 383, 9.9%), adeno/tonsillectomy (n = 299, 7.7%), femur fracture (n = 190, 4.4%), and posterior spinal fusion (n = 116, 3.0%). The median number (range) of days of opioid prescribed were: supracondylar fracture 5 (0.83–25), adeno/tonsillectomy 3.33 (0.83–10), femur fracture 5 (0.83–14), and posterior spinal fusion 6.67 (1.25–33.3).

**Discussion/Conclusions**: We observed wide variations in dosage and duration of opioids prescribed at time of discharge. Further prescriber and patient education and guideline development are needed in the pediatric population.

### Linkage between Self-Reported and Administrative Data: A Review about Patient’s Willingness to Share Their Health Insurance Number for Research Purposes

Véronique Gagnon^a^, M. Gabrielle Pagé 0000-0002-7742-2717^b^, Nabiha Benyamina Douma^a^, and Anaïs Lacasse 0000-0002-3992-5145^a^

^a^Département des sciences de la santé, Université du Québec en Abitibi-Témiscamingue (UQAT), Rouyn-Noranda, Québec, Canada; ^b^Centre de recherche du Centre hospitalier de l’Université de Montréal (CRCHUM); Département d’anesthésiologie et de médecine de la douleur, Faculté de médecine, Université de Montréal, Montréal, Québec, Canada

**CONTACT** Véronique Gagnon veronique.gagnon3@uqat.ca

© 2019 The Author(s). Published with license by Taylor & Francis Group, LLC.

This is an Open Access article distributed under the terms of the Creative Commons Attribution-NonCommercial License (http://creativecommons.org/licenses/by-nc/4.0/), which permits unrestricted non-commercial use, distribution, and reproduction in any medium, provided the original work is properly cited.

**Introduction/Aim**: To study real-world benefits and risks of pain medications in large populations, administrative health and prescription drug databases are important data sources. However, claims data are not designed for research purposes and many benefits arise from the linkage of such data with patient-reported clinical information. This narrative review aimed to document prospective studies reporting the acceptability of health insurance number (HIN) sharing to allow the linkage between self-reported and administrative data.

**Methods**: A search for peer-reviewed articles/government reports was performed in PubMed (which provides free access to **MEDLINE**) and Google. Were extracted the proportion of participants willing to share their HIN, and when reported, reasons/determinants of this choice.

**Results**: Three papers were found among Canadian research. In a first study, patients followed in multidisciplinary pain clinics and included in a provincial registry (Quebec, Canada) were contacted by letter asking for their HIN and authorization to link their clinical and administrative data. A total of 44% responded and agreed. In the context of Canadian Community Health Surveys, 72–74% of respondents share their HIN for such linkage. Another study obtained an 83% participation rate when asking patients recruited in Quebec clinics and community pharmacies the permission to obtain their public or private pharmacy claims.

**Discussion/Conclusions**: Several factors could explain the variability of results such as the presence of a pre-established relationship of trust, the notoriety of the initiative (e.g., official census program), or the means of recruitment (e.g., in person, by mail months/years after being recruited in a registry).

### Evaluation of Chronic Pain Patient Healthcare Costs before and after a Tele-Education Intervention for Primary Care Providers in Underserved Communities

Dominika Bhatia 0000-0002-9621-0672^a^, Jane Zhao^b^, Ralph Fabico^b^, John Flannery^c^, Garry Salisbury^d^, and Andrea Furlan^c^

^a^Institute of Health Policy, Management and Evaluation, University of Toronto, Toronto, Ontario, Canada; ^b^Toronto Rehabilitation Institute, University Health Network, Toronto, Ontario, Canada; ^c^Department of Medicine, University of Toronto, Toronto, Ontario, Canada; ^d^Ontario Ministry of Health and Long-Term Care, Health Services Branch, Toronto, Ontario, Canada

**CONTACT** Dominika Bhatia dominika.bhatia@mail.utoronto.ca

© 2019 The Author(s). Published with license by Taylor & Francis Group, LLC.

This is an Open Access article distributed under the terms of the Creative Commons Attribution License (http://creativecommons.org/licenses/by/4.0/), which permits unrestricted use, distribution, and reproduction in any medium, provided the original work is properly cited.

**Introduction/Aim**: Lack of formal training poses a significant barrier to effective chronic pain management in primary care. Project ECHO (Extension for Community Healthcare Outcomes) is a medical education model that uses weekly videoconferencing rounds and case-based learning to connect specialists with providers in resource-scarce areas. The Chronic Pain and Opioid Stewardship ECHO (“ECHO”) was launched in Ontario, Canada in 2014. We sought to describe the annual healthcare utilization costs of chronic pain patients before and after their case presentation at ECHO.

**Methods**: We conducted a single-group before-and-after study using routinely-collected Ontario Health Insurance Plan administrative claims data between April 1, 2011 and March 31, 2018. Only the direct medical costs from the perspective of a public payer were considered.

**Results**: Our sample consisted of 46 patients presented at ECHO between September 2014 and March 2018, representing 19% of all ECHO case presentations. The annual healthcare utilization costs per patient before and after ECHO were $2,385.54 and $2,027.91, respectively, representing a 15% decline. The largest decrease was observed in the frequency of hospital visits, while the frequency of assessment or consultation visits, use of diagnostic radiology services, overall healthcare encounters, and the number of unique billing physicians saw a slight increase in the post-ECHO period.

**Discussion/Conclusions**: We observed notable savings in healthcare costs among patients presented at ECHO, despite a slight increase in utilization of certain healthcare services. Future studies should explore drivers of healthcare costs among ECHO patients to assess the cost-effectiveness of the program.

### Reduction in Anger in Participants with Chronic Pain after a Mobile-Based Mindfulness Intervention

Vered Valeria Latman^a^, Muhammad Abid Azam^a^, Helia Ghazinejad^a^, Amir Zarie^a^, Fatma Al-Rubeye^a^, Natasha Aguanno^a^, Zahra Mohamedbhai^a^, Myra Massey^a^, and Joel Katz 0000-0002-8686-447X^a^

Psychology, York University, Toronto, Ontario, Canada

**CONTACT** Vered Valeria Latman vvlatman@yorku.ca 

© 2019 The Author(s). Published with license by Taylor & Francis Group, LLC.

This is an Open Access article distributed under the terms of the Creative Commons Attribution License (http://creativecommons.org/licenses/by/4.0/), which permits unrestricted use, distribution, and reproduction in any medium, provided the original work is properly cited.

**Introduction/Aim**: To evaluate the effects of a novel 12-minute mobile-based mindfulness intervention on anger in participants with chronic pain, depression/anxiety and condition-free controls.

**Methods**: Four groups of university students: n = 42 with chronic pain (CP+app), n = 39 with symptoms of depression/anxiety (DA+app), and 2 groups of condition-free controls (CF+app; n = 54 and CF-app; n = 26) completed the Anger subscale of the Profile of Mood States at baseline (pre) and after (post) a 12-min intervention, during which participants were instructed to pay attention to the flow of breath and press “breath” or “other” buttons on a smartphone at the sound of a tone. The CF-app group attended to their breath for 12 minutes without use of the smartphone app.

**Results**: We used a 2-way ANOVA with Time (baseline, post-intervention) and Group (CP+app, DA+app, CF+app, CF-app) to evaluate Anger scores. The simple main effect of Group was significant at baseline, *F*(3,152) = 14.83, *p* < .001, η*p2 *= .22, and post-intervention, *F*(3,152) = 9.57, *p* < .001, η*p2 *= .15. At baseline, the CP+app and DA+app did not differ in Anger scores, which were significantly higher than CF+app and CF-app (*p* < .05). Post-intervention, anger levels for CP+app dropped to meet those of both CF+app and CF-app, while DA+app remained significantly higher than the rest (*p* < .05). Simple main effects of Time were significant for CP+app, *F*(1,152) = 27.90, *p* < .001, η*p2 *= .15 and DA+app, *F*(1,152) = 15.06, *p* < .001, η*p2 *= .09, but not for CF+pp or CF-app.

**Discussion/Conclusions**: Research has shown that anger can lead to increased pain sensitivity and intensity; therefore regulating anger using mindfulness may be a desirable goal as part of CP treatment.

### Patients’ and Caregivers’ Experiences with Pain Management in Children and Teenagers with Sickle Cell Disease Requiring Admission for Vaso-Occlusive Crisis

Claire Arbitre^a^, Nathalie Gaucher^a^, Evelyne D. Trottier^a^, Claude Julie Bourque^b^, Jessica Darilus^c^, Priscille-Nice Sanon^d^, Lionel N. Dabirabe^d^, Nancy Robitaille^e^, and Yves Pastore^e^

^a^Pediatric Emergency Department, Université de Montréal, Montreal, Quebec, Canada; ^b^Unité de Recherche en partenariat Famille, Centre de Recherche, Université de Montréal, Montréal, Québec, Canada; ^c^Parent Partner, Montréal, Québec, Canada; ^d^Patient Partner, Montréal, Québec, Canada; ^e^Pediatric Hematology Department, Université de Montréal, Montreal, Quebec, Canada

**CONTACT** Claire Arbitre clairearbitre@yahoo.fr

© 2019 The Author(s). Published with license by Taylor & Francis Group, LLC.

This is an Open Access article distributed under the terms of the Creative Commons Attribution License (http://creativecommons.org/licenses/by/4.0/), which permits unrestricted use, distribution, and reproduction in any medium, provided the original work is properly cited.

**Introduction/Aim**: The quality of life of children with sickle cell disease (SCD) depends on the severity and number of vaso-occlusive crises (VOC). The objective of this study was to explore the experiences of pediatric patients and their families during VOC.

**Methods**: This qualitative study used semi-structured interviews, designed in partnership with two patients and one parent. Two groups of participants were interviewed independently: adolescent patients and parents of pediatric patients hospitalised within the last 2 years for VOC. Data was transcribed in full and analysed using NVivo12. Descriptive thematic content analysis was performed by coding themes emerging from participants’ discourses and systematically compared.

**Results**: Between June and August 2018, eight interviews were conducted. Ten parents and five adolescents participated. Teenagers’ and parents’ answers mirrored each other’s. Prompt pain relief was crucial, although the side effects of pain medications used were an added source of suffering. Recent quality improvement initiatives such as standardized order sheets were noteworthy improvements. Given the unpredictability and severity of VOC, their impact on both patients’ and families’ lives were substantial, as was the long term emotional burden. Parents felt guilty given the hereditary nature of the disease, they encouraged neonatal and prenatal testing, and they sought definitive treatments for both VOC and SCD. Tensions within parent-teenager relationships were described centered on developing autonomy and protecting the child to improve adherence to treatments.

**Discussion/Conclusions**: Participants emphasized the need to provide timely adequate analgesia. Understanding the impact of VOC on patients’ lives and their socio-familial context is important to tailor clinical interventions.

### Healthcare Provider Knowledge, Attitudes, Beliefs and Practices Surrounding the Prescription of Opioids for Chronic Non-Cancer Pain: Mixed-Method Systematic Review

Joshua A. Rash 0000-0003-0927-0712^a^, Norman Buckley 0000-0002-1031-6813^b^, Jason W. Busse 0000-0002-0178-8712^b^, Tavis S. Campbell^c^, Bill Casley^d^, Kimberly Corace^e^, Lynn Cooper^f^, David Flusk^g^, Alfonso Iorio 0000-0002-3331-8766^h^, Kim L. Lavoie^i^, Patricia A. Poulin 0000-0002-3934-9870^j^, and Becky Skidmore^k^

^a^Psychology, Memorial University of Newfoundland, St. John’s, Newfoundland and Labrador, Canada; ^b^Anesthesia, McMaster University, Hamilton, Ontario, Canada; ^c^Psychology, University of Calgary, Calgary, Alberta, Canada; ^d^Opioid Response Task Force, Health Canada, Ottawa, Ontario, Canada; ^e^Royal Ottawa Mental Health Centre, Ottawa, Ontario, Canada; ^f^Canadian Injured Workers Alliance, Thunder Bay, ON, Canada; ^g^Anesthesia, Memorial University of Newfoundland, St. John’s, Newfoundland and Labrador, Canada; ^h^Health Research Methods, Evidence and Impact, McMaster University, Hamilton, Ontario, Canada; ^i^Psychology, University of Quebec at Montreal, Montreal, Québec, Canada; ^j^Ottawa Hospital Research Institute, Ottawa, Ontario, Canada; ^k^Independent Information Specialist, Ottawa, Ontario, Canada

**CONTACT** Josh Rash jarash@mun.ca

© 2019 The Author(s). Published with license by Taylor & Francis Group, LLC.

This is an Open Access article distributed under the terms of the Creative Commons Attribution License (http://creativecommons.org/licenses/by/4.0/), which permits unrestricted use, distribution, and reproduction in any medium, provided the original work is properly cited.

**Introduction/Aim**: The objective of this review was to synthesize the published evidence about knowledge, attitudes, beliefs, and practices that healthcare providers hold regarding the prescription of opioids for chronic non-cancer pain.

**Methods**: Ovid MEDLINE, Embase, PsycINFO, Cochrane Library and CINAHL bibliographic databases were searched from inception until April 2018. Qualitative and quantitative study designs that report on healthcare provider knowledge, attitudes, beliefs or practices in North America were eligible for inclusion. Studies reporting on interventions were also eligible for inclusion. Research assistants screened articles for inclusion and performed data extraction and risk of bias using validated tools.Confidence in evidence was evaluated using the GRADE approach.

**Results**: 96 studies were included that reported on nearly 18,500 healthcare providers. Healthcare providers are reluctant to prescribe opioids for chronic non-cancer pain. Perceived inadequate training, low self-efficacy, apprehension about diversion, suspicion of drug seeking behavior, fear of regulatory scrutiny, and concern about patient addiction and side effects contribute to this reluctance. There was significant variability in estimates of healthcare provider adherence to recommendations made by clinical practice guidelines. There is reasonable confidence that adherence to risk reduction strategies is under 50%. 26 studies were included that evaluated an intervention.

**Discussion/Conclusions**: Conclusions were difficult to draw due to variability in interventions, high risk of bias, methodological concerns, and limited number of studies reporting on interventions. Multifaceted interventions targeting several provider-related barriers were promising, but may be challenging to reproduce.

### Don’t Think Too Hard: Investigating Choices between Physical Pain and Cognitive Effort

Todd A. Vogel^a^, A. Ross Otto^a^, and Mathieu Roy 0000-0002-3335-445X^a^

Department of Psychology, McGill University, Montreal, Quebec, Canada

**CONTACT** Todd A. Vogel todd.vogel@mail.mcgill.ca

© 2019 The Author(s). Published with license by Taylor & Francis Group, LLC.

This is an Open Access article distributed under the terms of the Creative Commons Attribution License (http://creativecommons.org/licenses/by/4.0/), which permits unrestricted use, distribution, and reproduction in any medium, provided the original work is properly cited.

Pain is aversive and entails a motivational drive to escape further harm (Eccleson & Crombez, 1999). The expenditure of cognitive effort can also be aversive and the decision to engage in effortful cognitive processing involves evaluating the costs and benefits of the action (Kurzban et al. 2013). As cognitively demanding tasks are commonly used in investigating pain modulation, we contrasted the aversiveness of pain with the aversiveness of expending cognitive effort using a novel trade-off paradigm. Thirty-nine participants were offered a series of choices between different levels of a painful thermal stimulus and a cognitively demanding task. We observed a trade-off between expending cognitive effort and experiencing physical pain, wherein participants were more likely to accept pain with increasing cognitive demands. Our findings highlight the aversiveness nature of cognitive effort and its shared characteristics with other primary aversive stimuli.

**Introduction/Aim**: The expenditure of cognitive effort can be aversive and the decision to engage in effortful cognitive processing involves evaluating the costs and benefits of the action (Kurzban et al. 2013). Pain is also aversive and entails a motivational drive to escape further harm (Eccleson & Crombez, 1999). While avoidance of cognitive effort has been examined using a secondary incentive, such as monetary reward (Westbrook et al., 2013), it remains unclear how the cost/benefit analysis of expending effort is affected when paired with a primary incentive, such as pain avoidance. As difficult cognitive tasks are often used to investigate modulation of pain (see Torta et al., 2017), the characteristics of performing a cognitively demanding task itself should be taken into consideration. Here we contrasted the aversiveness of cognitive effort expenditure with physical pain using a novel trade-off paradigm.

**Methods**: Thirty-nine participants were offered a series of choices between different levels of a painful thermal stimulus and a cognitively demanding task. Five different levels of the *N*-back working memory task (*N* = 0 to *N* = 4) were used as the cognitively demanding task. The level of cognitive effort required to perform the task increased parametrically with the level of cognitive load (Braver et al., 1997). For the painful thermal stimulus, five different temperatures were used to elicit a painful sensation (45°C to 49°C). Temperatures and *N*-back task speeds were individually calibrated to equate the sensation and difficulty, respectively, across participants. At the start of each trial, the participant was shown a given level of each task (e.g., 2-back or a temperature corresponding to 28/100 on a pain scale) and was asked to make a decision between the two. After a decision was made, the corresponding task was administered (e.g., several trials of the *N*-back task, or a painful thermal stimulus).

**Results**: Our findings indicated a trade-off between the level of cognitive effort required and the level of physical pain. That is as the level of cognitive demand increased, participants were more likely to accept physical pain than choose to expend cognitive effort, *F*(4,152) = 61.69, *p* < .001. Conversely, as the level of offered pain increased participants were less likely to accept physical pain and instead preferred to expend cognitive effort, *F*(4,152) = 51.18, *p* < .001. Response times to making a decision mirrored this observed trade-off. That is, as physical pain and cognitive effort reached their equivalence point (i.e., the levels at which acceptance for either option is 50%), participants took longer to make a decision, *b* = 2.04, *t*(43.10) = 9.60, *p* < .001.

**Discussion/Conclusions**: Our findings highlight the aversive nature of cognitive effort. When paired with a primary aversive stimulus, such as pain, people will sometimes forego expending cognitive effort in exchange for the aversive stimulus. These results suggest that cognitive effort shares characteristics with other primary aversive stimuli and that avoiding expenditure of effort is motivationally driven.

### Examining the Development of a Community of Practice in Paediatric Project ECHO for Acute and Chronic Pain

Yalinie Kulandaivelu^a^^b^, Chitra Lalloo^a^^b^, Joanna Sale^c^^d^, Emily Seto^e^, and Jennifer Stinson^d^^f^

^a^Management and Evaluation, Institute for Health Policy, Toronto, Ontario, Canada; ^b^Hospital for Sick Children, Child Health and Evaluative Sciences, Toronto, Ontario, Canada; ^c^Li Ka Shing Knowledge Institute, Musculoskeletal Health and Outcomes Research, St. Michael’s Hospital, Toronto, Ontario, Canada; ^d^Institute for Health Policy, Management and Evaluation, University of Toronto, Toronto, Ontario, Canada; ^e^Institute of Health Policy, Management and Evaluation, University of Toronto, Toronto, Ontario, Canada; ^f^Department of Anesthesia and Pain Medicine, Hospital for Sick Children, Child Health and Evaluative Sciences, University of Toronto, Toronto, Ontario, Canada

**CONTACT** Yalinie Kulandaivelu yalinie.kulandaivelu@sickkids.ca

© 2019 The Author(s). Published with license by Taylor & Francis Group, LLC.

This is an Open Access article distributed under the terms of the Creative Commons Attribution License (http://creativecommons.org/licenses/by/4.0/), which permits unrestricted use, distribution, and reproduction in any medium, provided the original work is properly cited.

**Introduction/Aim**: Project ECHO® is an innovative, interactive model for healthcare provider (HCP) education that expands access and capacity to provide evidence-informed care. It uses videoconference technology to create a community of practice (CoP) among interprofessional specialist teams at an academic “Hub” and community HCPs called “Spokes” across the province of Ontario. This study examines the development of a CoP in Project ECHO for paediatric acute and chronic pain, and barriers and facilitators to its development.

**Methods**: A qualitative description design was undertaken with semi-structured, audio- taped interviews. The interview guide focused on structural components of a CoP (domain, practice, community) and barriers and facilitators to its development through the perspectives of HCPs. Interviews were transcribed verbatim and analyzed using qualitative content analysis.

**Results**: 21 HCPs who had participated in Project ECHO were interviewed (10 of 14 Ontario Local Health Integration Networks represented; 19% physicians, 19% nursing professions, and 62% allied health). Participants’ responses indicated evidence for each component of a CoP. The main barrier to CoP development reported was differences in participants’ perceptions of the program aims; main facilitator was the ECHO program format.

**Discussion/Conclusions**: This is one of the first studies to examine the CoP elements of the ECHO model. Further development of the CoP concept for healthcare settings is needed to enhance program delivery. Findings will be used to aid revisions of future program iterations.

### Impact of Pain Neuroscience Education on Pain Catastrophizing, Kinesiophobia, and Readiness for Self-Management: Preliminary Results

Matilda E. Nowakowski^a^, Graham Nishikawa^b^, Cathy Page^b^, Harsha Shanthanna^c^, Philip Chan^c^, Heather Radman^b^, and Julie Holmes^b^

^a^Department of Psychiatry and Behavioural Neurosciences/Chronic Pain Clinic, McMaster University/St. Joseph’s Healthcare Hamilton, Hamilton, Ontario, Canada; ^b^Chronic Pain Clinic, St. Joseph’s Healthcare Hamilton, Hamilton, Ontario, Canada; ^c^Department of Anesthesia/Chronic Pain Clinic, McMaster University/St. Joseph’s Healthcare Hamilton, Hamilton, Ontario, Canada

**CONTACT** Matilda E. Nowakowski mnowakow@stjoes.ca

© 2019 The Author(s). Published with license by Taylor & Francis Group, LLC.

This is an Open Access article distributed under the terms of the Creative Commons Attribution License (http://creativecommons.org/licenses/by/4.0/), which permits unrestricted use, distribution, and reproduction in any medium, provided the original work is properly cited.

**Introduction/Aim**: There is growing research evidence highlighting the importance of patient pain neuroscience education in the management of chronic pain (Louw et al., 2016). The current study investigated the effects of a 2-session pain neuroscience education group on pain catastrophizing, kinesiophobia, and readiness for adopting a self-management approach to chronic pain.

**Methods**: Patients attended a 2-session pain neuroscience education group prior to partaking in an 8-week self-management for chronic pain group consisting of exercise therapy and cognitive-behaviour therapy. Patients completed the Pain Catastrophizing Scale (PCS; Sullivan et al., 1995), Tampa Scale for Kinesiophobia (TSK, Miller et al., 1991), and Pain Stages of Change Questionnaire (PSOCQ; Kerns et al., 1997) before and after completing the pain neuroscience education group.

**Results**: Twenty-three patients participated in the study (*M* age = 58.65 years, *SD* = 2.38, 74% female). The most common chronic pain complaint was lower back pain (65.2%) followed by neck pain (17.4%). There were significant decreases in PCS scores (Pre: *M* = 22.71, *SD* = 8.76, Post: *M* = 18.90, *SD* = 10.76, *t*(20) = 3.36, *p* < .01) and precontemplation scores on the PSOCQ (Pre: *M* = 3.1, *SD* = .70, Post: *M* = 2.86, *SD* = .63, *t*(19) = 2.36, *p* = .03) from pre to post pain neuroscience education group. There were no changes on TSK scores.

**Discussion/Conclusions**: The current study provides preliminary evidence for the potential usefulness of a brief 2-session pain neuroscience education group as part of a self-management for chronic pain intervention.

### Predictors of Persistent Post-Surgical Pain after Total Knee Replacement: A Systematic Review and Meta-Analysis of Observational Studies

Vahid Ashoorion^a^, Behnam Sadeghirad^a^, Li Wang 0000-0003-1585-8846^a^, Atefeh Noori^b^, Luciane C. Lopes^c^, Yechan Kim^d^, Yaping Chang^e^, Meisam Abdar^f^, Leila Nasiri^g^, Mehdi Ghasemi^h^, Nadia Rehman^b^, Rachel Couban^a^, Yasir Rehman^b^, Gordon H. Guyatt 0000-0003-2352-5718^b^, and Jason W. Busse^a^

^a^Department of Anesthesia, McMaster University, Hamilton, Ontario, Canada; ^b^Health Research Methods, Evidence and Impact, McMaster University, Hamilton, Ontario, Canada; ^c^Pharmaceutical Science, University of Sorocaba, Soracaba, Sao Paolo, Brazil; ^d^Firestone Institute for Respiratory Health, McMaster University, Hamilton, Ontario, Canada; ^e^OrthoEvidence Inc., Burlington, Ontario, Canada; ^f^Deputy of Health, Isfahan University of Medical Sciences, Isfahan, Iran; ^g^Tehran University of Medical Sciences, Tehran, Iran; ^h^Department of Anesthesia, Isfahan University of Medical Sciences, Isfahan, Iran

**CONTACT** Vahid Ashoorion ashooriv@mcmaster.ca

© 2019 The Author(s). Published with license by Taylor & Francis Group, LLC.

This is an Open Access article distributed under the terms of the Creative Commons Attribution License (http://creativecommons.org/licenses/by/4.0/), which permits unrestricted use, distribution, and reproduction in any medium, provided the original work is properly cited.

**Introduction/Aim**: Each year 67,000 Canadians undergo total knee arthroplasty (TKA) and approximately 20% develop persistent post-surgical pain (PPSP). A complaint associated with functional limitations, reduced quality of life, and increased care-seeking. We systematically reviewed observational studies to explore factors associated with PPSP after TKA.

**Methods**: We searched Medline, EMBASE, Cochrane CENTRAL, AMED, PsycINFO, SCOPUS and SPORTDiscuss from inception to October 31, 2018. When possible, we pooled estimates of association for all independent variables reported by more than one study. Random effects models were used for all meta-analysis. We assessed certainty in evidence using the Grading of Recommendations Assessment, Development and Evaluation framework.

**Results**: Twenty-five studies, involving 44,069 participants, were eligible for review. Mean age of enrolled patients was 68.56 ± 8.77 years and 76% were female. Eligible studies reported the association of 56 predictors with PPSP after TKA, which clustered into 6 groups: (1) patient characteristics, (2) clinical findings, (3) genetic factors, (4) surgical approach, (5) radiographic findings, and (6) peri-operative management. Low and very-low quality evidence showed that higher preoperative pain (OR = 3.82, 95%CI 1.27 to 11.47) and female sex (OR = 1.23, 95%CI 1.08 to 1.4) were significantly associate with PPSP after TKA. Neither older age (OR = 0.97, 95%CI 0.88 to 1.07), higher BMI (OR = 1.07, 95%CI 1.00 to 1.13), or mood disorders (OR = 1.14, 95%CI 1.0 to 1.3) were predictors of PPSP following TKA.

**Discussion/Conclusions**: Addressing high peri-operative pain may be important in reducing PPSP after TKA.

### The Effects of Prescribed Analgesics on Driving

Tiffany Got^a^, Muhamad Bonse^b^, Ryan Lewis^c^, Bruce Haycock^c^, Jennifer Campos^c^, Behrang Keshavarz^c^, Susan Gorski^c^, and Andrea Furlan^d^

^a^Faculty of Medicine, University of Toronto, Toronto, Ontario, Canada;; ^b^Faculty of Engineering, Ryerson University, Toronto, Ontario, Canada;; ^c^Toronto Rehabilitation Institute, University Health Network, iDAPT, Toronto, Ontario, Canada;; ^d^Department of Medicine, University of Toronto, Toronto, Ontario, Canada

**CONTACT** Tiffany Got tiffany.got@mail.utoronto.ca

© 2019 The Author(s). Published with license by Taylor & Francis Group, LLC.

This is an Open Access article distributed under the terms of the Creative Commons Attribution License (http://creativecommons.org/licenses/by/4.0/), which permits unrestricted use, distribution, and reproduction in any medium, provided the original work is properly cited.

**Introduction/Aim**: Opioids have a broad impact on the central nervous system; side effects may impact the psychomotor and cognitive skills required for driving. This study aims to evaluate the impact of chronic short-acting opioid therapy on driving performance measures in a high-fidelity driving simulator.

**Methods**: Adult chronic pain patients using short acting opioids (SAO) or not using any opioids (NO) were recruited from Toronto Rehab Hospital’s comprehensive integrated pain program. Participants completed a baseline off-road test battery assessing cognitive and visual function. After an acclimatization trial in the driving simulator, subjects completed three 10-minute-long scenarios under varying road conditions in the high-fidelity simulator (iDAPT DriverLab).

**Results**: This ongoing study has evaluated 14 patients (SAO = 6, NO = 8) to date. The final sample size will increase to N = 40 to ensure test power. From the preliminary analysis, the SAO and NO groups were comparable on demographic variables, pain characteristics and performance on the cognitive and visual test battery. Vehicular control differed between groups; the standard deviation of lane position was 225.3 ± 55.4 and 256.1 ± 39.7 mm in the NO and SAO groups respectively. The brake reaction times were similar, 1111 ± 234 ms (NO) and 1107 ± 92 ms (SAO).

**Discussion/Conclusions**: This study will complement the existing body of literature on driving and opioids, which is mainly composed of observational and epidemiological evidence, since it lacks high quality experimental studies and studies conducted in high fidelity driving simulators.

### The Efficacy of the iPACK Block for Pain Management in Patients Undergoing Total Knee Arthroplasty

Michelle Biehl^a^, Lisa Wild^b^, Kyle Waldman^c^, Sherida Chambers^b^, Farzana Haq^d^, Ronald A. Easteal^a^, and Monakshi Sawhney^e^

^a^Department of Biomedical and Molecular Sciences, Queen’s University, Kingston, Ontario, Canada; ^b^Department of Surgery, Humber River Hospital, Toronto, Ontario, Canada; ^c^Department of Anesthesiology, Humber River Hospital, Toronto, Ontario, Canada; ^d^Emerging Issues, Canadian Institute for Health Information (CIHI), Toronto, Ontario, Canada; ^e^School of Nursing, Queen’s University, Kingston, Ontario, Canada

**CONTACT** Michelle Biehl michelle.biehl@queensu.ca

© 2019 The Author(s). Published with license by Taylor & Francis Group, LLC.

This is an Open Access article distributed under the terms of the Creative Commons Attribution License (http://creativecommons.org/licenses/by/4.0/), which permits unrestricted use, distribution, and reproduction in any medium, provided the original work is properly cited.

**Introduction/Aim**: Total knee arthroplasty (TKA) is a painful procedure that requires effective pain management to facilitate early rehabilitation. Peripheral nerve blocks including the adductor canal block (ACB) and femoral nerve block (FNB) are effective in managing anterior knee pain but not posterior knee pain. The iPACK (Interspace between the Popliteal Artery and posterior Capsule of the Knee) targets posterior knee pain. However, there is limited data regarding the effectiveness of the iPACK block when added to other blocks. The aim of this study was to determine the efficacy of the iPACK block for pain management in patients undergoing TKA.

**Methods**: A retrospective chart review was conducted to identify the time to first opioid dose, pain, and ambulation distance postoperatively in patients (n = 607) who underwent TKA under spinal anesthesia and received either FNB, ACB, FNB plus iPACK, or ACB plus iPACK block between June 2017 and July 2018.

**Results**: Mean pain scores on movement were lower on the day of surgery for patients who received an iPACK block in addition to FNB (0/10) or ACB (1/10) compared to patients who received ACB (2/10), and FNB (1.5/10) alone (p = 0.25). There was no difference between the groups regarding time to first opioid dose, pain or ambulation distance to POD 2.

**Discussion/Conclusions**: iPACK block was associated with decreased pain on POD 0 when combined with FNB and ACB, however this was not clinically significant. The lack of significant findings regarding other outcomes may be related to the reliance on chart data. A prospective study may report different findings.

### Differential Risk Factors for Functional Disability versus Pain Interference One Year after Major Pediatric Major Surgery

Brittany N. Rosenbloom^a^, Lisa Isaac^b^, Fiona Campbell^b^, Gabrielle Pagé^c^, Jennifer Stinson^d^, and Joel Katz 0000-0002-8686-447X^a^

^a^Psychology, York University, Toronto, Ontario, Canada; ^b^Anesthesia, The Hospital for Sick Children, Toronto, Ontario, Canada; ^c^Centre de recherche du Centre hospitalier de l’Université de Montréal, Montreal, Québec, Canada; ^d^Nursing, The Hospital for Sick Children, Toronto, Ontario, Canada

**CONTACT** Brittany Rosenbloom bnrosen@yorku.ca

© 2019 The Author(s). Published with license by Taylor & Francis Group, LLC.

This is an Open Access article distributed under the terms of the Creative Commons Attribution License (http://creativecommons.org/licenses/by/4.0/), which permits unrestricted use, distribution, and reproduction in any medium, provided the original work is properly cited.

**Introduction/Aim**: The Functional Disability Inventory (FDI) and the Patient Reported Outcomes Measurement Information System (PROMIS) Pediatric Pain Interference Scale (PPIS) are commonly used measures of disability but the former is general whereas the latter is pain-specific. We compared the FDI and PPIS in children after major surgery and identified differential predictors for each scale.

**Methods**: We studied 79 children (58.23% female, mean age = 14.56 years, SD = 2.31) and their parents/guardians prior to surgery (T0), during hospitalization (T1), and at 6- (T2) and 12-months (T3) post-surgery. Data was collected by questionnaire and chart review.

**Results**: At T3, FDI (M = 12.51, SD = 11.01, Range: 0–42) and PPIS scores (M = 11.65, SD = 8.30, Range: 0–31) were highly correlated (r = .807, p < .001). Multivariate linear regression revealed the final model for T3 FDI included the following T0 variables: child pain anxiety, FDI, child chronic pain acceptance, and parent anxiety sensitivity [R^2^ = .363, F(4,53) = 4.401, p = .004]. T0 child FDI (B = .363, p = .010) and T0 parent anxiety sensitivity (B = .438, p = .027) were the only significant predictors of child T3 FDI. Multivariate regression analysis revealed the final model predicting T3 PPIS included T0 child pain anxiety, child symptoms of posttraumatic stress, child general anxiety, child anxiety sensitivity, child pain self-efficacy, child FDI, child PPIS, and parent anxiety sensitivity [R^2^ = .361, F(8,43) = 3.033, p = .009]. Only T0 parent pain anxiety was a significant predictor of T3 PPIS (B = .340, p = .039).

**Discussion/Conclusions**: While the FDI and PPIS are highly correlated for children after major surgery, they represent different constructs and the one-year outcomes for each measure are predicted by different risk factors.

### Recovery Expectancies, Pain, and PTSD Symptom Severity

Catherine Paré^a^, Michael J. L. Sullivan 0000-0002-4228-1678^a^, and Lara Kojok^a^

Department of Psychology, McGill University, Montreal, Quebec, Canada

**CONTACT** Catherine Paré catherine.pare2@mail.mcgill.ca

© 2019 The Author(s). Published with license by Taylor & Francis Group, LLC.

This is an Open Access article distributed under the terms of the Creative Commons Attribution License (http://creativecommons.org/licenses/by/4.0/), which permits unrestricted use, distribution, and reproduction in any medium, provided the original work is properly cited.

**Introduction/Aim**: Research suggests that post-traumatic stress symptoms are prevalent in individuals who have sustained whiplash injuries following motor vehicle accidents. “Mutual maintenance” models have been used to explain high rates of comorbidity between pain and Post-Traumatic Stress Disorder (PTSD). Although several studies have provided support for mutual maintenance models of PTSD and pain, the processes by which post-traumatic stress symptoms and pain are mutually maintained has not been systematically investigated. The purpose of the present study was to examine the role of expectancies as a mediator of the relationship between PTSD symptoms and pain.

**Methods**: A sample of 127 individuals with whiplash injuries completed measures of pain, PTSD, and recovery expectancies at the beginning and end of their participation in a 7-week multidisciplinary pain rehabilitation program.

**Results**: Correlational analyses revealed that recovery expectancies were significantly associated with pre-treatment PTSD symptoms and post-treatment pain severity. Cross-sectional linear regressions revealed that pre-treatment symptoms of PTSD, but not recovery expectancies, emerged as a significant predictor of pre-treatment pain severity. However, pre-treatment PTSD symptoms and recovery expectancies both contributed unique variance to the prediction of pain severity at treatment termination.

**Discussion/Conclusions**: The results of this study showed that recovery expectancies have a prospective, but not concurrent, relation to pain severity. Recovery expectancies predicted end-of-treatment pain severity but did not mediate the relation between PTSD symptoms and pain severity. The results suggest that intervention techniques designed to modify recovery expectancies could enhance treatment outcomes for individuals with whiplash injuries who are also experiencing symptoms of PTSD.

### Why Is Dyspareunia the “Neglected Symptom” of Endometriosis? Unexpected Insight from Qualitative Interviews

Kate Wahl^a^, Michelle Lisonek^b^, Kelly Smith^c^, Paul Yong^b^*, and Susan Cox^a^*

^a^School of Population and Public Health, University of British Columbia, Vancouver, British Columbia, Canada; ^b^Obstetrics and Gynecology, University of British Columbia, Vancouver, British Columbia, Canada; ^c^Obstetrics and Gynecology, Vancouver General Hospital, Vancouver, British Columbia, Canada

**CONTACT** Kate Wahl kate.wahl@cw.bc.ca *These authors contributed equally to this work

© 2019 The Author(s). Published with license by Taylor & Francis Group, LLC.

This is an Open Access article distributed under the terms of the Creative Commons Attribution License (http://creativecommons.org/licenses/by/4.0/), which permits unrestricted use, distribution, and reproduction in any medium, provided the original work is properly cited.

**Introduction/Aim**: Endometriosis is a gynecological condition that affects 10% of reproductive-age women. At least half of this population experience deep dyspareunia – pain with deep penetration during sexual activity. The neglect of dyspareunia by endometriosis patients and providers is well documented and has been attributed to taboos about sexual intercourse or normalization of sexual pain.

**Methods**: Semi-structured interviews about the nature of sexual pain with participants in a clinical data registry who consented to be contacted for future research, had suspected or diagnosed endometriosis, were at least 18 years old, and had current or previous sexual pain alone or with a partner. Thematic analysis of the interview transcripts was conducted.

**Results**: Six participants were interviewed. In addition to predetermined themes about the nature of dyspareunia, four emergent themes were related to the neglect of endometriosis-associated sexual pain in the patient-provider relationship. These were normalization, self-management, acceptance, and limited definition.

**Discussion/Conclusions**: As suggested in previous research, participants considered pain a normal part of sexual intercourse. Participants also described self-management techniques (changing positions, non-penetrative sexual activities) that reduce the impact of dyspareunia. Furthermore, participants accepted sexual pain because of its brief duration. Finally, conventional definitions of endometriosis-associated dyspareunia that focus on pelvic pain with vaginal penetration excluded the experiences of people whose pain occurred elsewhere (in the hips, back, thighs) or who primarily engaged in non-penetrative sexual activity. Each of these factors may contribute to the neglect of endometriosis-associated sexual pain in clinical and research encounters.

### Characterizing Neuropathic and Nociceptive Pain in the African Naked Mole-Rat (*Heterocephalus Glaber*)

Sandra J. Poulson^a^, Melissa M. Holmes^a^, and Loren J. Martin^a^

Department of Psychology, University of Toronto Mississauga, Mississauga, Ontario, Canada

**CONTACT** Sandra J. Poulson sandra.poulson@mail.utoronto.ca

© 2019 The Author(s). Published with license by Taylor & Francis Group, LLC.

This is an Open Access article distributed under the terms of the Creative Commons Attribution-NonCommercial License (http://creativecommons.org/licenses/by-nc/4.0/), which permits unrestricted non-commercial use, distribution, and reproduction in any medium, provided the original work is properly cited.

Neuropathic pain caused by injury or disease of the somatosensory system occurs less frequently in children. Young children show better sensory recovery and are less likely to develop long-term chronic neuropathic pain than adults following nerve injury. These differences also appear to strongly correlate with developmental stage. To investigate the influence of development on neuropathic pain, we applied a nerve injury model to naked mole-rat subordinate adults, the majority of which do not experience puberty. Using adult mice for comparison, we measured responses to three stimuli: mild and strong mechanical and mild cold. We found similar mechanical responses, but there was a lack of response to mild cold in the naked mole-rat. We followed with assays using stimuli that target nociceptive pathway receptors implicated in cold perception: mustard oil, icilin, menthol, and a cold sensitivity dry ice assay. Our results show species differences in stimuli responses, likely reflecting the unusual evolutionary pressures experienced by the naked mole-rat.

**Introduction/Aim**: The nociceptive system of the African naked mole-rat contains unique adaptations that make the species insensitive to skin pain from some chemical stimuli. Sensitivity to thermal and mechanical stimuli are hallmark symptoms of adult chronic pain following nerve injury, yet young children are less likely to develop chronic neuropathic pain. The majority of adult naked mole-rats never undergo puberty due to the eusocial nature of each colony and are socially subordinate to a single queen naked mole-rat and 1 to 3 breeding males. Thus, to distinguish differences in evoked stimuli sensitivity development typically observed in an adult (post-pubertal) rodent neuropathic pain model, we compared nociceptive behavior of adult subordinate (pre-pubertal) naked mole-rats and mice.

**Methods**: We developed the Decosterd and Woolf spared nerve injury (SNI) model of neuropathic pain for the naked mole-rat and applied SNI and sham surgery to male and female subordinate naked mole-rats, and also applied SNI and sham surgery to CD1 adult mice. We then measured behavior across 4 weeks using a mild mechanical assay (Chaplan up and down method, von Frey), a strong mechanical assay (Tal and Bennett 1994, pin prick test), and a mild cold assay (Yoon et al. 1994, acetone droplet test) to the hind paw ipsilateral to surgery.

**Results**: Sensitivity to a mild mechanical stimulus and a strong mechanical stimulus were comparable between naked mole-rats and mice, despite the higher baseline threshold in naked mole-rats (a difference also observed between rats and mice). However, naked mole-rats showed no sensitivity to mild cold application before or after nerve injury whereas mice showed a marked increase in sensitivity to mild cold stimulus after nerve injury.

**Discussion/Conclusions**: All naked mole-rat subordinate (pre-pubertal) adults develop mechanical but not mild cold sensitivity after nerve injury. The mechanical sensitivity after nerve injury may be due to a developed adult peripheral nervous system separate from a lack of pubertal maturity. This study provides a unique glimpse into separating adult status from pubertal status with respect to evoked stimulus sensitivity after nerve injury. The lack of sensitivity development to mild cold stimulus observed in naked mole-rats may be due to a modification or expression difference in receptors of nociceptive fibers, considering other rare sensory adaptations observed in this species. We followed the observation of lack of mild cold sensitivity with assays using mustard oil, icilin, menthol, and a cold sensitivity dry ice assay to determine differences in behavior compared to mice. Our results show differences between responses among subordinate naked mole-rats and mice, likely reflecting the unusual evolutionary pressures experienced by the eusocial, subterranean colony-dwelling naked mole-rat.

### Pain Intensity Ratings are Altered by Prior Exposure to an Unrelated Numeric Anchor

Rebecca E. Lewinson^a^, and Joel Katz 0000-0002-8686-447X^a^

Department of Psychology, York University, Toronto, Ontario, Canada

**CONTACT** Rebecca Lewinson lewinson@yorku.ca

© 2019 The Author(s). Published with license by Taylor & Francis Group, LLC.

This is an Open Access article distributed under the terms of the Creative Commons Attribution License (http://creativecommons.org/licenses/by/4.0/), which permits unrestricted use, distribution, and reproduction in any medium, provided the original work is properly cited.

**Background**: How others rate another person’s pain is an important field of study, as it can determine how and when patients receive treatment for their pain. Anchoring effects occur when exposure to a numeric quantity biases a person’s subsequent judgment involving other quantities. Anchoring effects have been demonstrated in clinical and non-clinical settings, but rarely have been studied in the context of pain. This study aims to determine whether exposure to a random numeric anchor influences subsequent pain intensity ratings.

**Methods**: 385 participants read a vignette describing a patient with chronic pain before being randomly assigned to one of four groups. Groups 1 and 2 spun an 11-wedge number wheel (0–10) that was programmed to stop on a high number (“8”) or a low number (“2”), respectively. Group 3 spun a similar letter wheel (A-K) that stopped on “C” or “I” (Control 1). Group 4 did not spin a wheel (Control 2). Participants were then asked to rate the patient’s pain intensity using a 0–10 numeric rating scale.

**Results**: The high-number group rated the patient’s pain (*Median±IQR *= 8 *± *2) significantly higher than the letter wheel control (*Median±IQR = 7 ± 2*, *p*= .023) and the low-number group (*Median±IQR *= 6 *± *2, *p* < .001). The low-number group rated the pain significantly lower than Control 1 and 2 (*Median±IQR = 7 ± 2*) (both *p* < .05).

**Conclusions**: Pain ratings were influenced by prior exposure to a random number with no relevant information about the patient’s pain. Anchoring effects may operate in clinical settings among health care providers, or patients themselves, when asked for a numeric pain rating.

### Mannitol Cream for Pain Control, a Chart Review

Helene Bertrand

Department of family practice, University of British Columbia, Vancouver, British Columbia, Canada

**CONTACT** Helene Bertrand dr.hbertrand@gmail.com

© 2019 The Author(s). Published with license by Taylor & Francis Group, LLC.

This is an Open Access article distributed under the terms of the Creative Commons Attribution License (http://creativecommons.org/licenses/by/4.0/), which permits unrestricted use, distribution, and reproduction in any medium, provided the original work is properly cited.

**Introduction/Aim**: topical mannitol was shown to down regulate the capsaicin pain receptor. How effective would it be for pain control in clinical practice?

**Methods**: a chart review of 235 patients who received a 30% mannitol, cream for pain. Each patient used the cream on whatever part of their body was painful. Their chart contained a data sheet with: date, time, numeric pain score 0 = no pain to 10 = worst imaginable pain before and 30 minutes after application, minutes until relief, duration of relief.

**Results**: Of the 235 participants, there were 366 different areas of the body that were treated. The average pain relief was 50.8% (SD 29.8), median 52.94%. Areas of the body experiencing over 60% relief were the thumb and fingers, wrists, thigh, leg and knee (where the affected nerves are closest to the skin surface). The following areas had pain relief scores between 50% and 59%: foot, neck, arm, hip and back. 7 people developed a transient rash in the area where the cream was being applied, which resolved when cream use was stopped.

**Discussion/Conclusions**: The average pain relief for mannitol cream was 50.8% whereas for narcotics it is 36%, for oral or topical NSAIDS it is 23% and acetaminophen it is 13%. This is, however, only a retrospective observational study, not a randomized placebo-controlled study so it is, subject to the placebo effect. Topical mannitol may prove useful in providing pain relief. It needs to be tested in a randomized, placebo-controlled study which is currently underway.

### Does Parent Sensitivity Moderate the Relationship between Preschooler Pain Behaviour and Preschool Attachment Status?

Monica C. O’Neill^a^, Rebecca R. Pillai Riddell^a^, Hartley Garfield^b^, and Saul Greenberg^b^

^a^Psychology, York University, Toronto, Ontario, Canada; ^b^Paediatrics, University of Toronto, Toronto, Ontario, Canada

**CONTACT** Monica C. O’Neill mconeill@yorku.ca

© 2019 The Author(s). Published with license by Taylor & Francis Group, LLC.

This is an Open Access article distributed under the terms of the Creative Commons Attribution License (http://creativecommons.org/licenses/by/4.0/), which permits unrestricted use, distribution, and reproduction in any medium, provided the original work is properly cited.

**Introduction/Aim**: Investigating the relationship between child-caregiver behaviours during preschoolers’ vaccinations and preschool attachment could inform the use of the vaccination paradigm for early screening and intervention by health care providers. The present study examined if caregiver sensitivity during the vaccination appointment moderated the relationship between preschooler pain behaviour and attachment.

**Methods**: Child-caregiver dyads (*n *= 40) with preschool (4–5 years) immunization and attachment data were selected from the OUCH Cohort.

Video footage from preschoolers’ vaccinations were used to code caregiver sensitivity (MBQS-SF; Tarabulsy et al., 2009) and pain reactivity (0–29 seconds post-needle) and regulation (30–59 seconds post-needle [FLACC; Merkel et al., 1997]). In a separate lab-based assessment, the separation-reunion procedure was coded using the PARS (Moss et al., 2015) to examine preschoolers’ scaled scores on the six attachment categories (secure, avoidant, ambivalent, disorganized, controlling-caregiving, controlling-punitive).

**Results**: Moderation analyses examined the unique contribution of caregiver sensitivity, preschoolers’ pain (reactivity and regulation separately), and their interaction on the six attachment scales. There was a significant interaction between preschooler pain regulation and caregiver sensitivity on controlling-punitive behaviours (R^2^ = .289, *B *= −.261, *p *= .002). Specifically, there was a significant positive relationship between preschooler pain-related distress during the regulatory period and controlling-punitive attachment behaviours, when caregiver sensitivity was low (*B = *.159, *p = *.001) or average (*B = *.060, *p = *.034).

**Discussion/Conclusions**: When caregiver sensitivity was low or average, higher preschooler pain during the regulatory period predicted higher controlling punitive attachment behaviours. Findings identify how preschooler pain regulation and caregiver responding may inform the child-caregiver relationship outside of the vaccination context.

### The Impact of High Salt Diet on the Nociceptive Pain Thresholds and Functional Phenotype of Myeloid Cells

Anni Fan^a^, Xiang Qun Shi^b^, and Ji Zhang^c^^d^

^a^Department of Microbiology and Immunology, Faculty of Medicine, McGill University, Montreal, Quebec, Canada; ^b^The Alan Edwards Centre for Research on Pain, McGill University, Montreal, Quebec, Canada; ^c^The Alan Edwards Centre for Research on Pain, Department of Neurology, McGill University, Montreal, Quebec, Canada; ^d^Neurosurgery, Department of Microbiology and Immunology, Faculty of Medicine, Faculty of Dentistry, McGill University, Montreal, Quebec, Canada

**CONTACT** Ji Zhang ji.zhang@mcgill.ca

© 2019 The Author(s). Published with license by Taylor & Francis Group, LLC.

This is an Open Access article distributed under the terms of the Creative Commons Attribution License (http://creativecommons.org/licenses/by/4.0/), which permits unrestricted use, distribution, and reproduction in any medium, provided the original work is properly cited.

**Introduction/Aim**: Though several studies have shown that high salt diet (HSD) is related to the development of chronic diseases, the effect of long-term high-salt intake on the immune system and pain behavior remains elusive. Here, we aimed to investigate whether and how HSD affected mouse pain thresholds and innate immunity, especially myeloid cells.

**Methods**: Healthy C57BL/6 male and female mice were fed with HSD (containing 4% NaCl in chow and 1% NaCl in water) right from weaning period (3-week after birth) for 1–3 months.

**Results**: Behavioral analysis demonstrated that HSD-fed mice were hypersensitive to mechanical stimuli. Moreover, HSD induced an expansion of circulating monocytes, especially the proinflammatory monocyte (Ly6C^high^, CCR2^+^) subsets. HSD also increased the density of Iba-1+ microglia and the number of FcγII/III receptor (CD16/32) expressing cells in the lumbar spinal cord. Prolonged HSD feeding, meanwhile, resulted in increased infiltration of monocyte to the peripheral nerves, contributing to the monocyte-derived macrophage (CD11b^+^, F4/80^+^) population. Finally, shifting the HSD back to the regular laboratory chow (with 0.3% NaCl) cannot reverse the HSD-induced mechanical hypersensitivity myeloid cell expansion and polarization towards the inflammatory subsets in the periphery. However, treating HSD mice with CCR2 antagonist effectively normalized the pain threshold and myeloid cell profile in the peripheral blood and nerves.

**Discussion/Conclusions**: Long term HSD feeding decreased mouse mechanical thresholds, in parallel with an expansion and polarization of myeloid cells towards inflammatory status. Such changes were not reversible when HSD-fed mice back to normal diet. However, blocking CCR2 signaling successfully attenuated HSD-induced hypersensitivity.

### The Impact of a Pain Educational Intervention on Nursing Students’ Knowledge, Attitudes, and Self-Efficacy regarding Pain Management

Eid Aldossary^a^, Monakshi Sawhney^a^, Joan Tranmer^a^, and Laurie Gedcke-Kerr^a^

Faculty of Health Sciences, School of Nursing, Queen’s University, Kingston, Ontario, Canada

**CONTACT** Eid Aldossary 16eha1@queensu.ca

© 2019 The Author(s). Published with license by Taylor & Francis Group, LLC.

This is an Open Access article distributed under the terms of the Creative Commons Attribution License (http://creativecommons.org/licenses/by/4.0/), which permits unrestricted use, distribution, and reproduction in any medium, provided the original work is properly cited.

**Introduction/Aim**: Poor pain management can result from nurses’ lack of knowledge, or personal beliefs or attitudes Recent studies report that nursing students have inadequate knowledge regarding effective pain management, and often perform poorly on pain knowledge and attitudes surveys Improving pain knowledge is an important step for nursing students to optimize ineffective pain management practices during their education and when in clinical practice. Therefor the purpose of this study was to examine nursing students’ knowledge, attitudes, and self-efficacy in assessing and managing pain before and after participating in a pain educational intervention.

**Methods**: This study included a sample of 26 undergraduate nursing students who are in the second year of the 4-year Baccalaureate program. All consenting participants completed a pre-test prior to the intervention. The intervention consisted of didactic and on-line education. The post-test was completed following the intervention.

**Results**: The results showed that the students had significantly higher KASRP scores after the educational program (67.7%) versus before (56.8%) (p < .001). Prior to the pain education, 39.4% of students indicated they were confident at assessing and managing pain; there was a statistically significant increase to 69.1% after the program (p < .001). Pharmacological knowledge was a topic in need of improvement amongst participants as we found that knowledge in this area did not increase after the intervention.

**Discussion/Conclusions**: This intervention increased students’ knowledge, attitudes and self-efficacy regarding pain management. Further research is needed to test this intervention in other programs in Ontario and Canada. Additionally, this information may be useful for educators when planning clinical opportunities for students to acquire appropriate pain knowledge and attitudes, with the goals of improving nursing practice, and enhancing patient care.

References1.
Ene
KW, Nordberg
G, Bergh
I, Johansson
FG, Sjostrom
B. Postoperative painmanagement–the influence of surgical ward nurses. J Clin Nurs. 2008;17:2042–50.1870578110.1111/j.1365-2702.2008.02278.x2.
Ung
A, Salamonson
Y, Hu
W, Gallego
G. Assessing knowledge, perceptions andattitudes to pain management among medical and nursing students: A review of the literature. BrJ Pain. 2016;10(1):8–21. doi:
10.1177/2049463715583142.27551407PMC4977961

### How Do Pain Characteristics, Comorbidity Severity and Patient Characteristics Influence Chronic Pain Patients’ Self-Management Priorities?

Marie-Eve Martel^a^, Chrystelle El-Khoury^b^, and M Gabrielle Pagé^c^

^a^Department of Psychology, Université du Québec à Trois-Rivières, Trois-Rivières, Québec, Canada; ^b^Centre de recherche, Centre hospitalier de l’Université de Montréal, Montreal, Quebec, Canada; ^c^Centre de recherche, Centre hospitalier de l’Université de Montréal, & Department of Anesthesiology and Pain Medicine, Université de Montréal, Montreal, Quebec, Canada

**CONTACT** Marie-Eve Martel Marie-Eve.Martel1@uqtr.ca

© 2019 The Author(s). Published with license by Taylor & Francis Group, LLC.

This is an Open Access article distributed under the terms of the Creative Commons Attribution License (http://creativecommons.org/licenses/by/4.0/), which permits unrestricted use, distribution, and reproduction in any medium, provided the original work is properly cited.

**Introduction/Aim**: More than half of chronic pain patients suffer from other chronic health conditions. This study aims to 1) examine the degree to which pain management is a priority among individuals with multiple medical conditions and 2) identify predictors of pain prioritization.

**Methods**: The sample is comprised of 99 patients suffering from chronic pain (≥ 3 months) and ≥ 1 other medical condition recruited through patient associations and social/conventional media. Self-report questionnaires (pain characteristics, patient characteristics, type and severity of comorbidities) were completed at baseline and a pain prioritization diary was completed twice weekly for 6 weeks. Univariate regression models were used to identify pain, patient and comorbidity variables (*p *> 0.20) that would be included in a final multivariate model to examine pain management priority (model 1) and stability of prioritization (model 2).

**Results**: Pain management prioritization scores ranged from 1.67 to 4.92 out of 5 (SD = 0.76) and 16.2% of participants reported ≥ 20% increase or decrease in the priority given to pain management over a six-week period. Model 1: Perceived illness burden was the only predictor retained in the univariate analysis (*p *= 0.074) to predict levels of pain management prioritization. Model 2: None of the characteristics examined were significantly associated with stability of prioritization of pain management symptoms.

**Discussion/Conclusions**: Prioritization of pain management is a dynamic process that does not seem to be influenced by severity of pain, individual characteristics or severity of comorbidities. The next step is to examine time-varying predictors of pain management prioritization.

### Informing an ‘All Hands on Deck’ Approach to the Opioid Crisis: A Review of Strategies to Prevent and Reduce Opioid-Related Harms

Nancy Carnide^a^, Morgane Le Pouésard^a^, Emma Irvin^a^, Dwayne Van Eerd^a^, Heather Johnston^a^, Quenby Mahood^a^, Margaret Tiong^a^, Zoe Sinkins^b^, Sara Macdonald^a^, Maria-Laura Santos, and Andrea D. Furlan^a^^c^^d^

^a^Institute for Work & Health; ^b^McMaster University, Hamilton, Ontario, Canada; ^c^Department of Medicine, University of Toronto, Toronto, Ontario, Canada; ^d^Toronto Rehab-University Health Network, Toronto, Ontario, Canada

**CONTACT** Nancy Carnide ncarnide@iwh.on.ca

© 2019 The Author(s). Published with license by Taylor & Francis Group, LLC.

This is an Open Access article distributed under the terms of the Creative Commons Attribution License (http://creativecommons.org/licenses/by/4.0/), which permits unrestricted use, distribution, and reproduction in any medium, provided the original work is properly cited.

**Introduction/Aim**: Canada is in the middle of a public health crisis related to opioids. Prior attempts to overcome the crisis in Canada have been insufficient as the number of people dying continues to increase.

**Methods**: This review was funded by the Canadian Institutes of Health Research (CIHR) to identify emerging strategies for subgroups at highest risk of opioid-related harms and likely to benefit most from the implementation of these strategies. Systematic methods were used to search, select, critically appraise and synthesize published and unpublished studies conducted in the past five years

**Results**: The research team found 46 studies of high and medium quality that reported on a variety of emerging strategies to tackle the opioid crisis. A sufficient number of recent high- and moderate-quality studies on novel approaches to address Canada’s opioid crisis suggest a number of evidence-based prevention strategies are available. They include various clinical practice, educational and regulatory/policy strategies to improve opioid prescribing practices, and various clinical practice strategies to reduce opioid-related emergency department visits. Too few recent studies of sufficient quality and consistency in their findings are available to suggest promising new treatment, harm reduction and enforcement strategies.

**Discussion/Conclusions**: Evidence-based prevention strategies that have not yet been widely used in Canada should be considered to help tackle the opioid crisis.

### Cortical Neuroplasticity after Focused Peripheral Radiation: Longitudinal Effects of Gamma Knife Radiosurgery for Classic Trigeminal Neuralgia

Peter Shih-Ping Hung^a^, Alborz Noorani^a^, Jia Y. Zhang^b^, and Mojgan Hodaie^c^

^a^Institute of Medical Science, University of Toronto, Toronto, Ontario, Canada; ^b^Krembil Research Institute, University Health Network, Toronto, Ontario, Canada; ^c^Department of Surgery, Division of Neurosurgery, University of Toronto, Toronto, Ontario, Canada

**CONTACT** Peter Shih-Ping Hung peter.hung@mail.utoronto.ca

© 2019 The Author(s). Published with license by Taylor & Francis Group, LLC.

This is an Open Access article distributed under the terms of the Creative Commons Attribution License (http://creativecommons.org/licenses/by/4.0/), which permits unrestricted use, distribution, and reproduction in any medium, provided the original work is properly cited.

**Introduction/Aim**: Classic trigeminal neuralgia (TN) is a severe chronic neuropathic facial pain disorder that, despite frequently being linked to microstructural changes within trigeminal nerve root entry zone, has well-documented cortical thickness alterations. These alterations occur in brain regions important for sensory and affective processing of pain and can be reversed by successful surgical interventions. As existing studies are limited to pre-treatment and early post-treatment time-points, longitudinal effects of non-invasive surgical interventions like Gamma Knife radiosurgery (GKRS) on the cortical thickness in TN patients remains unclear and are thus the focus of our current structural magnetic resonance imaging study.

**Methods**: 18 patients treated with GKRS as their first surgery for TN underwent pre-treatment, 6 months post-treatment, and 12 months post-treatment 3-Tesla imaging. FreeSurfer 6.0 facilitated extraction of cortical thickness from 68 Desikan-Killiany brain regions. 7 false discovery rate-corrected paired Student’s t-tests were then conducted per region to contrast cortical thicknesses between hemispheres and across time-points.

**Results**: GKRS resulted in longitudinal, bilateral increases in cortical thickness within fusiform gyrus, precentral gyrus, paracentral lobule, and inferior temporal gyrus. GKRS also resulted in transient increases in cortical thickness in contralateral banks of superior temporal sulcus, ipsilateral superior frontal gyrus, and contralateral caudal middle frontal gyrus.

**Discussion/Conclusions**: For the first time, we demonstrated that radiosurgery for TN leads to longitudinal and transient cortical thickness changes within default mode, executive control, limbic, somatomotor, and visual brain networks. Further studies are needed to ascertain the functional role these structural changes may play in GKRS’ therapeutic effect.

### Prevalence and Intensity of Persistent Post-Surgical Pain following Breast Cancer Surgery: A Systematic Review and Meta-Analysis of Observational Studies

Li Wang 0000-0003-1585-8846^a^, Niveditha Devasenapathy^b^, Brian Y. Hong^c^, Jared Cohen^d^, Sasha Kheyson^d^, Yvgeniy Oparin^d^, Sean Alexander Kennedy 0000-0002-4970-8152^e^, Beatriz Romerosa^f^, Nikita Arora^d^, Henry Kwon^g^, Daniel Lu^d^, Kate Jackson^d^, Allen Li^d^, Giuliana Guarna^d^, Rachel Couban^h^, and Jason W. Busse 0000-0002-0178-8712^h^

^a^Anesthesia, McMaster University, Hamilton, Ontario, Canada; ^b^Public health Foundation of India, Indian Institute of Public Health -Delhi, Gurgaon, India; ^c^Plastic Surgery, University of Toronto, Toronto, Ontario, Canada; ^d^McMaster University, Hamilton, Ontario, Canada;; ^e^Department of Medical Imaging, University of Toronto; ^f^Department of Anesthesia and Critical Care, University Hospital of Toledo, Toledo, Spain; ^g^Wayne State University School of Medicine, Detroit, MI, USA; ^h^Department of Anesthesia, McMaster University, Hamilton, Ontario, Canada

**CONTACT** Li Wang lwang246@gmail.com

© 2019 The Author(s). Published with license by Taylor & Francis Group, LLC.

This is an Open Access article distributed under the terms of the Creative Commons Attribution License (http://creativecommons.org/licenses/by/4.0/), which permits unrestricted use, distribution, and reproduction in any medium, provided the original work is properly cited.

**Introduction/Aim**: Persistent post-surgical pain (PPSP) is a common complication after breast cancer surgery; but reported prevalence rates range from 10% to 69%. We conducted a systematic review to address this uncertainty.

**Methods**: We searched MEDLINE, EMBASE, CINAHL and PsycINFO from inception to October 2018, to identify observational studies that reported the prevalence and intensity of PPSP after breast cancer surgery. We performed random effects meta-analysis with Freeman-Tukey transformation for overall, moderate and severe PPSP prevalence, and pooled pain intensity after converting all pain scales to the 10cm VAS.

**Results**: We included 184 observational studies with 298,549 patients. The overall prevalence of any PPSP was 33.6% (95%CI 30.3% to 36.9%). The pooled prevalence of moderate and severe pain was 16.1% (95%CI 12.6% to 19.8%) and 3.7% (95%CI 2.6% to 5.0%) respectively. PPSP prevalence was 36.2% (95%CI 29.5% to 43.2%) at 3 months to 1 year after surgery, 33.7% (95%CI 15.3% to 55.1%) at 1 to 2 years, 26.6% (95%CI 11.3% to 45.5%) at 2 to 4 years and 36.6% (95%CI 21.1% to 53.7%) at 4 years or later. The prevalence of neuropathic pain was 33.7% (95%CI 22% to 46.5%). The average pain intensity on a 10cm VAS was 2.7 (95%CI 2.2 to 3.1).

We found significant subgroup effects between any pain (40.9%, 95%CI 37.4% to 44.6%) vs specific pain (28.8%, 95%CI 24.8% to 33.0%; interaction p<0.001) and low threshold pain (mild to severe pain, 35.3%, 95%CI 31.4 to 39.2%) vs high threshold of pain (moderate to severe pain 24.9%, 95%CI 18.9% to 31.4%; interaction p=0.005). Meta-regression did not show significant association between PPSP and length of follow-up, proportion of loss to follow-up, proportion of breast conserving surgery, radiotherapy and chemotherapy.

**Discussion/Conclusions**: PPSP after breast surgery is common and affects approximately 1 in 3 women undergoing this procedure. Of those who develop persistent pain, 48% will report at least moderate pain and 11% will experience severe pain although the average pain is mild.

### Performance Metrics of a Multimedia Web-Based Platform for Dissemination of Knowledge about Cardiac Pain

Michael McGillion^a^, Carley Ouellette^a^, Sheila O’Keefe-Mccarthy 0000-0003-2909-8286^b^, Kathleen Dallessio^c^, Shaunattonie Henry^d^, and Judy Watt Watson^e^

^a^School of Nursing, McMaster University, Hamilton, Ontario, Canada; ^b^Brock University, St. Catherines, Ontario, Canada; ^c^Elesvier, Milton, Ontario, Canada; ^d^Faculty of Health Sciences, School of Nursing, McMaster University, Hamilton, Ontario, Canada; ^e^Faculty of Nursing, University of Toronto, Toronto

**CONTACT** Michael McGillion mmcgill@mcmaster.ca

© 2019 The Author(s). Published with license by Taylor & Francis Group, LLC.

This is an Open Access article distributed under the terms of the Creative Commons Attribution License http://creativecommons.org/licenses/by/4.0/, which permits unrestricted use, distribution, and reproduction in any medium, provided the original work is properly cited.

Persistent forms of cardiac pain, such as refractory angina, are difficult to treat and debilitating. We aimed to disseminate a web-based, multi-media resource centre for persistent forms of cardiac pain, CardiacPain.Net, as a vehicle for global-scale knowledge dissemination.

Conceptualization of CardiacPain.Net was based on the Canadian Institutes of Health Research (CIHR) knowledge to action cycle as well as the CIHR framework for citizen engagement. Content curated on the site for dissemination was categorized according to specific aspects of knowledge inquiry, synthesis, as well as knowledge product tools (e.g. clinical practice guidelines, diagnostic phenotype criteria). Dissemination strategies included opt-in email blasts to Elsevier’s global readership subscribers, as well as online banner journal advertising. Dissemination metrics included total site visits and components visited and downloaded. Customized metrics included unique and return visitor rates, bounce rate, stream views, unique end-user tracking, as well as geo-targeting. Metrics were analyzed in aggregate and distributions were examined for monthly trends. The total dissemination period was for 4 years (2014 to 2018). We aimed to engage 1,000 new end users per month.

Evaluation of our dissemination metrics found that CardiacPain.Net reached a total of 48,519 unique end users. Users engaged in a total of 53,927 sessions on the site, with a total of 72,794 page views. An average of 1.39 pages were viewed per user session. Geo-targeting analyses found that end-user engagement spanned 164 countries across 5 continents.

Our results suggest that a globally linked multimedia resource centre is an effective vehicle for widespread pain knowledge dissemination.

### Characterization of Key Sexually Dimorphic Regulators in Pain Processing

Shahrzad Ghazisaeidi^a^, Arun Ramani^b^, Parisa Shooshtari^b^, Amy Tu^c^, Katherine Halievski^c^, David Finn^d^, Sofia Assi^a^, Milind Muley^c^, Vivian Wang^c^, Ameet Sengar^c^, Rosanna Weksberg^e^, Michael Brudno 0000-0001-7947-2243^b^, and Michael W Salter^c^

^a^Physiology, University of Toronto, Toronto, Ontario, Canada; ^b^Centre for Computational Medicine, The Hospital for Sick Children, Toronto, Ontario, Canada; ^c^Neurosciences and Mental Health, The Hospital for Sick Children, Toronto, Ontario, Canada; ^d^Pharmacology & Therapeutics, National University of Ireland, Galway, Ireland; ^e^Genetics & Genome Biology, The Hospital for Sick Children, Toronto, Ontario, Canada

CONTACT Shahrzad Ghazisaeidi shahrzad.ghazisaeidi@mail.utoronto.ca

© 2019 The Author(s). Published with license by Taylor & Francis Group, LLC.

This is an Open Access article distributed under the terms of the Creative Commons Attribution License (http://creativecommons.org/licenses/by/4.0/), which permits unrestricted use, distribution, and reproduction in any medium, provided the original work is properly cited.

**Introduction/Aim**: Chronic pain affects 1 in 5 Canadians and costs over $43B annually, yet effective and safe treatment options remain elusive. Recent discoveries have brought to the forefront sex differences in mechanisms of pain as a potential explanation why novel pre-clinical therapeutics have not translated into successfully in clinical trials.

**Methods**: To begin understanding how males and females differ in pain processing, we analyzed gene expression, using RNA sequencing, and DNA methylation, using reduced representation bisulfite sequencing (RRBS), in rodent models of neuropathic pain.

**Results**: Across both sexes, our data reveals peripheral nerve injury (PNI) caused upregulation of 61 genes involved in innate immune responses in spinal cord. In females specifically, we observed PNI-induced downregulation of 5 genes involved in neuronal function and upregulation of two classes of Cathepsins. (C and E). On the other hand, in males, we observed upregulation of 14 genes including those involved in metabolism of purines and glutathione. Additionally we found that PNI leads to methylome remodeling in a sexually dimorphic manner: 125 promoters in rat spinal cord that were differentially methylated in injured males versus females.

**Discussion/Conclusions**: Our data shows robust sex specific DNA methylation and transcriptome signature after PNI. Additionally, our findings leads to the hypothesis that remapping of DNA methylation, with subsequent alterations in the transcriptome, are critically involved in the development of neuropathic pain. We anticipate that future research directed at understanding these differences may lead to effective drug development to combat chronic pain.

### Opioid Receptors and Brain Function

Brigitte L. Kieffer

**CONTACT** Brigitte L. Kieffer brigitte.kieffer@douglas.mcgill.ca

© 2019 The Author(s). Published with license by Taylor & Francis Group, LLC.

This is an Open Access article distributed under the terms of the Creative Commons Attribution License (http://creativecommons.org/licenses/by/4.0/), which permits unrestricted use, distribution, and reproduction in any medium, provided the original work is properly cited.

Opiates have been used since thousand years for their remarkable pain-relieving and rewarding properties. Opiates produce their potent effects by activating opioid receptors in the brain, highjacking an endogenous opioid system, which is central to hedonic and mood homeostasis. Recently, revolutions in G protein-coupled receptor research, fascinating developments in basic neuroscience and the rising opioid crisis have propelled opioid receptors back on stage. This presentation will discuss rapidly evolving areas in opioid receptor research for addiction, including the key question of whether we can we kill pain without addiction using mu opiates, and how opioid receptors operate within the neurocircuitry of addiction. Recent work linking mu opioid receptor gene and drug activities to whole-brain functional networks, using by fMRI in mice, will also be presented.
